# Taming Keteniminium Reactivity by Steering Reaction Pathways: Computational Predictions and Experimental Validations

**DOI:** 10.1021/jacs.2c09146

**Published:** 2022-12-16

**Authors:** Mark A. Maskeri, Anthony J. Fernandes, Giovanni Di Mauro, Nuno Maulide, K. N. Houk

**Affiliations:** † Department of Chemistry and Biochemistry, University of California, Los Angeles, California 90095, United States; ‡ Institute of Organic Chemistry, University of Vienna, Währinger Straße 38, Vienna 1090, Austria

## Abstract

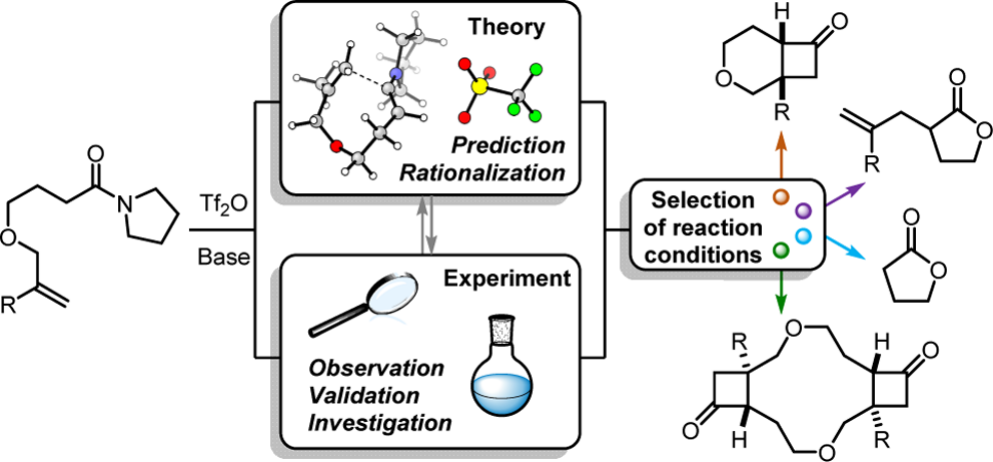

Keteniminium ions, the nitrogen analogues of ketenes, exhibit high reactivity toward olefins and π-systems. Previous results from the Maulide group demonstrated an unexpected propensity for an alternative intramolecular Belluš–Claisen-type rearrangement rather than an expected intramolecular (2 + 2) cycloaddition. We have conducted a cooperative density functional theory/experimental investigation of this process, seeking insights into the competition between the observed Claisen-type reaction and the historically expected (2 + 2) cyclization. Our calculations revealed a surprisingly small difference in the free energy barrier between these two intramolecular reactions. Further theoretical and experimental investigations probe the electronics of the substrate, rationalize a competing deallylation side reaction, and demonstrate the proof-of-concept for an enantioselective (2 + 2) variant.

## Introduction

**Scheme 1:**
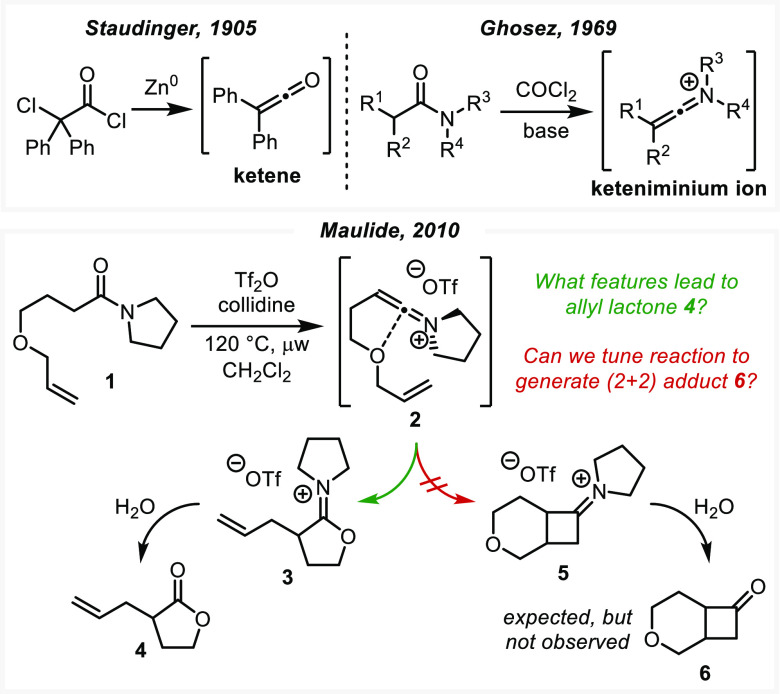
Top: Early Disclosures of the Ketene and Keteniminium Ions; Bottom: Maulide’s Unexpected Belluš–Claisen-Type Rearrangement to **4** and the Expected Bicyclic Cyclobutanone-Tetrahydropyran **6**

Ketenes hold a storied place in the organic chemist’s toolbox.^1−3^ Since Staudinger’s disclosure of the species in 1905 (Scheme 1, top left),^4^ ketenes have enabled the synthesis of cyclobutanone scaffolds through a prototypical thermal (_π_2_s_+_π_2_a_) reaction with olefins^5−7^ and have also served as critical rearrangement intermediates (e.g., Wolff rearrangement,^8,9^ among numerous others^1,2,10^). This prodigious reactivity is, however, often detrimental, as the desired ketene transformation may be plagued by side reactivity or dimerization.^11−13^ The generation of ketenes also often requires the use of sensitive intermediates (e.g., acyl chlorides) or high temperatures, which may pose challenges in complex-target synthesis or when delicate substrates are employed.

A variety of ketene equivalents and analogues have been formulated to temper errant reactivity and enable access to ketene-like behavior from convenient functional groups.^14−17^ The keteniminium ion, introduced by Ghosez and co-workers, represents a particularly convenient equivalent (Scheme 1, top right).^18−23^ Through the action of an electrophile (such as phosgene or, more typically, triflic anhydride) and a base, otherwise stalwart carboxamides can be transiently converted to keteniminium ions and subsequently engaged in polar chemistry. In addition to the ease of generation, keteniminium ions are less prone to dimerization and offer a particularly useful handle to manipulate selectivity through additional bonds to nitrogen.^24^

Seeking to leverage the reactivity and selectivity of keteniminium ions in a total synthesis campaign, the Maulide group in 2010 attempted the synthesis of cyclobutanone-tetrahydropyran bicycle **6** (Scheme 1, bottom).^25−27^ However, unlike related literature precedents for this reaction, the presence of an ethereal oxygen instead triggered a Belluš–Claisen-type rearrangement, furnishing allyl γ-lactones (**4**).^19,28,29^ None of the classic (2 + 2) product **6** was reported, and we sought to understand the factors controlling these two competing intramolecular reactions. In the process of our investigation, we engaged in a computational/experimental collaboration. The synergy between experiment and theory enabled a thorough investigation of the cycloaddition capacity of this reaction and uncovered methods by which the chemoselectivity of the reaction can be dramatically altered.

## Methods

Quantum mechanical investigations of the reactions of keteniminium ion **2** and congeners were conducted with density functional theory (DFT) calculations using Gaussian 16.^30^ Initial geometries were prepared with Grimme’s xTB^31^/CREST^32^ and Zimmerman’s GSM^33^ codes. Geometry optimization was completed at the ωB97X-D/def2-SVP level with the solvation model based on density (SMD) for dichloromethane.^34−36^ Single-point corrections to energy were made at the ωB97X-D/def2-TZVPP level with the SMD for dichloromethane. Quasiharmonic corrections to enthalpy and entropy were made using Paton’s GoodVibes software.^37−39^ Temperature corrections were applied at 393.15 K (120 °C) unless otherwise specified. Visualizations were prepared with Legault’s CYLview20^40^ and Gilbert’s IQmol. Please see the Supporting Information for experimental details.

## Results and Discussion

### Initial Studies

**Figure 1:**
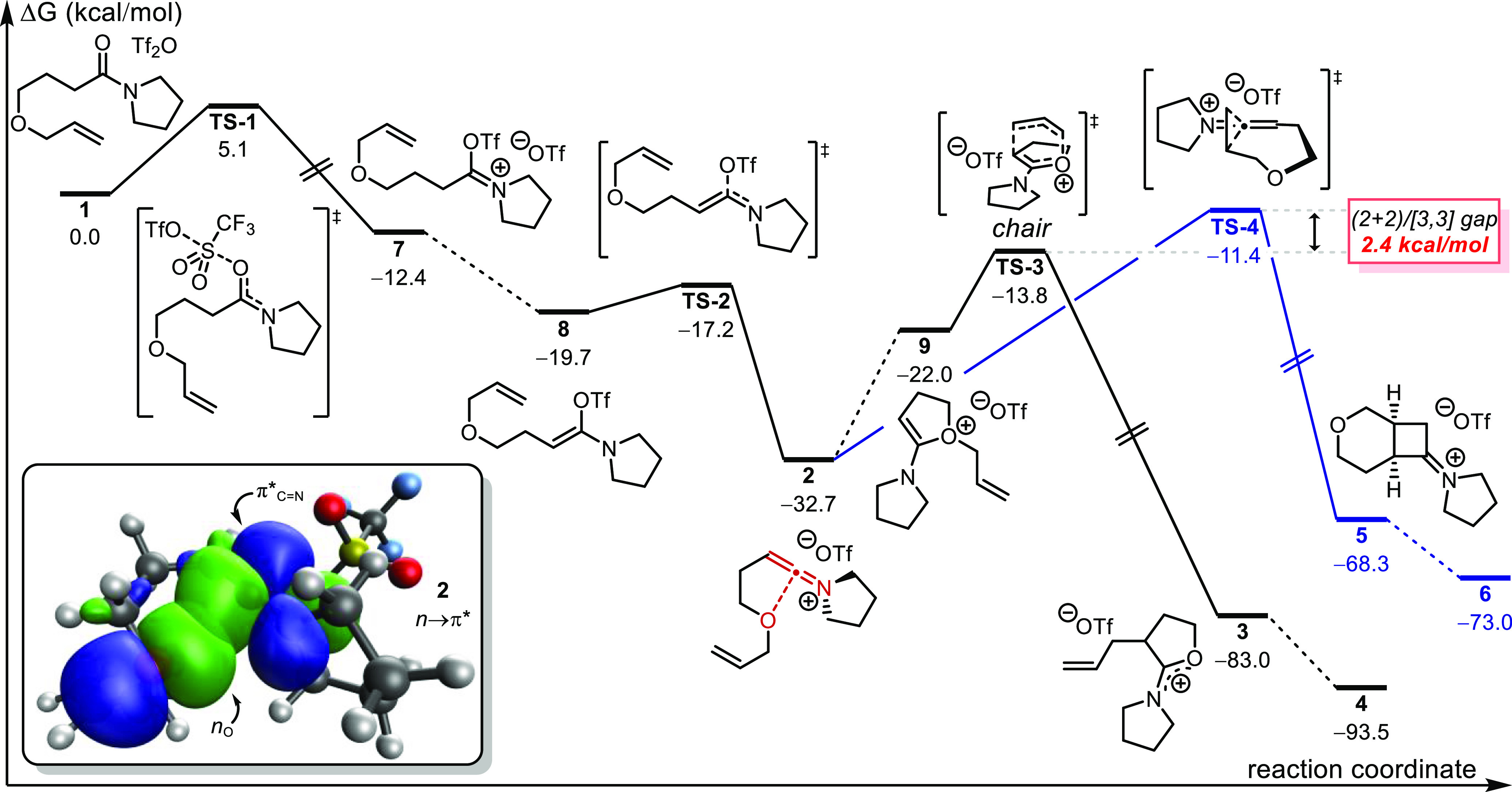
Energetics for the generation of allyl lactone **4** and (2 + 2) adduct **6** from amide **1**. Inset. NBO orbitals of **2** showing *n* → π* interaction. NBO orbitals depicting *n*_O_ (orbital 79, HOMO–7) and π*_C=N_ (orbital 87, LUMO) are drawn at an isovalue of 0.05. Geometries optimized at the ωB97X-D/def2-SVP/SMD(CH_2_Cl_2_) level of theory. Single-point corrections and NBO orbital generation were performed at the ωB97X-D/def2-TZVPP/SMD(CH_2_Cl_2_) level of theory. Energies are given in kcal/mol. TSs for the conversion of **7** to **8** and of **2** to **9** can be found in section 5.5 of the Supporting Information.

Our computational investigations began with the mechanism proposed by Maulide et al. for the conversion of amide **1** to allyl lactone **4** (Figure 1).^25^ We located intermediates and transition structures (TSs) with favorable energetics proceeding from the parent amide to the keteniminium ion **2**. The reversible addition of the collidine base to **2** was also established, with the resulting adduct demonstrating substantial stabilization of the keteniminium ion (see Supporting Information, section 5.3).^41,42^ Unlike the generation of keteniminium ions from ynamides—which is known to be endergonic^43^—this process is considerably exergonic (Δ*G* = −32.7 kcal/mol; see Supporting Information section 5.4 for ynamide model study). Keteniminium ion **2** adopts a low-energy conformation where the ethereal oxygen interacts with the π* orbital (*n* → π*, Figure 1 inset);^44^ this conformation essentially preorganizes the scaffold to proceed to the key allyloxonium ion species **9** (ΔΔ*G*^⧧^ = 10.7 kcal/mol; see Supporting Information, section 5.5).^43,45,46^

From **9**, we located Claisen-type TSs for the allyl rearrangement, the lower-energy chair conformer of which exhibits a reasonable energy barrier of 18.9 kcal/mol (**TS-3**).^47^ This keteniminium ion **2** also permitted us to identify a (2 + 2) TS (**TS-4**), proceeding to the *cis*-cyclobutane iminium ion **5** (*cis*-(2 + 2) adduct), that is only 2.4 kcal/mol higher in energy than the Claisen chair (see inset in Figure 1). This free energy difference is consistent with the previous report that only lactone product **4** was observed.^48^ However, 2.4 kcal/mol is not a particularly large free energy gap, and we hypothesized that small perturbations to the olefin electron density may promote (2 + 2) reactivity over the Claisen-type rearrangement.

### Obtaining (2 + 2) Products

**Scheme 2:**
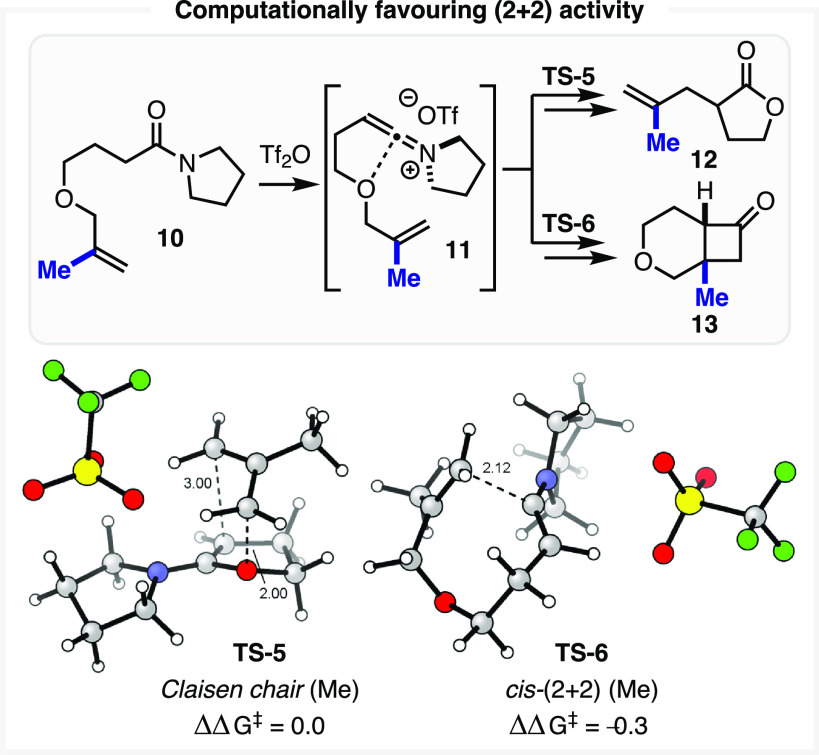
Selected Energetics for Methyl Olefin Substrate **10** Computed with ωB97X-D/def2-TZVPP/SMD(CH_2_Cl_2_)//ωB97X-D/def2-SVP/SMD(CH_2_Cl_2_), reported in kcal/mol.

We next studied the methallyl congener **10**, which exhibited an energetic profile similar to that of the unsubstituted parent substrate **1**, until the formation of the keteniminium ion **11** (see Supporting Information, section 5.6). Following on from **11**, we were gratified that the minor inductive electron donation of the methyl group was sufficient to remove the relative energy barrier between the chair sigmatropic rearrangement and the *cis*-(2 + 2) TSs (ΔΔ*G*^⧧^ = −0.3 kcal/mol, Scheme 2).

**Scheme 3:**
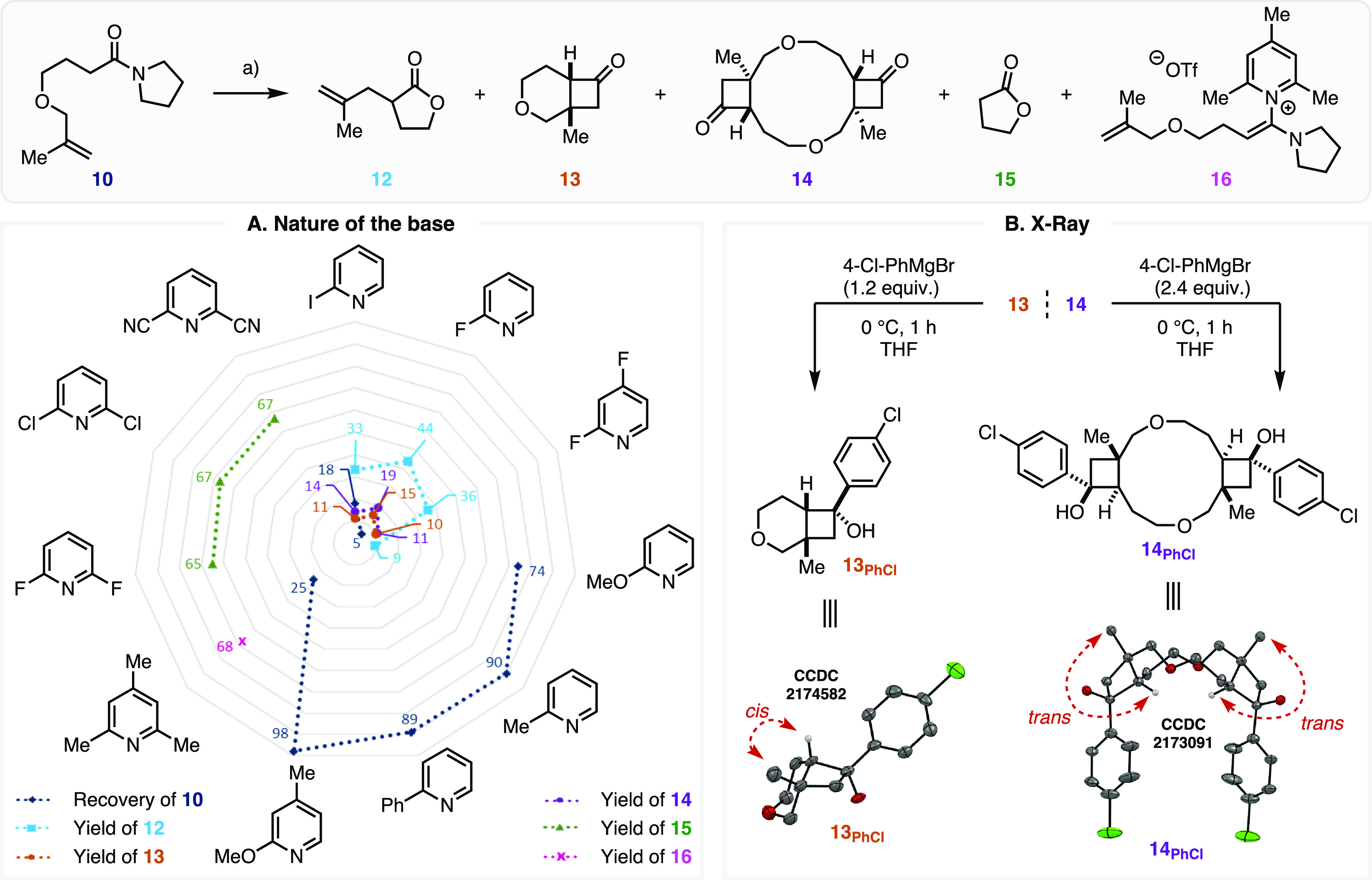
(A) Experimental Confirmation of (2 + 2) Adduct Formation and (B) XRDS Structure of **13**_**PhCl**_ and **14**_**PhCl**_ Obtained after Derivatization through Grignard Reagent Addition (a) Pyridine base (2.2 equiv), Tf_2_O (1.1 equiv), 0–20 °C, 4 h, then NaHCO_3__(aq)_, 20 °C, (16 h); (b) Plot exhibits NMR yields and recovery (%) using 1,3,5-trimethoxybenzene as the internal standard (IS).

While the initial report of this reaction for methyl olefin substrate **10** did not identify any of the (2 + 2) bicyclic product **13**—instead reporting a 61% yield of allyl lactone **12**(25)—the corresponding essentially isoenergetic transition structures (**TS-5** and **TS-6**, Scheme 2) suggest that the (2 + 2) reaction should be a competitive pathway. This computational prediction was readily tested experimentally. Gratifyingly, conducting the experiment utilizing modern, mild amide activation procedures^49^ (Scheme 3A, 2-fluoropyridine at 20 °C; see Supporting Information, section 2.3) allowed us to obtain a 15% yield of the *cis*-(2 + 2) cycloaddition product **13**. This result confirms that it is possible to steer the reaction pathway by modulating the olefin electronic traits. Notably, subjecting the unsubstituted olefin substrate **1** to these conditions provided an 85% NMR yield of the corresponding allyl lactone **4** with no evidence of the corresponding (2 + 2) cycloadduct **6**. A significant byproduct exhibiting NMR spectroscopic signals indicative of a *trans*-(2 + 2) cycloaddition product was also identified. Both **13** and this unexpected byproduct resisted crystallization but were successfully identified by X-ray diffraction spectroscopy (XRDS) of the crystalline derivatives obtained from the addition of a Grignard reagent (Scheme 3B, **13**_**PhCl**_ and **14**_**PhCl**_). This analysis confirmed the monomeric *cis*-(2 + 2) identity of **13** and identified the unknown byproduct as a dimeric species with a *trans*/*trans* tricyclic core (14).

When the activation of **10** was repeated using different pyridine bases (Scheme 3A), several interesting trends emerged. While electron-rich pyridines did not lead to significant conversion of **10**, highly electron-deficient pyridines led to the formation of γ-butyrolactone **15** via deallylation—as is the case in absence of a base.^50^ At the 20 °C temperature utilized for these studies, sterically crowded collidine produced the base-stabilized adduct **16**.^42^ When bases of intermediate basicity and steric hindrance were employed, both (2 + 2) adducts **13** and lactone **12** were formed along with dimerized product **14**. The halopyridines—particularly 2-fluoropyridine—appear appropriately tuned to the desired (2 + 2) behavior with regard to basicity and steric encumbrance.

**Figure 2:**
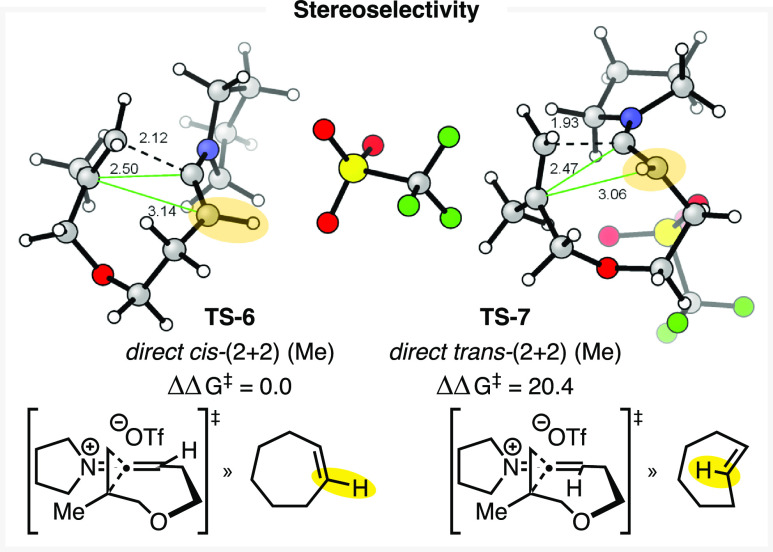
Direct transition structures for the generation of monomeric (2 + 2) adducts ***cis*-13** and ***trans*-13**. Analogous cycloheptene isomers illustrate the relative strain between the transition structures. Energies vs **TS-6**, ωB97X-D/def2-TZVPP/SMD(CH_2_Cl_2_)//ωB97X-D/def2-SVP/SMD(CH_2_Cl_2_) in kcal/mol.

While the observed dimer species **14** exhibited only *trans* cyclobutanone ring junctions, no trace of a monomeric *trans*-(2 + 2) adduct was detected. Significant differences manifest on a monomeric basis between the direct *cis*-(2 + 2) (Figure 2, **TS-6**) and *trans*-(2 + 2) (Figure 2, **TS-7**), whereby the *trans*-(2 + 2) is found to be higher in energy than its diastereomer by a barrier surpassing 20 kcal/mol. This can be rationalized by analogy to *cis*- and *trans*-cycloheptene, the geometries of which comprise the cores of these transition structures. These consequently exhibit a similar difference in energy, as can be identified by the positions of the *exo*- and *endo*-cyclic olefin protons (Figure 2, highlight).^51^ Additionally, the resulting *trans* adduct is 12.7 kcal/mol less stable than the *cis*, in line with the very few examples of related *trans*-fused small ring-containing bicycles previously reported in the literature.^29,52−54^

**Scheme 4:**
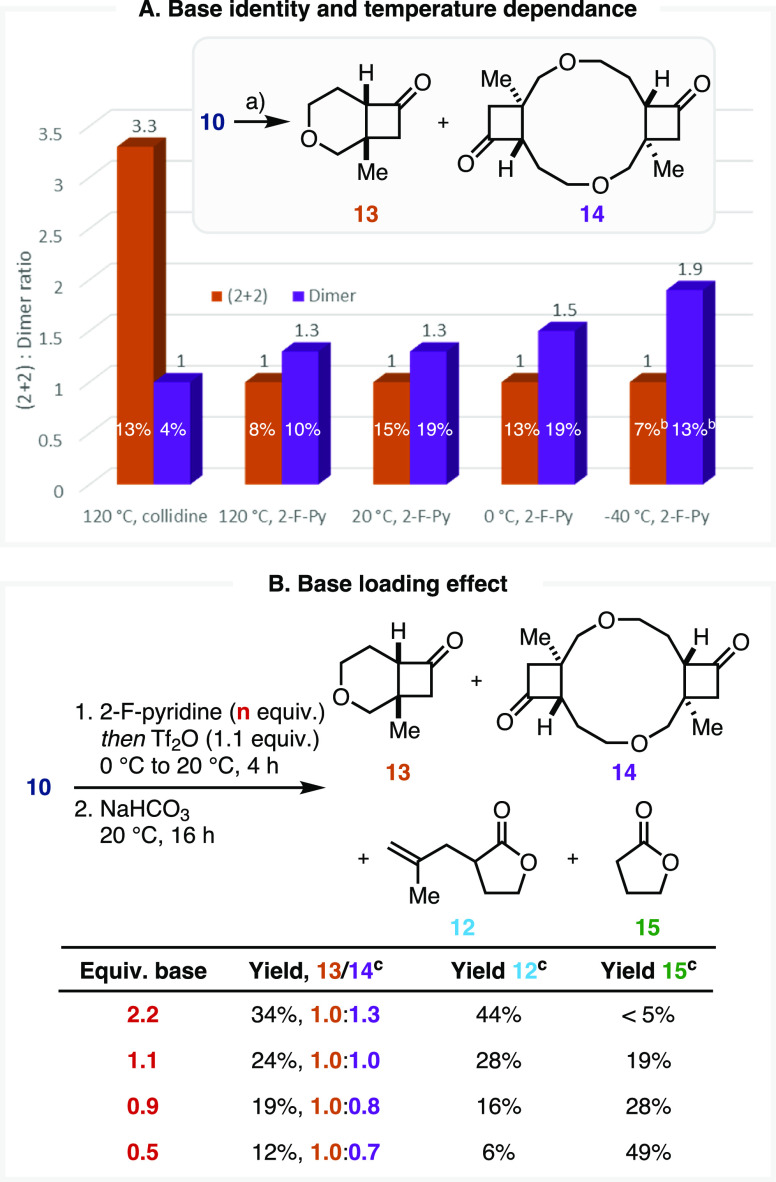
(A) Temperature and Base Identity Dependence of **13**:**14** Ratio; (B) Influence of Base Loading on the **13**:**14** Ratio and Product Distribution (a) Pyridine base (2.2 equiv), Tf_2_O (1.1 equiv), 120 °C (μw), 5 min, or temperature, 4 h, then NaHCO_3(aq)_, 20 °C, 16 h; NMR yields provided on the bars; (b) At 46% conversion. (c) NMR yields using 1,3,5-trimethoxybenzene as an IS.

We hypothesized that the dimer system is capable of *trans*/*trans* ring fusion as the initial intermolecular interaction does not necessitate ring strain analogous to the monomeric **TS-7**. This is not the whole picture, however, as we observed that different pyridine bases produce temperature-dependent product distributions (Scheme 4A).

### Rationalizing Base Effects

Indeed, employing 2-fluoropyridine favored **14,** while collidine at elevated temperatures favored the formation of **13** (Scheme 4A). Interestingly, the same **13**:**14** ratio was observed when the reaction was performed using 2-fluoropyridine at 20 °C instead of 120 °C, with the dimer product being more favored at even lower temperatures (Scheme 4A). These features indicate that the pyridine base is directly involved in one or more determining steps of the mechanism.

**Figure 3:**
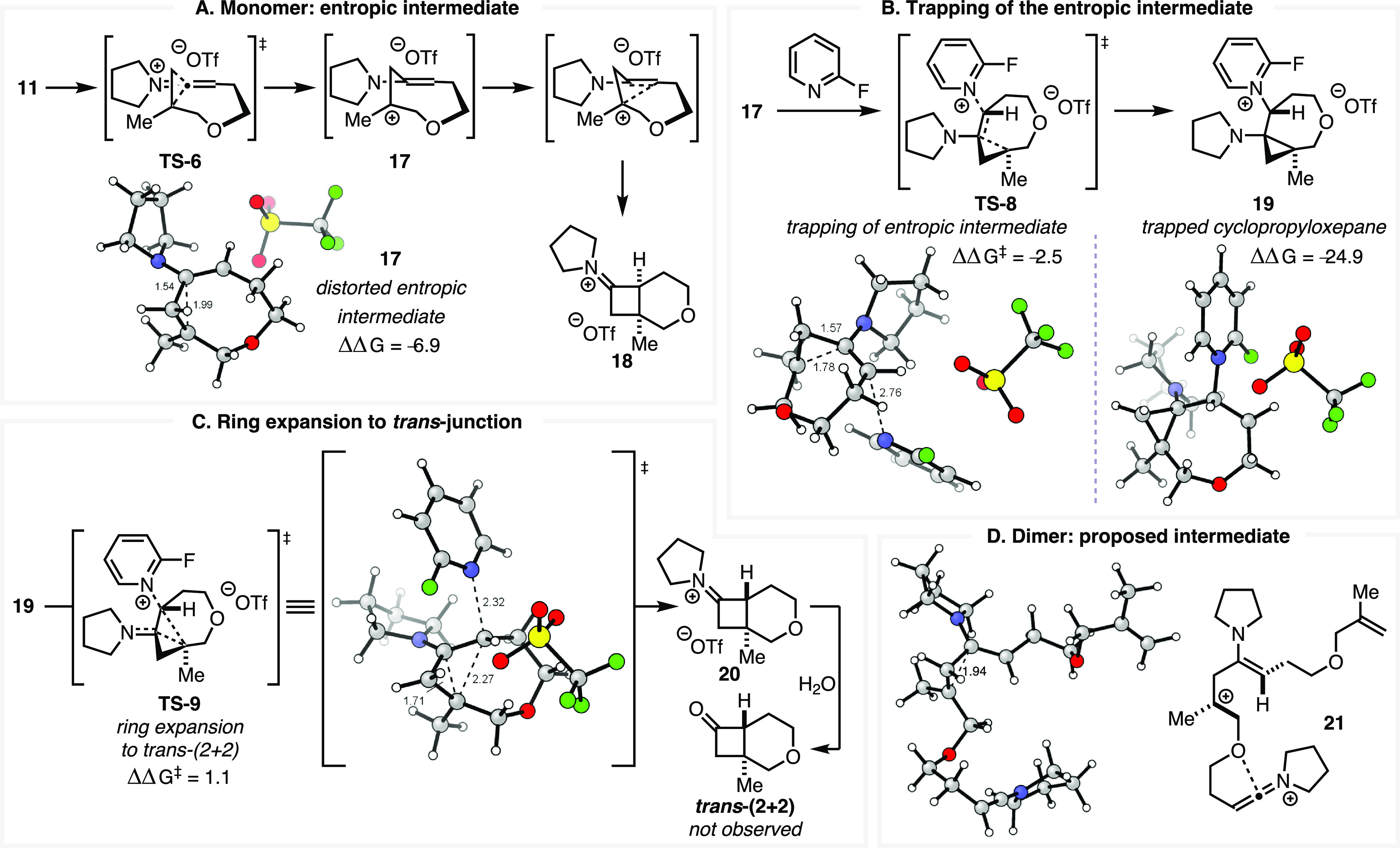
Computed mechanism for the formation of the *trans*-cyclobutane ring junction on the model monomer. (A) Distorted entropic intermediate **17** proceeding from **TS-6**. (B) Trapping of **17** with 2-fluoropyridine. (C) Ring expansion and expulsion of 2-fluoropyridine to produce **20**. (D) Comparison with dimeric species, revealing a stationary point. Triflate counterions omitted from D for clarity. Energies *vs***TS-6** (2-fluoropyridine complex, see the Supporting Information), ωB97X-D/def2-TZVPP/SMD(CH_2_Cl_2_)//ωB97X-D/def2-SVP/SMD(CH_2_Cl_2_).

However, the methyl olefin transition structures **TS-6** and **TS-7** do not require explicit involvement of base and there should be no such kinetic dependence. We, therefore, sought to elucidate potential routes by which an equivalent of a base could intercept these structures. One such possibility arose from our study of the intrinsic reaction coordinate (IRC) of **TS-6**. This IRC illustrates that the two new bonds of the product iminium cyclobutane **18** are formed in an energetically concerted, bonding-stepwise fashion (Figure 3; see Supporting Information, section 5.7). These bond-forming events are connected by a flat region in the IRC—a feature we have referred to in other contexts as an entropic intermediate^55^—that represents the intervening carbenium ion. We identified stationary points nearby the IRC for a distorted analogue of this monomeric entropic intermediate (**17**, Figure 3A) and the analogous dimer system (**21**, Figure 3D) that may be conformationally sampled over the course of the reaction.

**Scheme 5:**
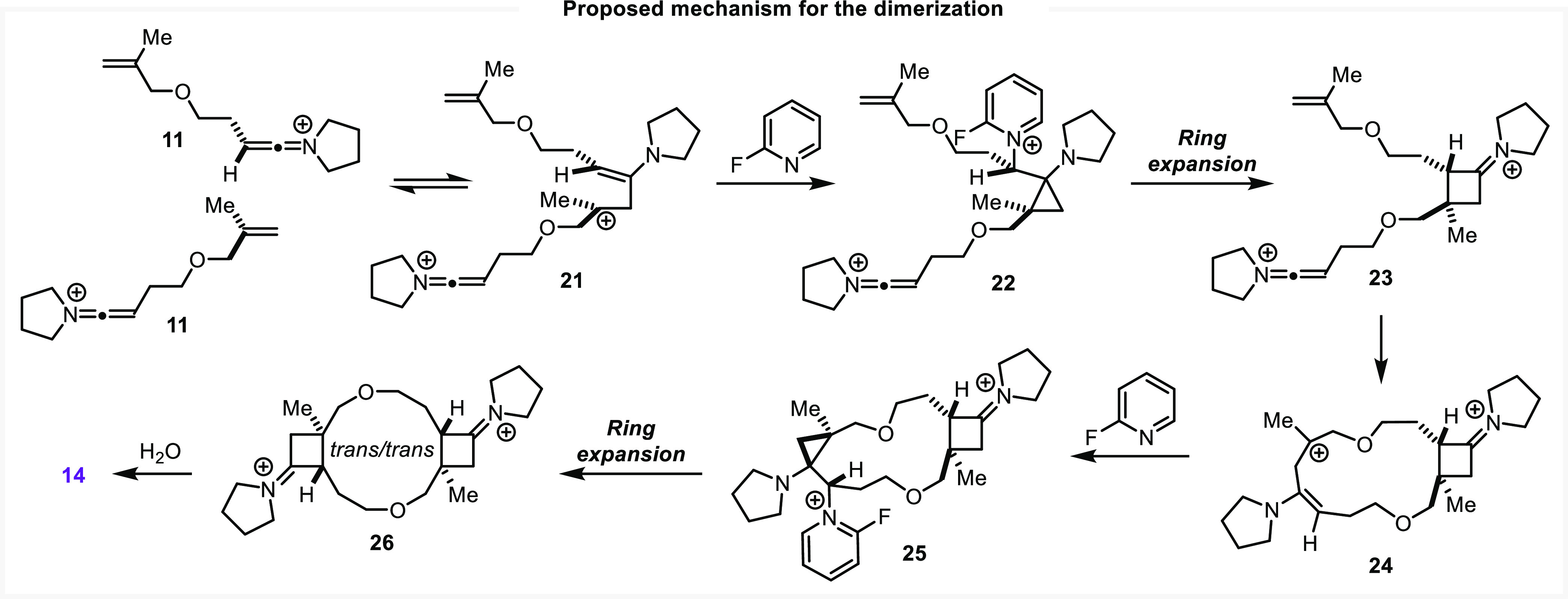
Proposed Mechanism for the Formation of Dimer **14** Based on Monomeric *trans*-(2 + 2) Computational Findings Triflate counterions omitted for clarity.

If such an entropic intermediate were sufficiently kinetically stable, we hypothesized that an equivalent of pyridine base may nucleophilically intercept the cationic species. The resulting complex would adopt the required geometry to produce the observed *trans*-cyclobutane ring junctions. While DFT geometry optimizations of the requisite dimeric TSs and intermediates did not converge, a representative analysis was successfully conducted for the monomeric system (Figure 3B,C; proposed dimer analogue shown in Scheme 5). Starting from distorted entropic intermediate **17**, we located structures representative of a plausible pathway for the generation of the *trans*-(2 + 2) adduct (Figure 3B). Trapping **17** with an equivalent of 2-fluoropyridine produces the pyridinium oxabicyclo[5.1.0]octane species **19** via **TS-8**.

Cyclopropyloxepane **19** presents a conformation analogous to the direct *trans*-(2 + 2) structure **TS-7**, with the pyridinium ring adopting a pseudoequatorial orientation. Simultaneous cyclopropyl ring expansion and expulsion of the pyridine base proceed to the *trans*-cyclobutylidene iminium adduct **20** (Figure 3C).

While the free energy of the expansion TS (**TS-9**) is marginally higher than that of the *cis*-(2 + 2) TS, the small difference in energy suggests that both pathways should be competitive. That the monomeric *trans*-(2 + 2) analogue of **13** is not observed suggests that the homoallylic carbenium ion **17** is *not* sufficiently kinetically stable as hypothesized and is not intercepted by the base at a competitive rate. As the *trans* motif is observed in the dimer species, we propose the dimeric analogue of **17** (**21**) is sufficiently long-lived to interact with the pyridine base and undergo this interception/ring expansion transformation to the observed *trans*/*trans* dimer **14** (Scheme 5).

**Figure 4:**
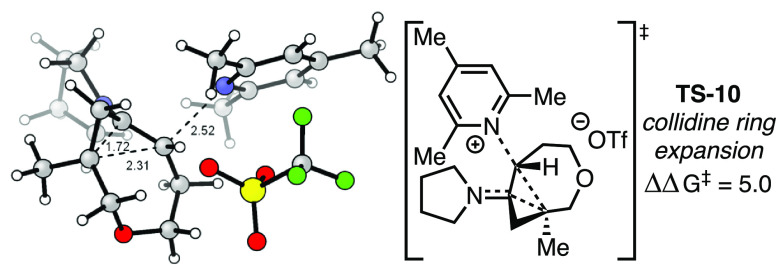
Collidine ring expansion of monomer. Computations with ωB97X-D/def2-TZVPP/SMD(CH_2_Cl_2_)//ωB97X-D/def2-SVP/SMD(CH_2_Cl_2_) in kcal/mol. Energy vs **TS-6** (collidine complex; see Supporting Information, section 5.12 for coordinates).

DFT studies conducted with collidine as the base similarly located **TS-10**, a trapping transition structure analogous to **TS-9** (Figure 4). This TS exhibits considerable steric crowding and is 5.0 kcal/mol higher in energy than the corresponding *cis*-(2 + 2) TS. A similar energy barrier for the dimer system would suggest the *trans*-(2 + 2) dimer should be disfavored. Indeed, this is consistent with our collidine experiments, as the monomeric *cis*-(2 + 2) cycloadduct is favored 3.3:1.^56^ As the formation of **21** should be independent of base, the observed product distribution suggests this initial dimerization is reversible (Scheme 5). An additional consequence of this interception/expansion and reversibility hypothesis is the computational prediction that the monomer/dimer ratio should be dependent on the stoichiometry of the base; a lower concentration of base should yield a higher proportion of *cis*-(2 + 2) adduct **13** over dimer **14** and vice versa. Indeed, experimentally repeating the 2-fluoropyridine-mediated process with different proportions of base confirms this prediction (Scheme 4B).

These base concentration studies also found that substoichiometric base loadings resulted in the appearance of the deallylated γ-lactone side product **15**, a side product that is also observed with the use of highly electron-deficient—and therefore less basic^57^—pyridines (Scheme 3A).

**Scheme 6:**
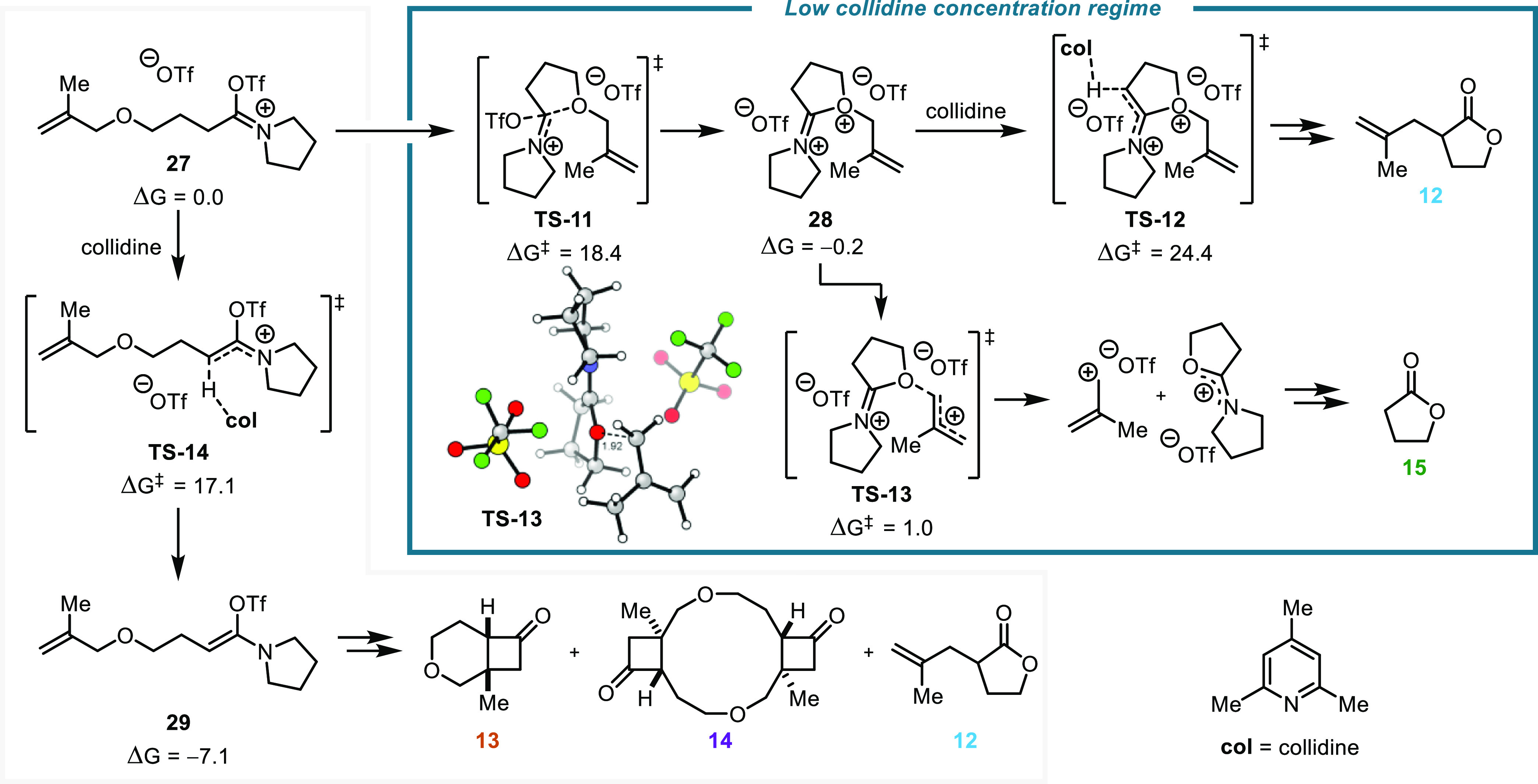
DFT Investigation of the Deallylation Side Reaction (Blue Box) Free energies *versus***27** computed with ωB97X-D/def2-TZVPP/SMD(CH_2_Cl_2_)//ωB97X-D/def2-SVP/SMD(CH_2_Cl_2_) and are reported in kcal/mol. **col** = collidine.

This had been previously observed by the Maulide group, whereby omitting the base entirely demonstrated utility for the synthesis of γ-lactones from amides.^50^ We sought a mechanistic understanding of this side reactivity (Scheme 6) and identified a competing pathway proceeding through the doubly cationic—though surprisingly low-energy—cyclic species **28**. This structure, which readily deallylates to produce an unsubstituted γ-lactone (**15**, ΔΔ*G*^⧧^ = +1.2 kcal/mol) after hydrolysis, is accessed by a cyclization **TS-11** that is expected to be highly competitive with the base-mediated isomerization **TS-14**, key to the above Claisen/(2 + 2) processes (ΔΔ*G*^⧧^ = +1.3 kcal/mol). Kinetically, we would expect this deallylation process to predominate when the relative concentration of the base is low, which corroborates our experimental findings (see Scheme 4).

### Steering toward (2 + 2) Products: the Reactant Olefin as a Handle

**Figure 5:**
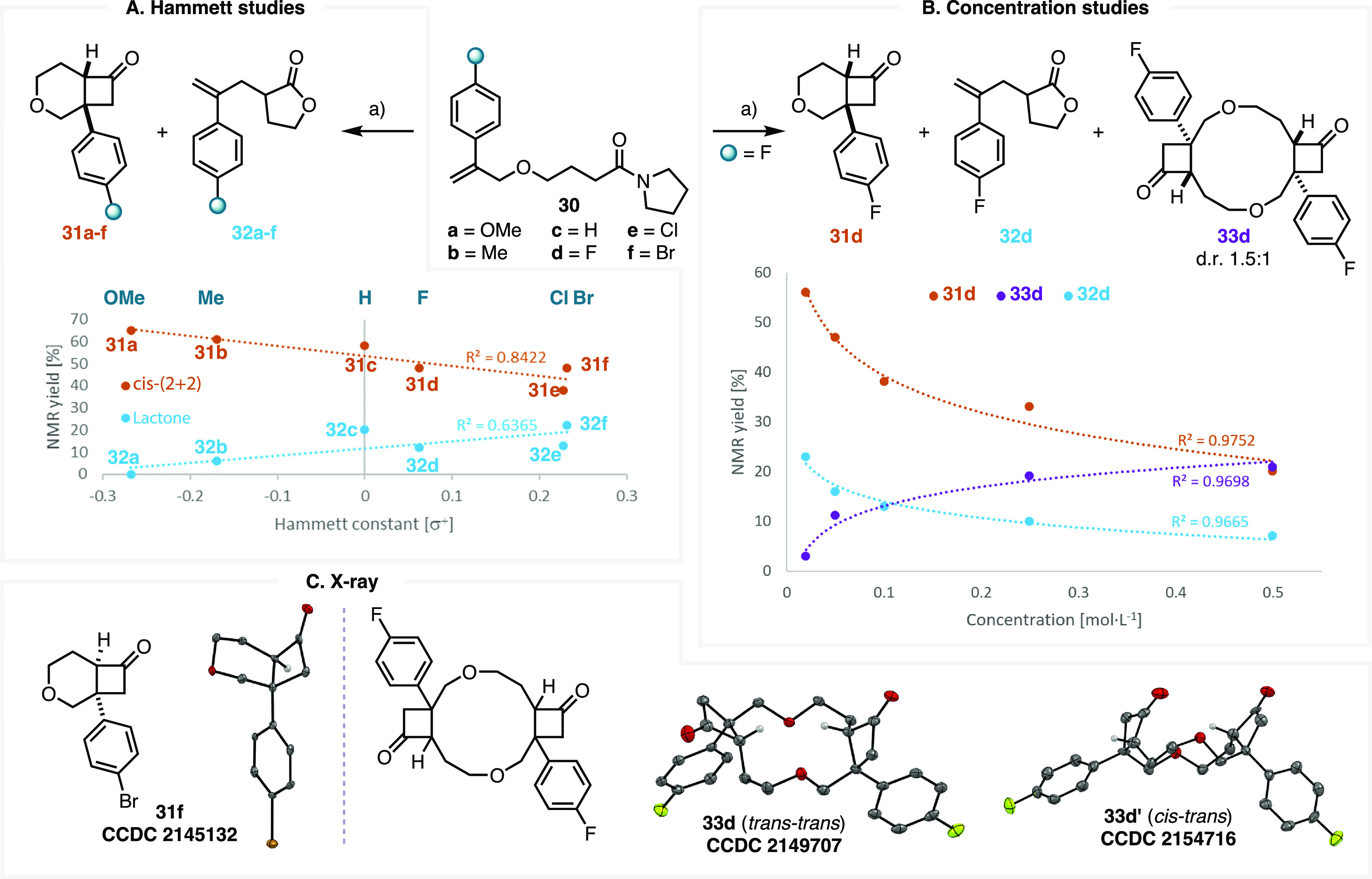
(A) Influence of the electronic nature of the arene on the product distribution. (a) 2-fluoropyridine (2.2 equiv), Tf_2_O (1.1 equiv), 0–20 °C, 4 h, then NaHCO_3(aq)_, 20 °C, (16 h). NMR yields using mesitylene as an IS. (B) Influence of concentration on the product distribution of 4-fluorophenyl substrate **30d**. (C) XRDS structures of **31f**, **33d,** and **33d′**.

Having successfully altered the chemoselectivity of this keteniminium ion cascade, we hypothesized that additional increases in the olefin electron density may further favor the formation of the (2 + 2) adduct. We sought to test this using substituted aryl olefin derivatives (**30a**–**f**, Figure 5A). DFT calculations conducted on derivatives **30a** and **30c** suggest the barrier to the *cis*-(2 + 2) TS is even lower than for methyl derivative **10** (ΔΔ*G*^⧧^ = −0.9 kcal/mol *vs* Claisen chair). To our delight, the aryl olefin substrates delivered high yields of the (2 + 2) adduct—in particular, the 4-methoxyphenyl derivative **30a**, which produced a 65% NMR yield of the monomeric *cis* adduct **31a**—and were in close agreement with the computationally predicted product ratio (see Supporting Information, section 5.8). The structure of the *cis* adduct of these aryl derivatives could be unequivocally assessed by XRDS of **31f**, for which enantiomerically pure monocrystals were obtained^58^ (Figure 5C). Additionally, we indeed observed a correlation between olefin electron density and (2 + 2) adduct yield, corroborating our hypothesis that electron-rich olefins promote (2 + 2) activity and simultaneously suppress allyl-lactone production (Figure 5A).

We sought to minimize dimer formation and performed concentration studies using **30d** (Figure 5B). As anticipated, the production of monomeric *cis*-(2 + 2) adduct **31d** was maximized at lower concentrations, while dimer **33d** could be favored at higher concentrations. Production of lactone **32d** was also inhibited at higher concentrations, consistent with an increasingly competitive trapping of the keteniminium ion by the allyl moiety of another activated substrate such as the aryl olefin congeners of **11** or **29**.

In contrast to the methyl olefin derivative **10**, the aryl olefin **30d** formed the dimer compound **33d,** exhibiting both *trans*/*trans*- and *cis*/*trans*-cyclobutanone configurations (**33d** and **33d′**, Figure 5B). These diastereomers were obtained as a 1.5:1 mixture in favor of the *trans*/*trans* derivative and could be isolated pure by preparative high-performance liquid chromatography and fully characterized by NMR spectroscopy as well as XRDS (Figure 5C). Analysis of the IRC of the monomeric *cis*-(2 + 2)-forming TS for analogues of **30** indicates this species proceeds to a stable carbocation, analogous to the entropic intermediate of the methyl olefin substrate **17** and methyl olefin dimer **21** (see Supporting Information, section 5.9). It is reasonable to assume that the benzylic stabilization of this carbocation is the reason for the formation of the two observed diastereomers, as the stability imparted permits the molecule to sample conformers capable of proceeding to both the *trans*/*trans* and *trans*/*cis* configurations.

**Scheme 7:**
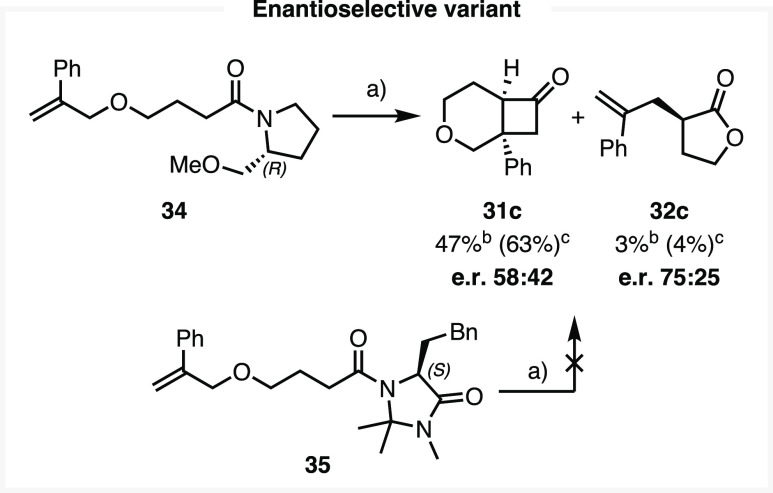
Enantioselective Variant Using Chiral Auxiliaries: α-Methoxymethylpyrrolidine **34** or MacMillan Imidazolidinone **35** (a) 2-Fluoropyridine (2.2 equiv), Tf_2_O (1.1 equiv), 0–20 °C, 4 h, then NaHCO_3(aq)_, 20 °C, 16 h; (b) Isolated yields; (c) NMR yields using mesitylene as an IS.

After reprogramming this process toward (2 + 2) adduct formation, we attempted to formulate an enantioselective variant by installing stereogenic elements on the keteniminium ion. Pleasingly, reactions conducted with α-methoxymethylpyrrolidine substrate **34** demonstrated asymmetric induction with a small but detectable enantiomeric ratio (e.r.) of 58:42 (Scheme 7), a selectivity in-line with previous keteniminium ion processes.^24,25,59^ Our DFT analysis of the four diastereomeric TSs finds that, while one pair of diastereomers—producing a single enantiomer on hydrolysis of the resulting iminium ion—is indeed favored, there is considerable conformational flexibility to the scaffold that renders the energy gap minute (predicted: 71:29 e.r. at 0 °C; see Supporting Information, section 5.10). While minor, this result does provide an interesting proof-of-concept for further experimental and computational development. Other chiral amine scaffolds, such as MacMillan’s imidazolidinones,^60−63^ were found to exhibit larger energy gaps between diastereomeric transition structures as determined by DFT (see Supporting Information, section 5.11). Unfortunately, substrate **35** did not produce observable (2 + 2) or Claisen products, presumably either due to side reactivity between the keteniminium ion and imidazolidinone pendent aryl ring or competition for Tf_2_O between both amide groups.^64^

## Conclusions

We have conducted a thorough computational analysis of Maulide’s 2010 Belluš–Claisen-type rearrangement of keteniminium ion scaffolds, identifying a significant opportunity to alter the observed chemoselectivity of the reaction toward an intramolecular (2 + 2) reaction. Computational predictions of substrates with greater electron density on the olefin moiety indicated that critical cyclization TSs should become highly competitive with the Claisen-type TSs, a prediction that was experimentally confirmed. A remarkable unexpected product forming in these reactions was identified as a dimeric species resulting from twofold (2 + 2) cycloadditions. We provide potential mechanisms—with experimental backing—for the production of monomeric *cis*-(2 + 2) adduct and (2 + 2) dimers, as well as DFT rationalization of a competing deallylation side reaction. We demonstrated experimentally the response of the (2 + 2) cyclization to olefin electronics as well as a proof-of-concept enantioselective (2 + 2) variant. These data demonstrate the mutability of useful intramolecular processes, illustrate the impact of small changes to scaffolds, and highlight the beneficial synergy of computation and experimentation in modern synthetic organic chemistry. Our study elucidates subtle differences between reaction pathways and demonstrates the control over chemoselectivity necessary for future applications of keteniminium ion chemistry to total synthesis and the further advancement of this versatile functional group.

## Supporting Information Available

Computational and experimental methods and procedures, energies, Cartesian coordinates of optimized structures, and experimental characterization data and spectra (PDF)
XYZ files for optimized structures (ZIP)

## Abbreviations

DFT: density functional theory
SMD: solvation model based on density
TS: transition structure
d.r.: diastereomeric ratio
DTBMP: 2,6-(di-*tert*-butyl)-4-methylpyridine
IRC: intrinsic reaction coordinate
IS: internal standard
e.r.: enantiomeric ratio
XRDS: X-ray diffraction spectroscopy
