# Supplementary material for: Taming Keteniminium Reactivity by Steering Reaction Pathways: Computational Predictions and Experimental Validations
Source: J Am Chem Soc. 2022 Dec 16;144(51):23358–67. doi: 10.1021/jacs.2c09146 (PMC9801433; doi:10.1021/jacs.2c09146)
Supplement: Supplementary file 1 — ja2c09146_si_001.pdf [file ja2c09146_si_001.pdf]

Taming keteniminium reactivity by steering reaction pathways: computational predictions and experimental validations

Mark A. Maskeri,[1,‡] Anthony J. Fernandes,[2,‡] Giovanni Di Mauro,[2] Nuno Maulide,[2,*] K. N. Houk[1,*]

[1] Department of Chemistry and Biochemistry, University of California, Los Angeles, California 90095, United States

[2] Institute of Organic Chemistry, University of Vienna, Währinger Straße 38, 1090 Vienna, Austria

KEYWORDS Keteniminium ions, density functional theory, DFT, (2+2) cycloaddition, Belluš-Claisen rearrangement.

1. **General Informations** S3

2. **Experimental Part** S3

2.1. Synthesis of Allyl Bromides Derivatives *S3*

SI.1: 1-Methoxy-4-(prop-1-en-2-yl)benzene (spectrum) S4

SI.2: 1-Bromo-4-(prop-1-en-2-yl)benzene (spectrum) S4

SI.3: 1-Chloro-4-(prop-1-en-2-yl)benzene (spectrum) S4

SI.4: 1-Fluoro-4-(prop-1-en-2-yl)benzene (spectrum) S4

SI.5: 1-Methyl-4-(prop-1-en-2-yl)benzene (spectrum) S5

SI.7: 1-Bromo-4-(3-bromoprop-1-en-2-yl)benzene (spectrum) S5

SI.8: 1-(3-Bromoprop-1-en-2-yl)-4-chlorobenzene (spectrum) S5

SI.9: 1-(3-Bromoprop-1-en-2-yl)-4-fluorobenzene (spectrum) S5

SI.10: (3-Bromoprop-1-en-2-yl)benzene (spectrum) S5

2.2. Synthesis of Alkene-Amide Derivatives *S6*

SI.12: 4-Hydroxy-1-(pyrrolidin-1-yl)butan-1-one (spectrum) S6

10: 4-((2-Methylallyl)oxy)-1-(pyrrolidin-1-yl)butan-1-one (spectrum) S6

30a: 4-((2-(4-Methoxyphenyl)allyl)oxy)-1-(pyrrolidin-1-yl)butan-1-one (spectra) S7

30b: 1-(Pyrrolidin-1-yl)-4-((2-(*p*-tolyl)allyl)oxy)butan-1-one (spectra) S7

30c: 4-((2-Phenylallyl)oxy)-1-(pyrrolidin-1-yl)butan-1-one (spectra) S7

30d: 4-((2-(4-Fluorophenyl)allyl)oxy)-1-(pyrrolidin-1-yl)butan-1-one (spectra) S8

30e: 4-((2-(4-Chlorophenyl)allyl)oxy)-1-(pyrrolidin-1-yl)butan-1-one (spectra) S8

30f: 4-((2-(4-Bromophenyl)allyl)oxy)-1-(pyrrolidin-1-yl)butan-1-one (spectra) S8

2.3. Synthesis of (2+2)-Cycloadduct and Allyl Lactone Derivatives *S9*

13: *Cis*-1-methyl-3-oxabicyclo[4.2.0]octan-7-one (spectra) S9

14: *Trans/trans*-1,9-dimethyl-3,11-dioxatricyclo[12.2.0.0^{6,9}]hexadecane-7,15-dione (spectra) S10

31a: C*is*-1-(4-methoxyphenyl)-3-oxabicyclo[4.2.0]octan-7-one (spectra) S10

31b: *Cis*-1-(p-tolyl)-3-oxabicyclo[4.2.0]octan-7-one (spectra) S10

32b: 3-(2-(*p*-Tolyl)allyl)dihydrofuran-2(3*H*)-one (spectra) S11

31c: *Cis*-1-phenyl-3-oxabicyclo[4.2.0]octan-7-one (spectra) S11

32c: 3-(2-Phenylallyl)dihydrofuran-2(3*H*)-one (spectra) S11

31d: *Cis*-1-(4-fluorophenyl)-3-oxabicyclo[4.2.0]octan-7-one (spectra) S12

32d: 3-(2-(4-Fluorophenyl)allyl)dihydrofuran-2(3*H*)-one (spectra) S12

33d and **33d'**: 1,9-bis(4-fluorophenyl)-2,11-dioxatricyclo[12.2.0.0]hexadecane-7,15-dione S12

31e: *Cis*-1-(4-chlorophenyl)-3-oxabicyclo[4.2.0]octan-7-one (spectra) S13

32e: 3-(2-(4-Chlorophenyl)allyl)dihydrofuran-2(3*H*)-one (spectra) S14
31f: *Cis*-1-(4-bromophenyl)-3-oxabicyclo[4.2.0]octan-7-one (spectra) S14
32f: 3-(2-(4-Bromophenyl)allyl)dihydrofuran-2(3*H*)-one (spectra) S14
2.4. Enantioselective Variant S15
SI.13: 4-((2-Phenylallyl)oxy)butanoic acid (spectra) S15
34: (*R*)-1-(2-(methoxymethyl)pyrrolidin-1-yl)-4-((2-phenylallyl)oxy)butan-1-one (spectra) S15
31c, chiral HPLC: S17
32c, chiral HPLC: S17
2.5. Grignard reagent addition to cyclobutanone derivatives S18
13_{PhCl}: *Cis*-7-(4-chlorophenyl)-1-methyl-3-oxabicyclo[4.2.0]octan-7-ol (spectra) S18
14_{PhCl}: *Trans/trans*-7,15-bis(4-chlorophenyl)-1,9-dimethyl-3,11-dioxatricyclo[12.2.0.06,9]hexadecane-7,15-diol (spectra) S18
2.6. Concentration effect S19
2.7. Collidine adduct S20
16 : (*Z*)-2,4,6-trimethyl-1-(4-((2-methylallyl)oxy)-1-(pyrrolidin-1-yl)but-1-en-1-yl)pyridin-1-ium trifluoromethanesulfonate (spectra) S20
3. NMR spectra S20
4. X-ray crystallography S92
5. Computational part S97
5.1. General Computational Procedure S97
5.2. Complete Authorship of Gaussian 16 .... S97
5.3. Reversible Stabilization of Keteniminium Ion 2 with Collidine S97
5.4. Ynamide Enamine Triflate Model Comparison S98
5.5. Potential Energy Surface: Generation of Allyloxonium Ion 9 from Amide 1 .... S99
5.6. Potential Energy Surface: Methyl Olefin Congener (10) S99
5.7. Forward Intrinsic Reaction Coordinate for TS-6 .... S100
5.8. Aryl Olefin Product Distribution Calculations S100
5.9. Stabilized Cationic Intermediates of Aryl Olefins 30a, 30c (2+2) Reactions S101
5.10. Diastereomeric Transition Structures of Methoxymethylpyrrolidine 34 .... S102
5.11. Representative Diastereomeric TS of Imidazolidinone 35 .... S103
5.12. Computed Structures and Energies S103
6. References S206

1. General Informations

Unless otherwise stated, all glassware was flame-dried before use and all reactions were performed under an atmosphere of argon. All solvents were distilled from appropriate drying agents prior to use or directly taken from commercial sealed bottles under an atmosphere of argon. All reagents were used as received from commercial suppliers unless otherwise stated. Trifluoromethanesulfonic anhydride (Tf_2O) was distilled over P_4O_{10} prior to use and stored under argon atmosphere at 4 °C for a maximum of roughly 3 weeks.[1] Reaction progress was monitored by thin layer chromatography (TLC) performed on aluminum plates coated with silica gel F254 with 0.2 mm thickness. Chromatograms were visualized by fluorescence quenching with UV light at 254 nm or by staining using potassium permanganate, vanillin (for amides) or dinitrophenylhydrazine (for ketones). Flash column chromatography was performed using silica gel 60 (230-400 mesh, Merck and co.). Neat infrared spectra were recorded using a Perkin-Elmer Spectrum 100 FT-IR spectrometer. Wavenumbers (ν_{max}) are reported in cm^{-1}. Mass spectra were obtained using a Finnigan MAT 8200 or (70 eV) or an Agilent 5973 (70 eV) spectrometer, using electrospray ionization (ESI). All ^1H NMR and ^{13}C NMR spectra were recorded using a Bruker AV-400, AV-600 spectrometer or AV-700 spectrometer at 300K. Chemical shifts are given in parts per million (ppm, δ), referenced to the solvent peak of $CDCl_3$, defined at δ = 7.26 ppm (^1H NMR) and δ = 77.16 (^{13}C NMR). Coupling constants are quoted in Hz (*J*). ^1H NMR splitting patterns are designated as singlet (s), doublet (d), triplet (t), quartet (q) and quintet (quint) as they appeared in the spectrum. Splitting patterns that could not be interpreted or easily visualized are designated as multiplet (m) or broad (br). Analytical and preparative HPLC analyses were performed using a Waters-Auto Purification LC/MS System including Waters 2767 Sampler Manager, Waters 2545 Binary Gradient Module, 515 PUMP Waters System Fluidics Organizer SFO, ACQUITY QDa Mass Detector (compact single quad mass detector equipped with an electrospray ionization interface) PC with Waters Masslynx and FractionLynx Software installed. A Waters 2489 UV/Visible Detector dual wavelength detector was used to acquire UV spectra at λ=220 nm and λ=254 nm. X-ray intensity data were measured on Bruker D8 Venture diffractometer equipped with multilayer monochromator, Mo K/α INCOATEC micro focus sealed tube and Oxford cooling system. The structure was solved by *Direct Methods*. Non-hydrogen atoms were refined with *anisotropic displacement parameters*. Hydrogen atoms were inserted at calculated positions and refined with riding model. The following software was used: *Bruker SAINT software package*[2] using a narrow-frame algorithm for frame integration, *SADABS*[3] for absorption correction, *OLEX2*[4] for structure solution, refinement, molecular diagrams and graphical user-interface, *Shelxle*[5] for refinement and graphical user-interface *SHELXS-2015*[6] for structure solution, *SHELXL-2015*[7] for refinement, *Platon*[8] for symmetry check.

2. Experimental Part

2.1. Synthesis of Allyl Bromides Derivatives

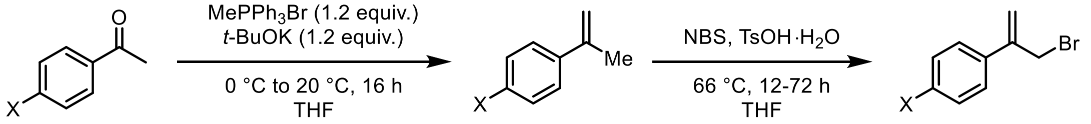

SI.1: X = OMe, 94%
SI.2: X = Br, 88%
SI.3: X = Cl, >99%
SI.4: X = F, 45%
SI.5: X = Me, 97%

SI.6: X = OMe, 28%
SI.7: X = Br, 68%
SI.8: X = Cl, 57%
SI.9: X = F, 44%
SI.10: X = H, 48%
SI.11: X = Me, 49%

Above styrenes SI.1–SI.11 were synthesized following a previously reported procedure.[9] Typical procedure is as follows:

Wittig reaction: A dry flask under argon atmosphere was charged with triphenylphosphonium bromide (1.20 equiv.) and anhydrous THF (0.67 M) and was cooled to 0 °C. *t*-BuOK (1.20 equiv.) was then added and the slurry was stirred at 0 °C for 1 h. A solution of the aryl ketone (1.00 equiv.) in anhydrous THF (2 M) was then added dropwise. The reaction was allowed to reach 20 °C and was stirred at that temperature for 16 h. The resulting slurry was then filtered through a pad of Celite, and the filter cake was thoroughly washed with THF. The filtrate was then concentrated under reduced pressure. Purification by column chromatography (SiO_2, Heptane/EtOAc, 100:0 to 98:2) afforded the desired styrene derivative.

Bromination: A dry flask under argon atmosphere was charged with the styrene derivative (1.00 equiv.) and anhydrous THF (0.67 M). *N*-Bromosuccinimide (1.10 equiv.) was then added and the reaction was heated to 66 °C and subsequently stirred for 12-72 h. After this time, the reaction mixture was allowed to cool to ambient temperature, before water was added and the aqueous layer was extracted three times with EtOAc. The organic

layers were gathered, dried over Na_2SO_4, filtered and the solvent was removed under reduced pressure. Purification by column chromatography (SiO_2, Heptane/EtOAc, 100:0 to 95:5) afforded the desired allyl bromide derivative.

SI.6, and **SI.11** were contaminated with inseparable (yet inconsequential) vinyl bromide, and were used directly in the next step.

SI.1: 1-Methoxy-4-(prop-1-en-2-yl)benzene (spectrum)

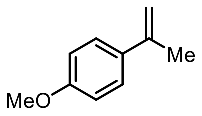

Chemical Formula: $C_{10}H_{12}O$
Exact Mass: 148,0888
Molecular Weight: 148,2050

Following the general procedure for the synthesis of styrene derivatives.[9] Colorless oil.

Yield: 94%.

CAS: 1712-69-2.

^{1}H NMR (400 MHz, $CDCl_3$) δ 7.49 - 7.38 (m, 2H), 6.91 – 6.78 (m, 2H), 5.43 – 5.22 (m, 1H), 5.11 – 4.92 (m, 1H), 3.82 (s, 3H), 2.14 (dd, *J* = 1.3, 0.7 Hz, 3H).

SI.2: 1-Bromo-4-(prop-1-en-2-yl)benzene (spectrum)

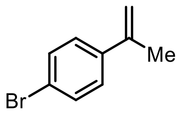

Chemical Formula: C_9H_9Br
Exact Mass: 195,9888
Molecular Weight: 197,0750

Following the general procedure for the synthesis of styrene derivatives.[9]

Colorless oil.

Yield: 88%.

CAS: 6888-79-5.

^{1}H NMR (400 MHz, $CDCl_3$) δ 7.47 – 7.43 (m, 2H), 7.35 – 7.30 (m, 2H), 5.36 (d, *J* = 0.5 Hz, 1H), 5.10 (p, *J* = 1.4 Hz, 1H), 2.13 (dd, *J* = 1.4, 0.8 Hz, 3H).

SI.3: 1-Chloro-4-(prop-1-en-2-yl)benzene (spectrum)

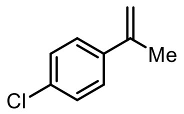

Chemical Formula: C_9H_9Cl
Exact Mass: 152,0393
Molecular Weight: 152,6210

Following the general procedure for the synthesis of styrene derivatives.[9]

Colorless oil.

Yield: >99%.

CAS: 1712-70-5.

^{1}H NMR (400 MHz, $CDCl_3$) δ 7.43 – 7.36 (m, 2H), 7.33 – 7.27 (m, 2H), 5.35 (s, 1H), 5.15 – 5.05 (m, 1H), 2.13 (s, 3H).

SI.4: 1-Fluoro-4-(prop-1-en-2-yl)benzene (spectrum)

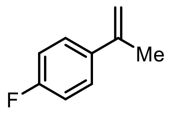

Chemical Formula: C_9H_9F
Exact Mass: 136,0688
Molecular Weight: 136,1694

Following the general procedure for the synthesis of styrene derivatives.[9]

Colorless oil.

Yield: 45%.

CAS: 350-40-3.

^{1}H NMR (200 MHz, $CDCl_3$) δ 7.58 – 7.34 (m, 2H), 7.11 – 6.87 (m, 2H), 5.30 (s, 1H), 5.06 (s, 1H), 2.14 (s, 3H).

SI.5: 1-Methyl-4-(prop-1-en-2-yl)benzene (spectrum)

Following the general procedure for the synthesis of styrene derivatives.[9]

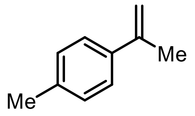

Chemical Formula: $C_{10}H_{12}$
Exact Mass: 132,0939
Molecular Weight: 132,2060

Colorless oil.

Yield: 97%.

CAS: 1195-32-0.

^{1}H NMR (400 MHz, $CDCl_3$) δ 7.38 (d, *J* = 8.2 Hz, 2H), 7.22 – 7.05 (m, 2H), 5.35 (s, 1H), 5.05 (s, 1H), 2.36 (s, 3H), 2.21 – 2.10 (m, 3H).

SI.7: 1-Bromo-4-(3-bromoprop-1-en-2-yl)benzene (spectrum)

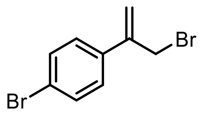

Chemical Formula: $C_9H_8Br_2$
Exact Mass: 273,8993
Molecular Weight: 275,9710

Following the general procedure for the synthesis of styrene derivatives.[9]

Colorless oil.

Yield: 68%.

CAS: 184773-89-5.

^{1}H NMR (200 MHz, $CDCl_3$) δ 7.61 – 7.43 (m, 2H), 7.43 – 7.28 (m, 2H), 5.55 (s, 1H), 5.51 (s, 1H), 4.34 (s, 2H).

SI.8: 1-(3-Bromoprop-1-en-2-yl)-4-chlorobenzene (spectrum)

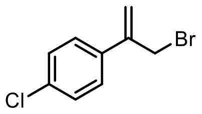

Chemical Formula: C_9H_8BrCl
Exact Mass: 229,9498
Molecular Weight: 231,5170

Following the general procedure for the synthesis of styrene derivatives.[9]

Colorless oil.

Yield: 57%.

CAS: 89220-51-9.

^{1}H NMR (400 MHz, $CDCl_3$) δ 7.48 – 7.38 (m, 2H), 7.39 – 7.30 (m, 2H), 5.54 (s, 1H), 5.50 (s, 1H), 4.35 (s, 2H).

SI.9: 1-(3-Bromoprop-1-en-2-yl)-4-fluorobenzene (spectrum)

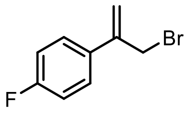

Chemical Formula: C_9H_8BrF
Exact Mass: 213,9793
Molecular Weight: 215,0654

Following the general procedure for the synthesis of styrene derivatives.[9]

Colorless oil.

Yield: 44%.

CAS: 132927-05-0.

^{1}H NMR (200 MHz, $CDCl_3$) δ 7.61 – 7.33 (m, 2H), 7.19 – 6.92 (m, 2H), 5.50 (s, 1H), 5.48 (s, 1H), 4.35 (s, 2H).

SI.10: (3-Bromoprop-1-en-2-yl)benzene (spectrum)

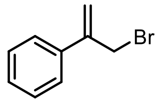

Chemical Formula: C_9H_9Br
Exact Mass: 195,9888
Molecular Weight: 197,0750

Following the general procedure for the synthesis of styrene derivatives.[9]

Colorless oil.

Yield: 48%.

CAS: 3360-54-1.

^{1}H NMR (400 MHz, $CDCl_3$) δ 7.57 – 7.45 (m, 2H), 7.45 – 7.28 (m, 2H), 5.56 (s, 1H), 5.54 – 5.47 (m, 1H), 4.47 – 4.33 (m, 2H).

2.2. Synthesis of Alkene-Amide Derivatives

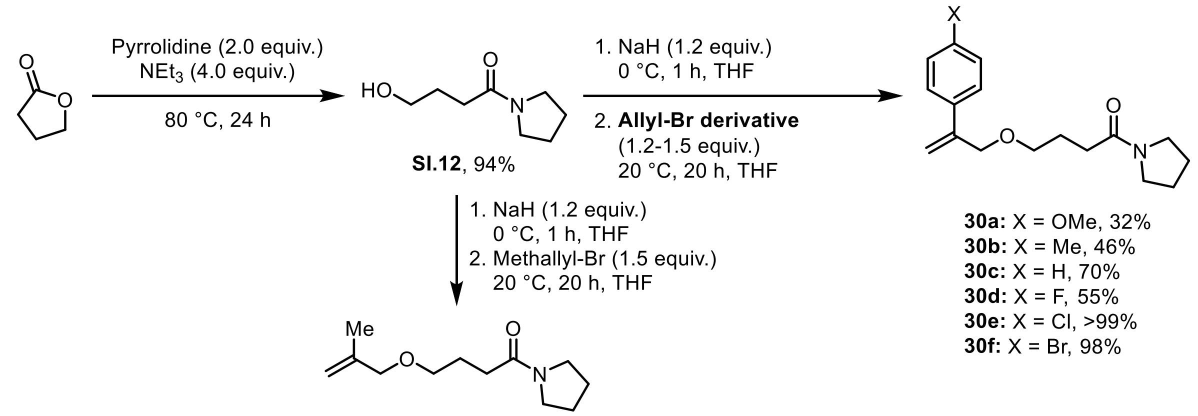

10, >99%

Based on a reported procedure.[10] Typical procedure is as follows:

Hydroxy-amide synthesis:

A flask equipped with a magnetic stirrer and a condenser was charged with γ-butyrolactone (4.61 mL, 60.00 mmol, 1.00 equiv.), pyrrolidine (9.85 mL, 120.00 mmol, 2.00 equiv.) and triethylamine (33.5 mL, 240.00 mmol, 4.00 equiv.) and the solution was heated to 80 °C for 24 h. After subsequent cooling to ambient temperature, the volatiles were removed under reduced pressure. Purification by column chromatography (SiO_2, Heptane/EtOAc, 1:1 to 0:1) afforded the desired product **SI.12** as an orange oil (8.84 g, 56.20 mmol, 94%).

Allylation step:

A dry flask under argon atmosphere was charged with **SI.12** (1.00 equiv.) and anhydrous THF (0.5 M) and was cooled to 0 °C. Sodium hydride (60% suspension in mineral oil, 1.20 equiv.) was added portion-wise, and the reaction mixture was stirred for 1 h at 0 °C. The appropriate **allyl bromide** (1.20-2.30 equiv.) was added and the reaction was then allowed to reach room temperature and was stirred at 20 °C for 20 h. Excess base was quenched by the addition of a NH_4Cl saturated aqueous solution and the aqueous layer was extracted three times with CH_2Cl_2. The organic layers were gathered, dried over Na_2SO_4, filtered and the solvent removed under reduced pressure. Purification by column chromatography (SiO_2, Heptane/EtOAc, 8:2 to 0:1) afforded the desired product.

SI.12: 4-Hydroxy-1-(pyrrolidin-1-yl)butan-1-one (spectrum)

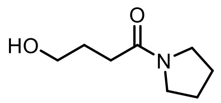

Chemical Formula: $C_8H_{15}NO_2$
Exact Mass: 157,1103
Molecular Weight: 157,2130

Orange oil.

Yield: 94% (8.84 g, 56.20 mmol).

R$_f$: 0.20 (EtOAc).

^{1}H NMR (200 MHz, $CDCl_3$) δ 3.87 – 3.58 (m, 3H), 3.59 – 3.34 (m, 4H), 2.46 (t, J = 6.4 Hz, 2H), 2.11 – 1.61 (m, 6H).

The data match those reported in the literature.[10]

10: 4-((2-Methylallyl)oxy)-1-(pyrrolidin-1-yl)butan-1-one (spectrum)

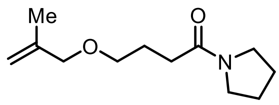

Chemical Formula: $C_{12}H_{21}NO_2$
Exact Mass: 211,1572
Molecular Weight: 211,3050

Obtained from hydroxyl-amide **SI.12** (1.00 g, 6.36 mmol), THF (12.00 mL), NaH 60% (305.0 mg, 7.63 mmol), methallyl bromide (1.00 mL, 9.54 mmol, 1.50 equiv.).

Colorless oil.

Yield: >99% (1.34 g, 6.34 mmol).

R$_f$: 0.30 (EtOAc).

^{1}H NMR (200 MHz, $CDCl_3$) δ 4.93 (s, 1H), 4.86 (s, 1H), 3.85 (s, 2H), 3.62 – 3.29 (m, 6H), 2.37 (t, J = 7.4 Hz, 2H), 2.06 – 1.77 (m, 6H), 1.72 (s, 3H).

The data match those reported in the literature.[10]

30a: 4-((2-(4-Methoxyphenyl)allyl)oxy)-1-(pyrrolidin-1-yl)butan-1-one (spectra)

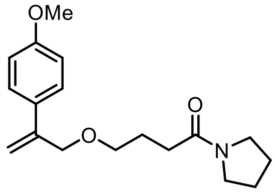

Chemical Formula: $C_{18}H_{25}NO_3$
Exact Mass: 303,1834
Molecular Weight: 303,4020

Obtained from hydroxyl-amide **SI.12** (157.0 mg, 1.00 mmol), THF (2.00 mL), NaH 60% (48.0 mg, 1.20 mmol), **SI.6** (1.19 g, 2.30 mmol).

Colorless oil.

Yield: 32% (123.0 mg, 0.32 mmol).

R$_f$: 0.35 (EtOAc).

^{1}H NMR (700 MHz, $CDCl_3$) δ 7.59 – 7.37 (m, 2H), 6.86 (d, *J* = 8.9 Hz, 2H), 5.42 (d, *J* = 1.1 Hz, 1H), 5.30 – 5.12 (m, 1H), 4.36 – 4.30 (m, 2H), 3.81 (s, 3H), 3.52 (t, *J* = 6.0 Hz, 2H), 3.43 (t, *J* = 6.9 Hz, 2H), 3.29 (t, *J* = 6.8 Hz, 2H), 2.26 (t, *J* = 7.3 Hz, 2H), 1.98 – 1.87 (m, 4H), 1.87 – 1.78 (m, 2H).

^{13}C NMR (176 MHz, $CDCl_3$) δ 171.4, 159.4, 143.9, 131.4, 127.4 (2C), 113.8 (2C), 112.8, 72.9, 69.3, 55.4, 46.6, 45.7, 31.3, 26.2, 25.0, 24.6.

IR (neat) ν_{max} (cm^{-1}): 2971, 2932, 2870, 1639, 1606, 1512, 1436, 1247, 1182, 1117, 1080, 1031, 836.

HRMS (ESI+): *m/z* calculated for $[M+Na]^+$ = 326.1727, *m/z* found = 326.1726.

30b: 1-(Pyrrolidin-1-yl)-4-((2-(*p*-tolyl)allyl)oxy)butan-1-one (spectra)

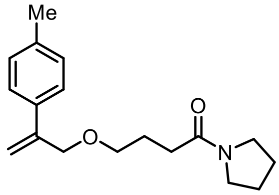

Chemical Formula: $C_{18}H_{25}NO_2$
Exact Mass: 287,1885
Molecular Weight: 287,4030

Obtained from hydroxyl-amide **SI.12** (157.0 mg, 1.00 mmol), THF (2.00 mL), NaH 60% (48.0 mg, 1.20 mmol), **SI.11** (253.0 mg, 1.20 mmol).

Colorless oil.

Yield: 46% (133.0 mg, 0.46 mmol).

R$_f$: 0.30 (Heptane/EtOAc, 3:7).

^{1}H NMR (400 MHz, $CDCl_3$) δ 7.40 – 7.32 (m, 2H), 7.13 (d, *J* = 8.0 Hz, 2H), 5.52 – 5.41 (m, 1H), 5.26 (d, *J* = 1.3 Hz, 1H), 4.36 – 4.31 (m, 2H), 3.53 (t, *J* = 6.0 Hz, 3H), 3.43 (t, *J* = 6.8 Hz, 3H), 3.28 (t, *J* = 6.7 Hz, 3H), 2.34 (s, 3H), 2.26 (t, *J* = 7.3 Hz, 2H), 1.97 – 1.86 (m, 4H), 1.86 – 1.78 (m, 2H).

^{13}C NMR (101 MHz, $CDCl_3$) δ 171.4, 144.4, 137.6, 136.1, 129.1 (2C), 126.1 (2C), 113.5, 72.8, 69.4, 46.6, 45.7, 31.3, 26.2, 25.0, 24.6, 21.3.

IR (neat) ν_{max} (cm^{-1}): 2971, 2950, 2925, 2870, 1640, 1435, 1116, 1079, 825.

HRMS (ESI+): *m/z* calculated for $[M+Na]^+$ = 310.1778, *m/z* found = 310.1777.

30c: 4-((2-Phenylallyl)oxy)-1-(pyrrolidin-1-yl)butan-1-one (spectra)

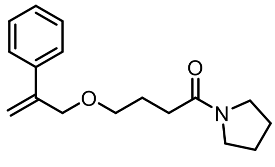

Chemical Formula: $C_{17}H_{23}NO_2$
Exact Mass: 273,1729
Molecular Weight: 273,3760

Obtained from hydroxyl-amide **SI.12** (314.0 mg, 2.00 mmol), THF (4.00 mL), NaH 60% (96.0 mg, 2.40 mmol), **SI.10** (473.0 mg, 1.20 mmol).

Colorless oil.

Yield: 70% (383.0 mg, 1.40 mmol).

R$_f$: 0.25 (Heptane/EtOAc, 3:7).

^{1}H NMR (400 MHz, $CDCl_3$) δ 7.49 – 7.43 (m, 2H), 7.36 – 7.25 (m, 3H), 5.50 (d, *J* = 0.6 Hz, 1H), 5.31 (d, *J* = 1.3 Hz, 1H), 4.36 (d, *J* = 0.6 Hz, 2H), 3.54 (t, *J* = 6.0 Hz, 2H), 3.43 (t, *J* = 6.8 Hz, 2H), 3.28 (t, *J* = 6.7 Hz, 2H), 2.27 (t, *J* = 7.3 Hz, 2H), 1.99 – 1.86 (m, 4H), 1.86 – 1.77 (m, 2H).

^{13}C NMR (101 MHz, $CDCl_3$) δ 171.3, 144.6, 139.0, 128.4 (2C), 127.8, 126.3 (2C), 114.4, 72.8, 69.4, 46.6, 45.7, 31.3, 26.2, 25.0, 24.6.

IR (neat) ν_{max} (cm^{-1}): 2971, 2930, 2870, 1639, 1434, 1083, 1074, 1029, 857, 780, 712.

HRMS (ESI+): *m/z* calculated for $[M+Na]^+$ = 296.1621, *m/z* found = 296.1625.

30d: 4-((2-(4-Fluorophenyl)allyl)oxy)-1-(pyrrolidin-1-yl)butan-1-one (spectra)

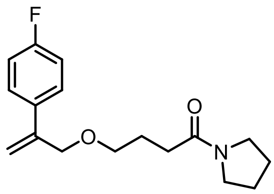

Chemical Formula: $C_{17}H_{22}FNO_2$
Exact Mass: 291,1635
Molecular Weight: 291,3664

Obtained from hydroxyl-amide **SI.12** (104.0 mg, 0.66 mmol), THF (1.30 mL), NaH 60% (32.0 mg, 0.79 mmol), **SI.9** (170.0 mg, 0.79 mmol).

Colorless oil.

Yield: 55% (105.0 mg, 0.36 mmol).

Rf: 0.25 (Heptane/EtOAc, 3:7).

^{1}H NMR (400 MHz, $CDCl_3$) δ 7.49 – 7.40 (m, 2H), 7.01 (t, J = 8.8 Hz, 2H), 5.45 (s, 1H), 5.31 – 5.25 (m, 1H), 4.36 – 4.28 (m, 2H), 3.52 (t, J = 6.0 Hz, 2H), 3.43 (t, J = 6.8 Hz, 2H), 3.29 (t, J = 6.7 Hz, 2H), 2.25 (t, J = 7.3 Hz, 2H), 1.97 – 1.87 (m, 4H), 1.83 (p, J = 6.5 Hz, 2H).

^{13}C NMR (101 MHz, $CDCl_3$) δ 171.3, 162.6 (d, J = 246.8 Hz), 143.7, 135.0 (d, J = 3.3 Hz), 128.0 (d, J = 7.9 Hz, 2C), 115.2 (d, J = 21.3 Hz, 2C), 114.5, 72.9, 69.4, 46.6, 45.7, 31.2, 26.2, 24.9, 24.5.

^{19}F NMR (377 MHz, $CDCl_3$) δ -114.7 – -114.8 (m).

IR (neat) ν_{max} (cm^{-1}): 2972, 2952, 2928, 2875, 1640, 1510, 1437, 1224, 1161, 1118, 840.

HRMS (ESI+): *m/z* calculated for $[M+Na]^+$ = 314.1527, *m/z* found = 314.1517.

30e: 4-((2-(4-Chlorophenyl)allyl)oxy)-1-(pyrrolidin-1-yl)butan-1-one (spectra)

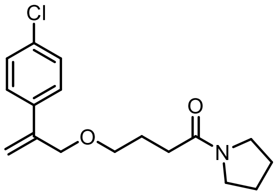

Chemical Formula: $C_{17}H_{22}ClNO_2$
Exact Mass: 307,1339
Molecular Weight: 307,8180

Obtained from hydroxyl-amide **SI.12** (157.0 mg, 1.00 mmol), THF (2.00 mL), NaH 60% (48.0 mg, 1.20 mmol), **SI.8** (278.0 mg, 1.20 mmol).

Colorless oil.

Yield: >99% (307 mg, 1.0 mmol).

Rf: 0.30 (Heptane/EtOAc, 2:8).

^{1}H NMR (400 MHz, $CDCl_3$) δ 7.43 – 7.36 (m, 2H), 7.34 – 7.27 (m, 2H), 5.49 (s, 1H), 5.32 (d, J = 1.2 Hz, 1H), 4.38 – 4.28 (m, 2H), 3.51 (t, J = 6.0 Hz, 2H), 3.43 (t, J = 6.8 Hz, 2H), 3.26 (t, J = 6.7 Hz, 2H), 2.23 (t, J = 7.3 Hz, 2H), 1.91 (p, J = 6.6 Hz, 4H), 1.83 (p, J = 6.6 Hz, 2H).

^{13}C NMR (101 MHz, $CDCl_3$) δ 171.2, 143.7, 137.4, 133.6, 128.6 (2C), 127.7 (2C), 115.1, 72.7, 69.3, 46.6, 45.7, 31.2, 26.2, 24.9, 24.6.

IR (neat) ν_{max} (cm^{-1}): 2972, 2951, 2929, 2871, 1640, 1492, 1437, 1118, 1091, 1012, 835.

HRMS (ESI+): *m/z* calculated for $[M+Na]^+$ = 330.1231, *m/z* found = 330.1233.

30f: 4-((2-(4-Bromophenyl)allyl)oxy)-1-(pyrrolidin-1-yl)butan-1-one (spectra)

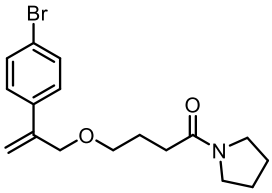

Chemical Formula: $C_{17}H_{22}BrNO_2$
Exact Mass: 351,0834
Molecular Weight: 352,2720

Obtained from hydroxyl-amide **SI.12** (157.0 mg, 1.00 mmol), THF (2.00 mL), NaH 60% (48.0 mg, 1.20 mmol), **SI.7** (331.0 mg, 1.20 mmol).

Colorless oil.

Yield: 98% (346.0 mg, 0.98 mmol).

Rf: 0.30 (Heptane/EtOAc, 2:8).

^{1}H NMR (700 MHz, $CDCl_3$) δ 7.49 – 7.40 (m, 2H), 7.38 – 7.31 (m, 2H), 5.50 (s, 1H), 5.32 (d, J = 1.2 Hz, 1H), 4.45 – 4.19 (m, 2H), 3.51 (t, J = 6.0 Hz, 2H), 3.43 (t, J = 6.9 Hz, 2H), 3.26 (t, J = 6.8 Hz, 2H), 2.22 (t, J = 7.3 Hz, 2H), 1.91 (h, J = 6.2 Hz, 4H), 1.83 (p, J = 6.9 Hz, 2H).

^{13}C NMR (176 MHz, $CDCl_3$) δ 171.2, 143.7, 137.8, 131.5 (2C), 128.0 (2C), 121.8, 115.2, 72.6, 69.3, 46.6, 45.7, 31.2, 26.3, 24.9, 24.6.

IR (neat) ν_{max} (cm^{-1}): 2971, 2949, 2871, 1638, 1489, 1436, 1132, 1087, 1036, 911, 834.

HRMS (ESI+): *m/z* calculated for $[M+Na]^+$ = 374.0726, *m/z* found = 374.0724.

2.3. Synthesis of (2+2)-Cycloadduct and Allyl Lactone Derivatives

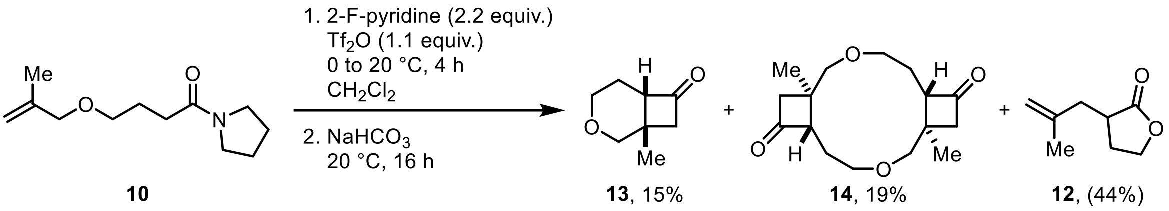

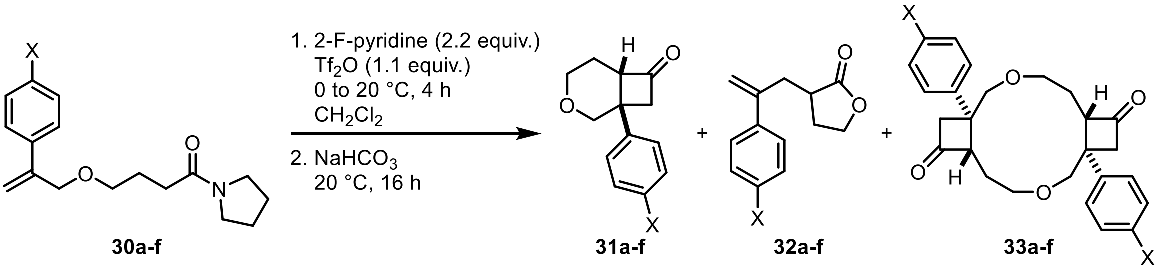

	31a-f	32a-f	33a-f
**a:** X = OMe	47% (65%)	n.d.	(9%)
**b:** X = Me	46% (61%)	5% (6%)	(12%)
**c:** X = H	43% (53%)	16% (18%)	(11%)
**d:** X = F	39% (48%)	11% (12%)	(12%)
**e:** X = Cl	30% (38%)	12% (13%)	(9%)
**f:** X = Br	36% (48%)	22% (22%)	(10%)

NMR yield in parenthesis using mesitylene as internal standard

The general procedure for the (2+2)-cycloaddition is as follow:

A dry flask under argon atmosphere was charged with alkene-amide **10** or **30a-f** (1.00 equiv.), CH_2Cl_2 (0.1 M) and 2-fluoro-pyridine (2.20 equiv.) and was then cooled to 0 °C. After reaching this temperature, triflic anhydride (1.10 equiv.) was added dropwise and the solution was stirred for 10 min at 0 °C. The reaction was then allowed to reach room temperature and was stirred at 20 °C for 4 h. A $NaHCO_3$ saturated aqueous solution was added and the biphasic mixture was vigorously stirred at 20 °C for 16 h. The aqueous layer was extracted then three times with CH_2Cl_2. The organic layers were gathered, dried over Na_2SO_4, filtered and the solvent removed under reduced pressure. Purification by column chromatography (SiO_2, CH_2Cl_2/EtOAc, 100:0 to 99:1) afforded the desired cycloadducts and the lactone.

13: *Cis*-1-methyl-3-oxabicyclo[4.2.0]octan-7-one (spectra)

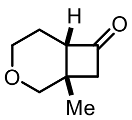

Chemical Formula: $C_8H_{12}O_2$
Exact Mass: 140,0837
Molecular Weight: 140,1820

Obtained from alkene-amide **10** (1.10 g, 5.21 mmol), CH_2Cl_2 (50.00 mL), 2-fluoro-pyridine (0.99 mL, 11.40 mmol) and Tf_2O (0.96 mL, 5.72 mmol).

Yield: 15% (110.0 mg, 0.79 mmol).

Colorless oil.

R$_f$: 0.55 (CH_2Cl_2/EtOAc, 9:1).

^{1}H NMR (400 MHz, $CDCl_3$) δ 3.78 (d, *J* = 12.1 Hz, 1H), 3.76 – 3.71 (m, 1H), 3.51 – 3.43 (m, *J* = 10.6, 5.4 Hz, 1H), 3.44 (d, *J* = 12.1 Hz, 1H), 3.01 (dd, *J* = 7.3, 2.8 Hz, 1H), 2.81 (s, 2H), 1.95 – 1.86 (m, 1H), 1.83 – 1.72 (m, 1H), 1.41 (s, 3H).

^{13}C NMR (101 MHz, $CDCl_3$) δ 207.8, 75.0, 64.9, 59.8, 55.3, 28.6, 24.1, 20.3.

IR (neat) ν_{max} (cm^{-1}): 2956, 2919, 2868, 2844, 1772, 1463, 1173, 1105, 1091, 1059, 849.

HRMS (ESI+): *m/z* calculated for $[M+Na]^+$ = 163.0730, *m/z* found = 163.0733.

14: *Trans/trans*-1,9-dimethyl-3,11-dioxatricyclo[12.2.0.0^{6,9}]hexadecane-7,15-dione (spectra)

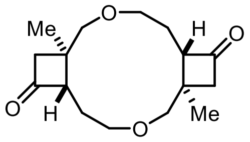

Chemical Formula: $C_{16}H_{24}O_4$
Exact Mass: 280,1675
Molecular Weight: 280,3640

Obtained from alkene-amide **10** (1.10 g, 5.21 mmol), CH_2Cl_2 (50.00 mL), 2-fluoro-pyridine (0.99 mL, 11.40 mmol) and Tf_2O (0.96 mL, 5.72 mmol).

Yield: 19% (141.0 mg, 1.01 mmol).

Colorless oil.

R$_f$: 0.45 (CH_2Cl_2/EtOAc, 9:1).

^{1}H NMR (400 MHz, $CDCl_3$) δ 3.97 – 3.89 (m, 2H), 3.67 (ddd, *J* = 10.0, 5.6, 4.4 Hz, 2H), 3.55 (d, *J* = 9.1 Hz, 2H), 3.37 (td, *J* = 9.6, 3.5 Hz, 2H), 3.27 (d, *J* = 9.1 Hz, 2H), 3.14 (dd, *J* = 15.9, 2.5 Hz, 2H), 2.30 (dd, *J* = 15.8, 0.9 Hz, 2H), 2.00 (ddd, *J* = 19.2, 9.1, 4.4 Hz, 2H), 1.62 (dddd, *J* = 14.9, 9.2, 5.7, 3.6 Hz, 2H), 1.11 (s, 6H).

^{13}C NMR (101 MHz, $CDCl_3$) δ 209.0 (2C), 75.8 (2C), 68.2 (2C), 58.5 (2C), 53.0 (2C), 33.9 (2C), 23.4 (2C), 18.7 (2C).

IR (neat) ν$_{max}$ (cm^{-1}): 2955, 2922, 2855, 2793, 1765, 1433, 1119, 1086, 1060, 891.

HRMS (ESI+): *m/z* calculated for $[M+Na]^+$ = 303.1567, *m/z* found = 303.1569.

31a: C*is*-1-(4-methoxyphenyl)-3-oxabicyclo[4.2.0]octan-7-one (spectra)

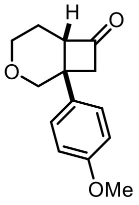

Chemical Formula: $C_{14}H_{16}O_3$
Exact Mass: 232,1099
Molecular Weight: 232,2790

Obtained from alkene-amide **30a** (61.0 mg, 0.20 mmol), CH_2Cl_2 (2.00 mL), 2-fluoro-pyridine (38.0 µL, 0.44 mmol) and Tf_2O (37.0 µL, 0.22 mmol). The lactone by-product was not detected.

Yield: 47% (22.0 mg, 0.090 mmol), 65% NMR using mesitylene as internal standard.

Colorless oil.

R$_f$: 0.15 (CH_2Cl_2).

^{1}H NMR (600 MHz, $CDCl_3$) δ 7.40 – 7.20 (m, 2H), 7.03 – 6.81 (m, 2H), 3.99 (d, *J* = 12.1 Hz, 1H), 3.94 (ddd, *J* = 11.3, 7.5, 1.9 Hz, 1H), 3.82 (s, 3H), 3.65 (d, *J* = 12.1 Hz, 1H), 3.62 (dt, *J* = 11.3, 5.6 Hz, 1H), 3.59 (dd, *J* = 5.1, 2.5 Hz, 1H), 3.34 (dd, *J* = 16.3, 2.2 Hz, 1H), 3.16 (dd, *J* = 16.3, 2.0 Hz, 1H), 2.16 (ddt, *J* = 14.6, 5.9, 2.1 Hz, 1H), 2.10 – 1.99 (m, 1H).

^{13}C NMR (151 MHz, $CDCl_3$) δ 206.8, 158.6, 137.3, 127.3 (2C), 114.2 (2C), 76.2, 64.7, 59.9, 55.5, 55.3, 36.2, 21.1.

IR (neat) ν$_{max}$ (cm^{-1}): 2966, 2937, 2914, 2837, 1776, 1513, 1247, 1112, 1097, 1050, 1032, 830.

HRMS (ESI+): *m/z* calculated for $[M+Na]^+$ = 255.0992, *m/z* found = 255.0994.

31b: *Cis*-1-(p-tolyl)-3-oxabicyclo[4.2.0]octan-7-one (spectra)

Obtained from alkene-amide **30b** (58.0 mg, 0.20 mmol), CH_2Cl_2 (2.00 mL), 2-fluoro-pyridine (38.0 µL, 0.44 mmol) and Tf_2O (37.0 µL, 0.22 mmol).

Yield: 46% (20.0 mg, 0.090 mmol), 61% NMR using mesitylene as internal standard.

Colorless oil.

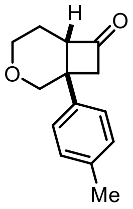

Chemical Formula: $C_{14}H_{16}O_2$
Exact Mass: 216,1150
Molecular Weight: 216,2800

R$_f$: 0.20 (CH_2Cl_2).

^{1}H NMR (400 MHz, $CDCl_3$) δ 7.21 (q, *J* = 8.2 Hz, 4H), 4.00 (d, *J* = 12.1 Hz, 1H), 3.94 (ddd, *J* = 11.2, 7.5, 2.0 Hz, 1H), 3.66 (d, *J* = 12.1 Hz, 1H), 3.66 – 3.57 (m, 2H), 3.36 (dd, *J* = 16.4, 2.2 Hz, 1H), 3.17 (dd, *J* = 16.4, 1.9 Hz, 1H), 2.36 (s, 3H), 2.17 (ddt, *J* = 14.5, 5.9, 2.1 Hz, 1H), 2.06 (ddt, *J* = 14.5, 11.1, 7.3 Hz, 1H).

^{13}C NMR (101 MHz, $CDCl_3$) δ 206.8, 142.2, 136.7, 129.5 (2C), 126.1 (2C), 76.1, 64.7, 59.8, 55.2, 36.5, 21.1, 21.1.

IR (neat) ν$_{max}$ (cm^{-1}): 2968, 2919, 2848, 1778, 1515, 1114, 1098, 1064, 1049, 816, 538.

HRMS (ESI+): *m/z* calculated for $[M+Na]^+$ = 239.1043, *m/z* found = 239.1041.

32b: 3-(2-(*p*-Tolyl)allyl)dihydrofuran-2(3*H*)-one (spectra)

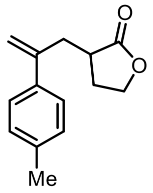

Chemical Formula: $C_{14}H_{16}O_2$
Exact Mass: 216,1150
Molecular Weight: 216,2800

Obtained as a by-product starting from alkene-amide **30b**.

Yield: 5% (2.0 mg, 0.010 mmol), 6% NMR using mesitylene as internal standard.

Colorless oil.

R$_f$: 0.45 (CH_2Cl_2).

^{1}H NMR (400 MHz, $CDCl_3$) δ 7.31 (d, *J* = 8.1 Hz, 2H), 7.16 (d, *J* = 8.0 Hz, 2H), 5.31 (s, 1H), 5.09 (s, 1H), 4.31 (td, *J* = 8.8, 2.6 Hz, 1H), 4.10 (td, *J* = 9.5, 6.7 Hz, 1H), 3.33 (dd, *J* = 14.5, 2.7 Hz, 1H), 2.70 – 2.58 (m, 1H), 2.40 (dd, *J* = 14.7, 11.5 Hz, 1H), 2.35 (s, 3H), 2.18 (dddd, *J* = 12.8, 9.0, 6.7, 2.7 Hz, 1H), 1.98 – 1.85 (m, 1H).

^{13}C NMR (101 MHz, $CDCl_3$) δ 179.3, 145.4, 138.0, 136.8, 129.4 (2C), 126.3 (2C), 114.0, 66.7, 38.3, 36.8, 28.6, 21.2.

IR (neat) ν_{max} (cm^{-1}): 2986, 2913, 1767, 1681, 1563, 1375, 1205, 1152, 1024, 825, 735.

HRMS (ESI+): *m/z* calculated for $[M+Na]^+$ = 239.1043, *m/z* found = 239.1042.

31c: *Cis*-1-phenyl-3-oxabicyclo[4.2.0]octan-7-one (spectra)

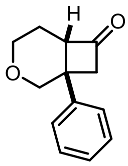

Chemical Formula: $C_{13}H_{14}O_2$
Exact Mass: 202,0994
Molecular Weight: 202,2530

Obtained from alkene-amide **30c** (55.0 mg, 0.20 mmol), CH_2Cl_2 (2.00 mL), 2-fluoropyridine (38.0 µL, 0.44 mmol) and Tf_2O (37.0 µL, 0.22 mmol).

Yield: 43% (18.0 mg, 0.090 mmol), 53% NMR using mesitylene as internal standard.

Colorless oil.

R$_f$: 0.15 (CH_2Cl_2).

^{1}H NMR (700 MHz, $CDCl_3$) δ 7.41 – 7.36 (m, 2H), 7.35 – 7.32 (m, 2H), 7.31 – 7.28 (m, 1H), 4.02 (d, *J* = 12.2 Hz, 1H), 3.95 (ddd, *J* = 11.3, 7.6, 1.8 Hz, 1H), 3.67 (d, *J* = 12.2 Hz, 1H), 3.63 (dq, *J* = 11.4, 5.9 Hz, 2H), 3.38 (dd, *J* = 16.3, 2.2 Hz, 1H), 3.19 (dd, *J* = 16.3, 2.0 Hz, 1H), 2.18 (ddt, *J* = 14.7, 5.7, 2.0 Hz, 1H), 2.12 – 2.01 (m, 1H).

^{13}C NMR (176 MHz, $CDCl_3$) δ 206.7, 145.2, 128.9 (2C), 127.0, 126.2 (2C), 76.1, 64.7, 59.7, 55.2, 36.8, 21.1.

IR (neat) ν_{max} (cm^{-1}): 2967, 2941, 2916, 2848, 1776, 1496, 1446, 1099, 1050, 766, 701.

HRMS (ESI+): *m/z* calculated for $[M+Na]^+$ = 225.0886, *m/z* found = 225.0880.

32c: 3-(2-Phenylallyl)dihydrofuran-2(3*H*)-one (spectra)

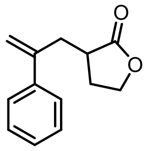

Chemical Formula: $C_{13}H_{14}O_2$
Exact Mass: 202,0994
Molecular Weight: 202,2530

Obtained as a by-product starting from alkene-amide **30c**.

Yield: 16% (6.0 mg, 0.030 mmol), 18% NMR using mesitylene as internal standard.

Colorless oil.

R$_f$: 0.45 (CH_2Cl_2).

^{1}H NMR (400 MHz, $CDCl_3$) δ 7.46 – 7.27 (m, 5H), 5.35 (t, *J* = 1.0 Hz, 1H), 5.14 (q, *J* = 1.3 Hz, 1H), 4.31 (td, *J* = 8.8, 2.7 Hz, 1H), 4.10 (ddd, *J* = 9.9, 9.1, 6.7 Hz, 1H), 3.39 – 3.28 (m, 1H), 2.63 (tdd, *J* = 10.6, 8.6, 3.8 Hz, 1H), 2.43 (ddd, *J* = 14.5, 11.1, 0.8 Hz, 1H), 2.19 (dddd, *J* = 12.8, 8.7, 6.7, 2.7 Hz, 1H), 1.92 (dtd, *J* = 12.8, 10.1, 8.5 Hz, 1H).

^{13}C NMR (101 MHz, $CDCl_3$) δ 179.2, 145.7, 139.8, 128.7 (2C), 128.1, 126.4 (2C), 114.8, 66.7, 38.3, 36.8, 28.6.

IR (neat) ν_{max} (cm^{-1}): 2986, 2910, 2908, 1762, 1374, 1206, 1150, 1023, 913, 780, 701.

HRMS (ESI+): *m/z* calculated for $[M+Na]^+$ = 225.0886, *m/z* found = 225.0880.

31d: *Cis*-1-(4-fluorophenyl)-3-oxabicyclo[4.2.0]octan-7-one (spectra)

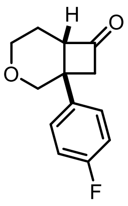

Chemical Formula: $C_{13}H_{13}FO_2$
Exact Mass: 220,0900
Molecular Weight: 220,2434

Obtained from alkene-amide **30d** (58.0 mg, 0.20 mmol), CH_2Cl_2 (2.00 mL), 2-fluoro-pyridine (38.0 µL, 0.44 mmol) and Tf_2O (37.0 µL, 0.22 mmol). Single crystals suitable for X-ray diffraction spectroscopy were obtained from slow evaporation of a saturated CH_2Cl_2 solution (See section 4).

Yield: 39% (17.0 mg, 0.080 mmol), 48% NMR using mesitylene as internal standard.

Colorless oil.

R_f: 0.20 (CH_2Cl_2).

^{1}H NMR (700 MHz, $CDCl_3$) δ 7.34 – 7.28 (m, 2H), 7.11 – 7.03 (m, 2H), 4.01 (d, *J* = 12.1 Hz, 1H), 3.95 (ddd, *J* = 11.4, 7.5, 1.8 Hz, 1H), 3.63 (d, *J* = 12.2 Hz, 1H), 3.62 – 3.56 (m, 2H), 3.34 (dd, *J* = 16.3, 2.1 Hz, 1H), 3.15 (dd, *J* = 16.3, 1.9 Hz, 1H), 2.17 (ddt, *J* = 14.7, 5.6, 1.9 Hz, 1H), 2.09 – 1.99 (m, 1H).

^{13}C NMR (176 MHz, $CDCl_3$) δ 206.0, 161.9 (d, *J* = 245.8 Hz), 141.0 (d, *J* = 3.2 Hz), 127.8 (d, *J* = 7.9 Hz, 2C), 115.7 (d, *J* = 21.3 Hz, 2C), 76.2, 64.8, 59.9, 55.2, 36.3, 20.9.

^{19}F NMR (659 MHz, $CDCl_3$) δ -115.7.

IR (neat) ν_{max} (cm^{-1}): 2941, 2917, 2849, 1775, 1510, 1220, 1160, 1128, 1100, 1049, 833, 807, 541.

HRMS (ESI+): *m/z* calculated for $[M+Na]^+$ = 243.0792, *m/z* found = 243.0790.

32d: 3-(2-(4-Fluorophenyl)allyl)dihydrofuran-2(3*H*)-one (spectra)

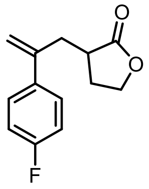

Chemical Formula: $C_{13}H_{13}FO_2$
Exact Mass: 220,0900
Molecular Weight: 220,2434

Obtained as a by-product starting from alkene-amide **30d**.

Yield: 11% (5.0 mg, 0.020 mmol), 12% NMR using mesitylene as internal standard.

Colorless oil.

R_f: 0.45 (CH_2Cl_2).

^{1}H NMR (700 MHz, $CDCl_3$) δ 7.41 – 7.35 (m, 2H), 7.06 – 7.01 (m, 2H), 5.30 (s, 1H), 5.13 (s, 1H), 4.32 (td, *J* = 8.8, 2.7 Hz, 1H), 4.17 – 4.09 (m, 1H), 3.37 – 3.25 (m, 1H), 2.60 (tdd, *J* = 10.7, 8.7, 3.9 Hz, 1H), 2.42 (dd, *J* = 14.7, 11.0 Hz, 1H), 2.20 (dddd, *J* = 12.8, 9.0, 6.7, 2.7 Hz, 1H), 1.92 (dtd, *J* = 12.8, 10.1, 8.6 Hz, 1H).

^{13}C NMR (176 MHz, $CDCl_3$) δ 179.1, 162.7 (d, *J* = 247.3 Hz), 144.6, 135.8 (d, *J* = 3.3 Hz), 128.0 (d, *J* = 8.0 Hz, 2C), 115.6 (d, *J* = 21.4 Hz, 2C), 114.8, 66.7, 38.2, 36.9, 28.6.

^{19}F NMR (659 MHz, $CDCl_3$) δ -114.2.

IR (neat) ν_{max} (cm^{-1}): 2987, 2918, 2876, 1762, 1508, 1374, 1224, 1205, 1160, 1023, 840.

HRMS (ESI+): *m/z* calculated for $[M+Na]^+$ = 243.0792, *m/z* found = 243.0794.

33d and **33d'**: 1,9-bis(4-fluorophenyl)-2,11-dioxatricyclo[12.2.0.0]hexadecane-7,15-dione

Obtained starting from alkene-amide **30d** (1.00 mmol scale), while using a concentrated solution in DCM (0.5M). Yield 19% (83.0 mg, 0.19 mmol), d.r. = 1.5:1 (*trans/trans* : *trans/cis*). 21% NMR using mesitylene as internal standard. The two diastereomers where separated by preparative HPLC [Column, XBridge C18 5µm 30x150mm, (H_2O + 0.1% HCOOH)/ACN, 40:60 to 20:80 for 15 min, then 1 min up to 5:95, and 4 min 5:95] and fully characterized. Single crystal suitable for X-ray diffraction spectroscopy were obtained from slow evaporation of a concentrated CH_2Cl_2 solution (see section 4).

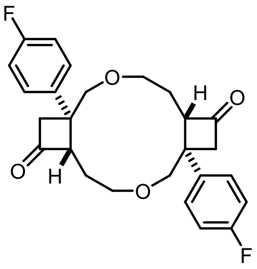

Chemical Formula: $C_{26}H_{26}F_2O_4$
Exact Mass: 440,1799
Molecular Weight: 440,4868

33d: *Trans/trans*-1,9-bis(4-fluorophenyl)-2,11-dioxatricyclo[12.2.0.0^{6,9}]hexadecane-7,15-dione (spectra)

Yield: 5% (23.0 mg, 0.052 mmol).

White solid.

R$_f$: 0.45 (CH_2Cl_2).

^{1}H NMR (600 MHz, $CDCl_3$) δ 7.29 – 7.24 (m, 2H), 7.07 – 7.01 (m, 2H), 4.25 (dtt, *J* = 5.4, 3.6, 1.7 Hz, 1H), 4.01 (d, *J* = 9.0 Hz, 1H), 3.63 (d, *J* = 8.9 Hz, 1H), 3.56 (ddd, *J* = 10.4, 8.9, 3.7 Hz, 1H), 3.50 – 3.41 (m, 1H), 2.96 (dd, *J* = 16.9, 1.2 Hz, 1H), 1.88 (dtd, *J* = 15.1, 5.7, 3.7 Hz, 1H), 1.37 (dddd, *J* = 15.3, 8.8, 6.5, 4.0 Hz, 1H).

^{13}C NMR (151 MHz, $CDCl_3$) δ 208.4 (2C), 161.6 (d, $^1J_{C,F}$ = 247.1 Hz, 2C), 136.9 (2C), 128.3 (d, $^1J_{C,F}$ = 7.7 Hz, 4C), 115.4 (d, $^1J_{C,F}$ = 20.9 Hz, 4C), 74.0 (2C), 68.5 (2C), 62.3 (2C), 54.1 (2C), 40.6 (2C), 24.4 (2C).

^{19}F NMR (565 MHz, $CDCl_3$) δ -115.5 – -115.5 (m).

IR (neat) ν$_{max}$ (cm^{-1}): 3052, 2921, 2861, 1768, 1602, 1512, 1235, 1120, 835, 735.

HRMS (ESI+): *m/z* calculated for $[M+Na]^+$ = 463.1691, *m/z* found = 463.1683.

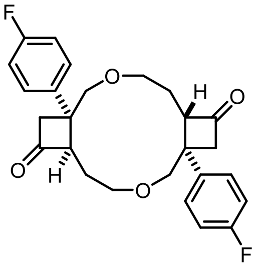

Chemical Formula: $C_{26}H_{26}F_2O_4$
Exact Mass: 440,1799
Molecular Weight: 440,4868

33d': *Trans/cis*-1,9-bis(4-fluorophenyl)-2,11-dioxatricyclo[12.2.0.0^{6,9}]hexadecane-7,15-dione (spectra)

Single crystals suitable for X-ray diffraction spectroscopy were obtained from slow evaporation of a saturated CH_2Cl_2 solution (See section 4).

Yield: 2% (8.0 mg, 0.017 mmol).

White solid.

R$_f$: 0.42 (CH_2Cl_2).

^{1}H NMR (700 MHz, $CDCl_3$) δ 7.31 (dd, *J* = 8.9, 5.2 Hz, 2H), 7.15 – 7.12 (m, 2H), 7.02 (t, *J* = 8.6 Hz, 2H), 6.99 (t, *J* = 8.7 Hz, 2H), 4.20 (d, *J* = 9.0 Hz, 1H), 4.19 – 4.15 (m, 1H), 3.96 – 3.92 (m, 1H), 3.89 – 3.86 (m, 1H), 3.83 (d, *J* = 9.1 Hz, 1H), 3.56 (d, *J* = 9.8 Hz, 1H), 3.53 – 3.49 (m, 2H), 3.35 – 3.27 (m, 2H), 3.10 – 2.98 (m, 4H), 2.24 – 2.17 (m, 1H), 2.10 – 2.03 (m, 1H), 1.95 – 1.87 (m, 1H), 1.12 – 1.04 (m, 1H).

^{13}C NMR (176 MHz, $CDCl_3$) δ 209.5, 204.5, 161.8 (d, $^1J_{C,F}$ = 245.8 Hz), 161.5 (d, $^1J_{C,F}$ = 245.7 Hz), 141.1 (d, $^4J_{C,F}$ = 3.2 Hz), 138.0 (d, $^4J_{C,F}$ = 3.0 Hz), 128.7 (d, $^3J_{C,F}$ = 7.7 Hz, 2C), 128.0 (d, $^3J_{C,F}$ = 8.0 Hz, 2C), 115.6 (d, $^2J_{C,F}$ = 21.4 Hz, 2C), 115.2 (d, $^2J_{C,F}$ = 21.0 Hz, 2C), 77.0, 73.2, 67.1, 66.2, 63.7, 61.4, 55.9, 52.3, 43.1, 40.5, 26.2, 25.5.

^{19}F NMR (659 MHz, $CDCl_3$) δ -115.6, -116.3.

IR (neat) ν$_{max}$ (cm^{-1}): 2922, 2856, 1775, 1601, 1512, 1235, 1100, 835.

HRMS (ESI+): *m/z* calculated for $[M+Na]^+$ = 463.1691, *m/z* found = 463.1688.

31e: *Cis*-1-(4-chlorophenyl)-3-oxabicyclo[4.2.0]octan-7-one (spectra)

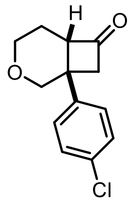

Chemical Formula: $C_{13}H_{13}ClO_2$
Exact Mass: 236,0604
Molecular Weight: 236,6950

Obtained from alkene-amide **30f** (62.0 mg, 0.20 mmol), CH_2Cl_2 (2.00 mL), 2-fluoro-pyridine (38.0 µL, 0.44 mmol) and Tf_2O (37.0 µL, 0.22 mmol).

Yield: 30% (14.0 mg, 0.060 mmol), 38% NMR using mesitylene as internal standard.

Colorless oil.

R$_f$: 0.15 (CH_2Cl_2).

^{1}H NMR (400 MHz, $CDCl_3$) δ 7.38 – 7.32 (m, 2H), 7.31 – 7.24 (m, 2H), 4.01 (d, *J* = 12.2 Hz, 1H), 3.96 (ddd, *J* = 11.3, 7.4, 1.7 Hz, 1H), 3.63 (d, *J* = 12.2 Hz, 1H), 3.59 (td, *J* = 11.3, 5.7 Hz, 2H), 3.34 (dd, *J* = 16.3, 2.1 Hz, 1H), 3.15 (dd, *J* = 16.3, 1.9 Hz, 1H), 2.16 (ddt, *J* = 14.7, 5.6, 1.9 Hz, 1H), 2.03 (ddt, *J* = 14.6, 11.4, 7.1 Hz, 1H).

^{13}C NMR (101 MHz, $CDCl_3$) δ 205.8, 143.7, 133.0, 129.0 (2C), 127.6 (2C), 76.0, 64.8, 59.9, 55.1, 36.4, 20.9.

IR (neat) ν_{max} (cm^{-1}): 2944, 2916, 2849, 1778, 1494, 1108, 1094, 1049, 1013, 826.

HRMS (ESI+): *m/z* calculated for $[M+Na]^+$ = 259.0496, *m/z* found = 259.0494.

32e: 3-(2-(4-Chlorophenyl)allyl)dihydrofuran-2(3*H*)-one (spectra)

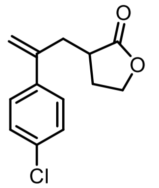

Chemical Formula: $C_{13}H_{13}ClO_2$
Exact Mass: 236,0604
Molecular Weight: 236,6950

Obtained as a by-product starting from alkene-amide **30e**.

Yield: 12% (6.0 mg, 0.030 mmol), 13% NMR using mesitylene as internal standard.

Colorless oil.

R_f: 0.45 (CH_2Cl_2).

^{1}H NMR (400 MHz, $CDCl_3$) δ 7.38 – 7.30 (m, 4H), 5.34 (s, 1H), 5.16 (s, 1H), 4.32 (td, *J* = 8.8, 2.6 Hz, 1H), 4.12 (td, *J* = 9.5, 6.7 Hz, 1H), 3.35 – 3.24 (m, 1H), 2.60 (tdd, *J* = 10.7, 8.7, 3.8 Hz, 1H), 2.42 (dd, *J* = 14.6, 11.0 Hz, 1H), 2.19 (dddd, *J* = 12.8, 9.0, 6.7, 2.6 Hz, 1H), 1.99 – 1.84 (m, 1H).

^{13}C NMR (101 MHz, $CDCl_3$) δ 179.0, 144.6, 138.2, 134.0, 128.9 (2C), 127.7 (2C), 115.3, 66.6, 38.2, 36.7, 28.6.

IR (neat) ν_{max} (cm^{-1}): 2987, 2912, 1761, 1491, 1373, 1150, 1091, 1024, 1010, 913, 836.

HRMS (ESI+): *m/z* calculated for $[M+Na]^+$ = 259.0496, *m/z* found = 259.0496.

31f: *Cis*-1-(4-bromophenyl)-3-oxabicyclo[4.2.0]octan-7-one (spectra)

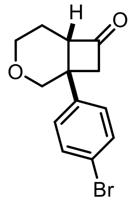

Chemical Formula: $C_{13}H_{13}BrO_2$
Exact Mass: 280,0099
Molecular Weight: 281,1490

Obtained from alkene-amide **30a** (71.0 mg, 0.20 mmol), CH_2Cl_2 (2.00 mL), 2-fluoro-pyridine (38.0 µL, 0.44 mmol) and Tf_2O (37.0 µL, 0.22 mmol).

Yield: 36% (20.0 mg, 0.070 mmol), 48% NMR using mesitylene as internal standard.

Colorless oil.

R_f: 0.15 (CH_2Cl_2).

^{1}H NMR (400 MHz, $CDCl_3$) δ 7.53 – 7.47 (m, 2H), 7.25 – 7.18 (m, 2H), 4.01 (dd, *J* = 12.2, 1.1 Hz, 1H), 3.95 (ddd, *J* = 11.4, 7.4, 1.9 Hz, 1H), 3.63 (d, *J* = 12.2 Hz, 1H), 3.63 – 3.54 (m, 2H), 3.34 (dd, *J* = 16.3, 2.1 Hz, 1H), 3.14 (dd, *J* = 16.3, 1.9 Hz, 1H), 2.16 (ddt, *J* = 14.8, 5.7, 2.1 Hz, 1H), 2.02 (ddt, *J* = 14.5, 11.3, 7.1 Hz, 1H).

^{13}C NMR (101 MHz, $CDCl_3$) δ 205.7, 144.2, 131.9 (2C), 128.0 (2C), 121.0, 76.0, 64.8, 59.9, 55.0, 36.4, 20.9.

IR (neat) ν_{max} (cm^{-1}): 2941, 2916, 2849, 1772, 1491, 1394, 1106, 1095, 1047, 1008, 819, 734, 537.

HRMS (ESI+): *m/z* calculated for $[M+Na]^+$ = 302.9991, *m/z* found = 302.9990.

32f: 3-(2-(4-Bromophenyl)allyl)dihydrofuran-2(3*H*)-one (spectra)

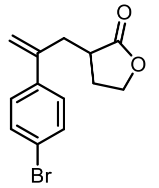

Chemical Formula: $C_{13}H_{13}BrO_2$
Exact Mass: 280,0099
Molecular Weight: 281,1490

Obtained as a by-product starting from alkene-amide **30f**.

Yield: 22% (13.0 mg, 0.040 mmol), 22% NMR using mesitylene as internal standard.

Colorless oil.

R_f: 0.45 (CH_2Cl_2).

^{1}H NMR (400 MHz, $CDCl_3$) δ 7.52 – 7.44 (m, 2H), 7.31 – 7.26 (m, 2H), 5.34 (t, *J* = 0.9 Hz, 1H), 5.16 (dt, *J* = 1.9, 1.0 Hz, 1H), 4.32 (td, *J* = 8.8, 2.7 Hz, 1H), 4.12 (ddd, *J* = 9.9, 9.1, 6.7 Hz, 1H), 3.28 (ddd, *J* = 14.6, 4.0, 1.5 Hz, 1H), 2.60 (tdd, *J* = 10.6, 8.6, 3.8 Hz, 1H), 2.42 (ddd, *J* = 14.6, 11.0, 0.8 Hz, 1H), 2.19 (dddd, *J* = 12.8, 9.0, 6.7, 2.7 Hz, 1H), 1.91 (dtd, *J* = 12.8, 10.1, 8.5 Hz, 1H).

^{13}C NMR (101 MHz, $CDCl_3$) δ 178.9, 144.6, 138.7, 131.9 (2C), 128.0 (2C), 122.1, 115.4, 66.6, 38.2, 36.6, 28.6.

IR (neat) ν_{max} (cm^{-1}): 3087, 2987, 2908, 1761, 1488, 1373, 1150, 1023, 1006, 912, 833, 734.

HRMS (ESI+): *m/z* calculated for $[M+Na]^+$ = 302.9991, *m/z* found = 302.9986.

2.4. Enantioselective Variant

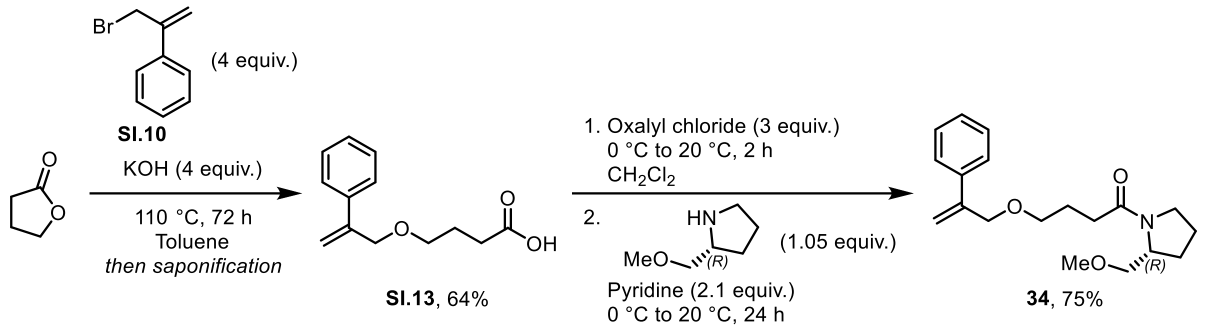

SI.13: 4-((2-Phenylallyl)oxy)butanoic acid (spectra)

Based on a related procedure:[11] To γ-butyrolactone (0.38 mL, 5.00 mmol, 1.00 equiv.) in toluene (5.00 mL, 1.0 M) were added KOH (1.12 g, 20.00 mmol, 4.00 equiv.) and **SI.10** (3.94 g, 20.00 mmol, 4.00 equiv.). The mixture was stirred and heated at reflux (110 °C) for 72 h, and then allowed to cool to room temperature, before being diluted with H_2O and extracted three times with Et_2O. The organic layers were gathered, dried over Na_2SO_4, filtered and the solvent was removed under reduced pressure. The residue was then heated at 100 °C with H_2O (2.50 mL, 2.0 M) and NaOH (460.0 mg, 11.50 mmol, 2.30 equiv.) for 20 h, then, once cooled to room temperature, acidified with aqueous H_2SO_4 (3 M) and extracted three with Et_2O. The organic layers were gathered, dried over Na_2SO_4, filtered and the solvent removed under reduced pressure. Purification by column chromatography (SiO_2, Heptane/EtOAc, 100:0 to 7:3) afforded the desired product as a light-yellow oil (706.0 mg, 3.21 mmol, 64%).

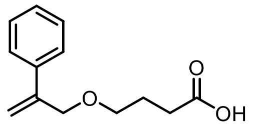

Chemical Formula: $C_{13}H_{16}O_3$
Exact Mass: 220,1099
Molecular Weight: 220,2680

R$_f$: 0.20 (Heptane/EtOAc, 1:1).

^{1}H NMR (400 MHz, $CDCl_3$) δ 7.48 – 7.42 (m, 2H), 7.38 – 7.27 (m, 3H), 7.18 (brs, 1H), 5.64 – 5.43 (m, 1H), 5.32 (d, *J* = 1.3 Hz, 1H), 4.47 – 4.26 (m, 2H), 3.53 (t, *J* = 6.1 Hz, 2H), 2.41 (t, *J* = 7.3 Hz, 2H), 1.91 (ddd, *J* = 13.4, 7.2, 6.2 Hz, 2H).

^{13}C NMR (101 MHz, $CDCl_3$) δ 178.4, 144.4, 138.8, 128.5 (2C), 128.0, 126.2 (2C), 114.4, 73.0, 69.0, 30.9, 24.9.

IR (neat) ν_{max} (cm^{-1}): 3084, 3057, 3030, 2933, 2856, 1704, 1445, 1412, 1120, 1081, 905, 779, 710.

HRMS (ESI+): *m/z* calculated for $[M+Na]^+$ = 243.0992, *m/z* found = 243.0993.

34: (*R*)-1-(2-(methoxymethyl)pyrrolidin-1-yl)-4-((2-phenylallyl)oxy)butan-1-one (spectra)

Based on a related procedure:[12] A solution of oxalyl chloride (84.0 µL, 0.99 mmol, 3.00 equiv.) in anhydrous CH_2Cl_2 (0.50 mL, 3.8 M) was added dropwise to a stirred solution of carboxylic acid **SI.13** (73.0 mg, 0.33 mmol, 1.00 equiv.) in anhydrous CH_2Cl_2 (2.00 mL) at 0 °C under argon. After 5 min, 1 drop (ca. 10.0 µL) of DMF was added and the mixture was stirred at 20 °C for 2 h. The solvent was then removed under reduced pressure, and the crude acyl chloride was dissolved into anhydrous CH_2Cl_2 (1.50 mL), after which (*R*)-(−)-2-(methoxymethyl)pyrrolidine (43.0 µL, 0.35 mmol, 1.05 equiv.) was added. After again cooling to 0 °C, pyridine (56.0 µL, 0.70 mmol, 2.10 equiv.) was added, and the reaction mixture was allowed to reach 20 °C and was stirred for 24 h. Water was added, the layers were separated and the aqueous layer was extracted three times with CH_2Cl_2. The organic layers were gathered, dried over Na_2SO_4, filtered and the solvent removed under reduced pressure. Purification by column chromatography (SiO_2, Heptane/EtOAc, 100:0 to 2:8) afforded the desired product as a colorless oil (79.0 mg, 0.25 mmol, 75%).

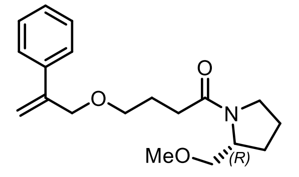

Chemical Formula: $C_{19}H_{27}NO_3$
Exact Mass: 317,1991
Molecular Weight: 317,4290

R_f: 0.30 (Heptane, EtOAc, 4:6).

2 rotamers by NMR, noted *Maj.* and *min* below.

^{1}H NMR (400 MHz, $CDCl_3$) δ 7.50 – 7.42 (m, 2H), 7.37 – 7.27 (m, 3H), 5.52 – 5.50 (m, 1H), 5.32 (dq, *J* = 4.1, 1.4 Hz, 1H), 4.36 (dt, *J* = 2.0, 1.0 Hz, 2H), 4.29 – 4.13 (m, 1H, *Maj.*), 4.00 – 3.90 (m, 1H, *min.*), 3.57 – 3.48 (m, 3H), 3.45 – 3.38 (m, 1H, *min.*), 3.39 – 3.34 (m, 1H, *Maj.*), 3.33 (s, 3H, *Maj.*), 3.31 (s, 3H, *min.*), 3.29 – 3.15 (m, 2H), 2.44 – 2.35 (m, 2H, *min.*), 2.31 – 2.22 (m, 2H, *Maj.*), 2.02 – 1.80 (m, 6H).

^{13}C NMR (101 MHz, $CDCl_3$) δ 172.0 (*min.*), 171.7 (*Maj.*), 144.6, 139.0 (*min.*), 139.0 (*Maj.*), 128.4, 127.9 (*min.*), 127.8 (*Maj.*), 126.3 (*Maj.*), 126.2 (*min.*), 114.4 (*Maj.*), 114.2 (*min.*), 74.1 (*min.*), 72.8 (*Maj.*), 72.8 (*min.*), 72.5 (*Maj.*), 69.5 (*min.*), 69.4 (*Maj.*), 59.2 (*min.*), 59.1 (*Maj.*), 57.0 (*min.*), 56.4 (*Maj.)*, 47.3 (*Maj.*), 45.7 (*min.*), 31.5 (*Maj.*), 30.8 (*min.*), 28.8 (*min.*), 27.6 (*Maj.*), 25.5 (*min.*), 24.9 (*Maj.*), 24.2 (*Maj.*), 22.0 (*min.*).

IR (neat) ν_{max} (cm^{-1}): 3083, 3057, 2924, 2873, 1640, 1418, 1116, 904, 781, 712.

HRMS (ESI+): *m/z* calculated for $[M+Na]^+$ = 340.1883, *m/z* found = 340.1875.

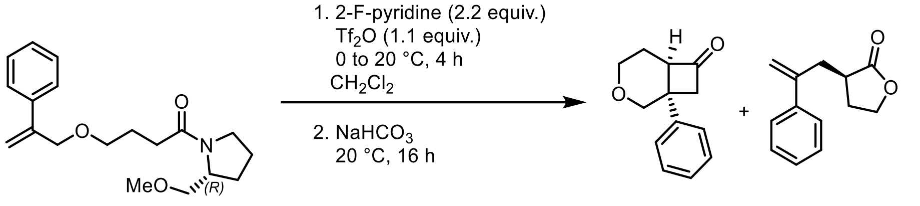

34

31c
47% (63%)
e.r. 58:42

32c
4% (4%)
e.r. 76:24

Following the general procedure for the (2+2)-cycloaddition, using **34** (50.0 mg, 0.16 mmol, 1.00 equiv.), CH_2Cl_2 (1.60 mL), 2-fluoro-pyridine (30.0 µL, 0.35 mmol, 2.20 equiv.), Tf_2O (29.0 µL, 0.17 mmol, 1.10 equiv.) to afford the desired cycloadduct **31c** (15.1 mg, 0.070 mmol, 47%) and the lactone **32c** (3.5 mg, 0.005 mmol, 4%). (Absolute configuration unknown).

31c, chiral HPLC:

Column Lux-3 Cellulose-3 250x4.6 mm, 3µm, Heptane/*i*-PrOH, 80:20, flow 0.7 mL/min, 25 °C, e.r. 58:42.

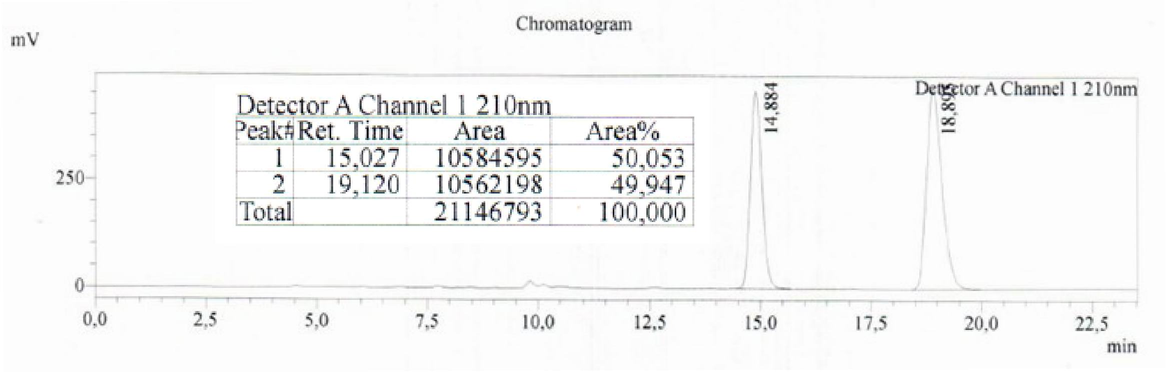

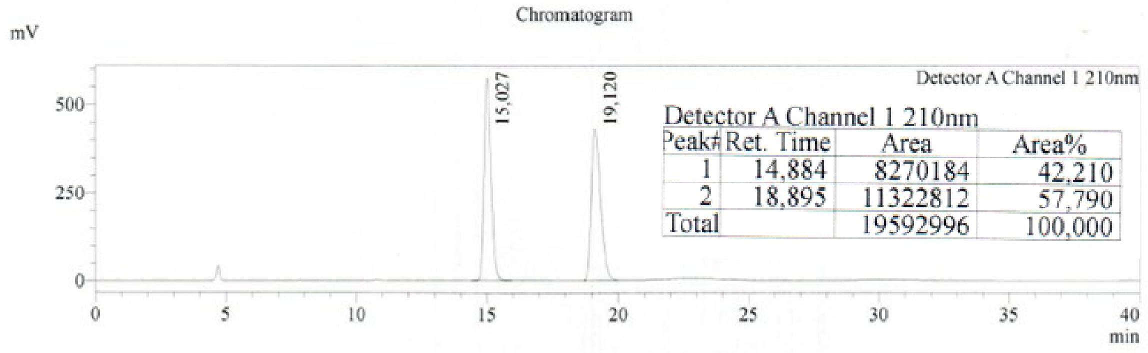

32c, chiral HPLC:

Column Chiralpak IH-3 150x4.6 mm, (Heptane+0.1% *i*-PrOH)/*i*-PrOH, 95:5, 1 mL/min, 25 °C, e.r. 76:24.

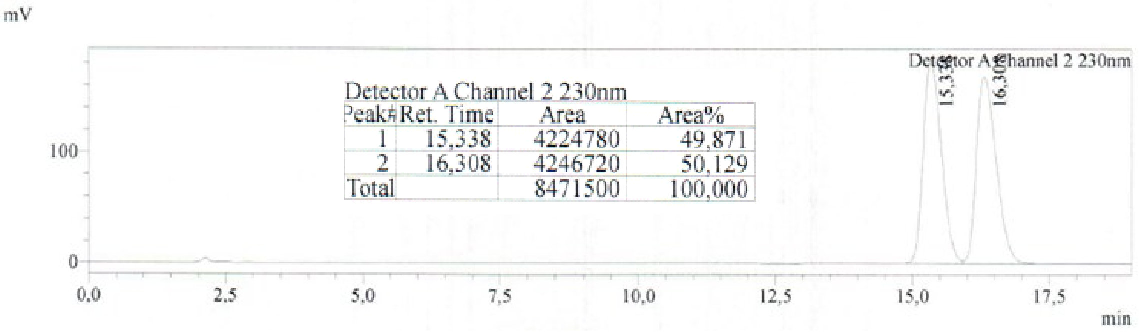

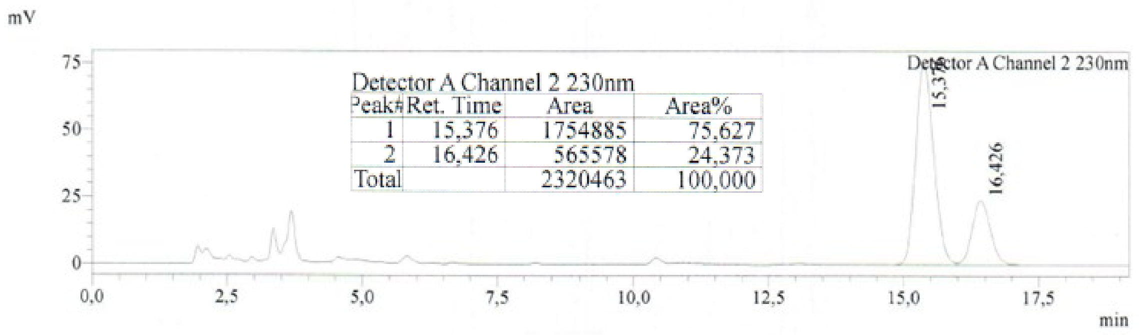

2.5. Grignard reagent addition to cyclobutanone derivatives

13_{PhCl}: *Cis*-7-(4-chlorophenyl)-1-methyl-3-oxabicyclo[4.2.0]octan-7-ol (spectra)

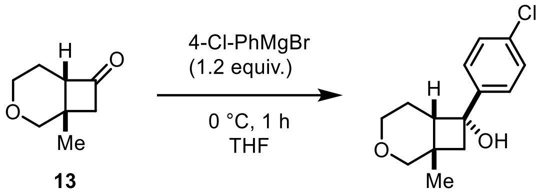

13_{PhCl}
63%, d.r. >20:1

A dry flask under argon atmosphere was charged with cyclobutanone **13** (15.0 mg, 0.11 mmol, 1.00 equiv.) and anhydrous THF (1.10 mL) and was cooled to 0 °C. A 1 M solution of 4-chlorophenylmagnesium bromide in THF (130.0 µL, 0.128 mmol, 1.20 equiv.) was then added dropwise and the reaction was stirred at 0 °C for 1 h. A NH_4Cl saturated aqueous solution was added, and the aqueous layer was extracted three times with CH_2Cl_2. The organic layers were gathered, dried over Na_2SO_4, filtered and the solvent removed under reduced pressure. Purification by column chromatography (SiO_2, CH_2Cl_2/EtOAc, 1:0 to 85:15) afforded the desired product as a white solid (17.1 mg, 0.070 mmol, 63%, d.r. >20:1). Single crystals suitable for X-ray diffraction spectroscopy were obtained from slow evaporation of a saturated CH_2Cl_2 solution (See section 4).

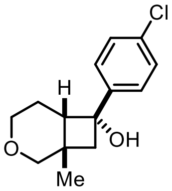

Chemical Formula: $C_{14}H_{17}ClO_2$
Exact Mass: 252,0917
Molecular Weight: 252,7380

R_f: 0.50 (CH_2Cl_2/EtOAc, 7:3).

^{1}H NMR (400 MHz, $CDCl_3$) δ 7.43 – 7.37 (m, 2H), 7.34 – 7.30 (m, 2H), 4.12 – 3.98 (m, 1H), 3.90 (dd, *J* = 11.5, 0.7 Hz, 1H), 3.65 (ddd, *J* = 11.2, 8.3, 5.2 Hz, 1H), 3.55 (s, 1H), 3.38 (d, *J* = 11.5 Hz, 1H), 2.55 (ddt, *J* = 8.1, 2.1, 1.1 Hz, 1H), 2.47 (dt, *J* = 13.2, 0.8 Hz, 1H), 2.28 (dd, *J* = 13.2, 1.0 Hz, 1H), 1.97 (dddd, *J* = 15.2, 8.1, 5.2, 2.0 Hz, 1H), 1.86 – 1.74 (m, 1H), 1.15 (s, 3H).

^{13}C NMR (101 MHz, $CDCl_3$) δ 145.7, 132.8, 128.5 (2C), 126.6 (2C), 76.4, 74.2, 65.1, 47.7, 47.6, 31.0, 25.3, 19.4.

IR (neat) ν_{max} (cm^{-1}): 3355, 2934, 2877, 1490, 1398, 1323, 1198, 1088, 1053, 1014, 959, 918, 825, 737, 568.

HRMS (ESI+): *m/z* calculated for $[M+Na]^+$ = 275.0809, *m/z* found = 275.0810.

14_{PhCl}: *Trans/trans*-7,15-bis(4-chlorophenyl)-1,9-dimethyl-3,11-dioxatricyclo[12.2.0.06,9]hexadecane-7,15-diol (spectra)

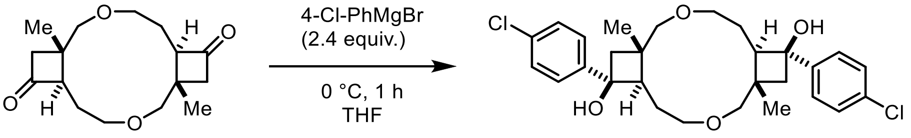

14

14_{PhCl}
68%, d.r. >20:1

A dry flask under argon atmosphere was charged with cyclobutanone dimer **14** (5.0 mg, 0.018 mmol, 1.00 equiv.) and anhydrous THF (0.50 mL) and was cooled to 0 °C. A 1 M solution of 4-chlorophenylmagnesium bromide in THF (9.2 µL, 43.0 µmol, 2.40 equiv.) was then added dropwise and the reaction was stirred at 0 °C for 1 h. A NH_4Cl saturated aqueous solution was added, and the aqueous layer was extracted three times with CH_2Cl_2. The organic layers were gathered, dried over Na_2SO_4, filtered and the solvent removed under reduced pressure. Purification by column chromatography (SiO_2, CH_2Cl_2/EtOAc, 1:0 to 9:1) afforded the desired product as a white solid (6.1 mg, 0.12 µmol, 68%). Single crystals suitable for X-ray diffraction spectroscopy were obtained from slow evaporation of a saturated CH_2Cl_2 solution (See section 4).

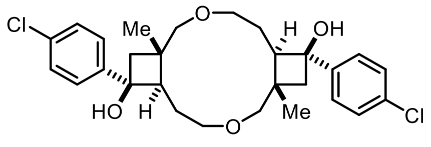

Chemical Formula: $C_{28}H_{34}Cl_2O_4$
Exact Mass: 504,1834
Molecular Weight: 505,4760

R_f: 0.50 (CH_2Cl_2/EtOAc, 9:1).

^{1}H NMR (700 MHz, $CDCl_3$) δ 7.29 – 7.27 (m, 4H), 7.12 – 7.09 (m, 4H), 3.71 (dt, J = 9.9, 4.1 Hz, 2H), 3.45 (dd, J = 11.0, 4.6 Hz, 2H), 3.32 (d, J = 9.3 Hz, 2H), 3.29 (ddd, J = 11.1, 9.9, 3.3 Hz, 2H), 2.93 (d, J = 9.3 Hz, 2H), 2.44 (d, J = 12.1 Hz, 2H), 1.87 – 1.72 (m, 8H), 1.29 (s, 6H).

^{13}C NMR (176 MHz, $CDCl_3$) δ 146.2 (2C), 132.8 (2C), 128.6 (4C), 126.3 (4C), 78.1 (2C), 75.8 (2C), 68.8 (2C), 43.5 (2C), 42.5 (2C), 38.0 (2C), 24.0 (2C), 19.5 (2C).

IR (neat) ν_{max} (cm^{-1}): 3433, 2925, 2851, 1764, 1596, 1490, 1120, 1092, 1012, 823.

HRMS (ESI+): *m/z* calculated for $[M+Na]^+$ = 527.1726, *m/z* found = 527.1727.

2.6. Concentration effect

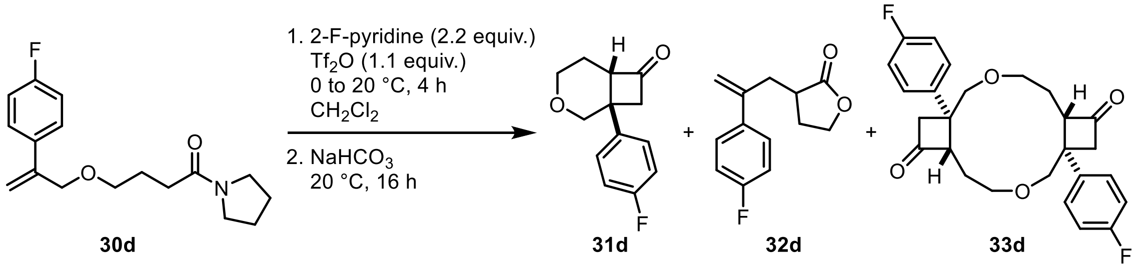

	31d	32d	33d
c = 0.50 M	20%	7%	21%
c = 0.25 M	33%	10%	19%
c = 0.10 M	38%	13%	13%
c = 0.05 M	47%	16%	11%
c = 0.02 M	56%	23%	3%

NMR yields using mesitylene as internal standard

2.7. Collidine adduct

16 : (*Z*)-2,4,6-trimethyl-1-(4-((2-methylallyl)oxy)-1-(pyrrolidin-1-yl)but-1-en-1-yl)pyridin-1-ium trifluoromethanesulfonate (spectra)

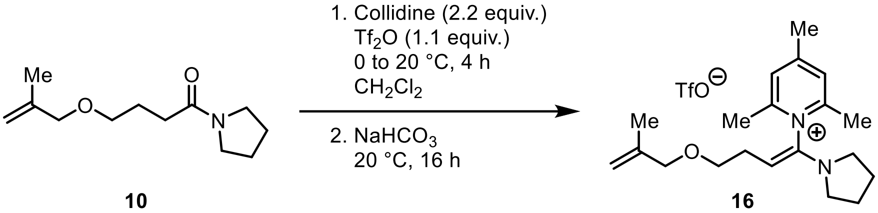

10 **16**

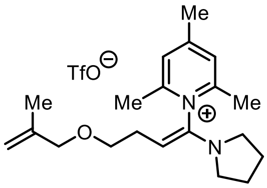

Chemical Formula: $C_{21}H_{31}F_3N_2O_4S$
Exact Mass: 464,1957
Molecular Weight: 464,5442

Following the general procedure for the (2+2)-cycloaddition: obtained from alkene-amide **10** (42.3 mg, 0.20 mmol), CH_2Cl_2 (2.00 mL), collidine (58.1 µL, 0.44 mmol) and Tf_2O (37.0 µL, 0.22 mmol). Purification by column chromatography (SiO_2, EtOAc 100% then DCM/MeOH 100:0 to 90:10).

Yield: 62% (58.0 mg, 0.13 mmol), 71% NMR using mesitylene as internal standard.

Yellow oil.

R_f: 0.40 (CH_2Cl_2/MeOH, 9:1).

^{1}H NMR (400 MHz, $CDCl_3$) δ 7.73 (s, 2H), 4.91 – 4.85 (m, 2H), 4.44 (t, *J* = 7.3 Hz, 1H), 3.80 (s, 2H), 3.36 (t, *J* = 5.8 Hz, 2H), 2.99 – 2.90 (m, 4H), 2.72 (s, 6H), 2.67 (s, 3H), 1.99 – 1.90 (m, 4H), 1.83 – 1.73 (m, 2H), 1.68 (s, 3H).

^{13}C NMR (151 MHz, $CDCl_3$) δ 161.3, 154.3 (2C), 142.0, 138.6, 128.6 (2C), 120.9 (d, *J* = 320.1 Hz), 112.5, 94.3, 75.1, 69.1, 47.7 (2C), 27.4, 25.1 (2C), 22.3, 20.6 (2C), 19.6.

^{19}F NMR (565 MHz, $CDCl_3$) δ -78.3.

IR (neat) ν_{max} (cm^{-1}): 3068, 2972, 2935, 2850, 1669, 1638, 1374, 1260, 1148, 1030, 637.

HRMS (ESI+): *m/z* calculated for [M-collidine]$^+$ = 194.1539, *m/z* found = 194.1540. *m/z* calculated for [M]$^+$ = 315.2431, *m/z* found = 315.2431.

HRMS (ESI-): *m/z* calculated for [M]$^-$ = 148.9526, *m/z* found = 148.9524.

3. NMR spectra

SI.1: (^{1}H NMR, $CDCl_3$, 400 MHz)

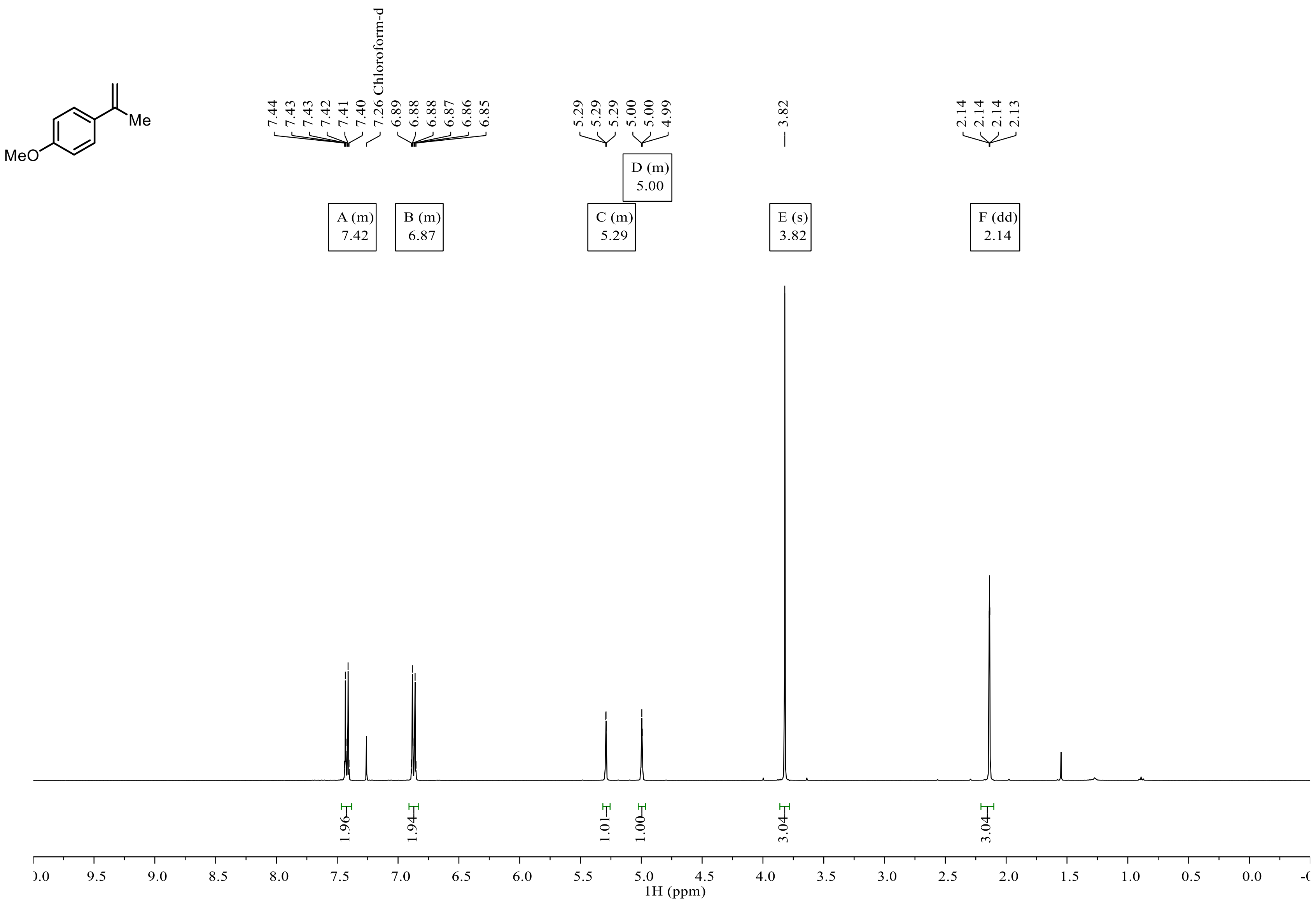

SI.2: (^{1}H NMR, $CDCl_3$, 400 MHz)

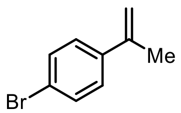

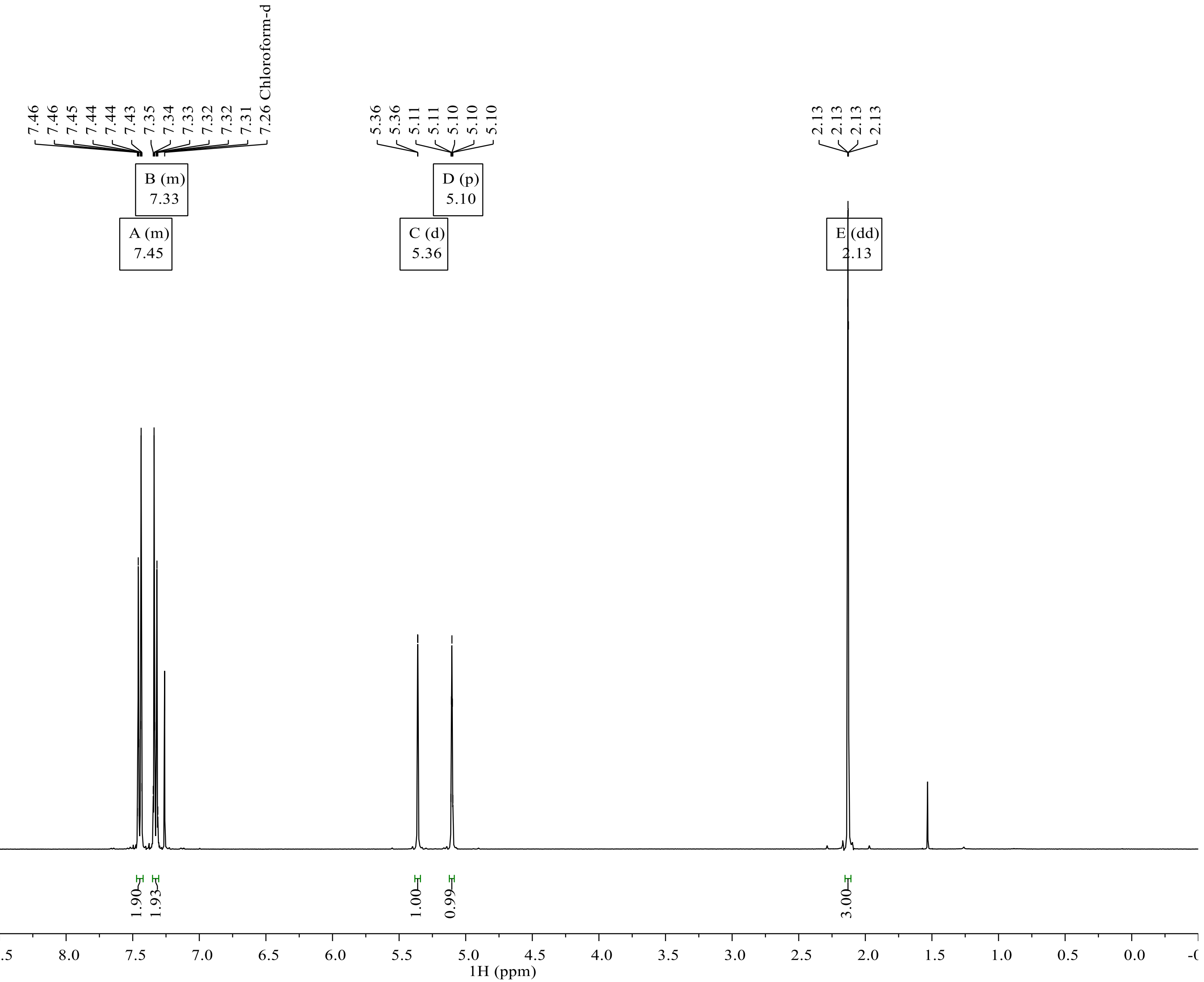

SI.3: (^{1}H NMR, $CDCl_3$, 400 MHz)

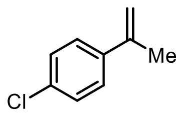

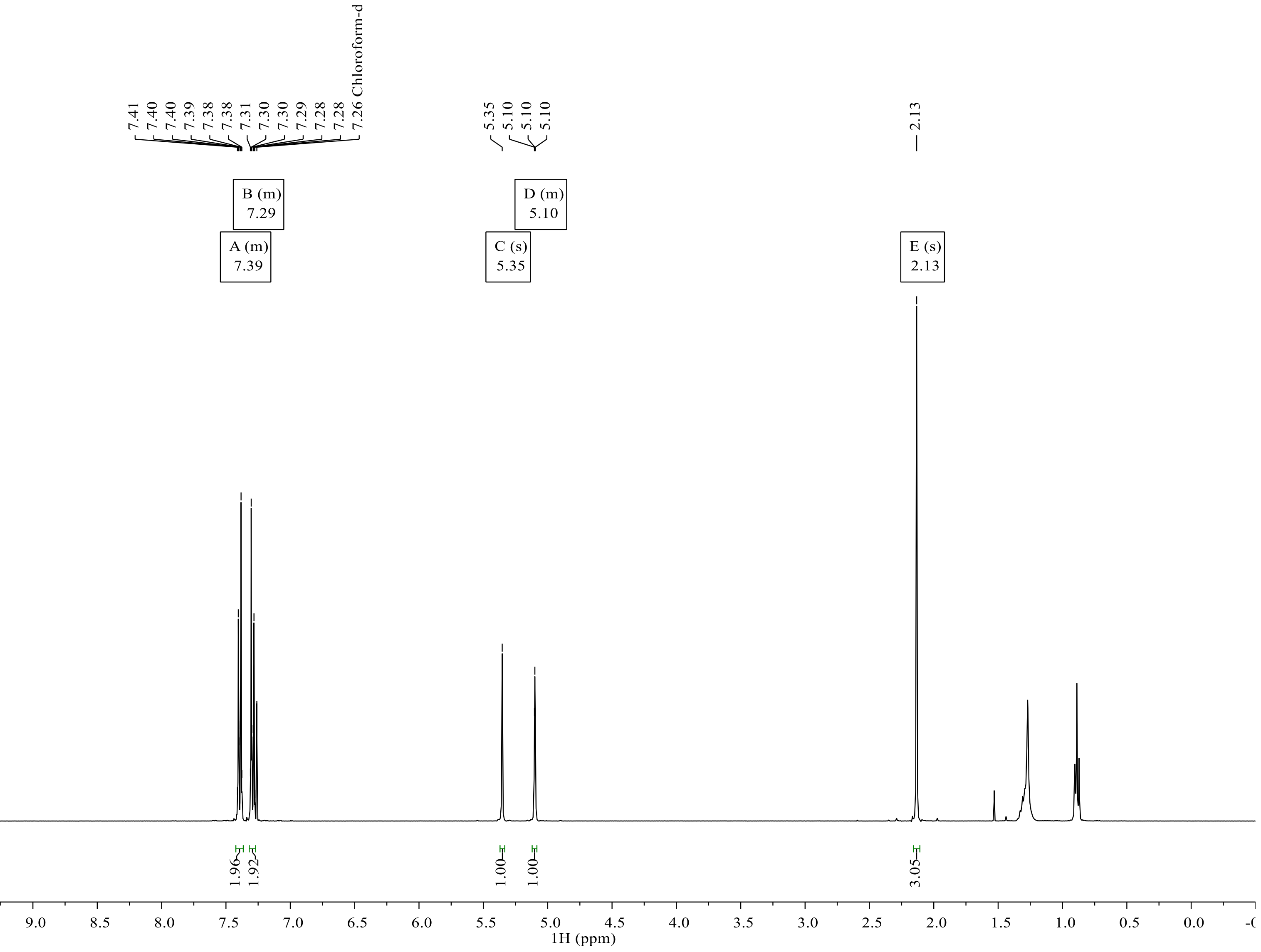

SI.4: (^{1}H NMR, $CDCl_3$, 200 MHz)

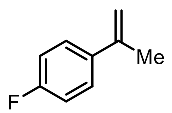

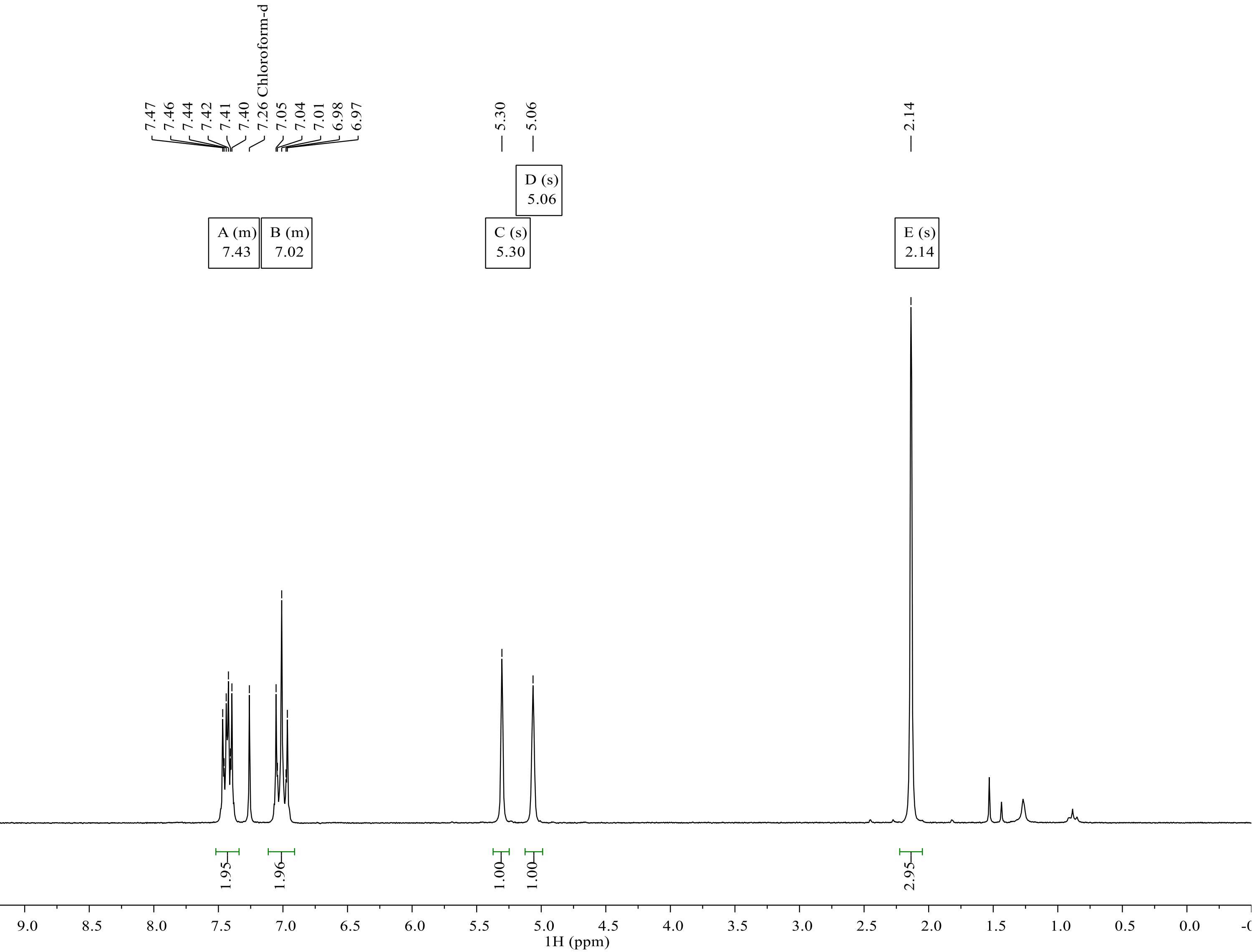

SI.5: (^{1}H NMR, $CDCl_3$, 400 MHz)

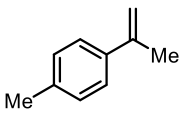

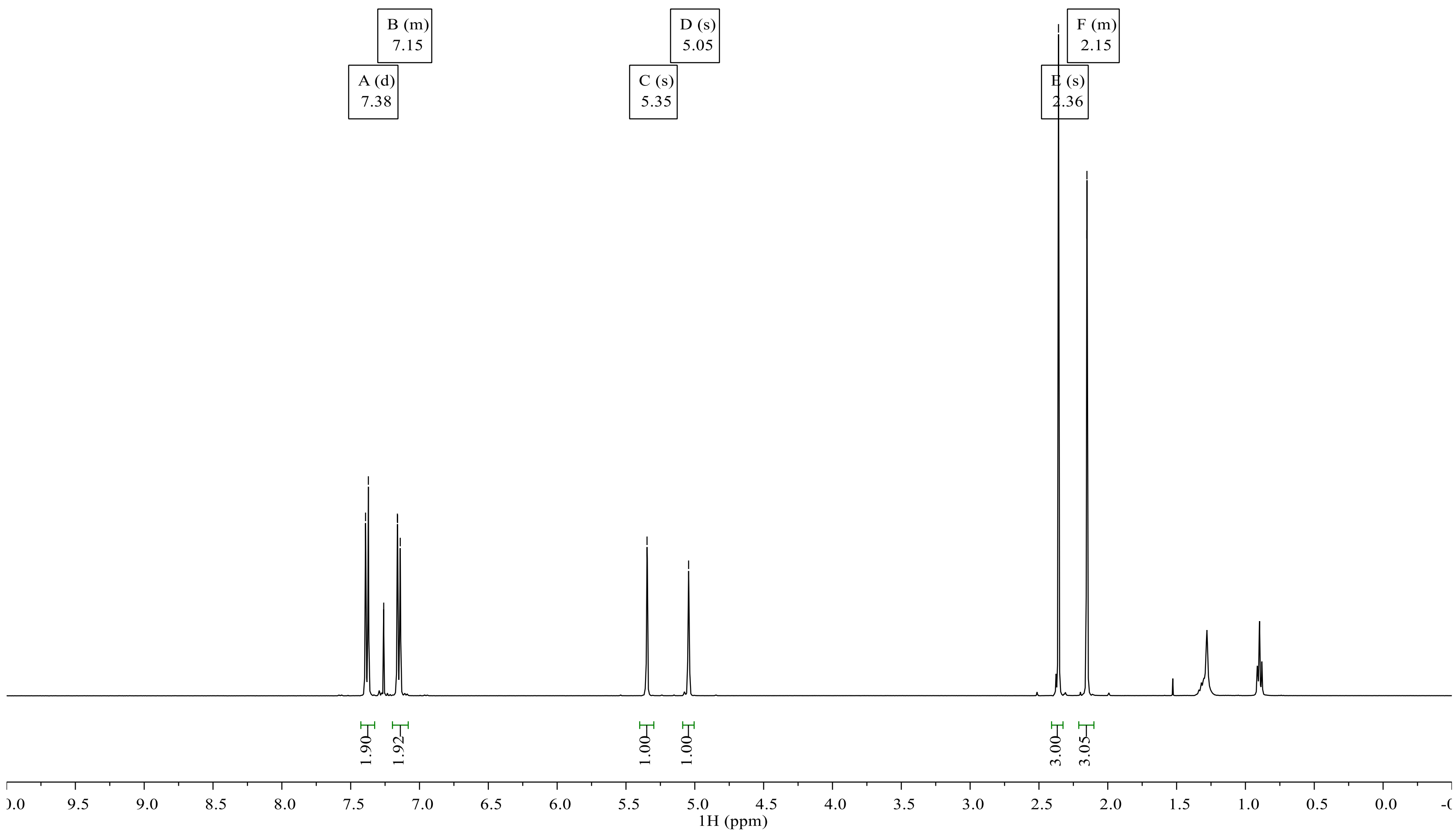

SI.7: (^{1}H NMR, $CDCl_3$, 200 MHz)

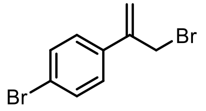

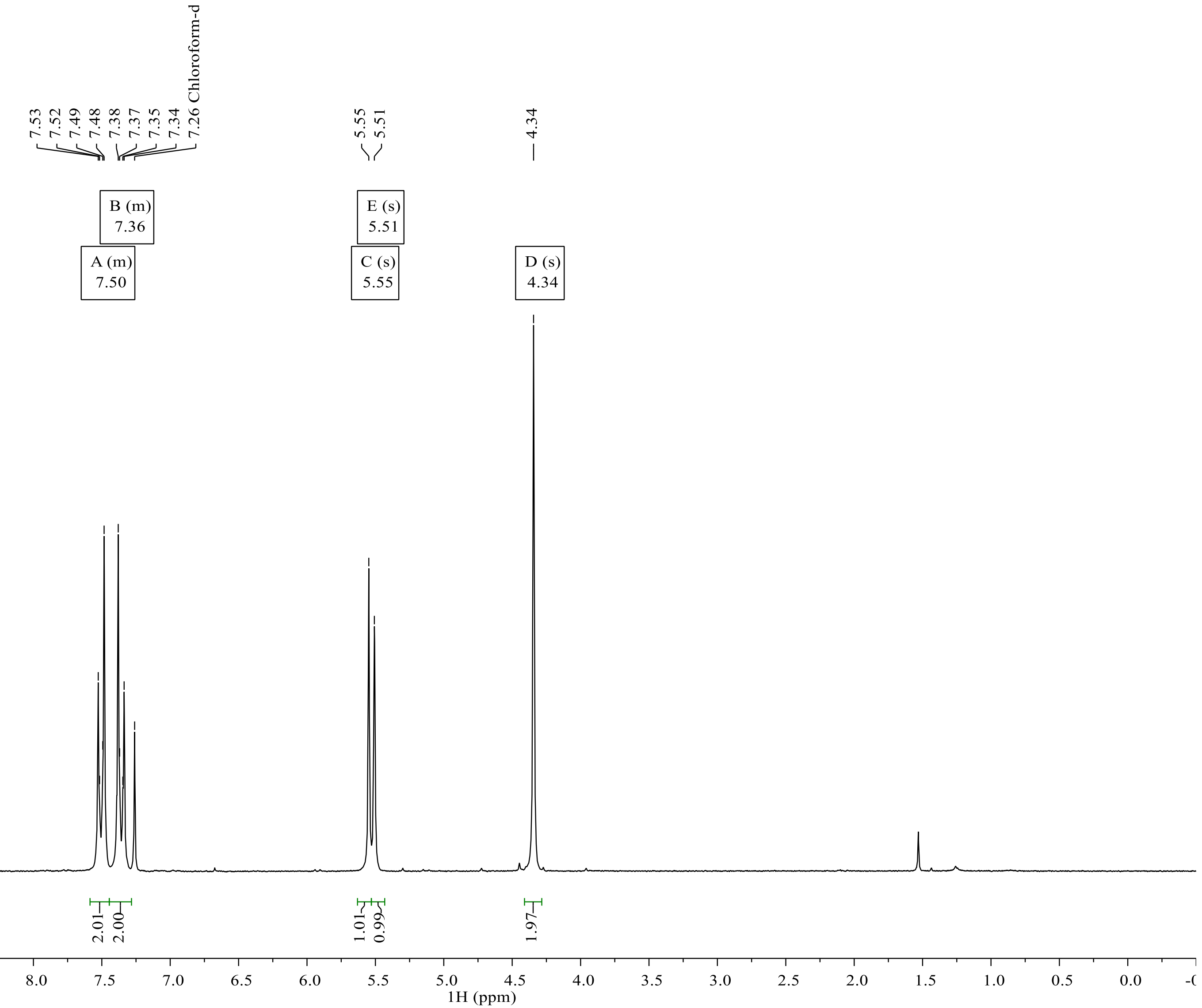

SI.8: (^{1}H NMR, $CDCl_3$, 400 MHz)

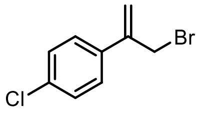

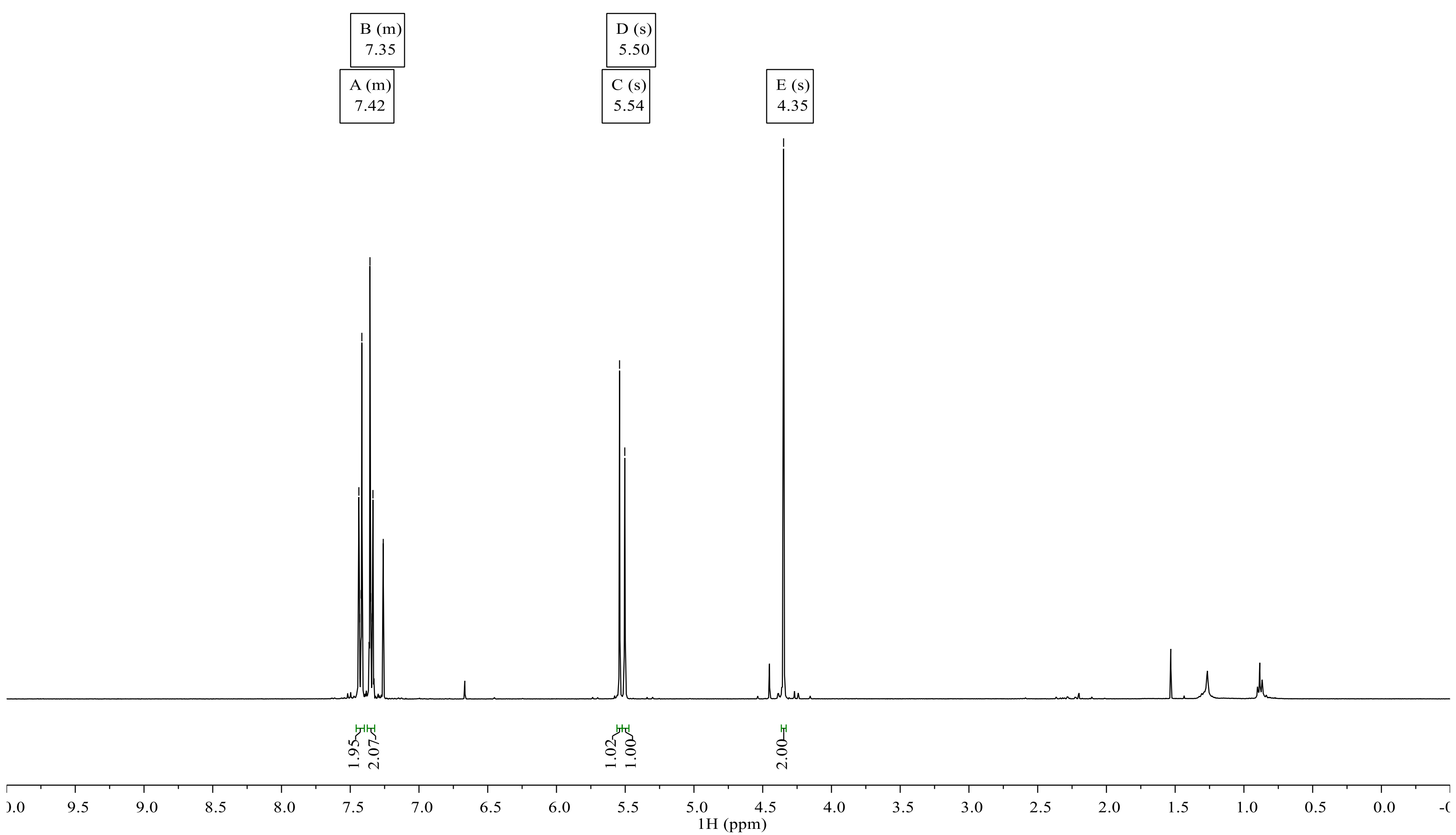

SI.9: (^{1}H NMR, $CDCl_3$, 200 MHz)

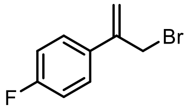

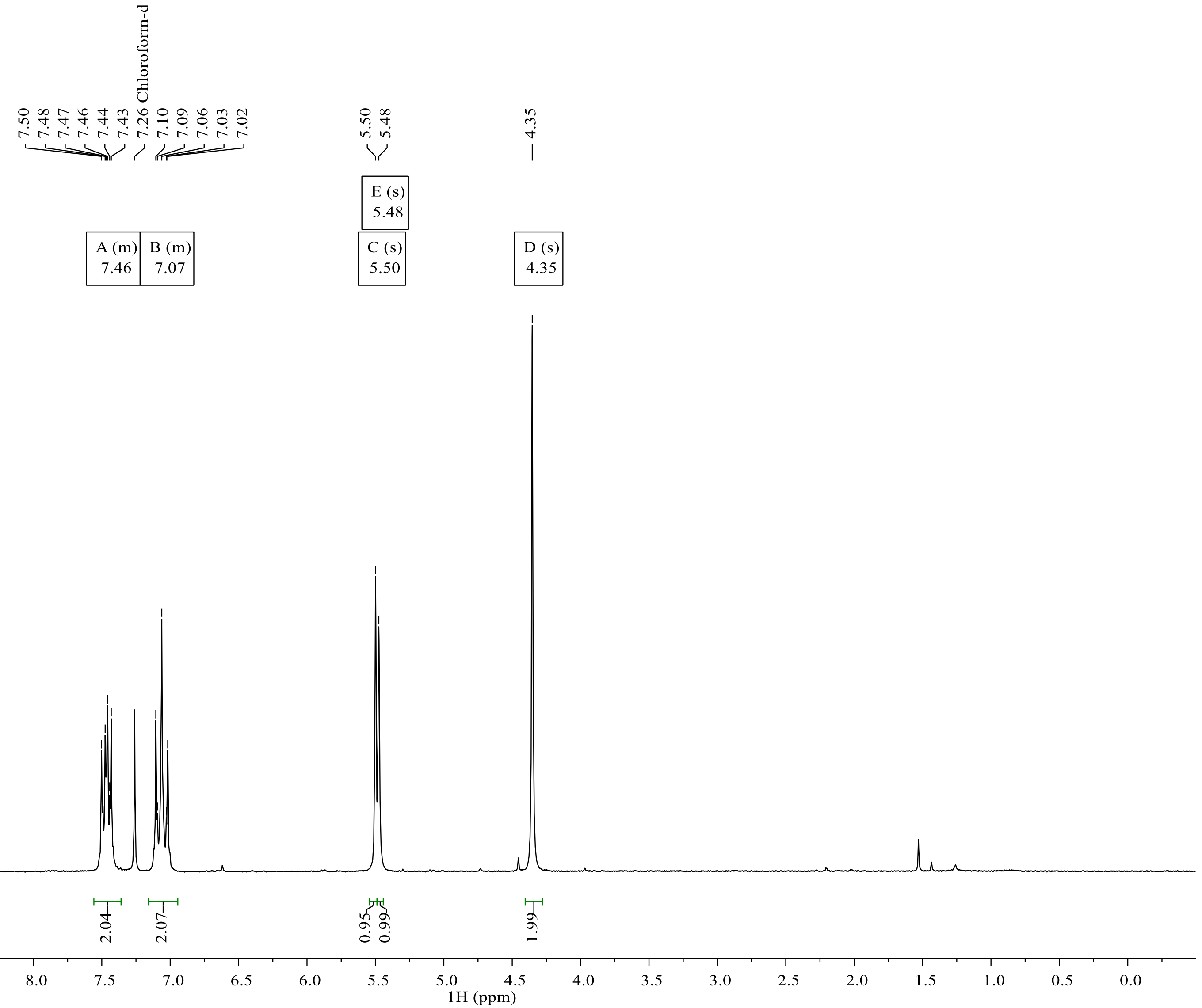

SI.10: (^{1}H NMR, $CDCl_3$, 400 MHz)

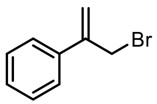

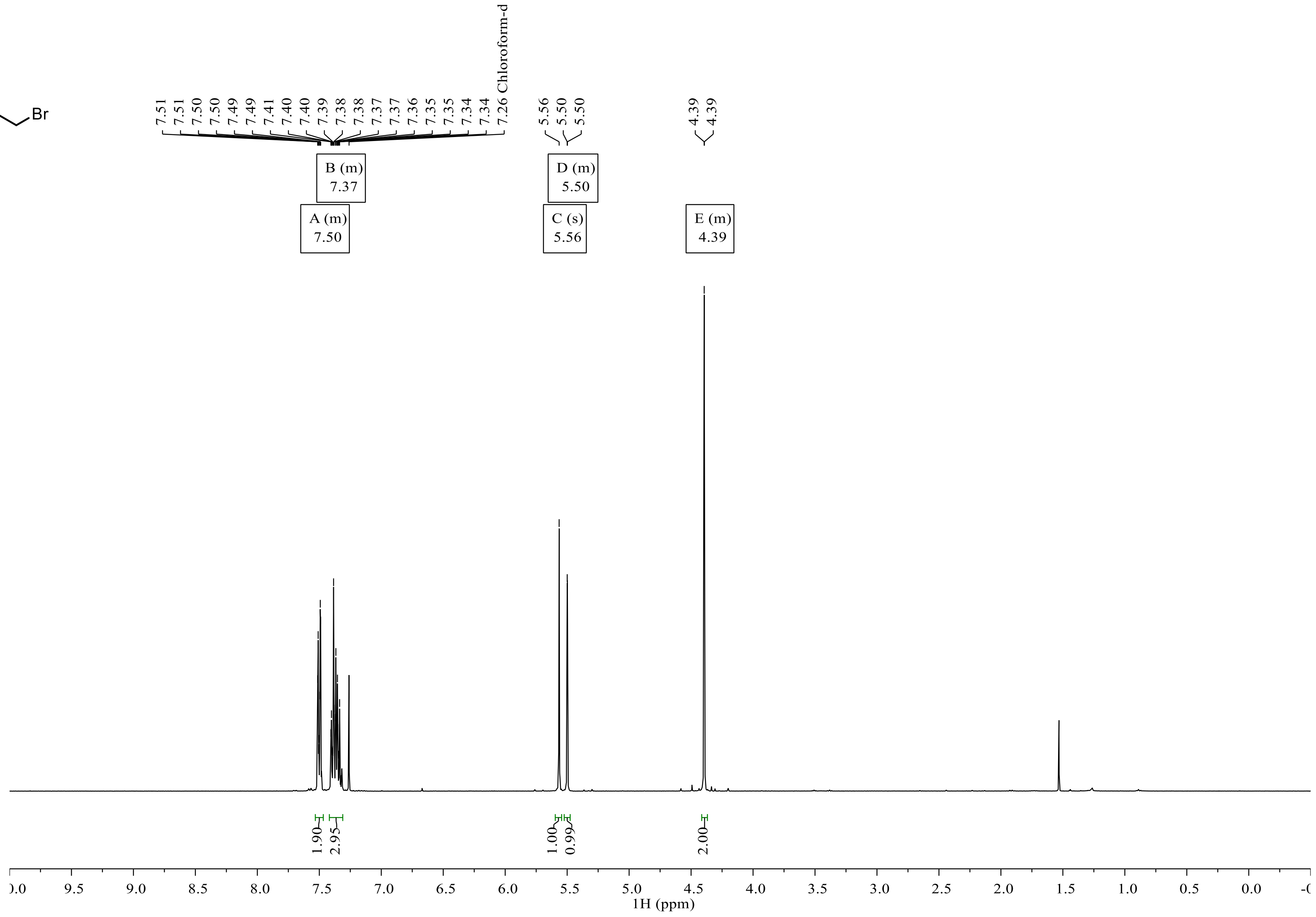

SI.12: (^{1}H NMR, $CDCl_3$, 200 MHz)

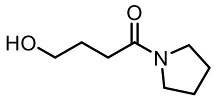

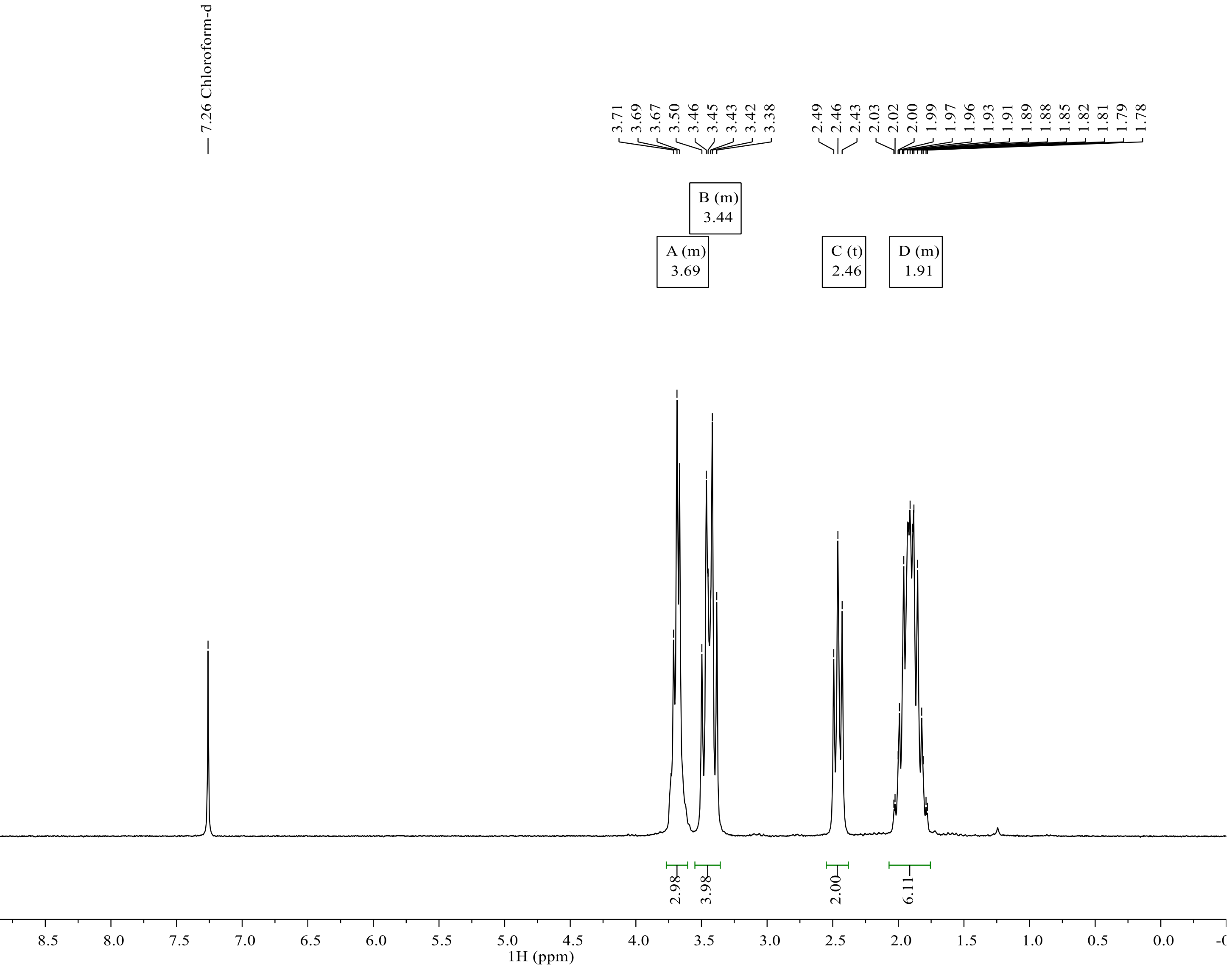

10: (^{1}H NMR, $CDCl_3$, 200 MHz)

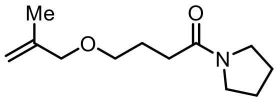

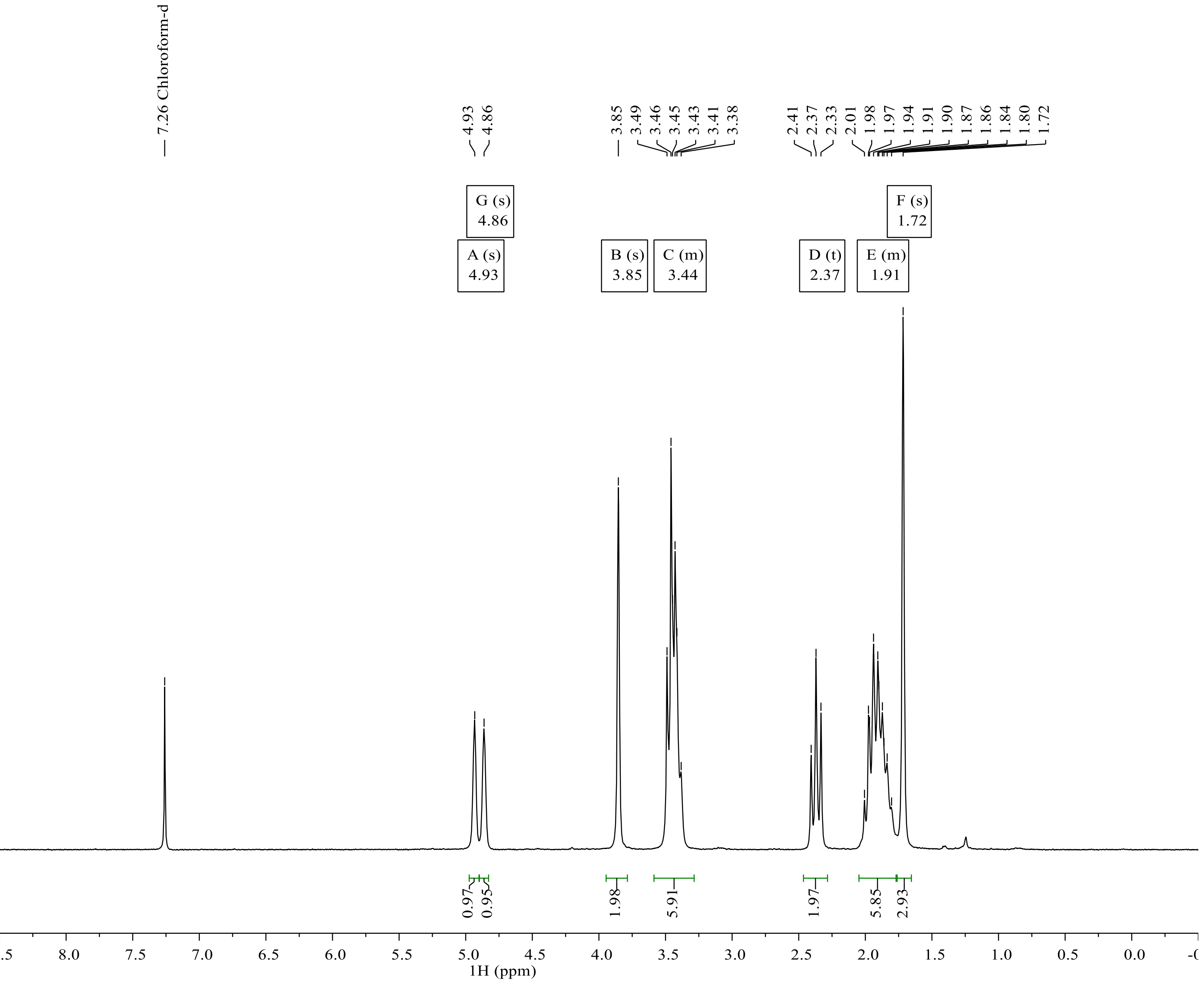

30a: (^{1}H NMR, $CDCl_3$, 700 MHz)

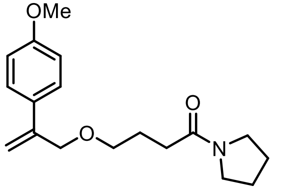

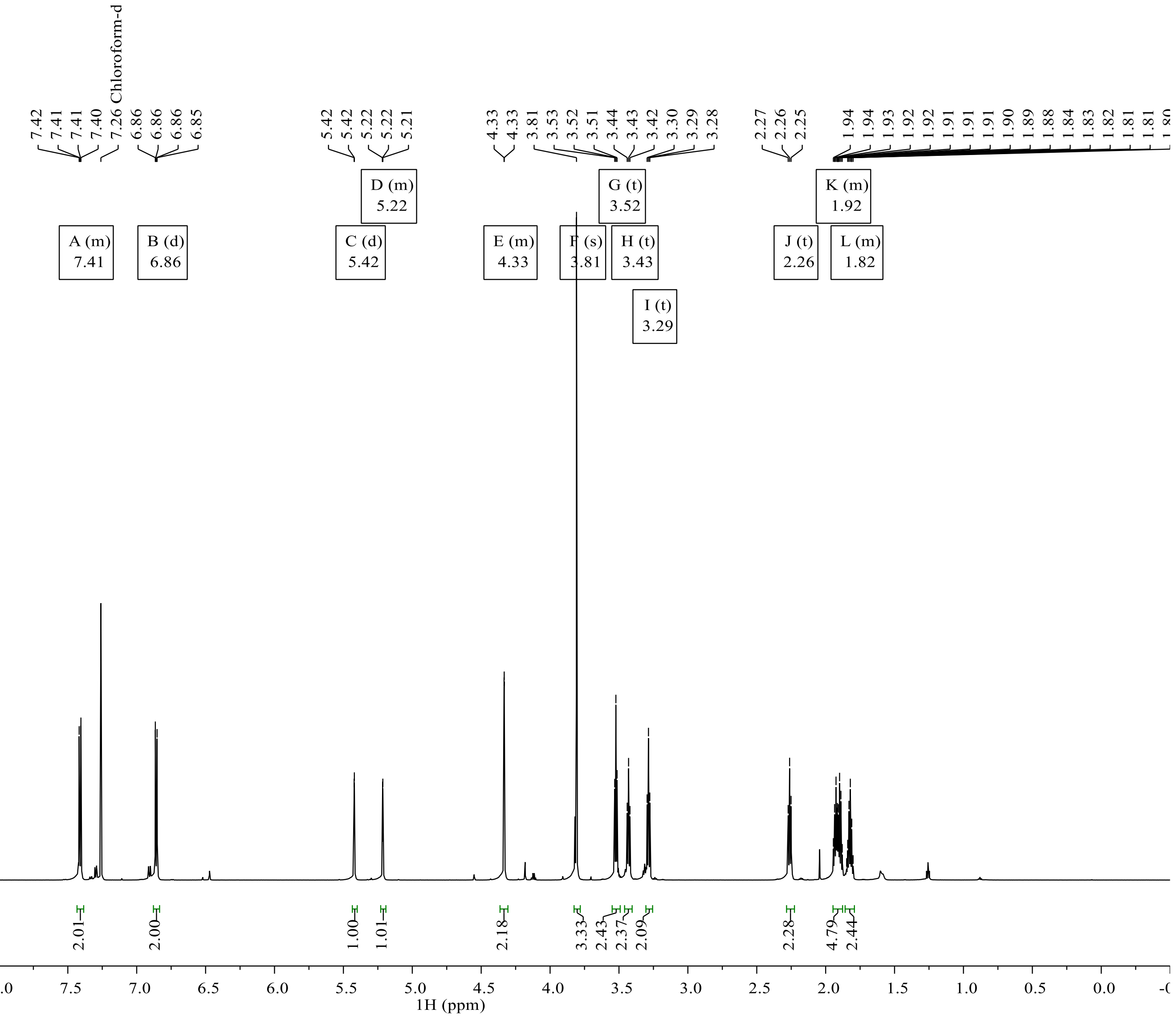

(^{13}C NMR, $CDCl_3$, 176 MHz)

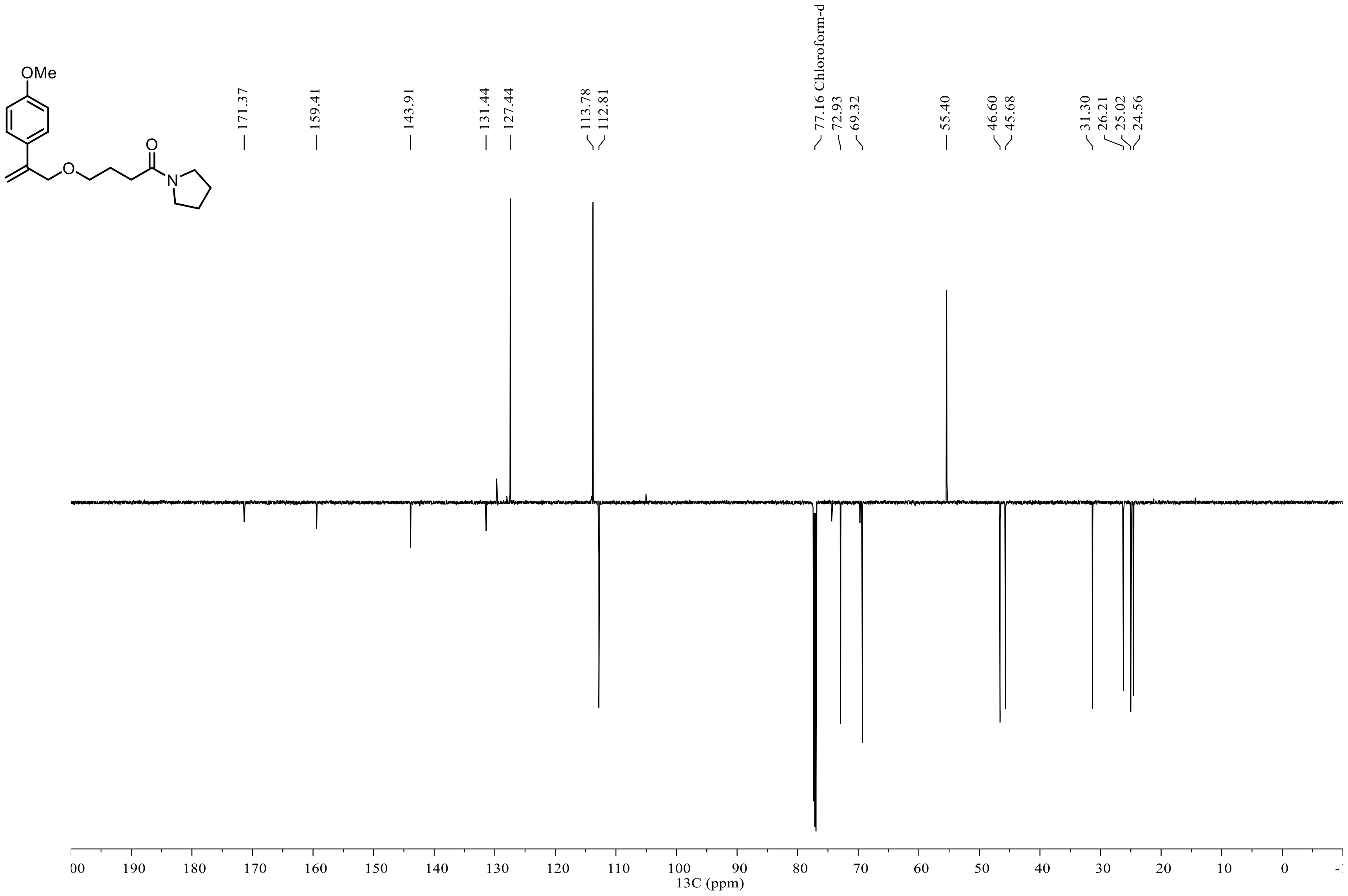

30b: (^{1}H NMR, $CDCl_3$, 400 MHz)

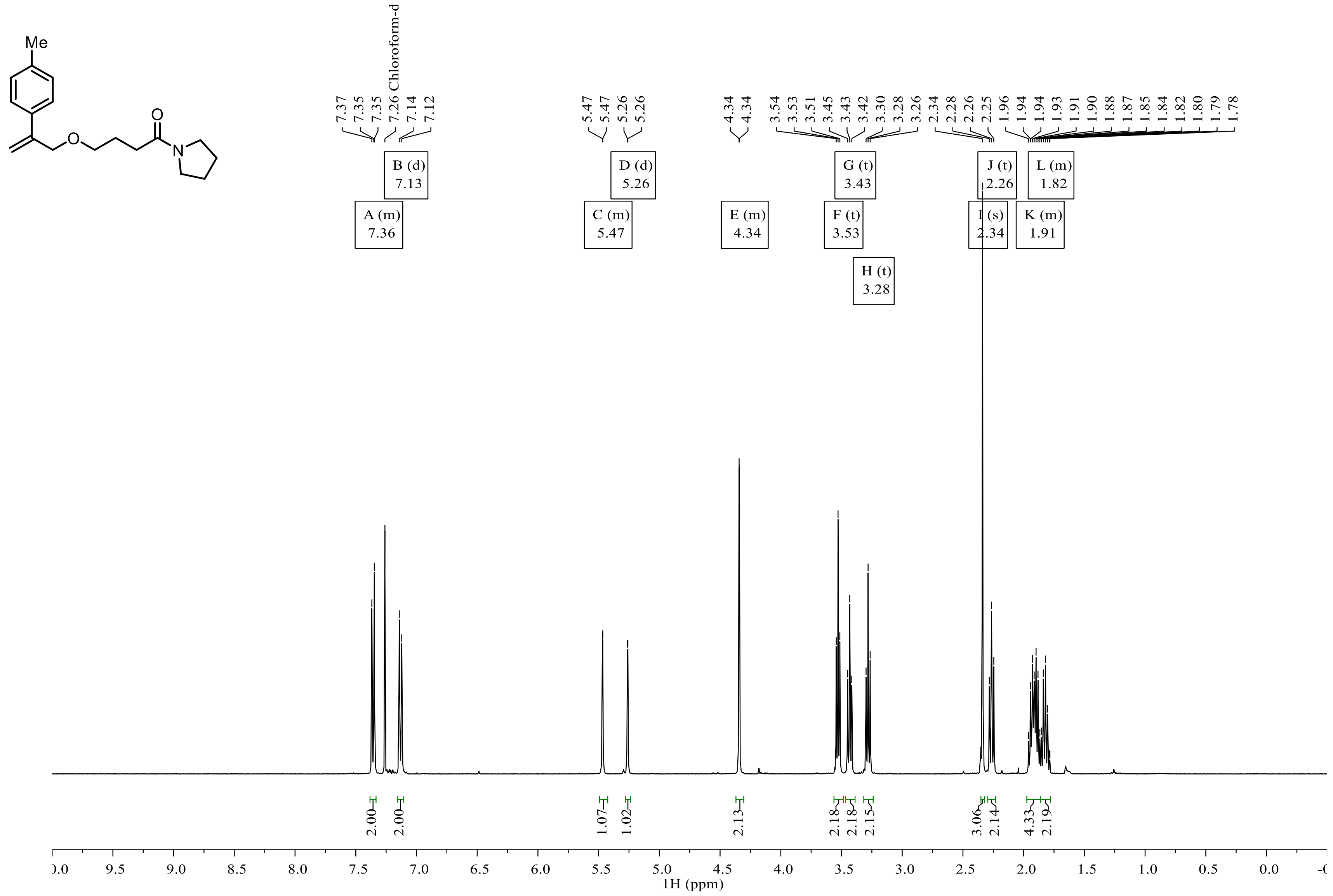

(^{13}C NMR, $CDCl_3$, 101 MHz)

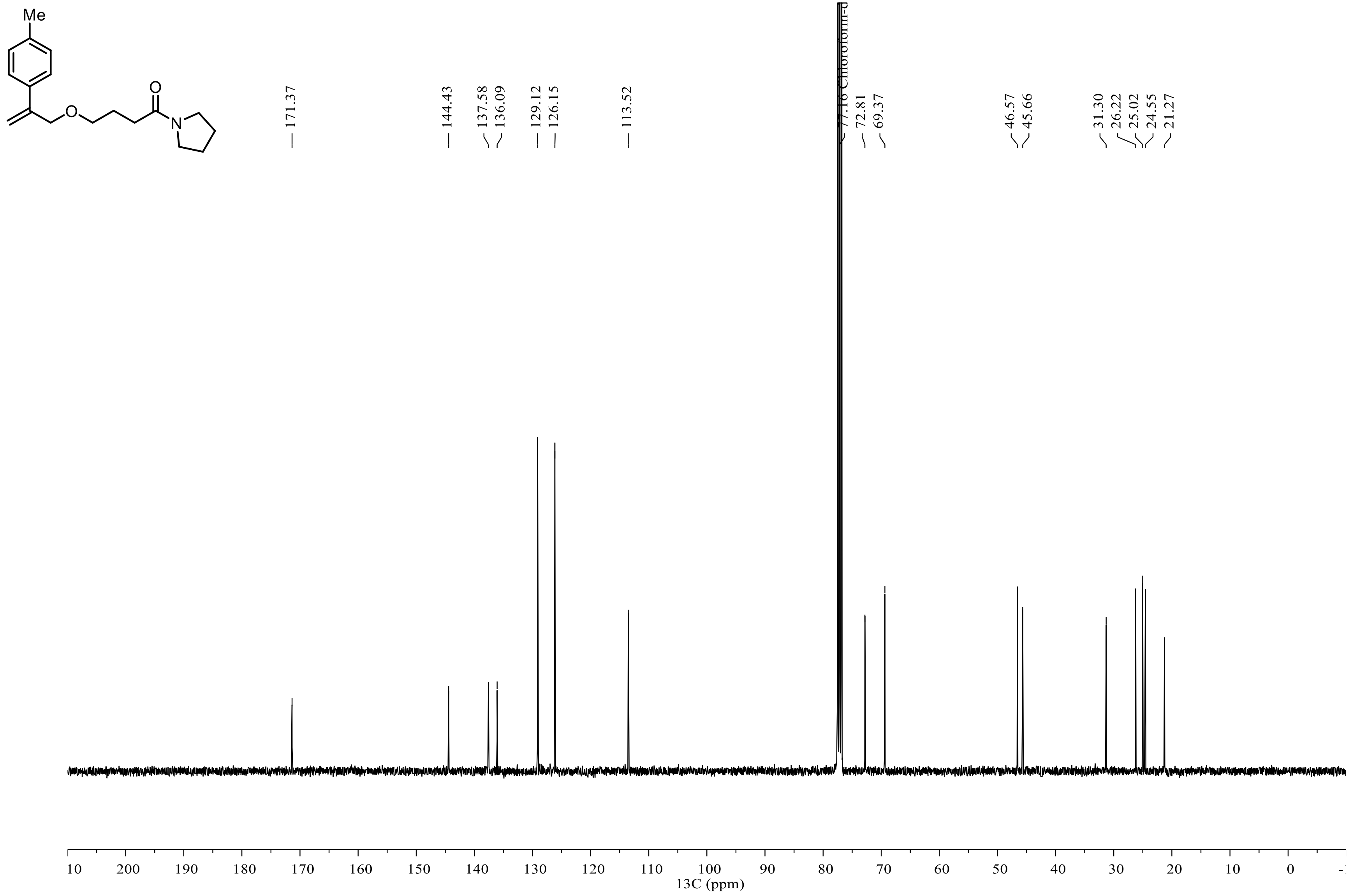

30c: (^{1}H NMR, $CDCl_3$, 400 MHz)

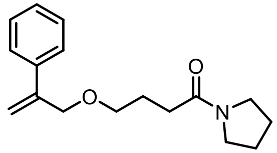

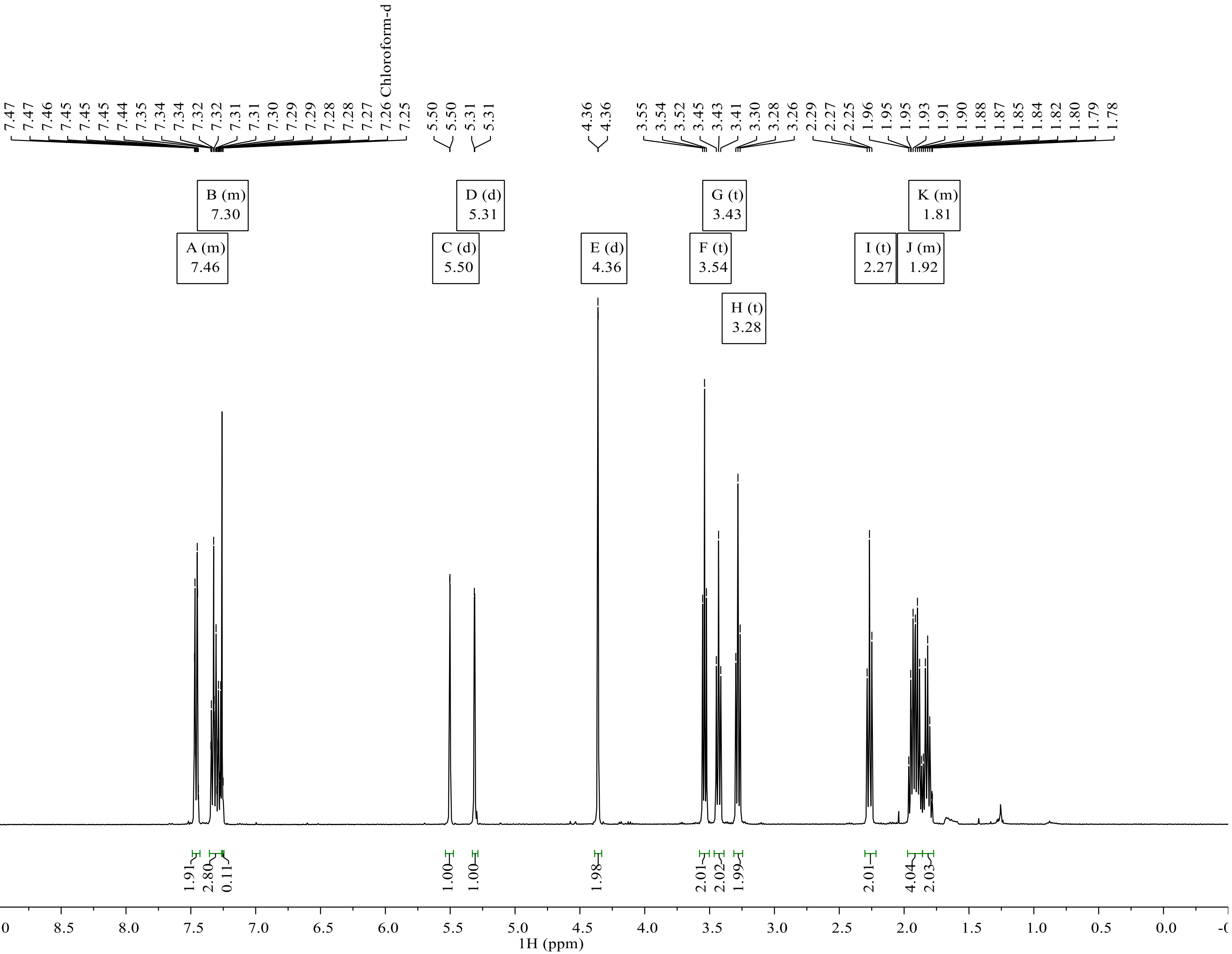

(^{13}C NMR, $CDCl_3$, 101 MHz)

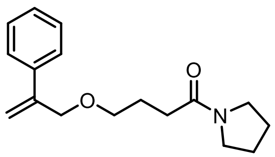

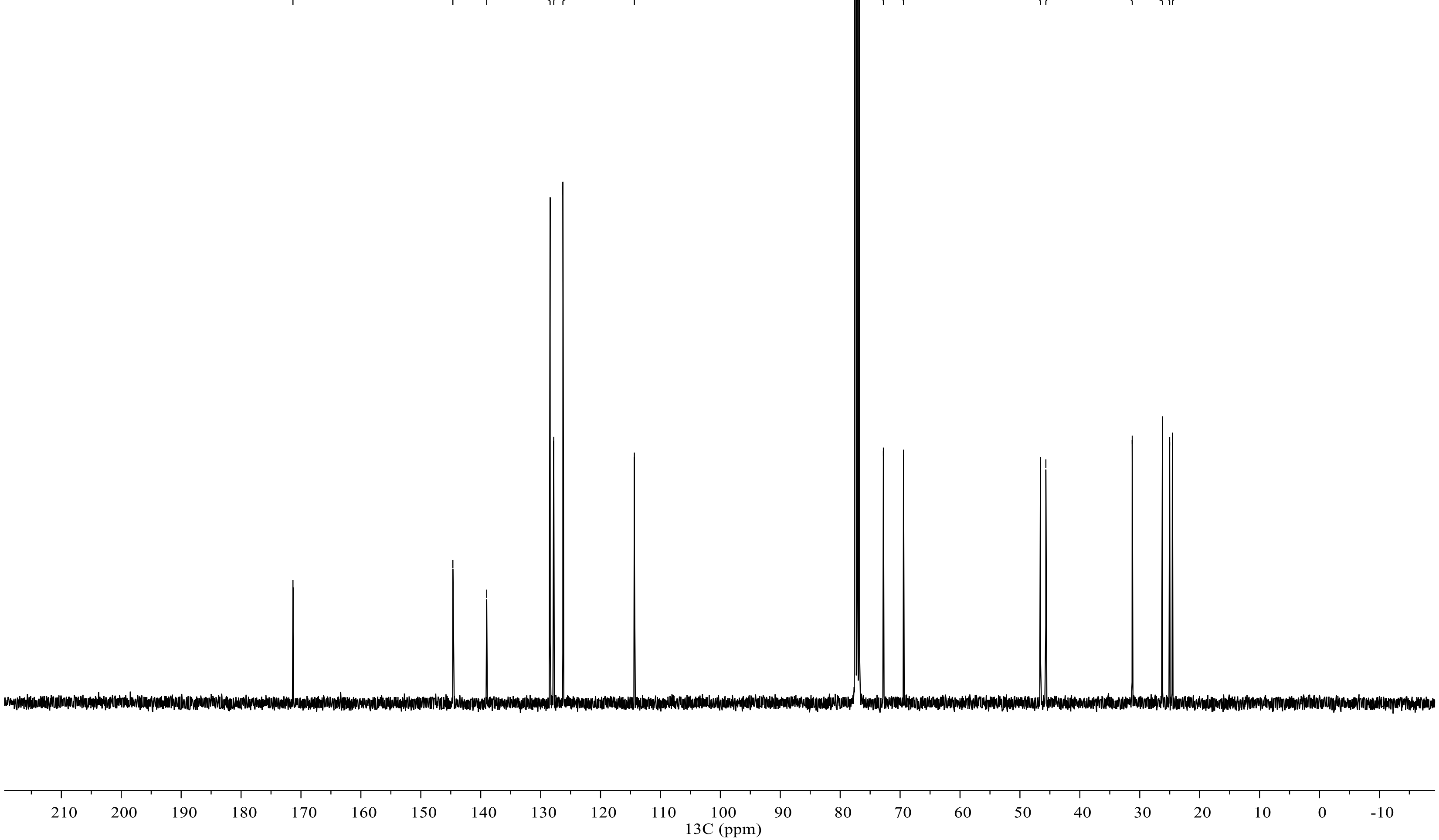

30d: (^{1}H NMR, $CDCl_3$, 400 MHz)

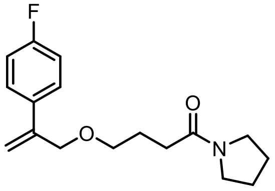

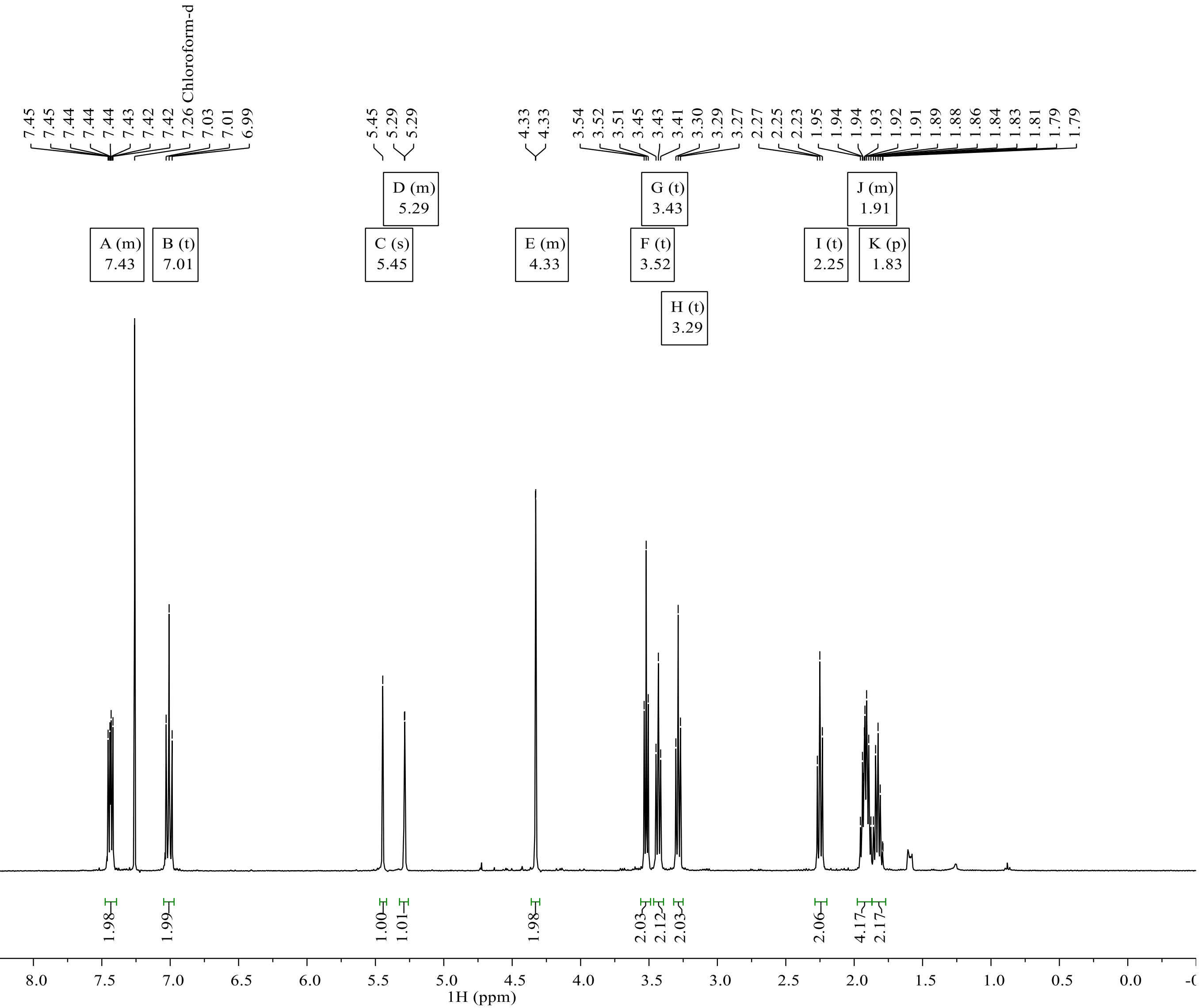

(^{13}C NMR, $CDCl_3$, 101 MHz)

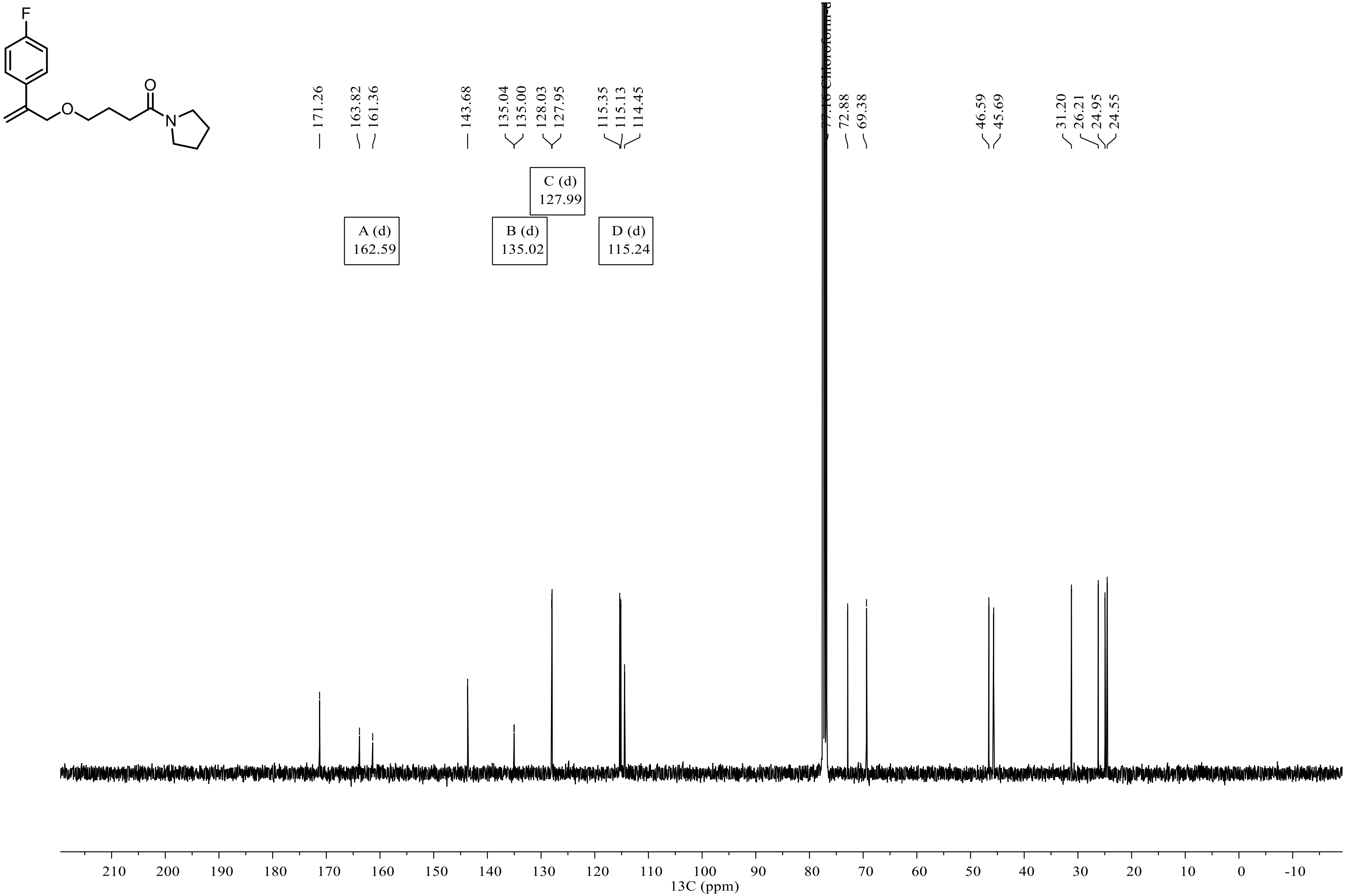

(^{19}F NMR, $CDCl_3$, 377 MHz)

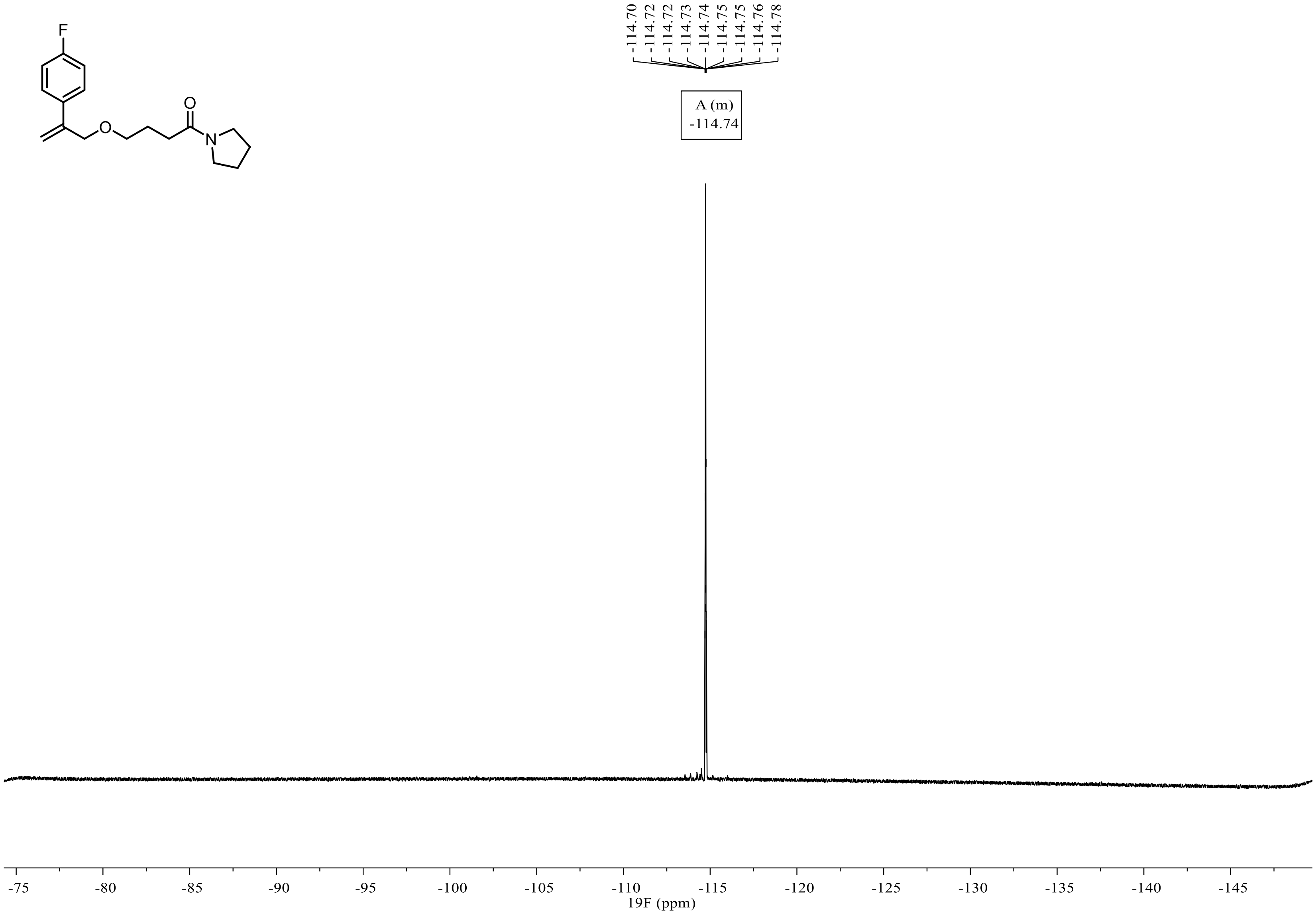

30e: (^{1}H NMR, $CDCl_3$, 400 MHz)

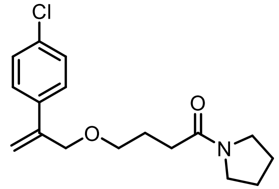

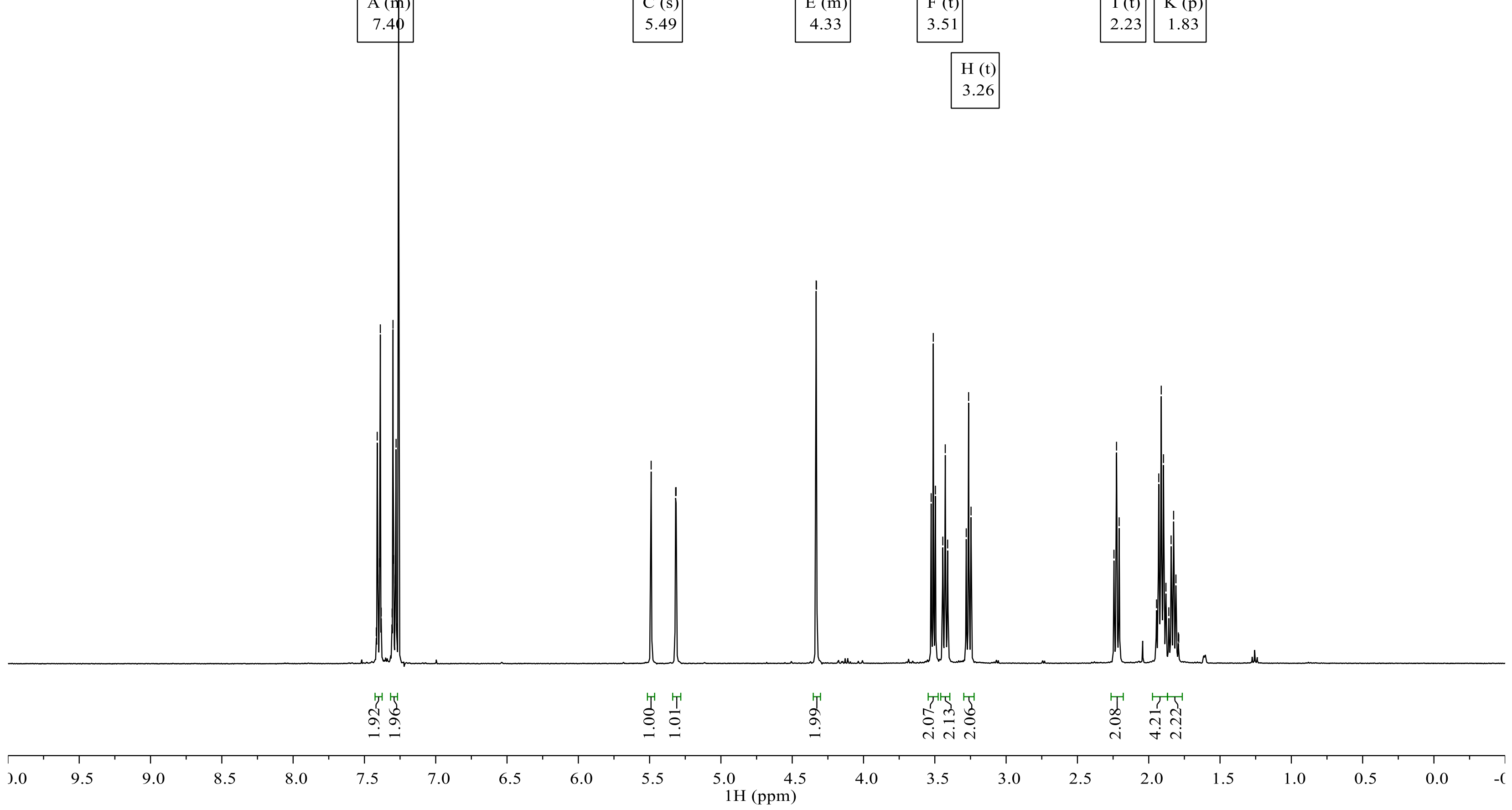

(^{13}C NMR, $CDCl_3$, 101 MHz)

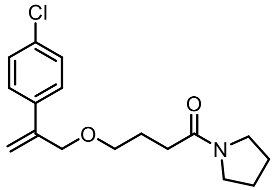

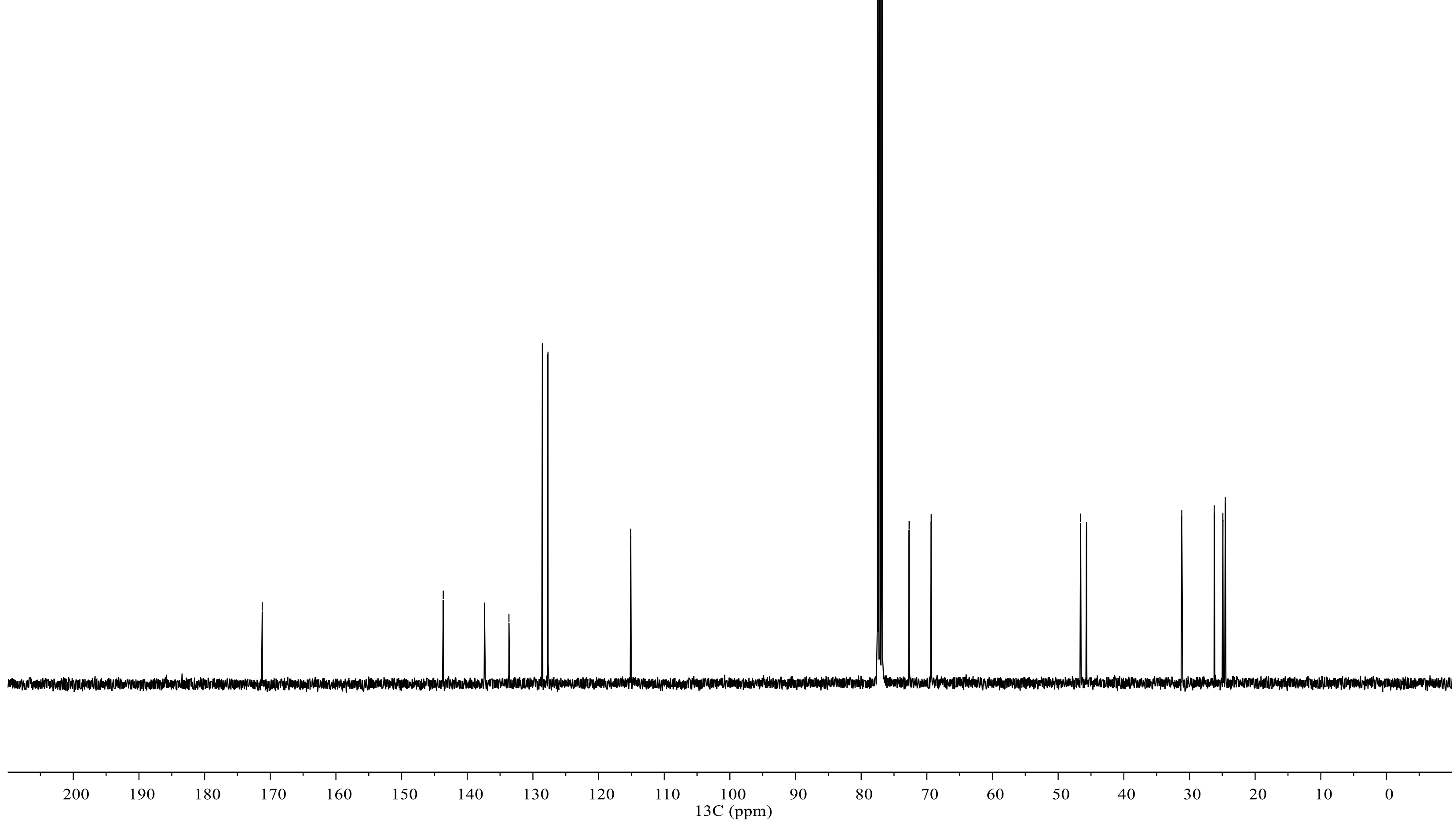

30f: (^{1}H NMR, $CDCl_3$, 700 MHz)

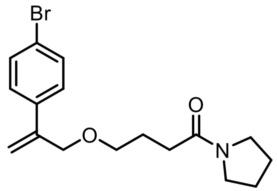

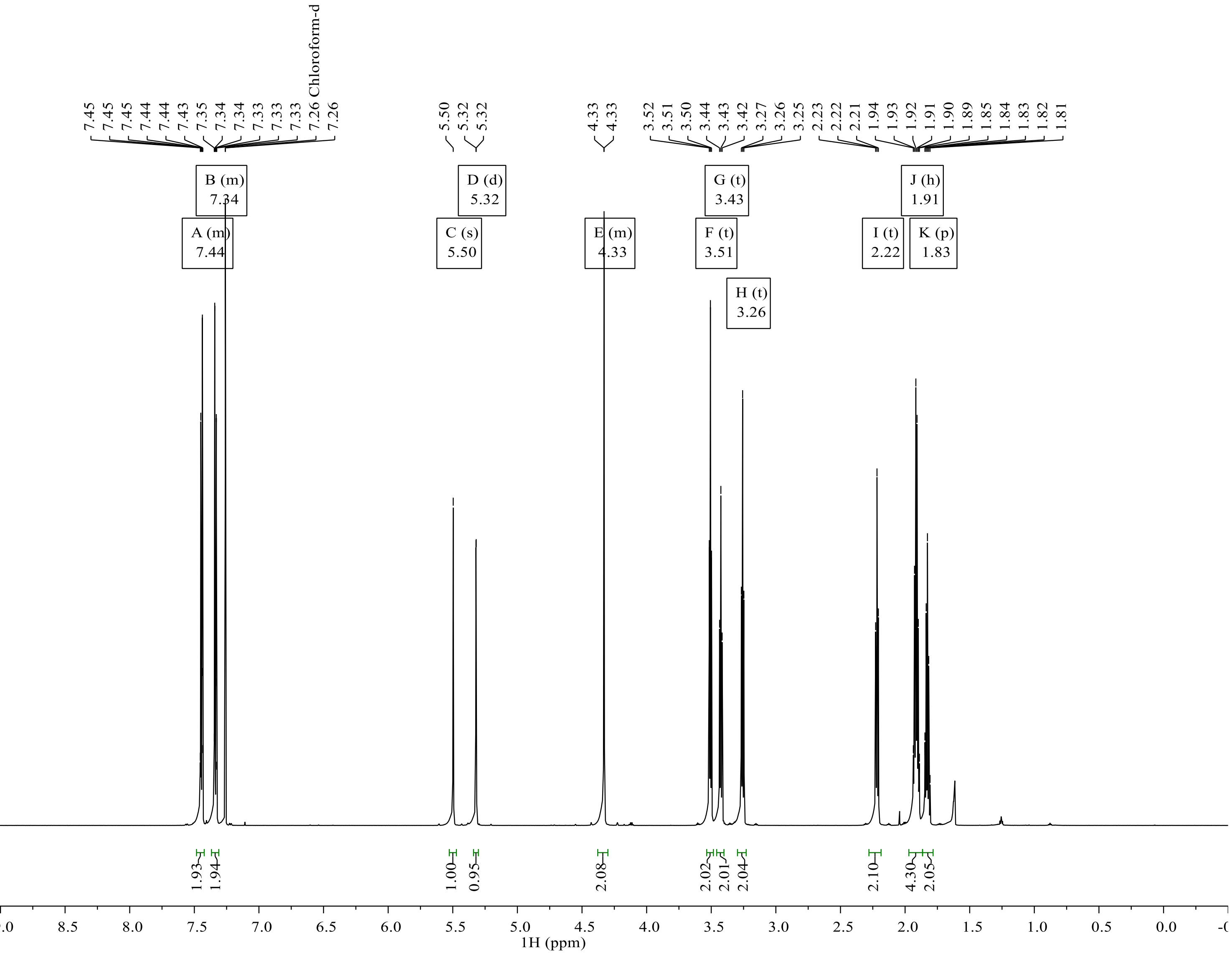

(^{13}C NMR, $CDCl_3$, 176 MHz)

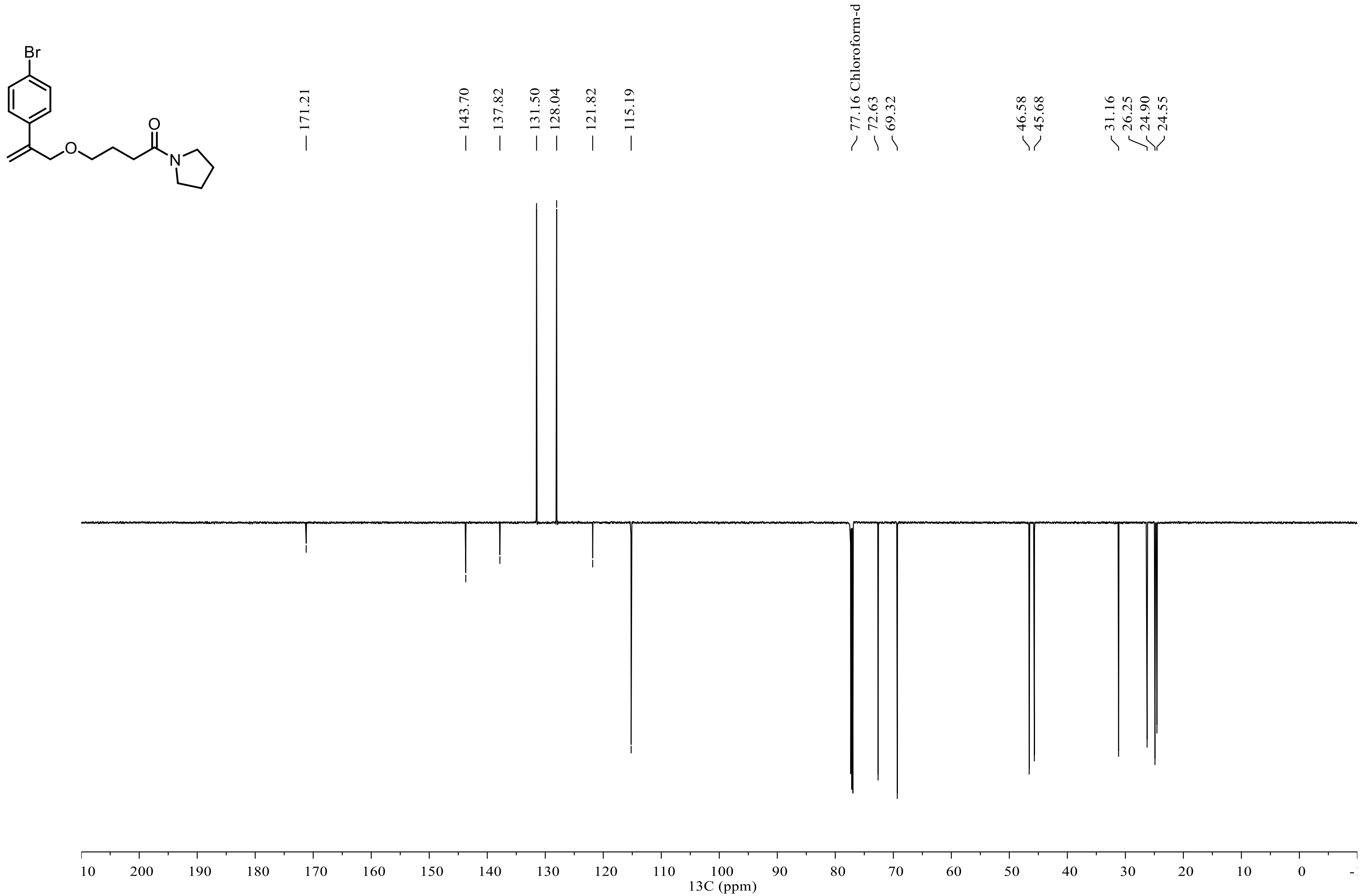

13: (^{1}H NMR, $CDCl_3$, 400 MHz)

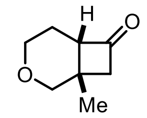

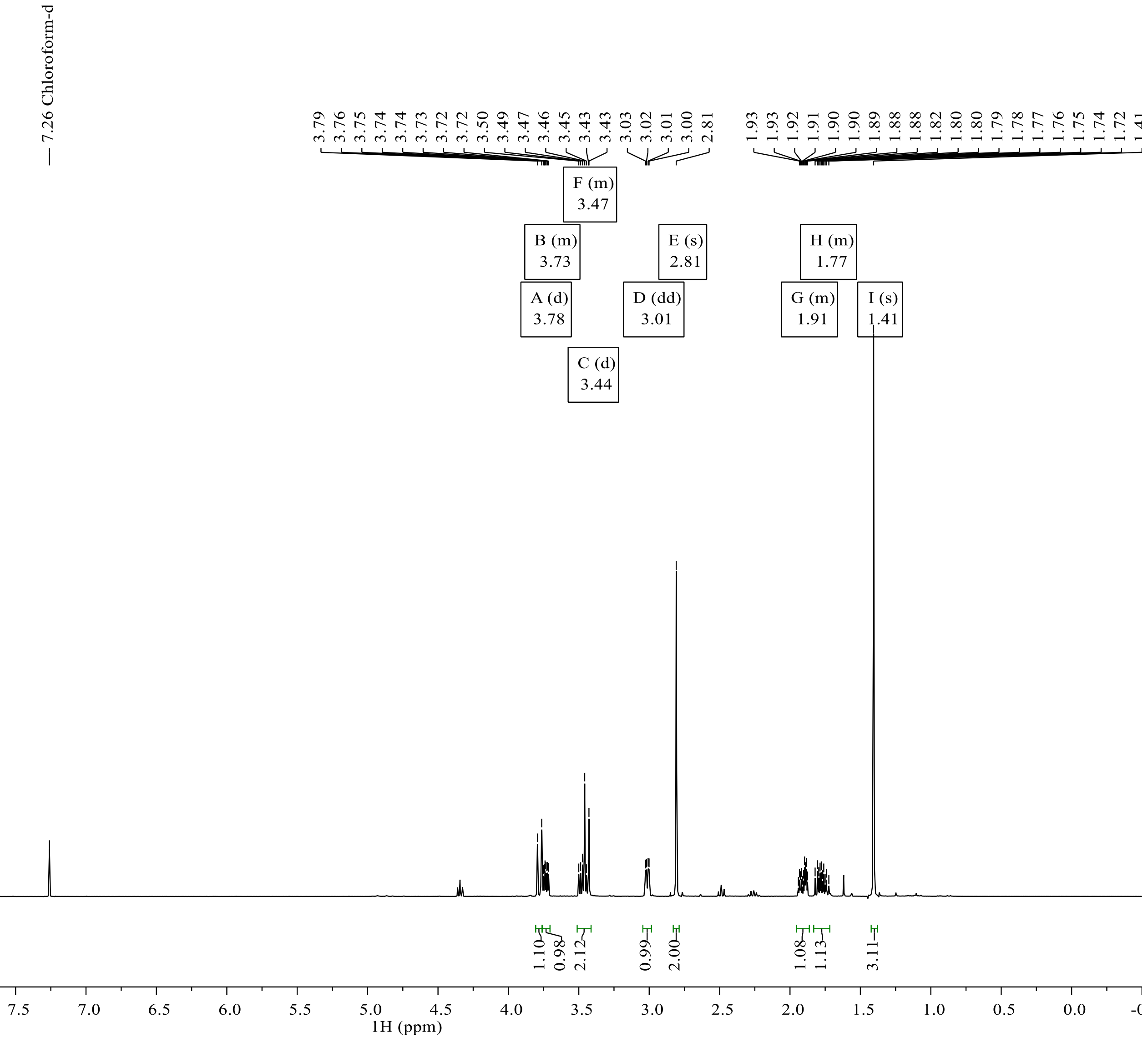

(^{13}C NMR, $CDCl_3$, 101 MHz)

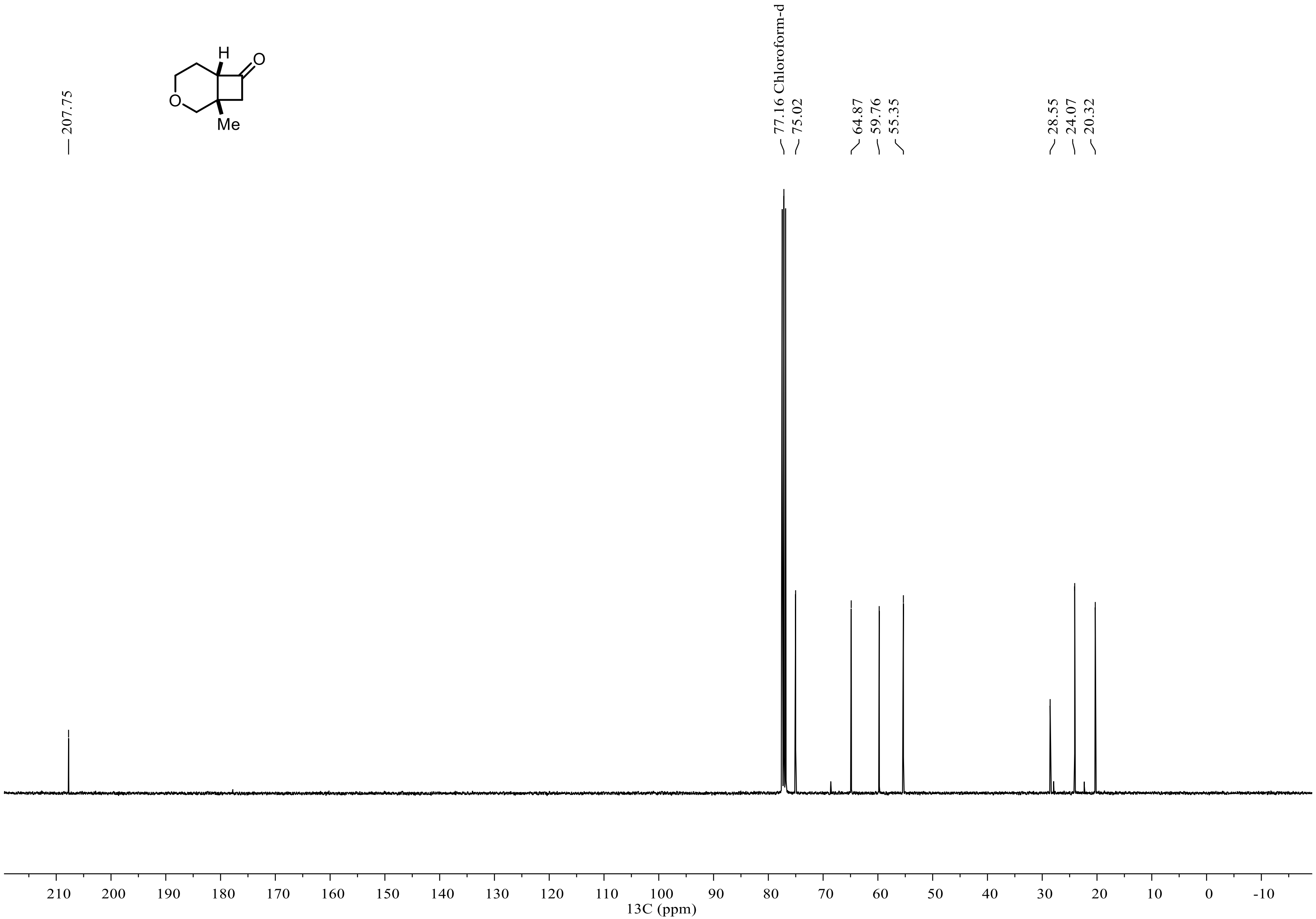

14: (^{1}H NMR, $CDCl_3$, 400 MHz)

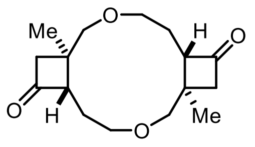

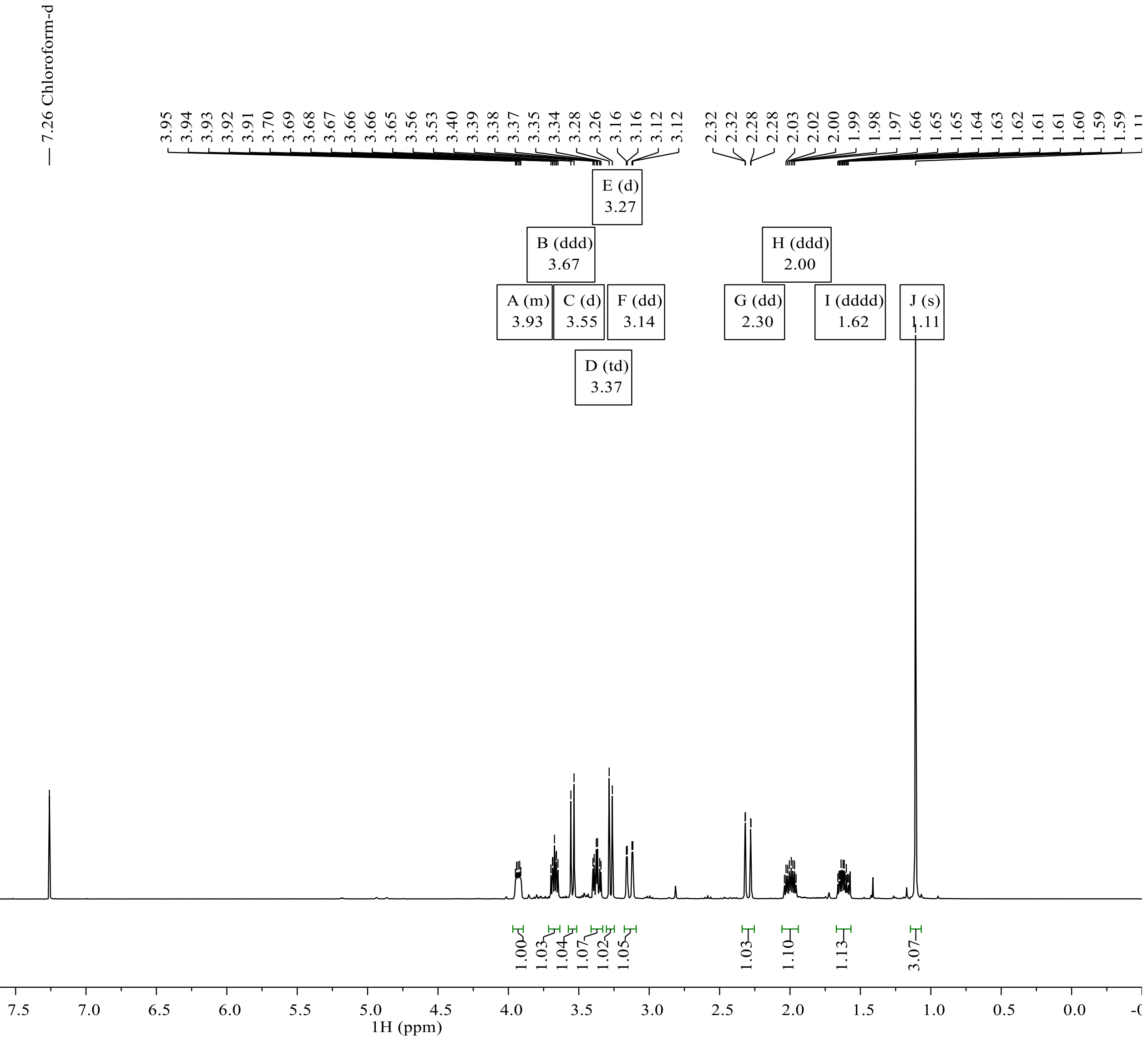

(^{13}C NMR, $CDCl_3$, 101 MHz)

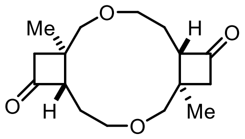

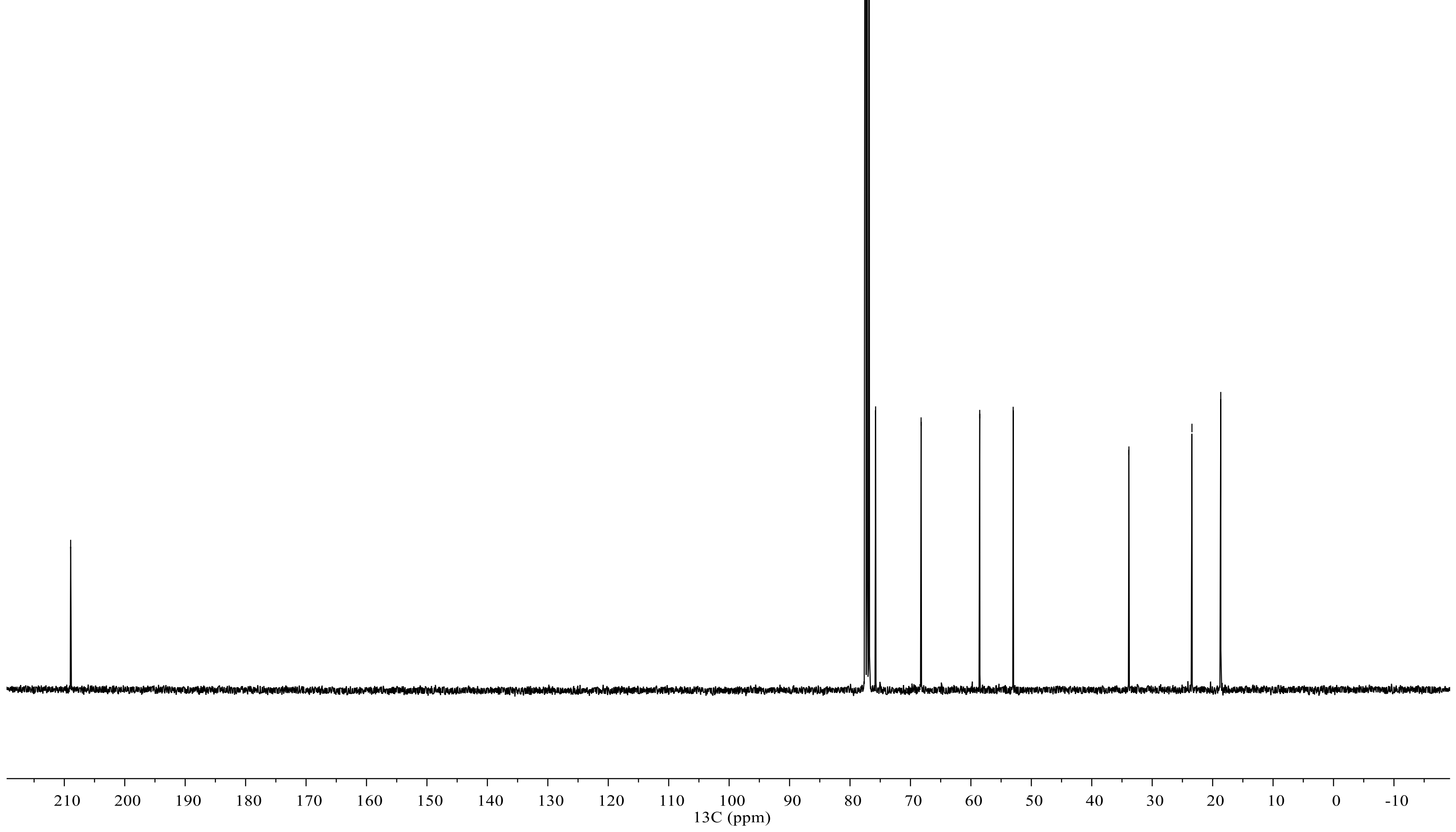

31a: (^{1}H NMR, $CDCl_3$, 600 MHz)

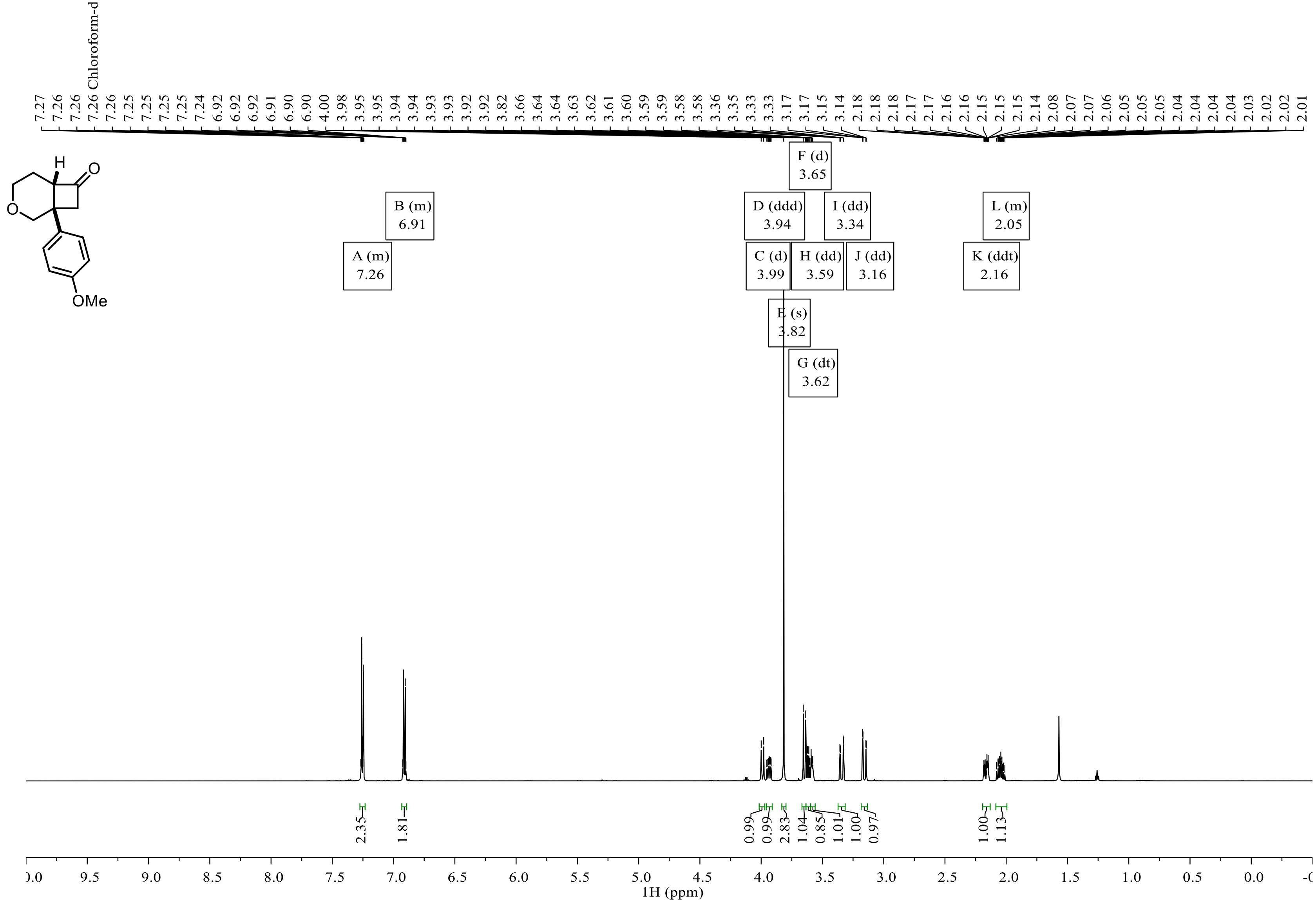

(^{13}C NMR, $CDCl_3$, 151 MHz)

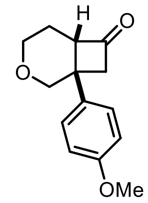

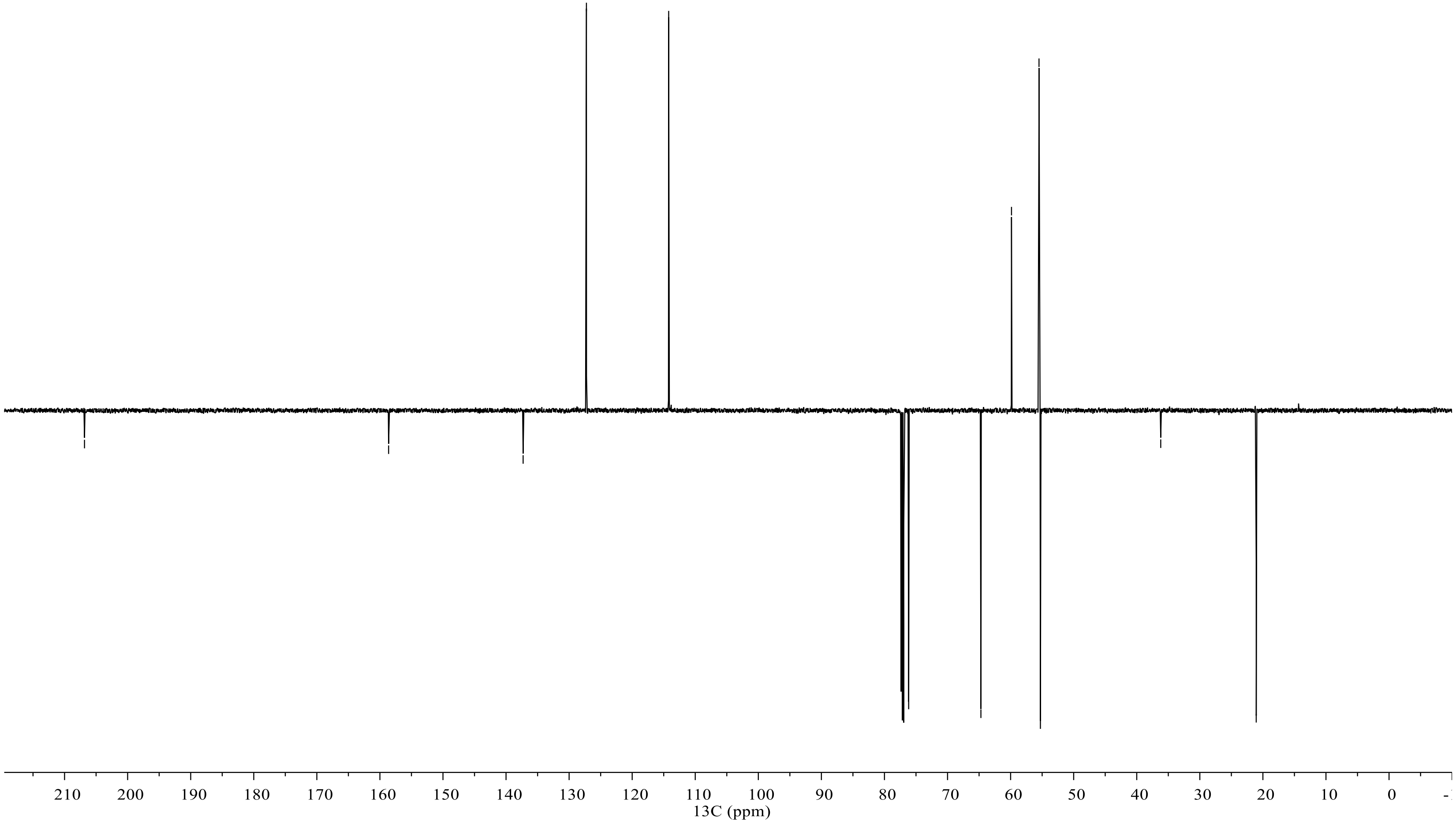

(^1H-^1H NOESY, $CDCl_3$, 600 MHz)

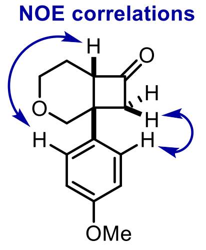

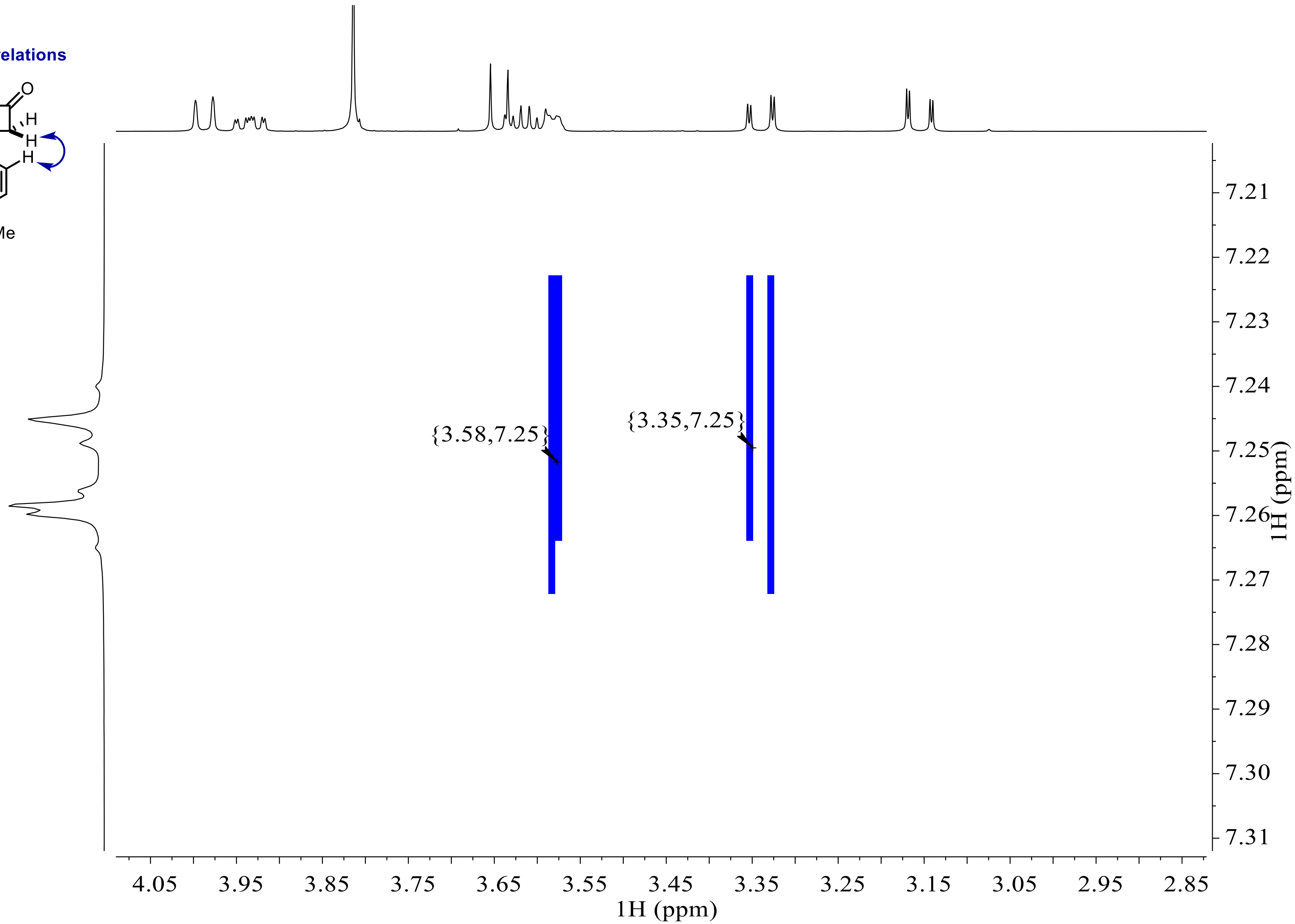

31b: (^{1}H NMR, $CDCl_3$, 400 MHz)

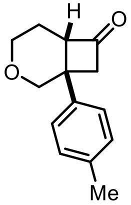

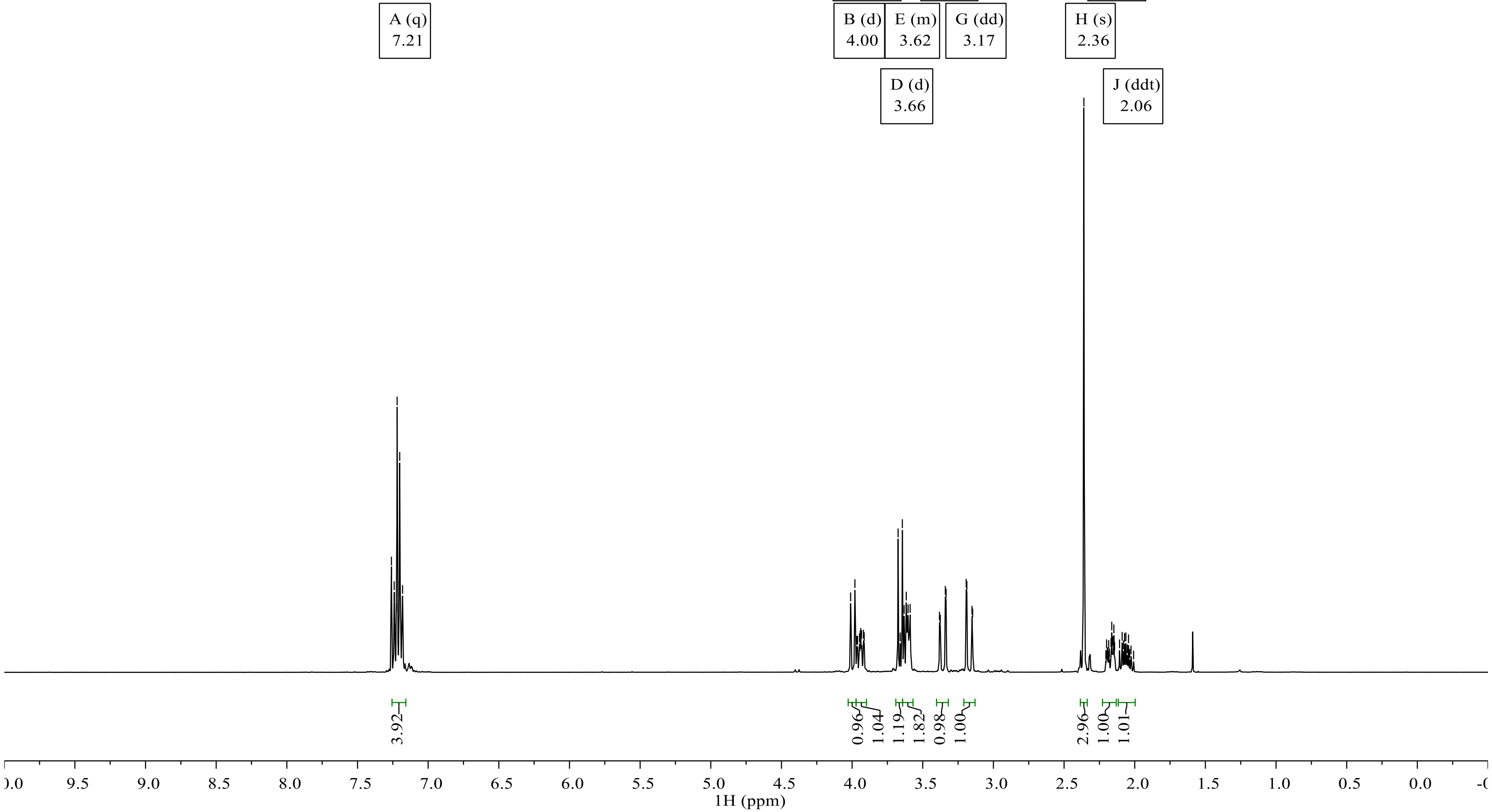

(^{13}C NMR, $CDCl_3$, 101 MHz)

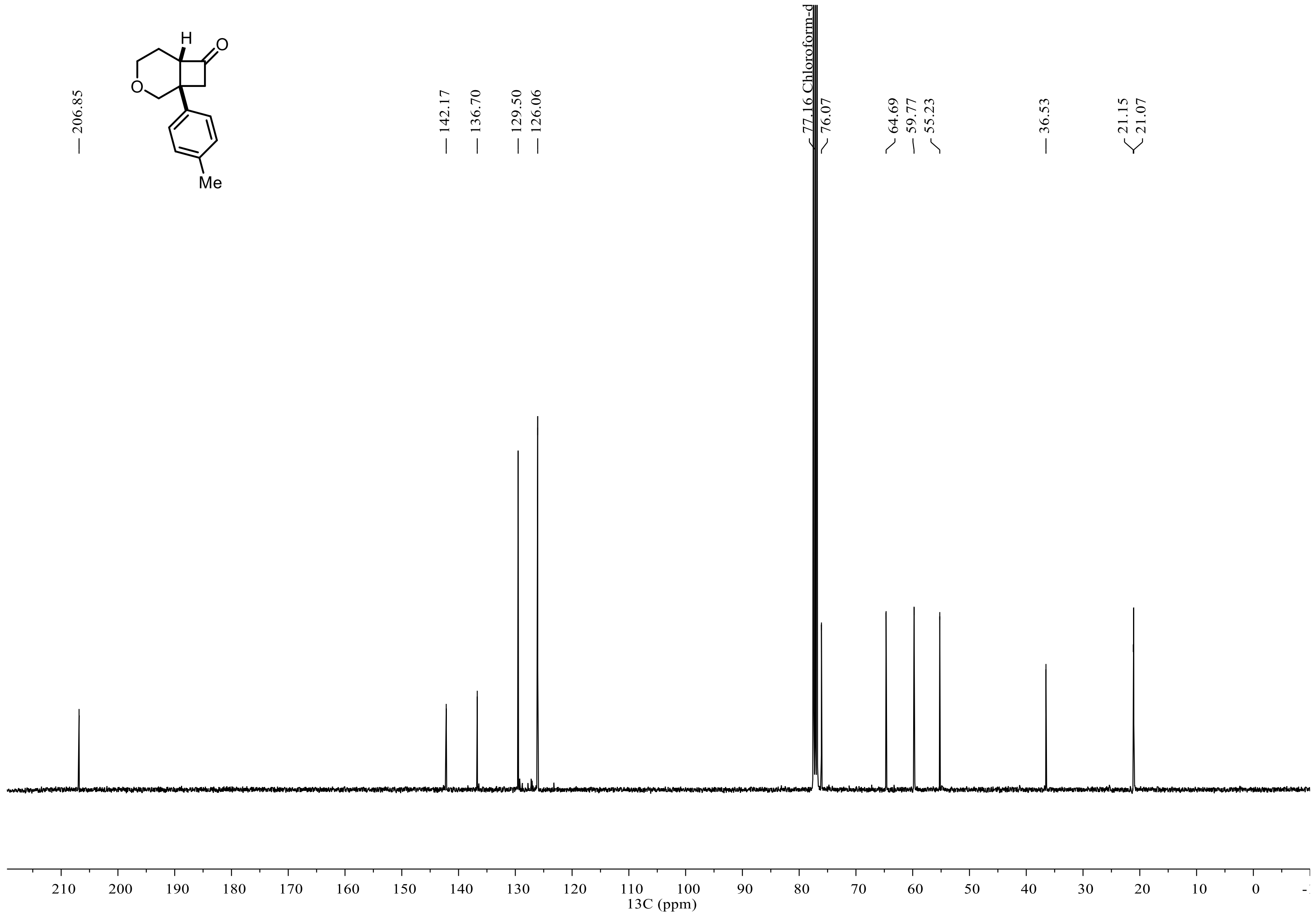

32b: (^{1}H NMR, $CDCl_3$, 400 MHz)

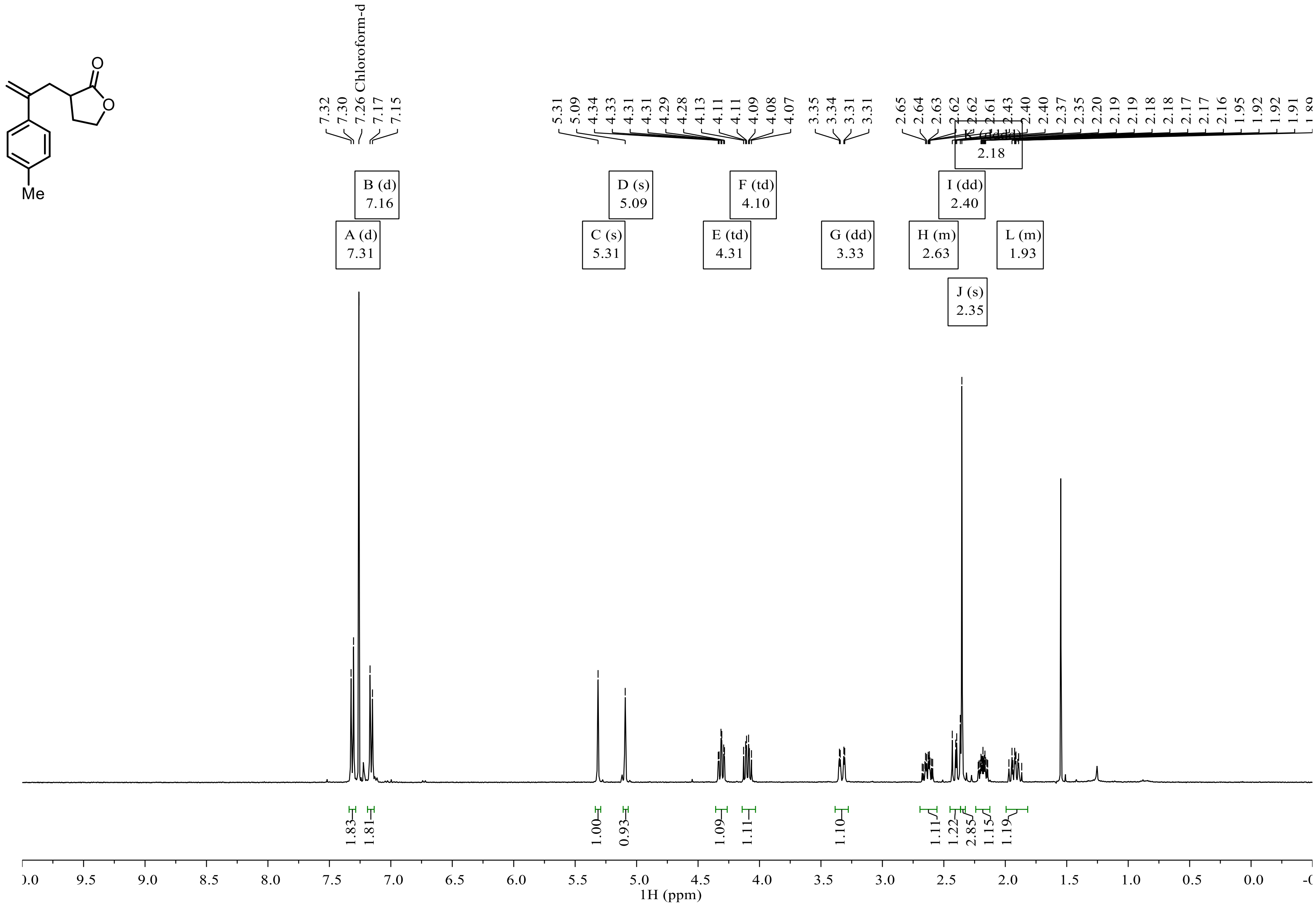

(^{13}C NMR, $CDCl_3$, 101 MHz)

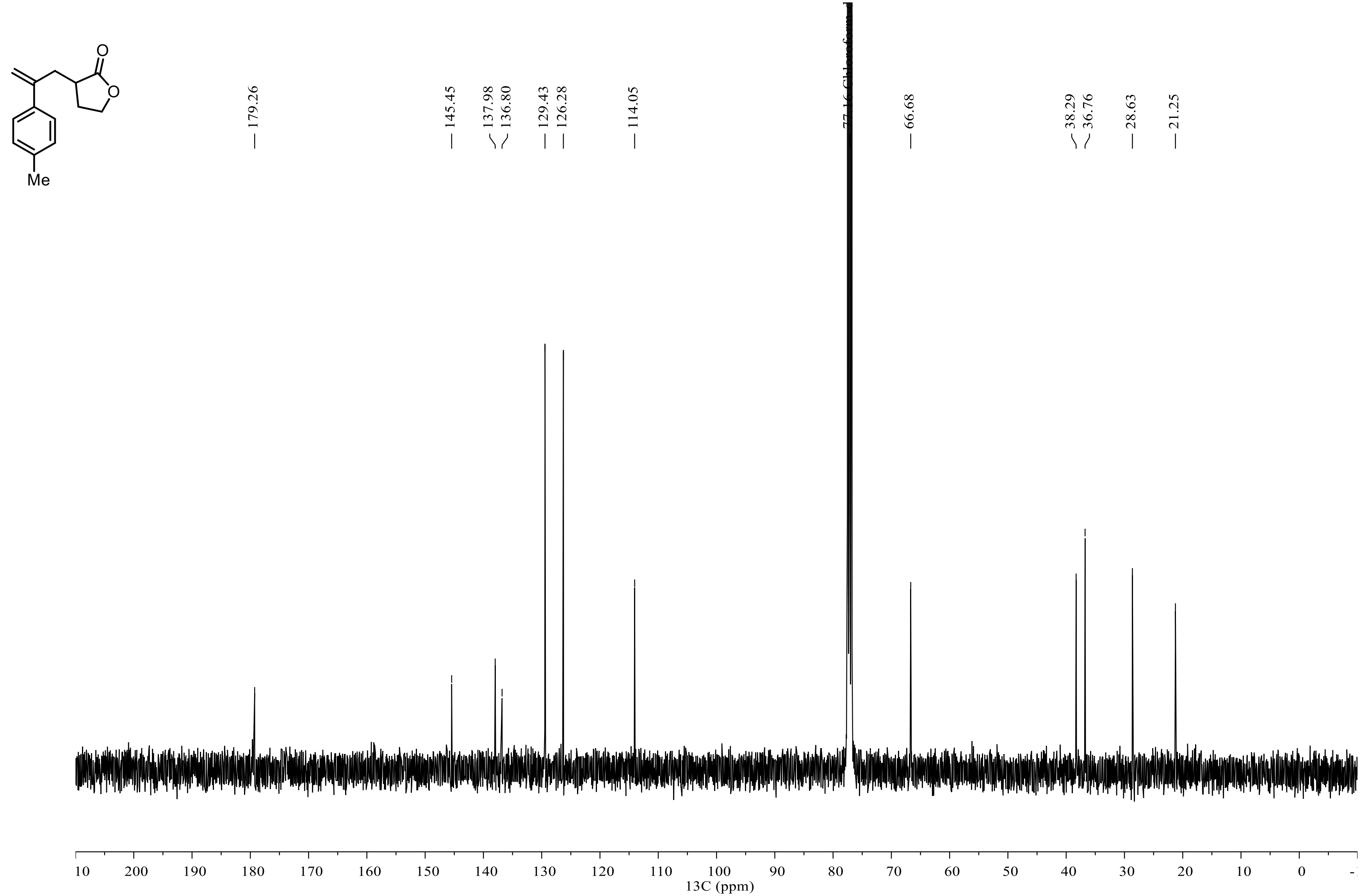

31c: (^{1}H NMR, $CDCl_3$, 700 MHz)

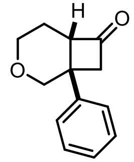

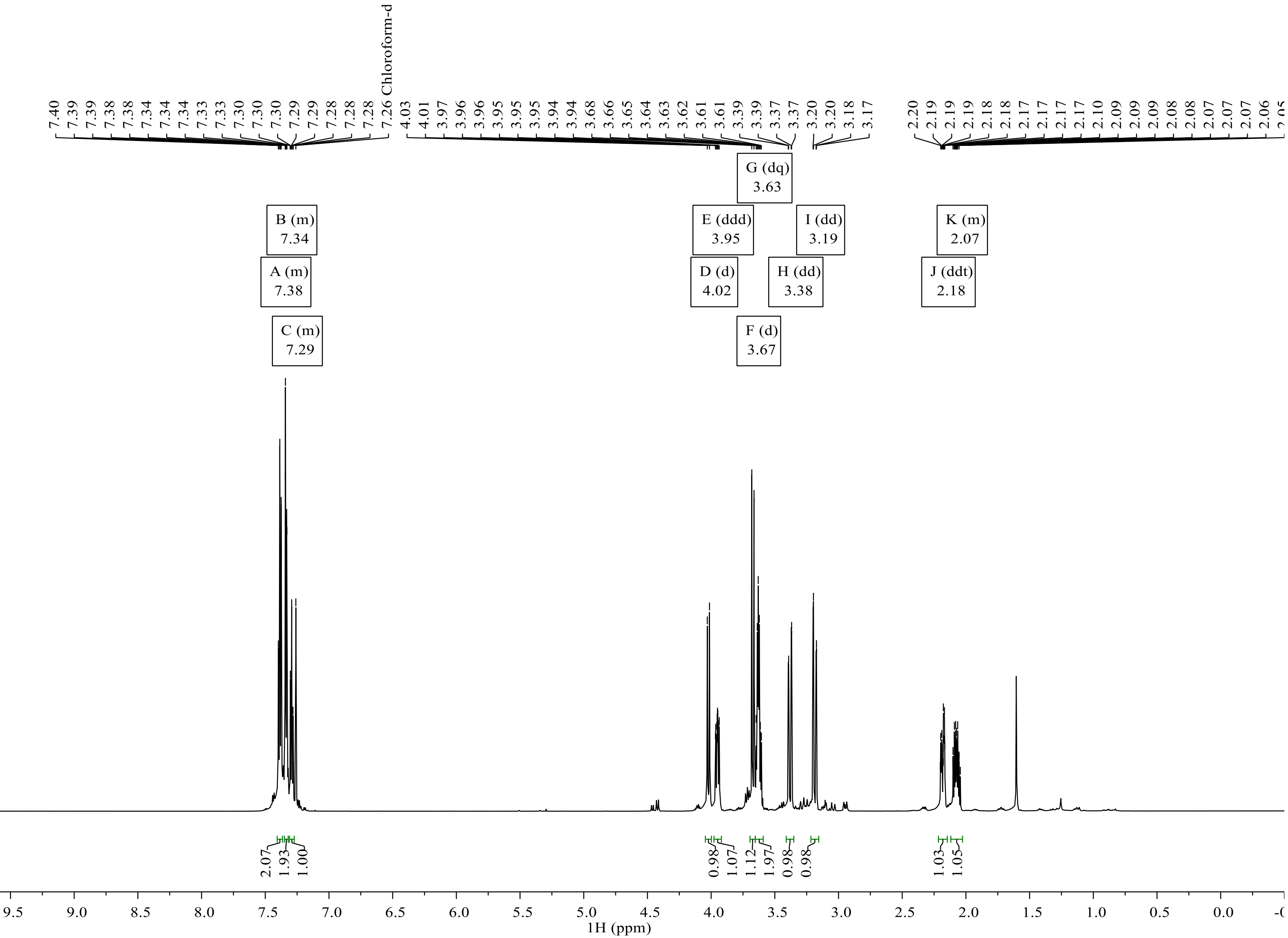

(^{13}C NMR, $CDCl_3$, 176 MHz)

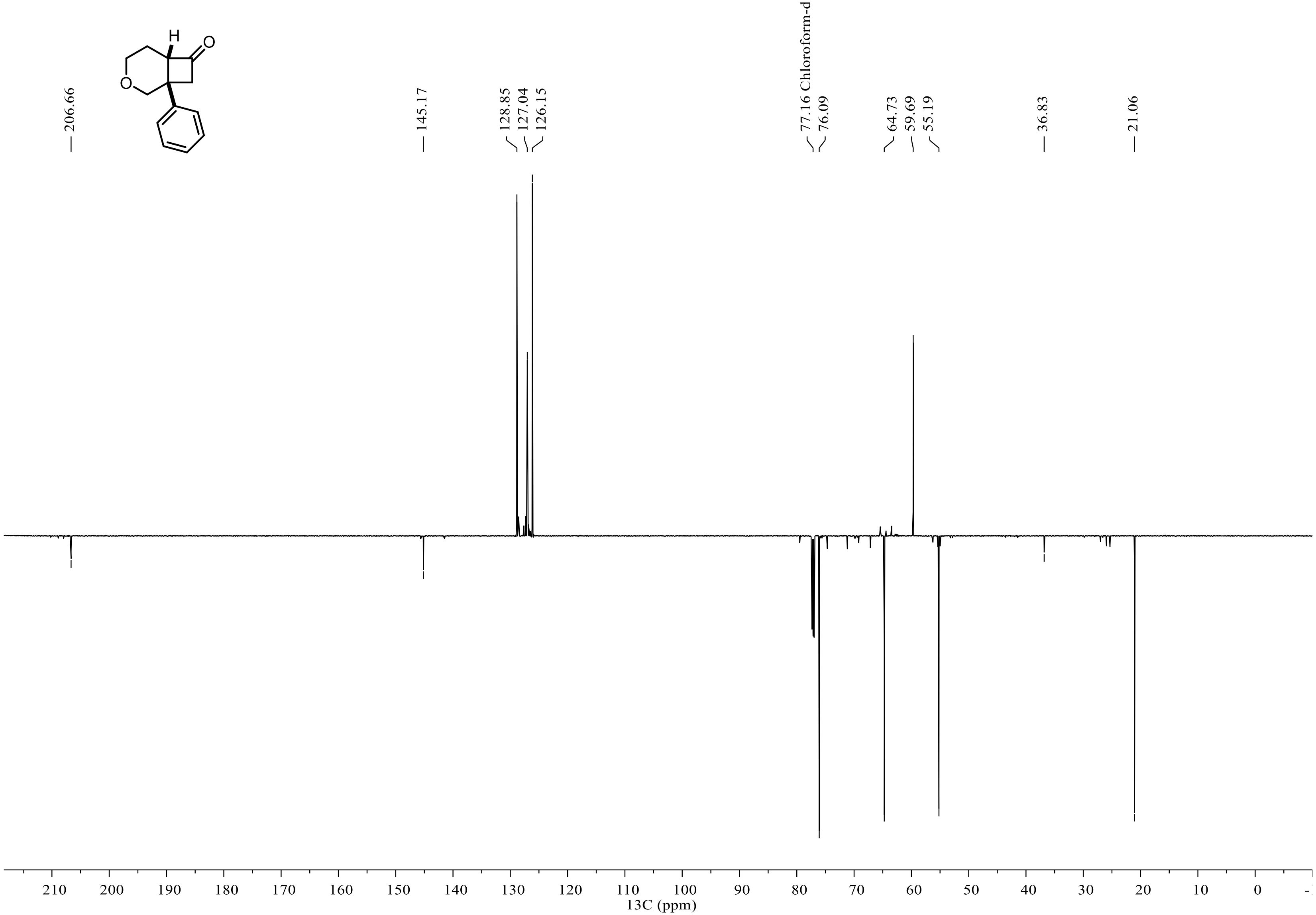

32c: (^{1}H NMR, $CDCl_3$, 400 MHz)

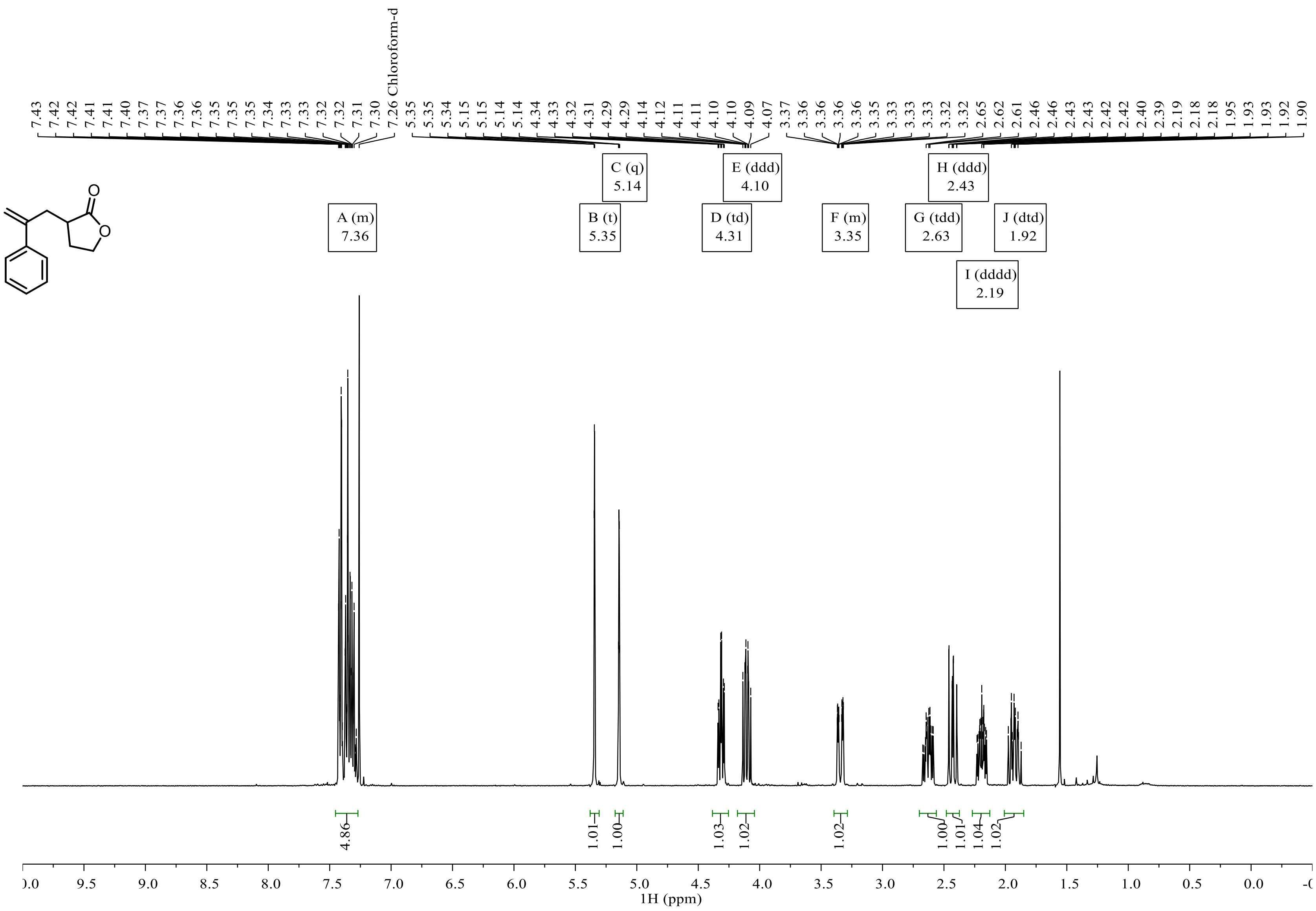

(^{13}C NMR, $CDCl_3$, 101 MHz)

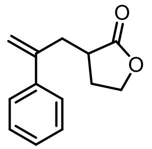

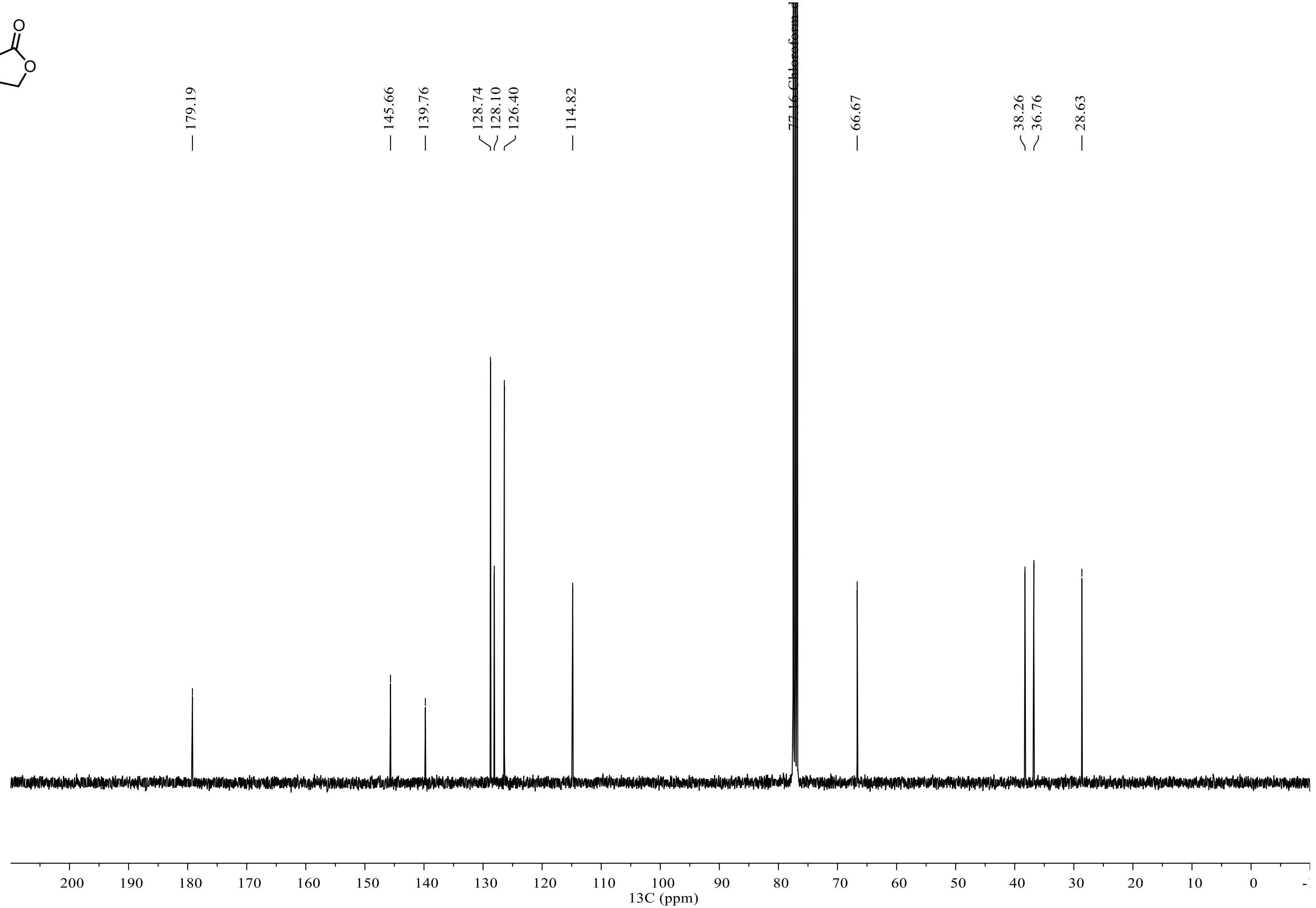

31d: (^{1}H NMR, $CDCl_3$, 700 MHz)

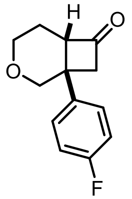

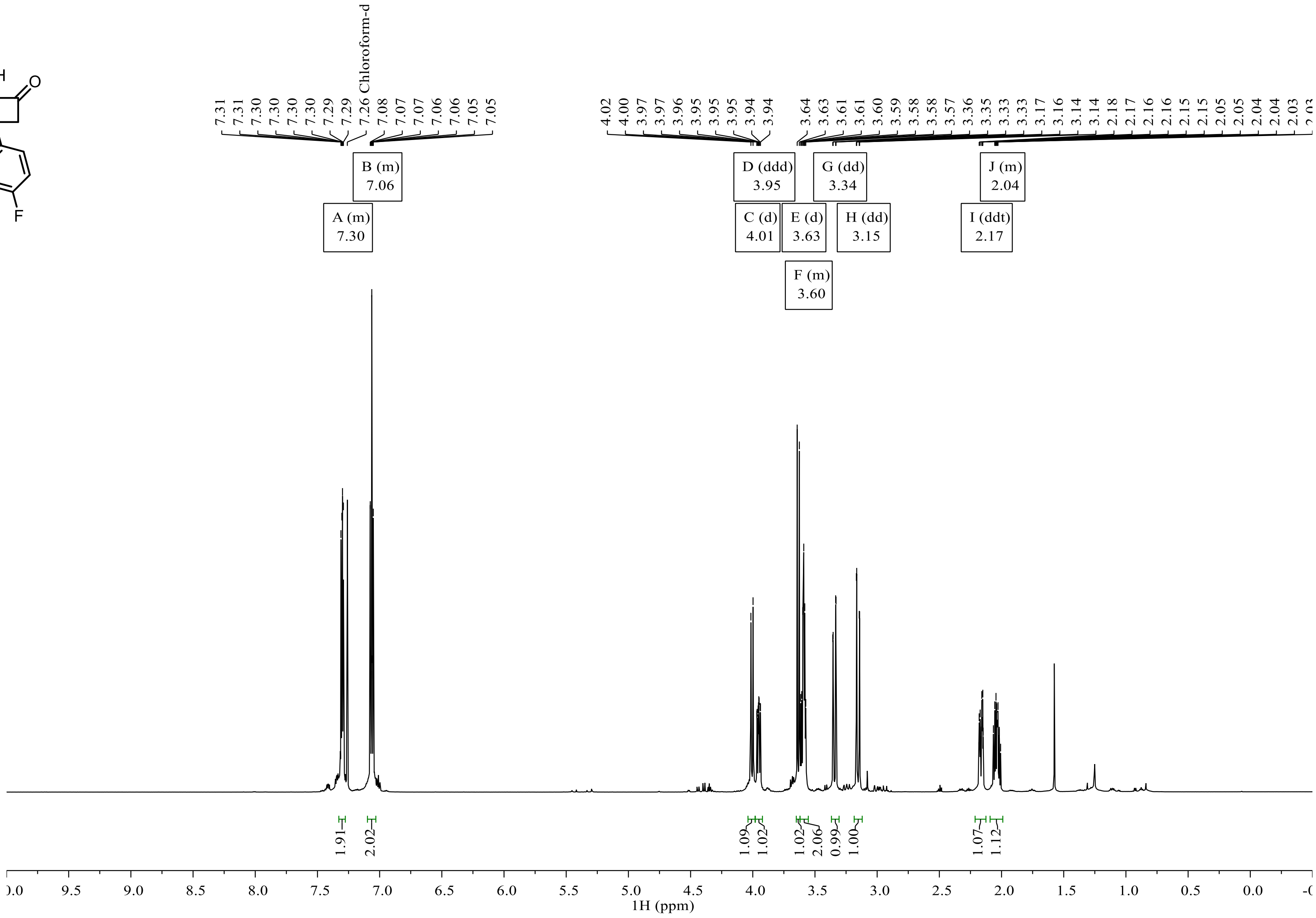

(^{13}C NMR, $CDCl_3$, 176 MHz)

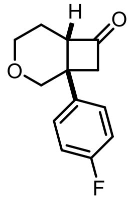

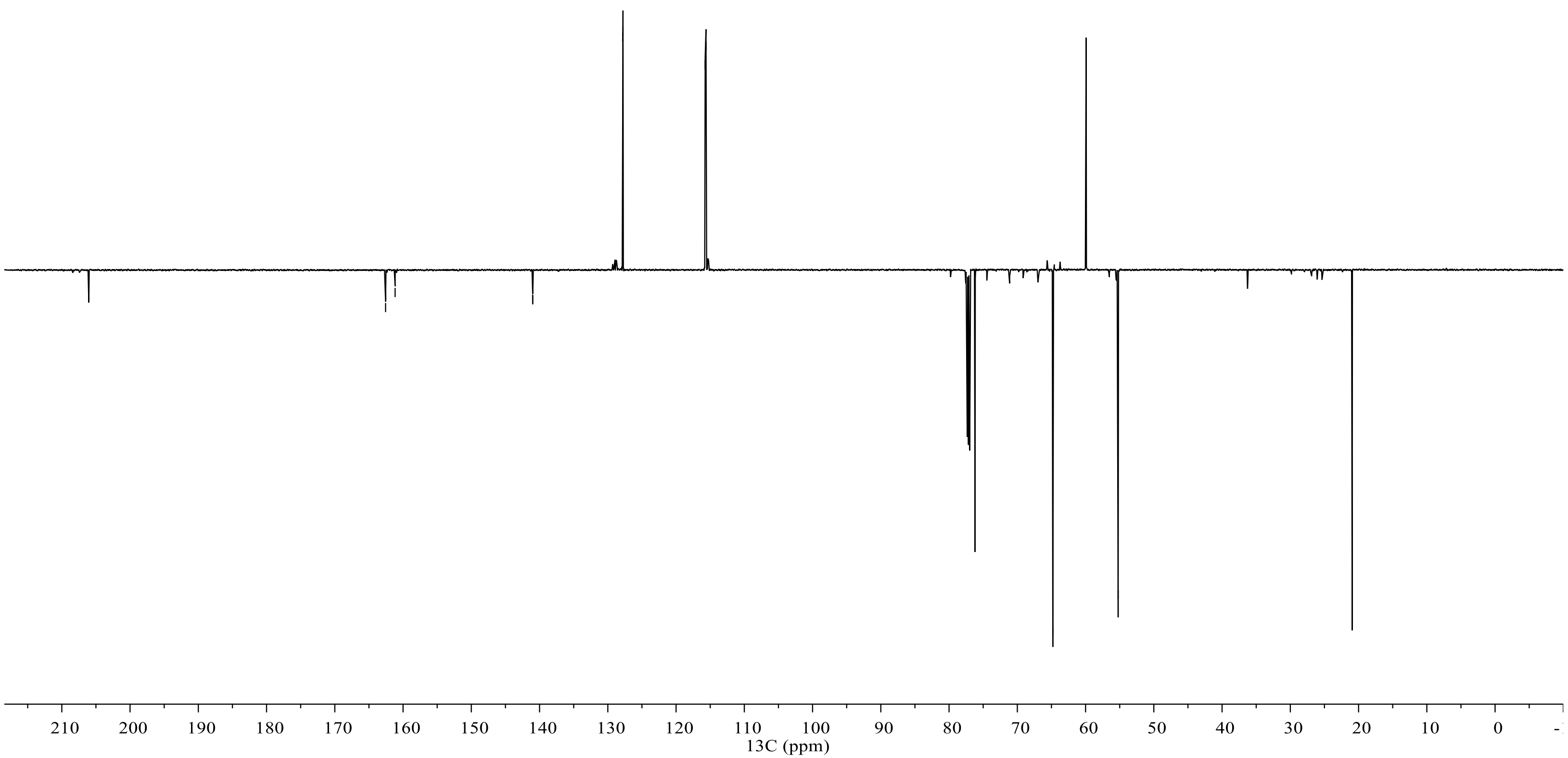

(^{19}F NMR, $CDCl_3$, 659 MHz)

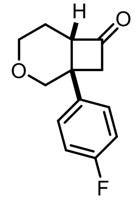

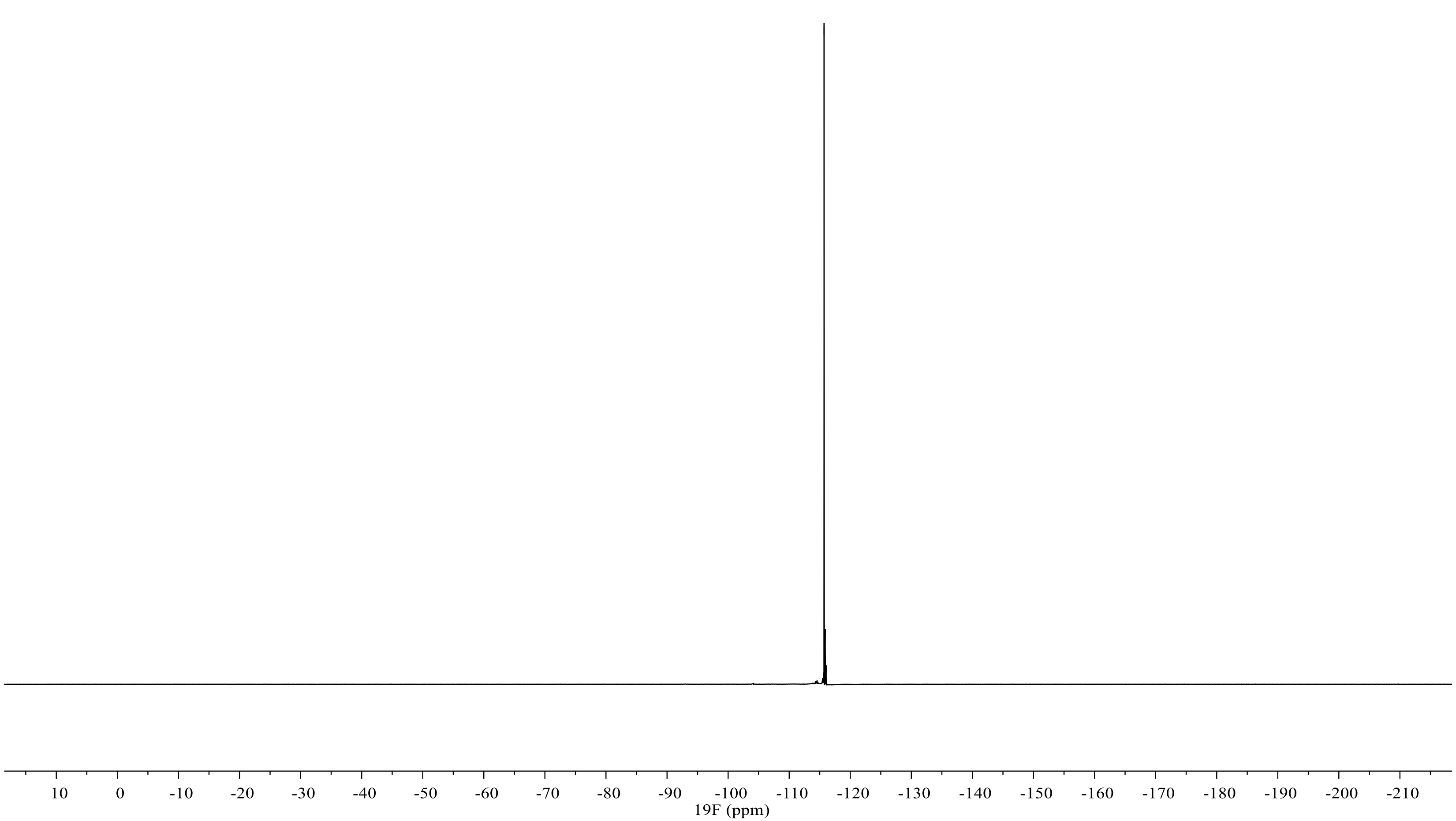

32d: (^{1}H NMR, $CDCl_3$, 700 MHz)

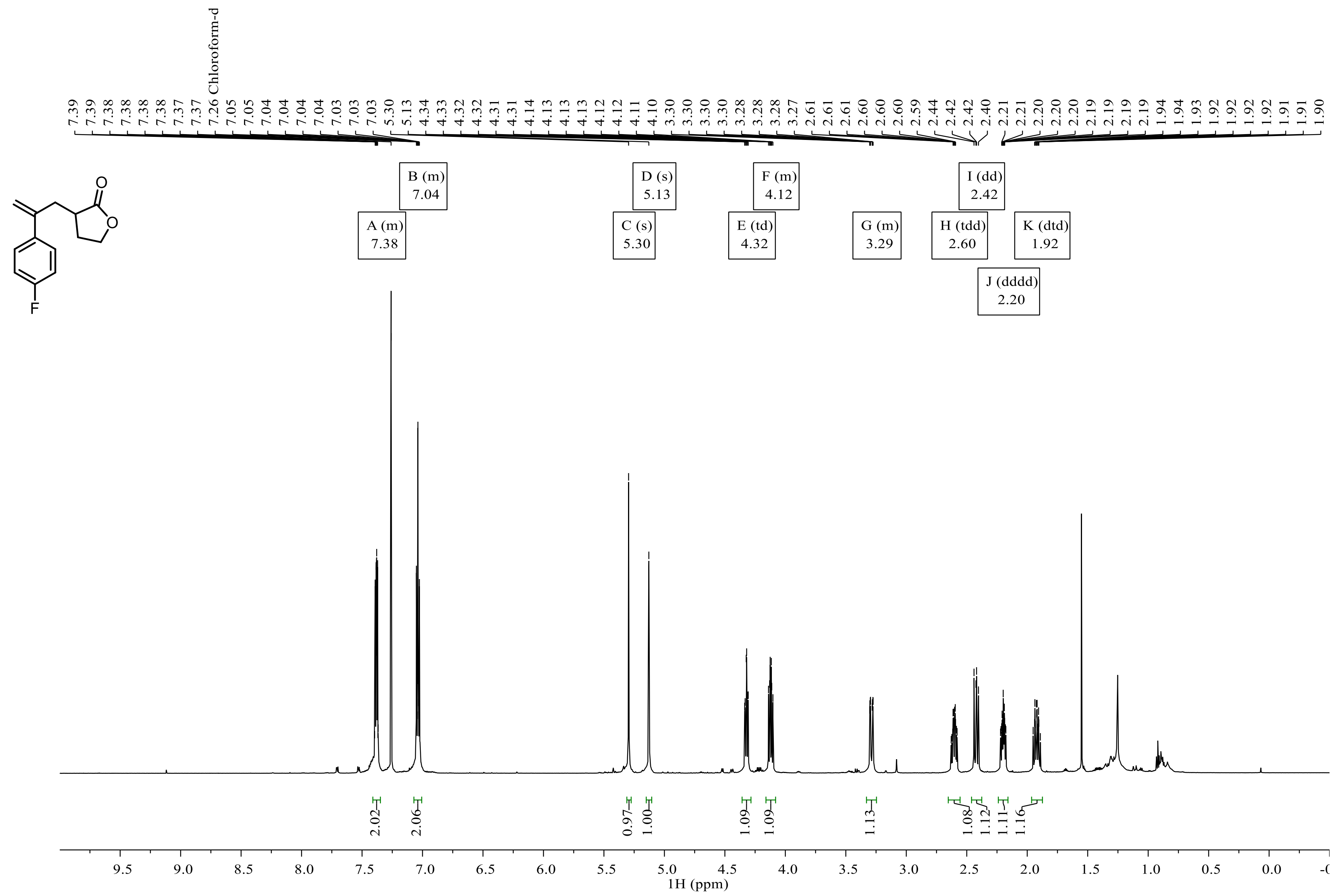

(^{13}C NMR, $CDCl_3$, 176 MHz)

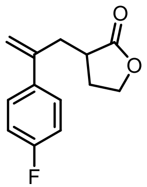

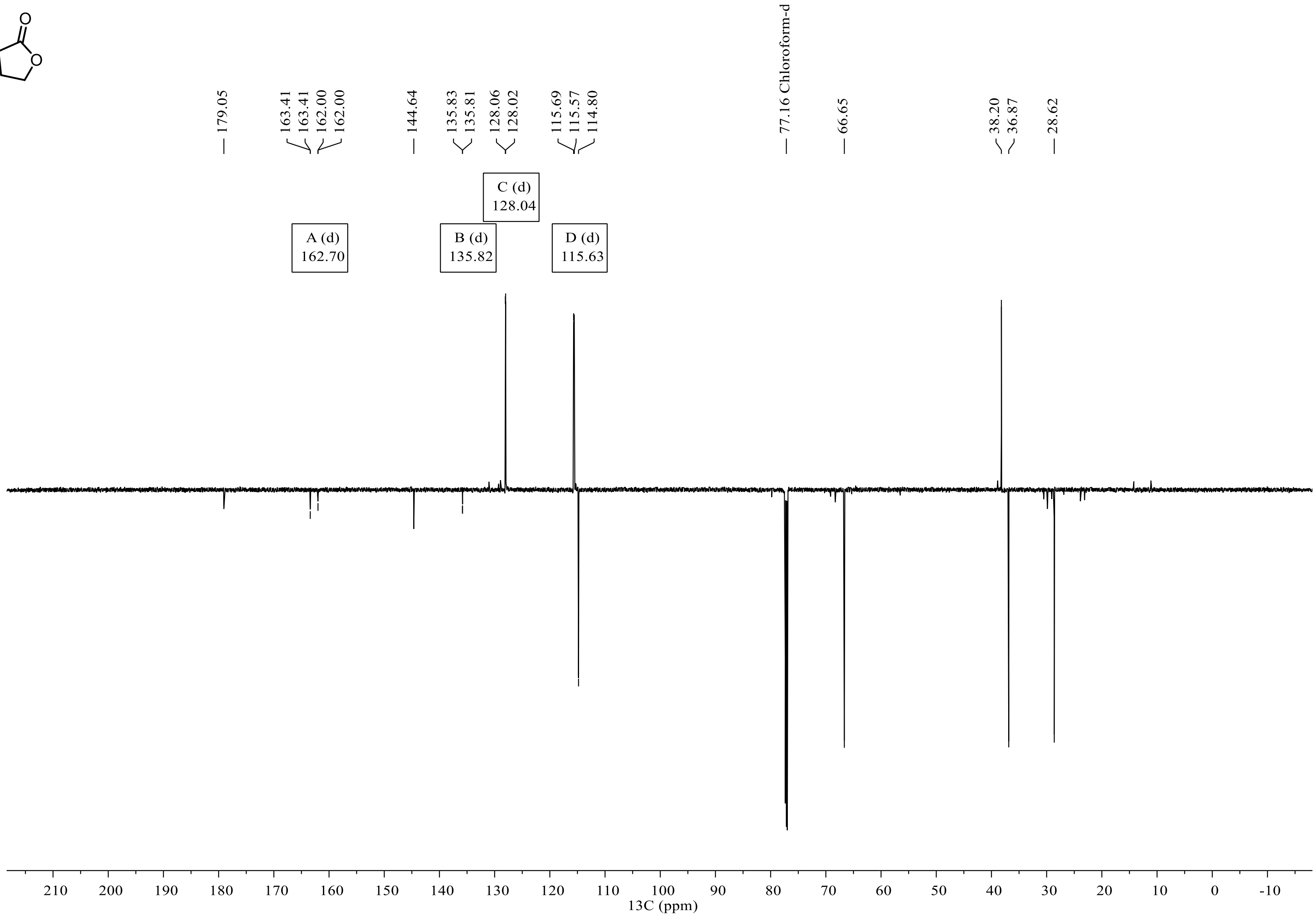

(^{19}F NMR, $CDCl_3$, 659 MHz)

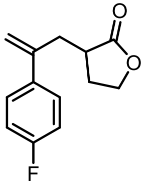

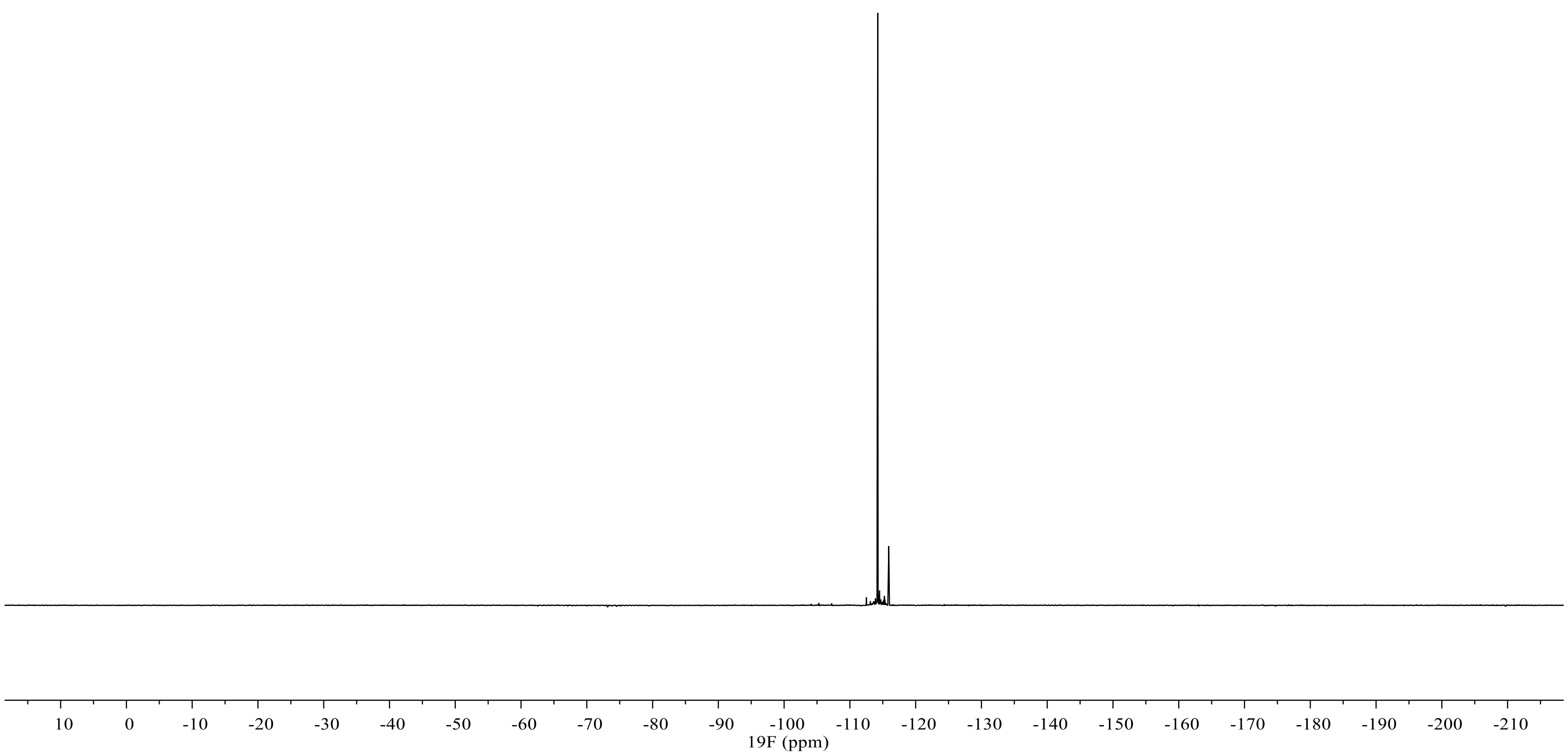

33d: (^{1}H NMR, $CDCl_3$, 600 MHz)

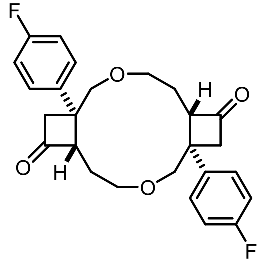

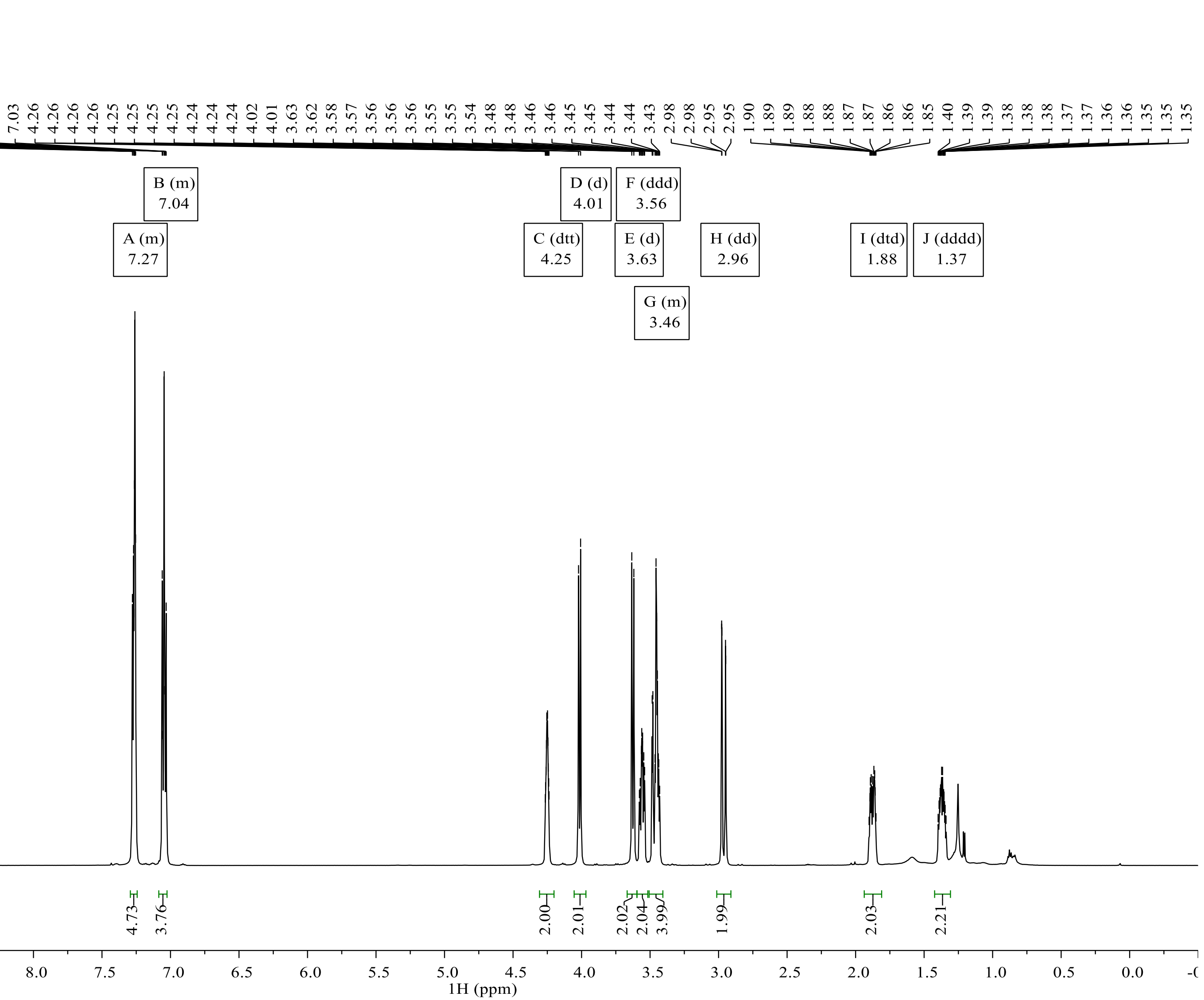

(^{13}C NMR, $CDCl_3$, 151 MHz)

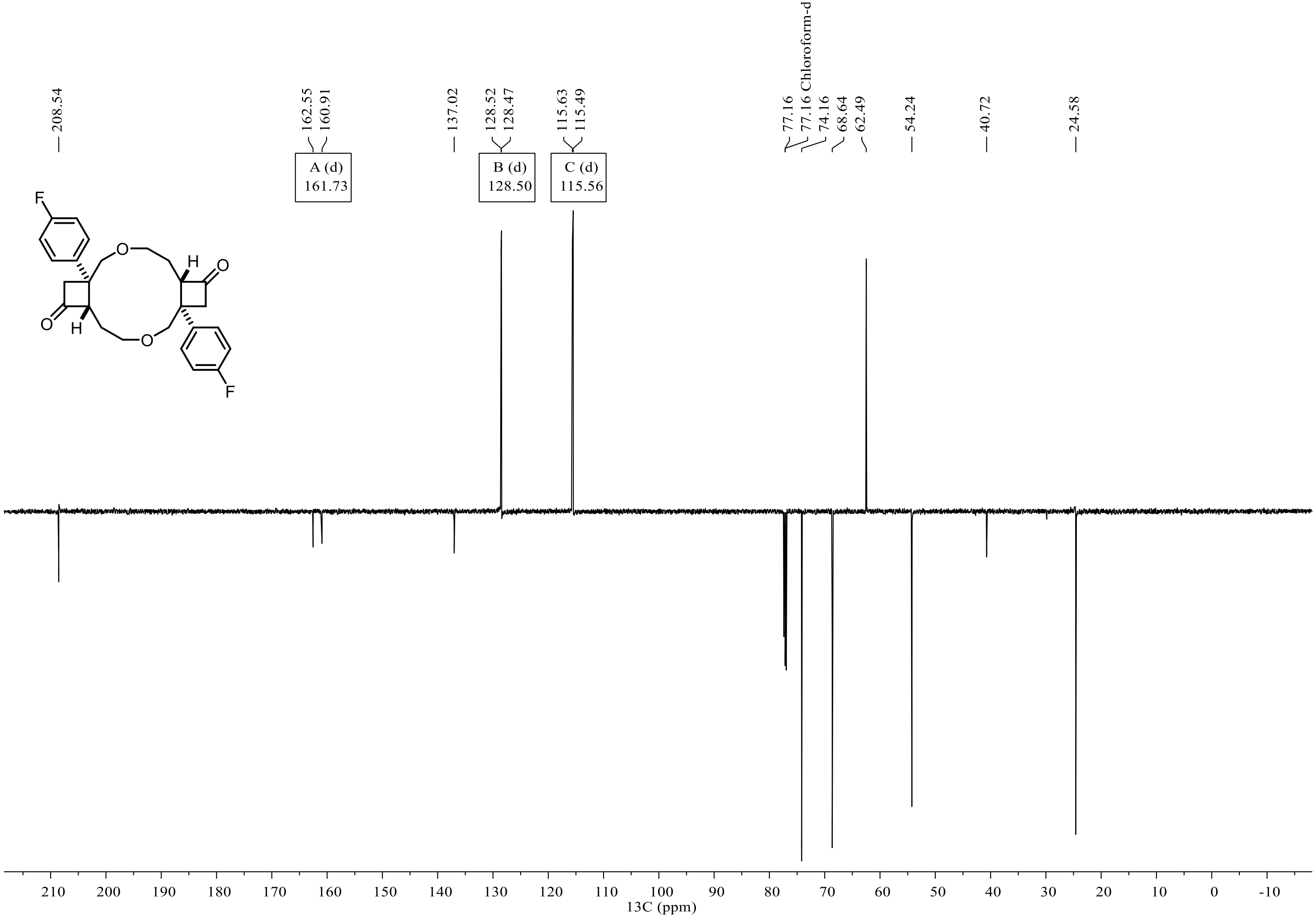

(^{19}F NMR, $CDCl_3$, 565 MHz)

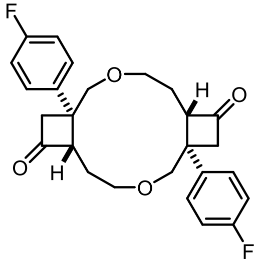

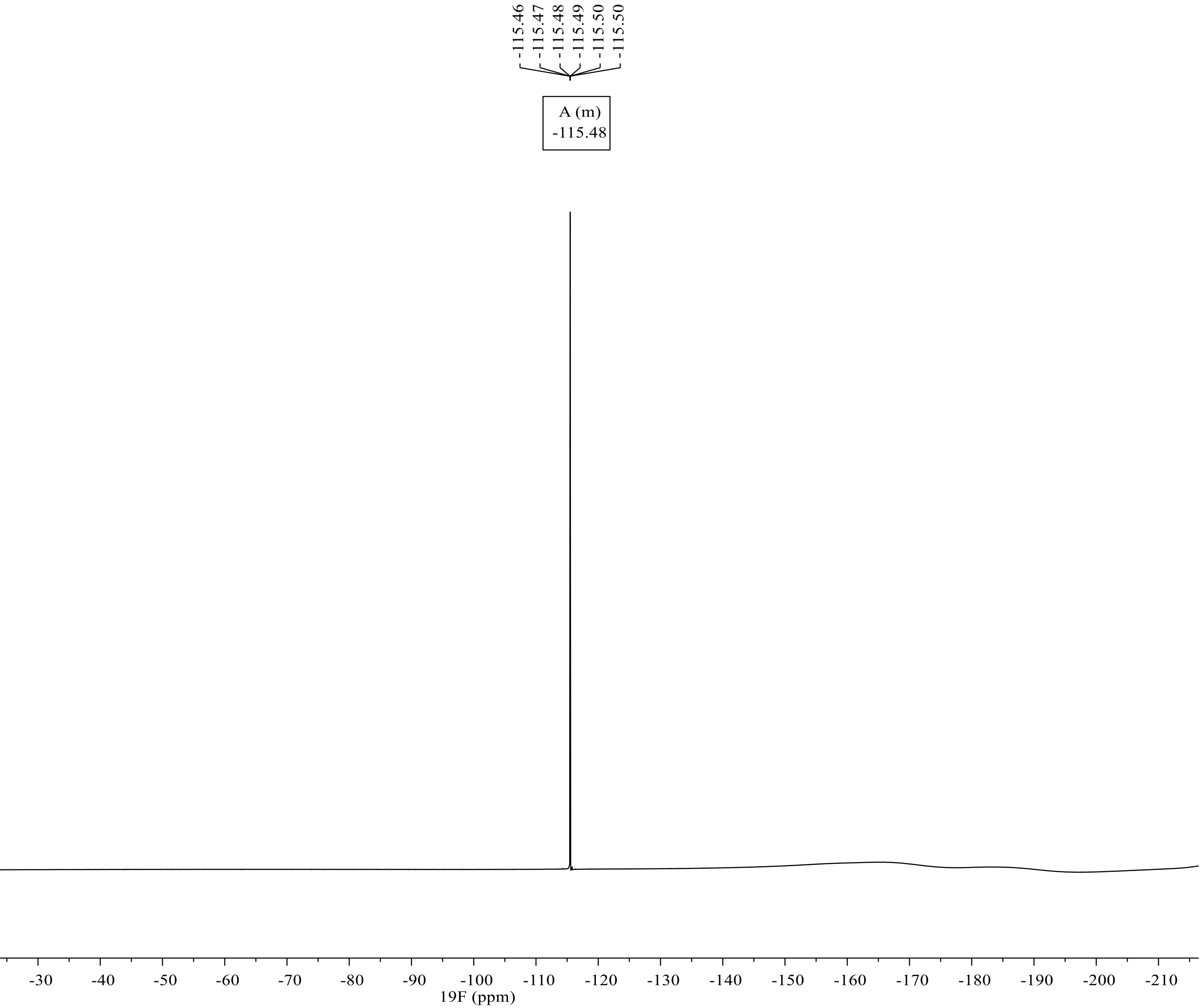

33d': (^{1}H NMR, $CDCl_3$, 700 MHz)

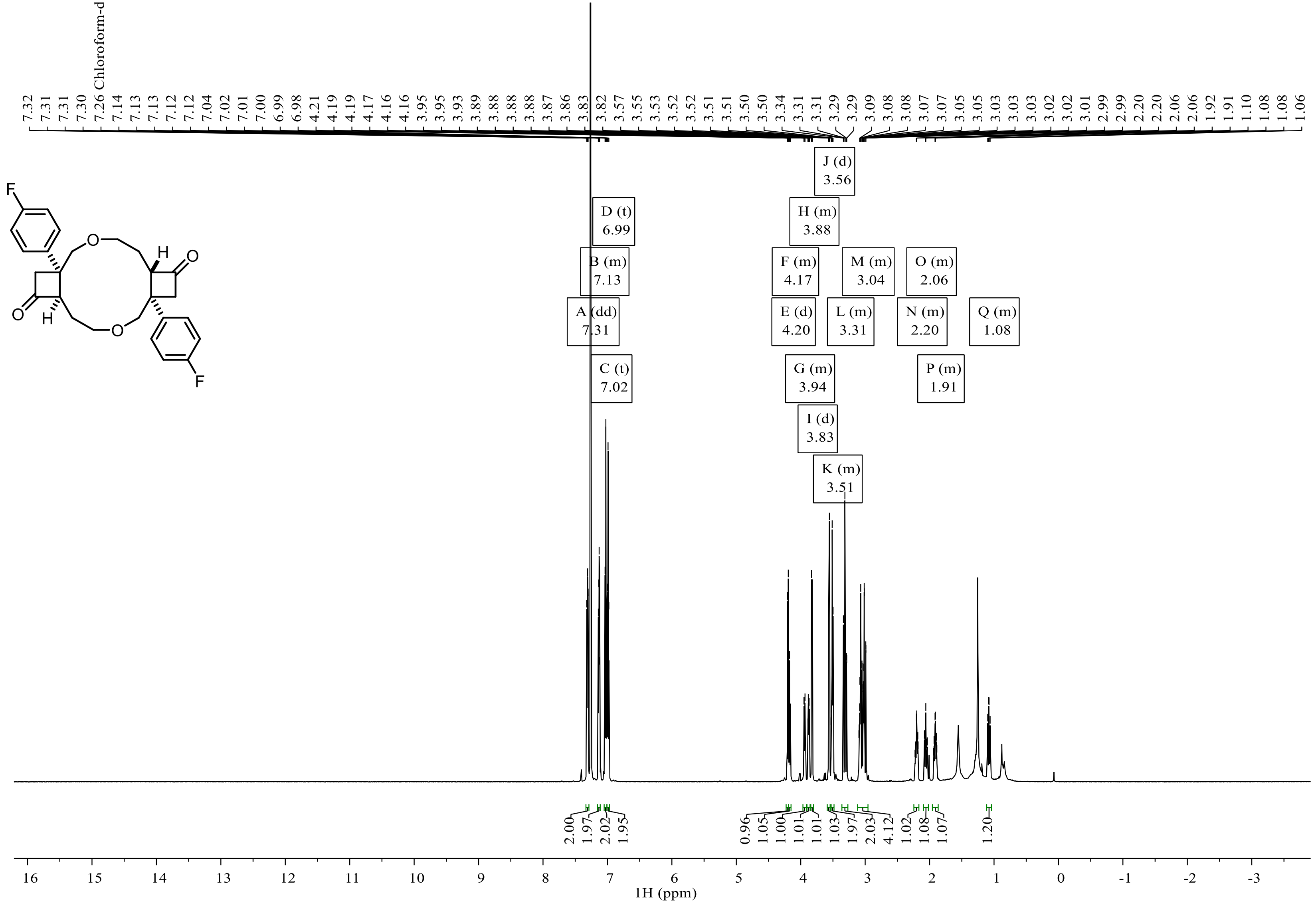

(^{13}C NMR, $CDCl_3$, 176 MHz)

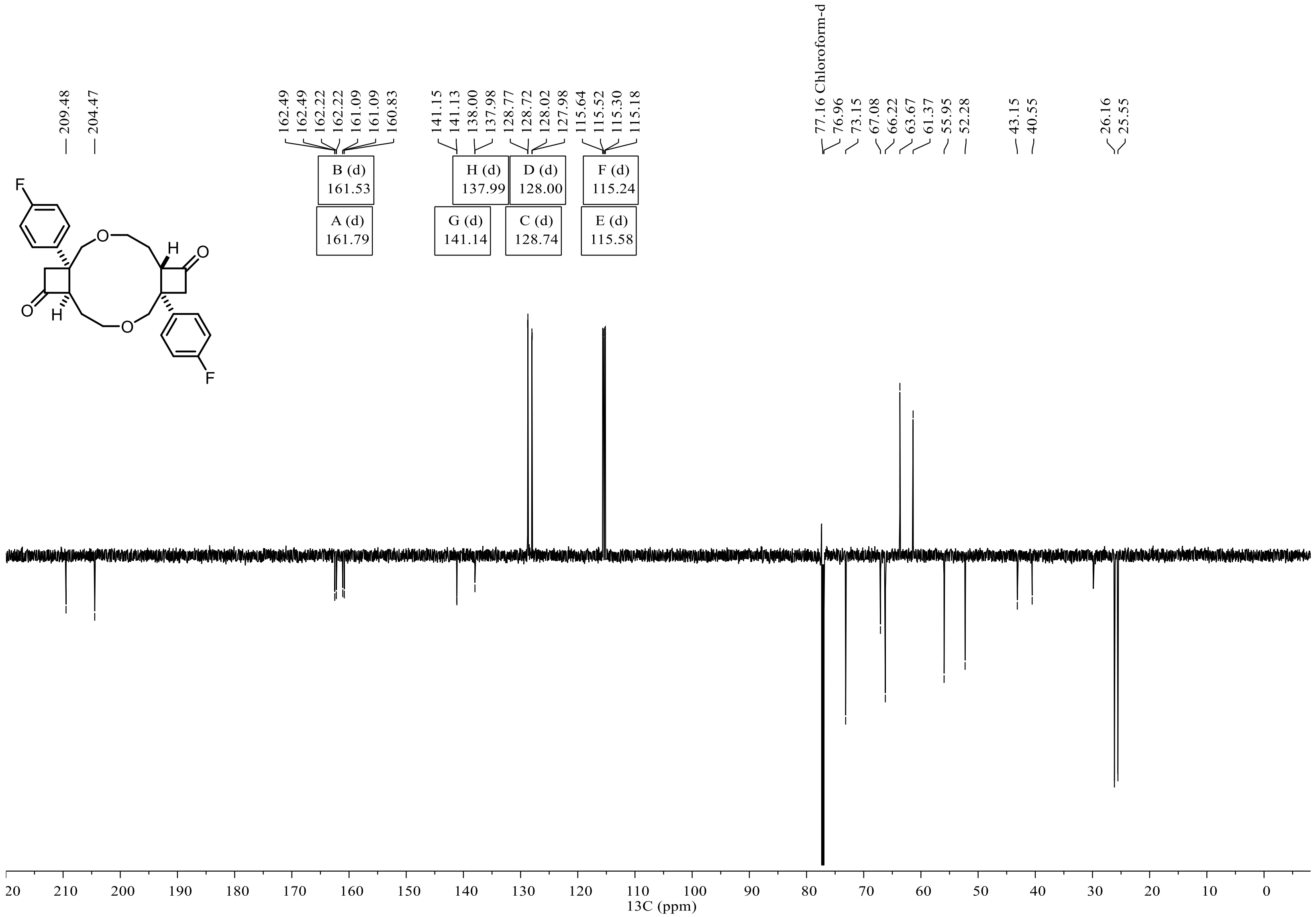

(^19^F NMR, $CDCl_3$, 659 MHz)

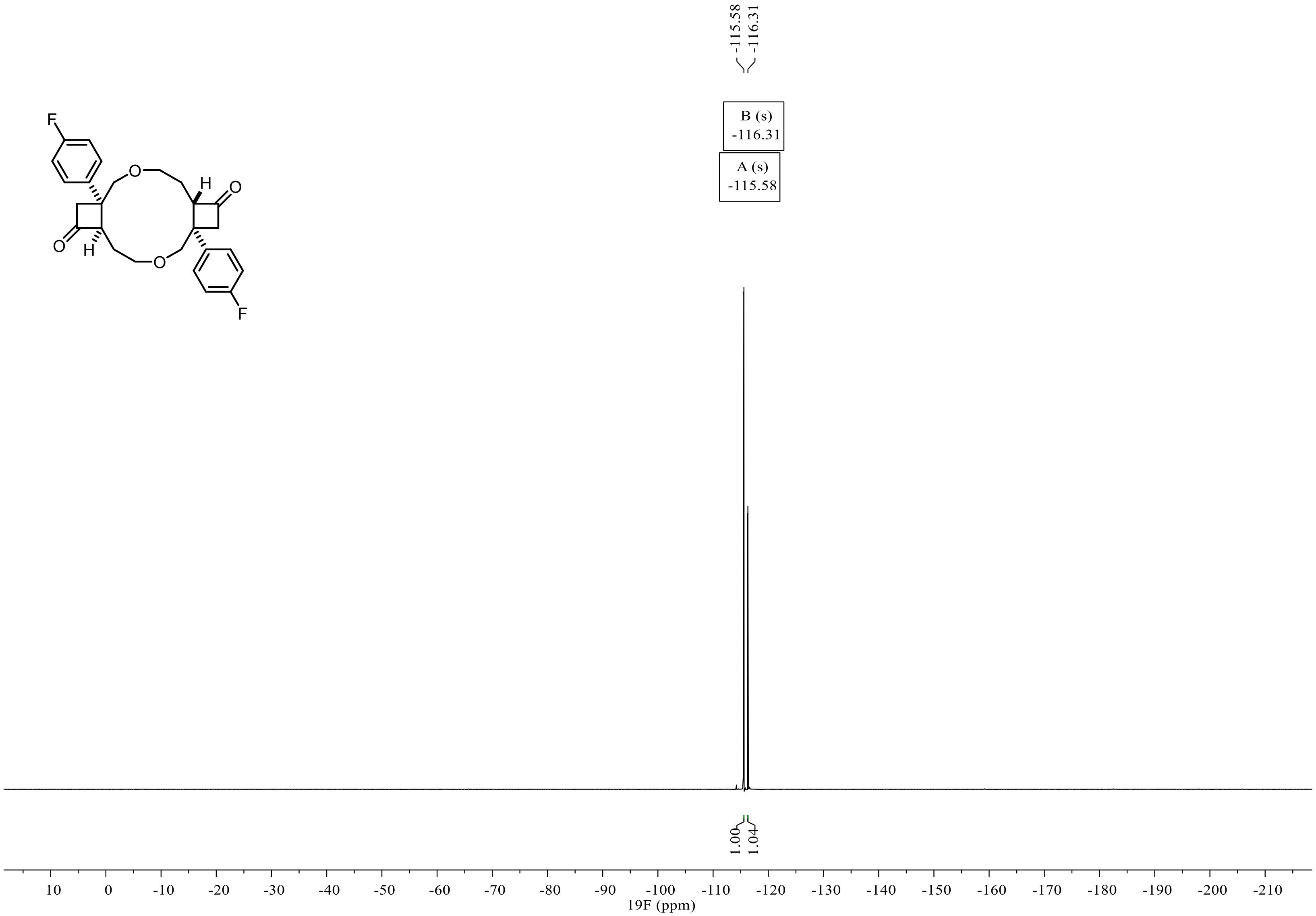

31e: (^{1}H NMR, $CDCl_3$, 400 MHz)

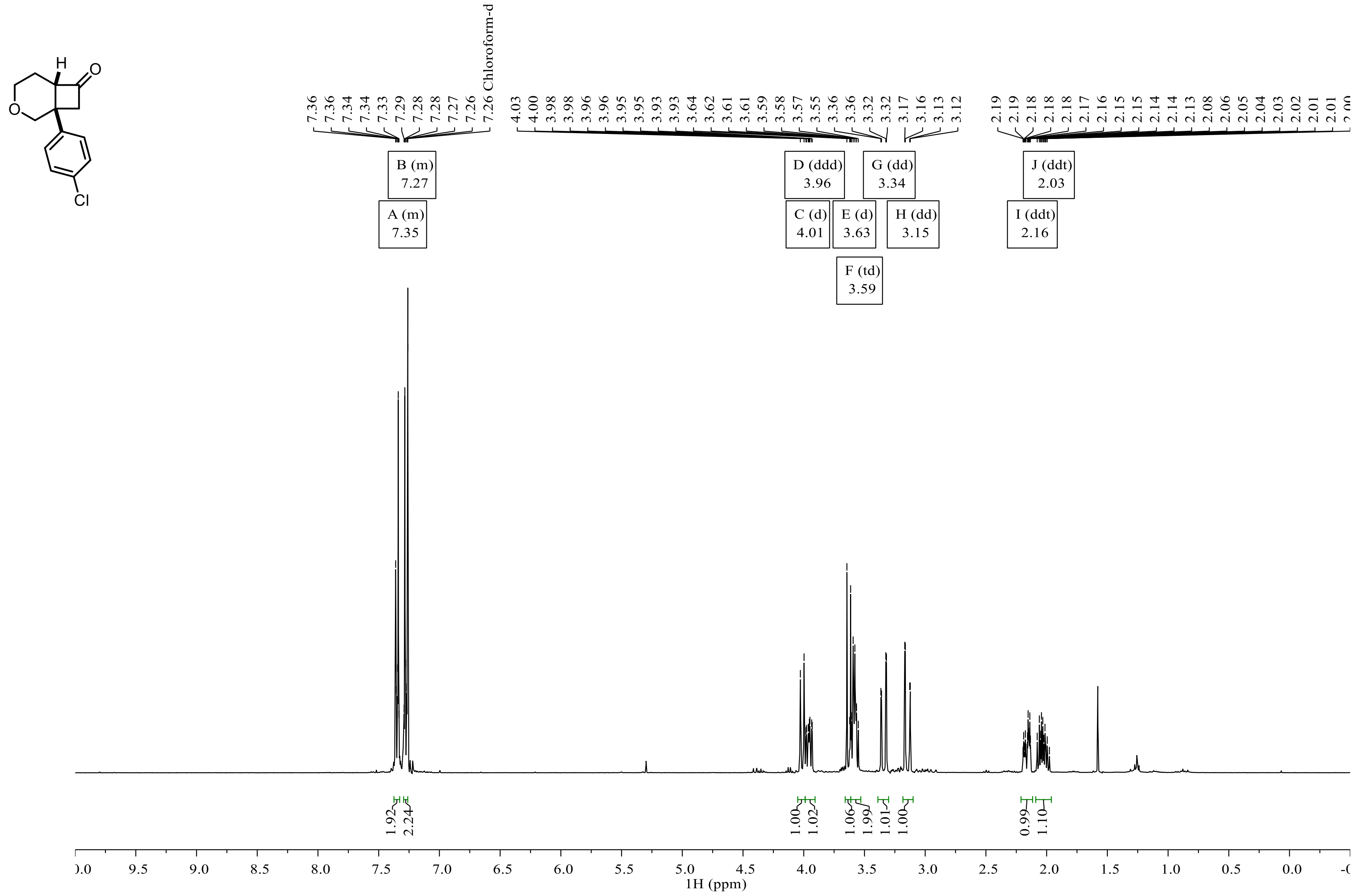

(^{13}C NMR, $CDCl_3$, 101 MHz)

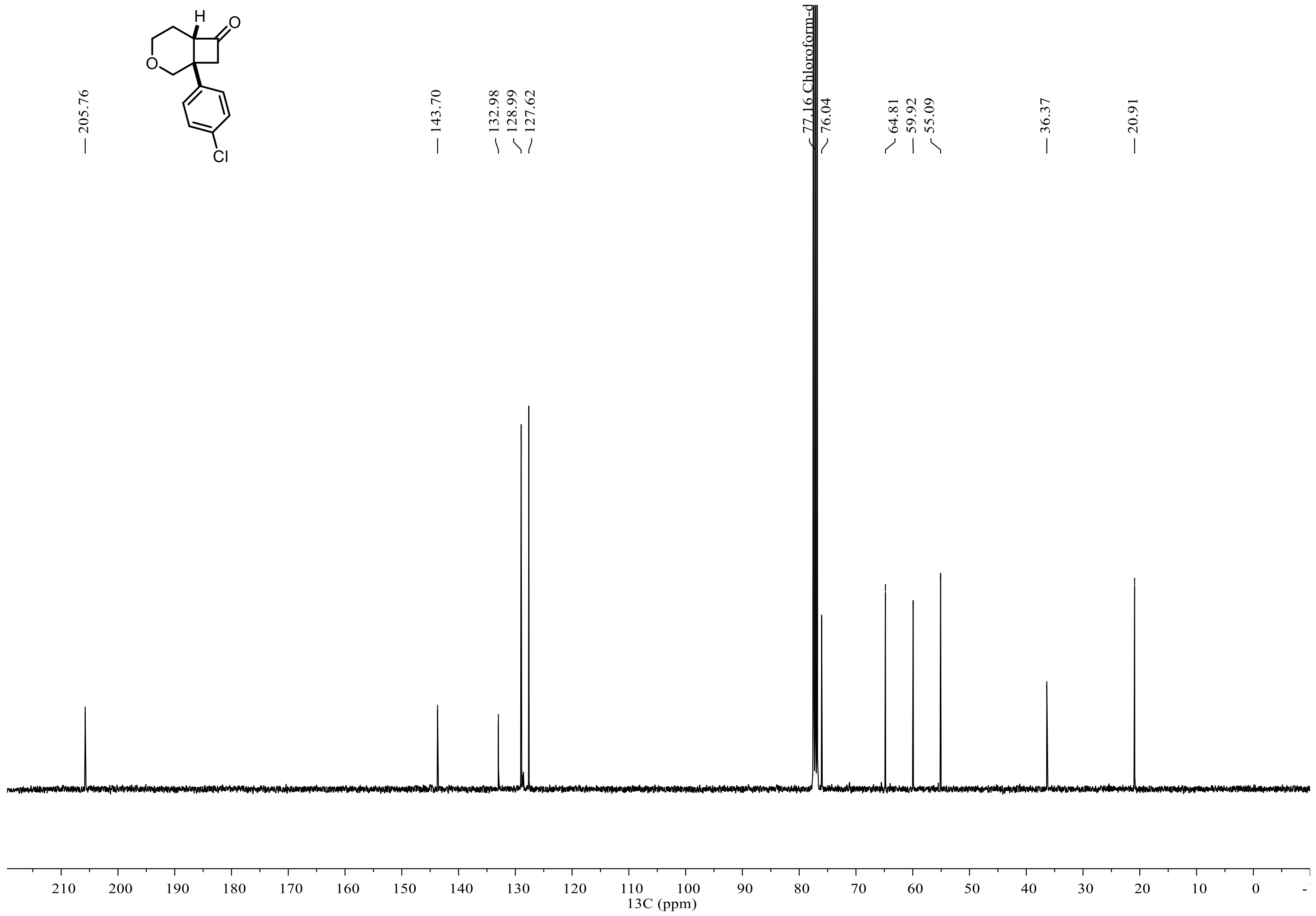

32e: (^{1}H NMR, $CDCl_3$, 400 MHz)

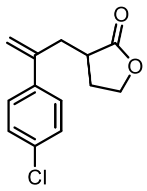

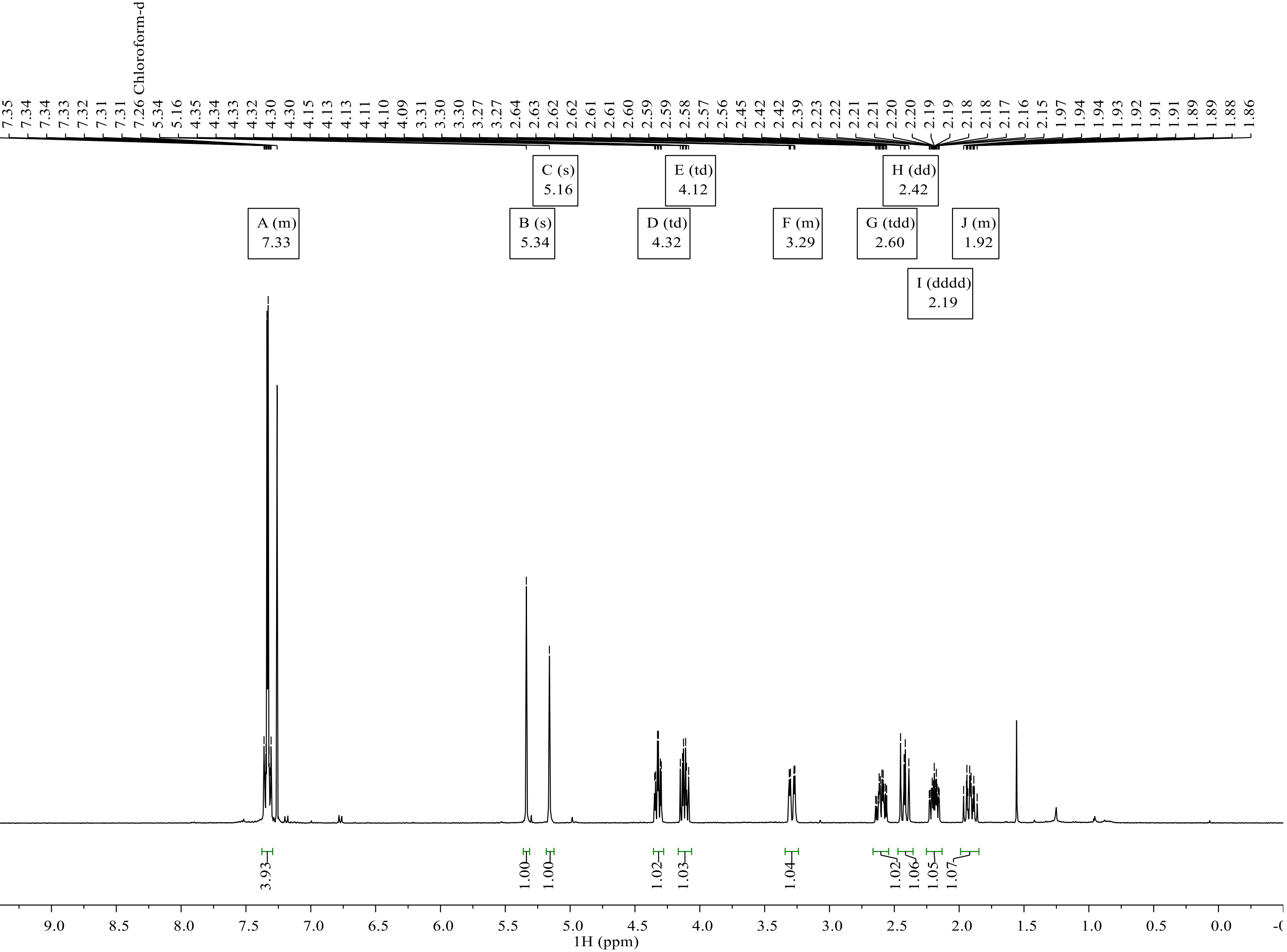

(^{13}C NMR, $CDCl_3$, 101 MHz)

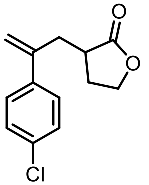

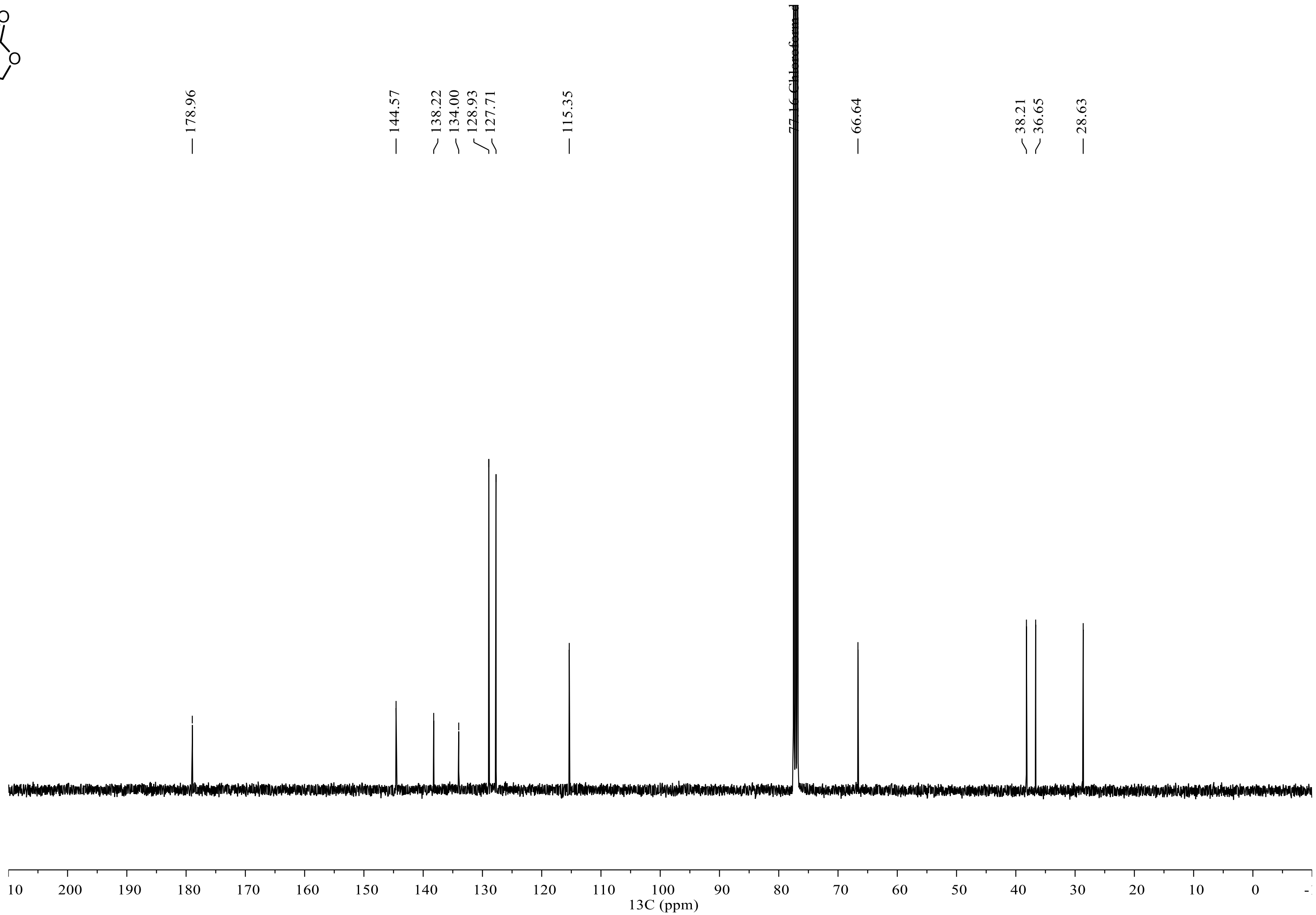

31f: (^{1}H NMR, $CDCl_3$, 400 MHz)

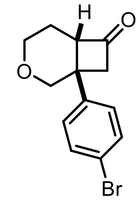

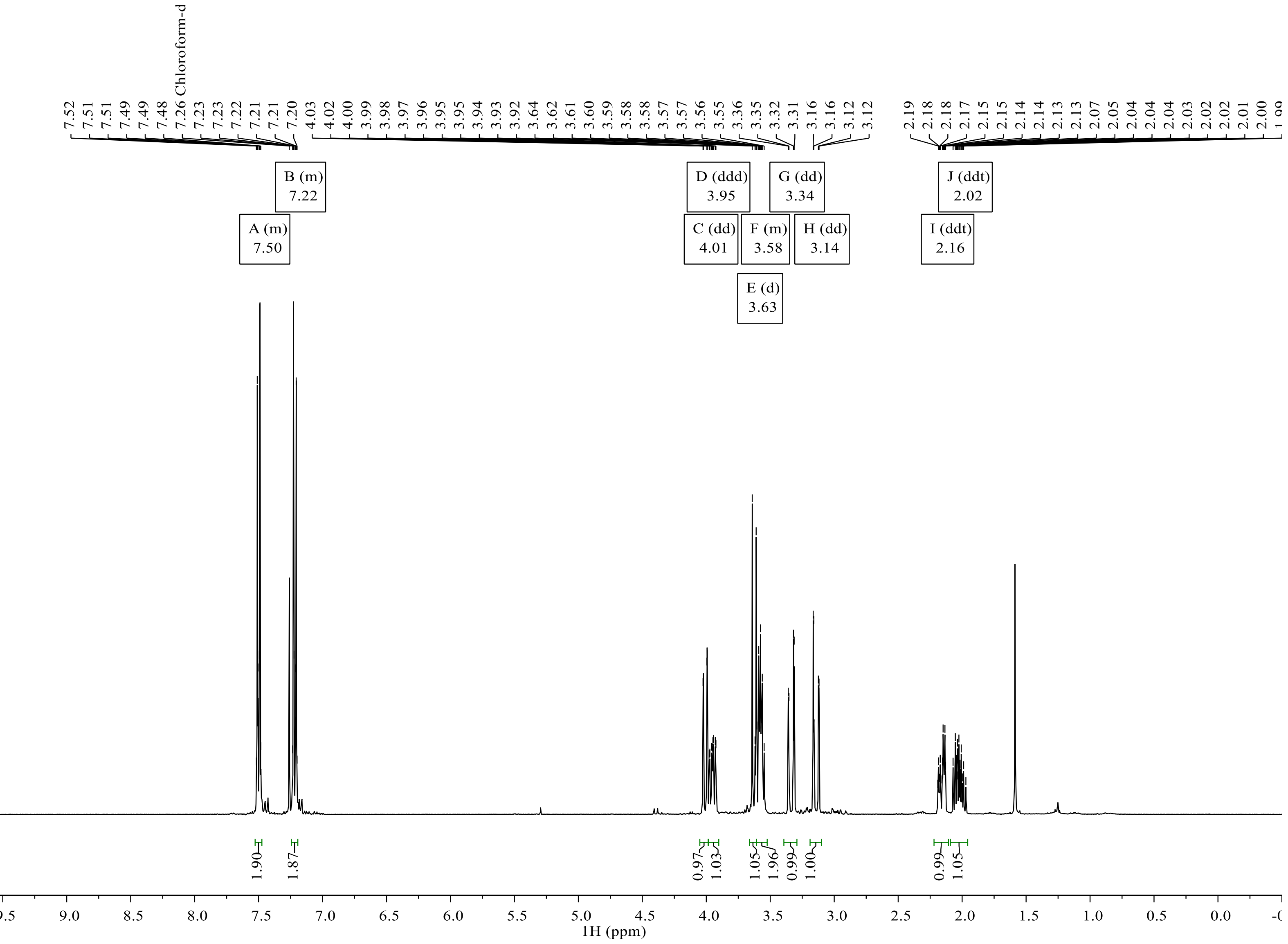

(^{13}C NMR, $CDCl_3$, 101 MHz)

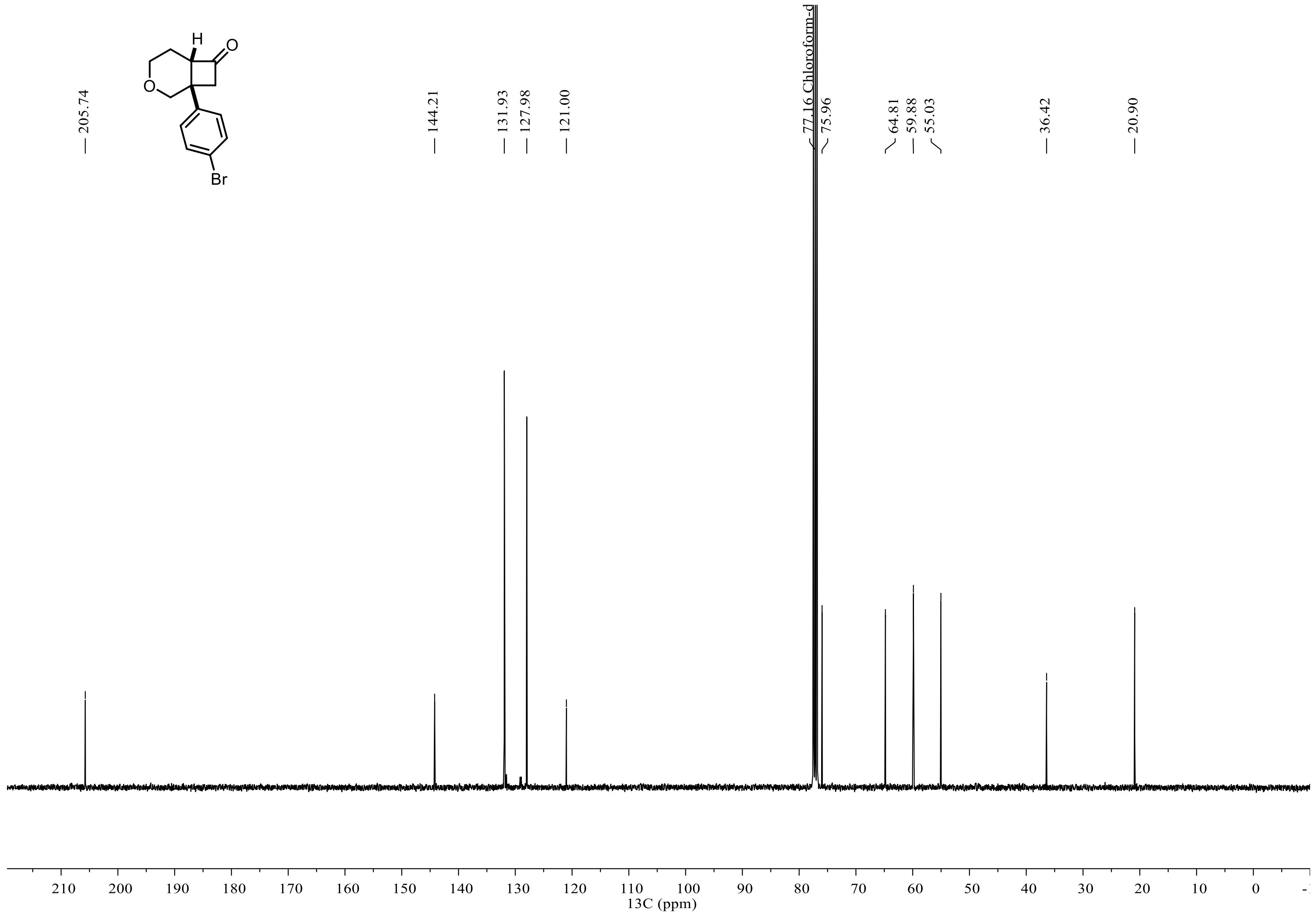

32f: (^{1}H NMR, $CDCl_3$, 400 MHz)

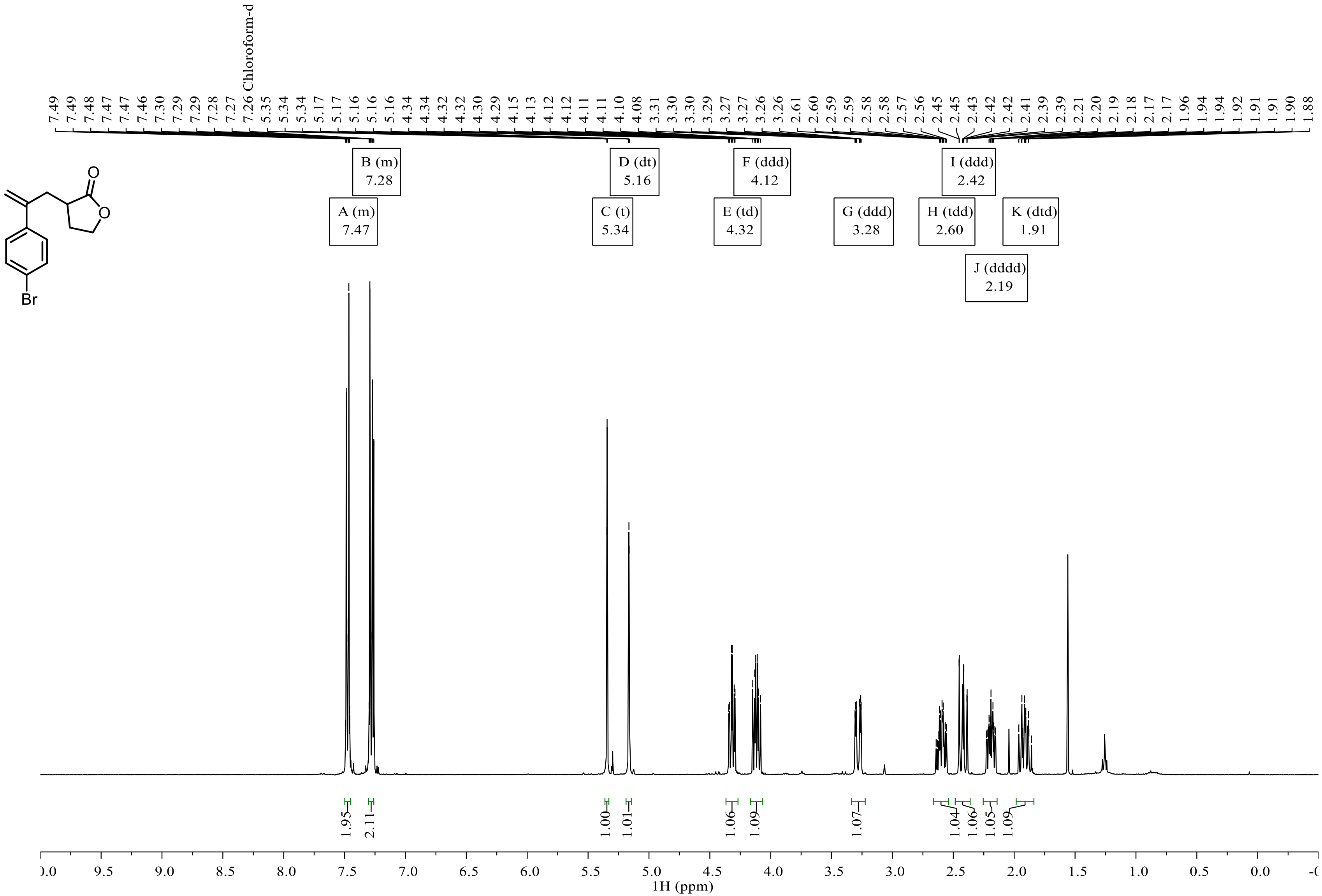

(^{13}C NMR, $CDCl_3$, 101 MHz)

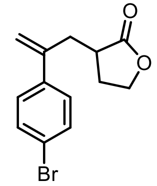

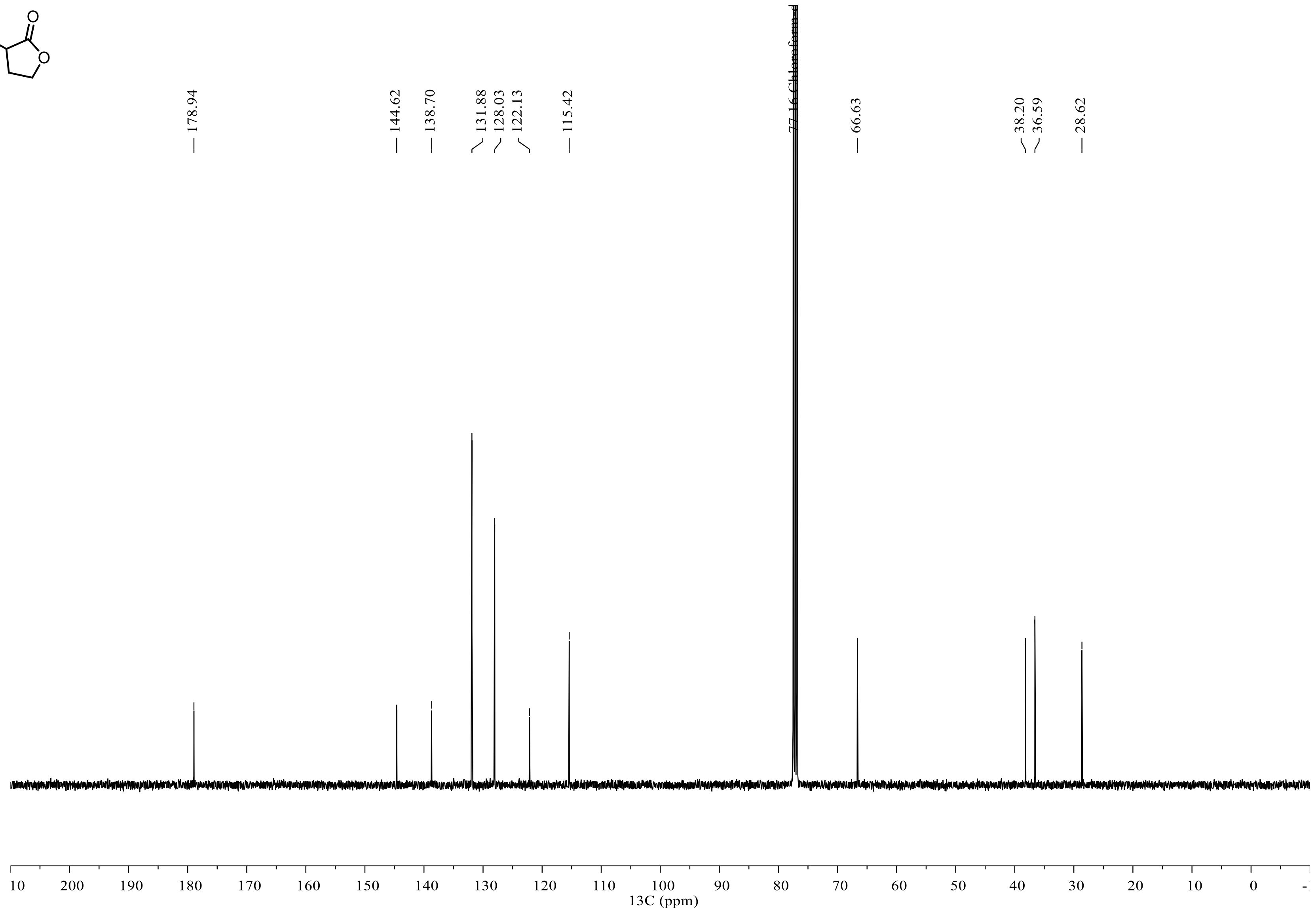

SI.13: (^{1}H NMR, $CDCl_3$, 400 MHz)

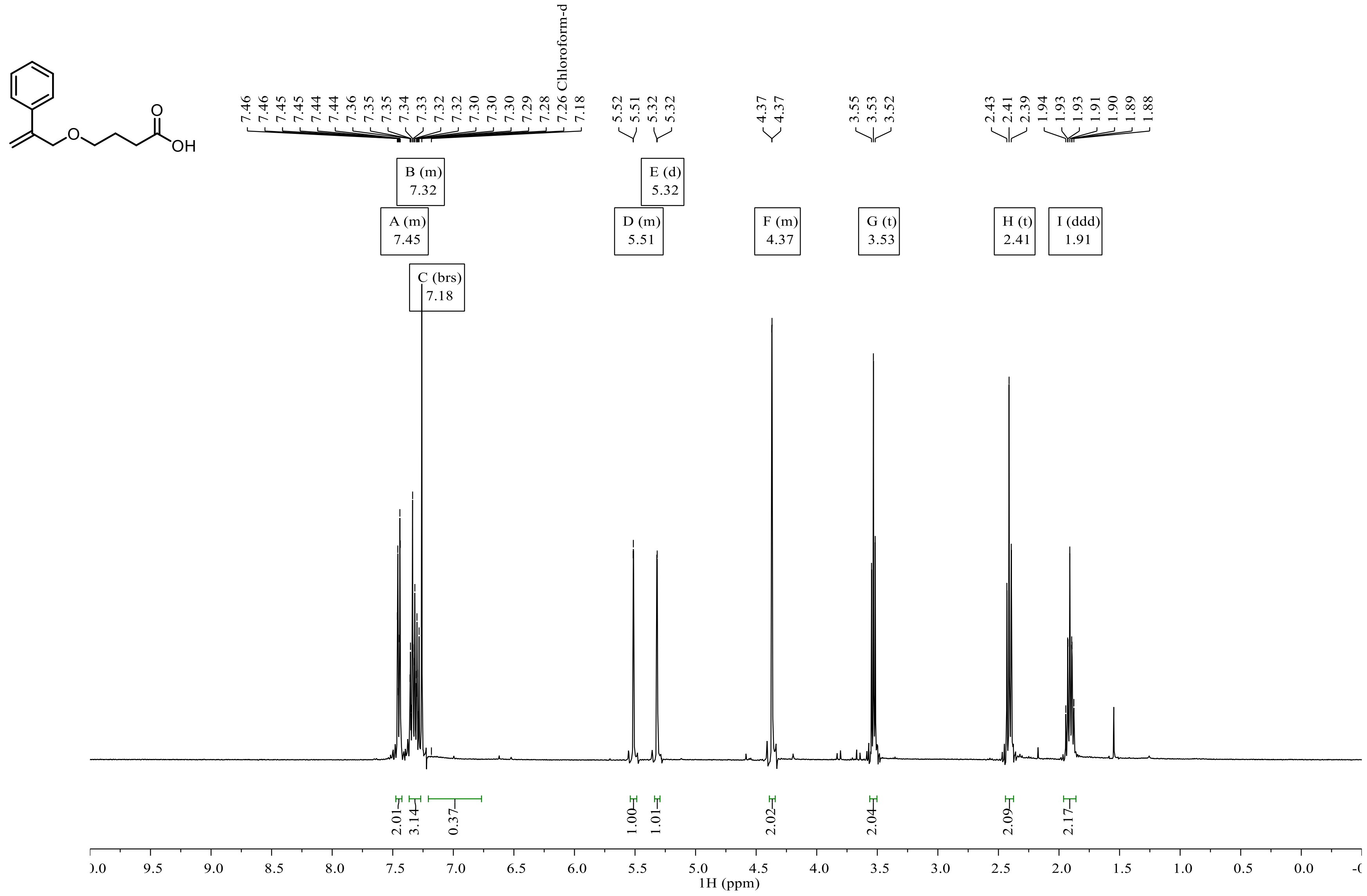

(^{13}C NMR, $CDCl_3$, 101 MHz)

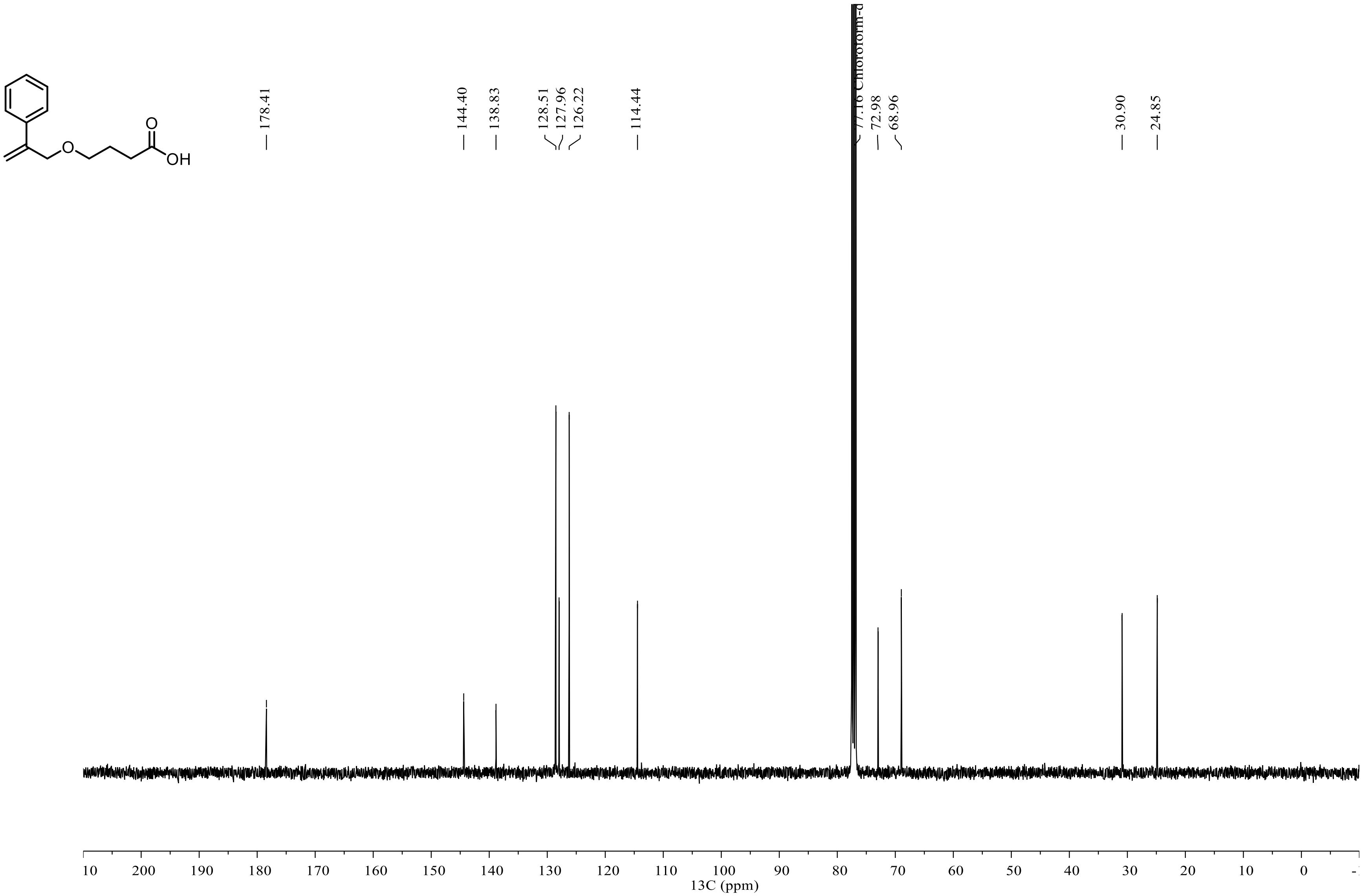

34: (^{1}H NMR, $CDCl_3$, 400 MHz)

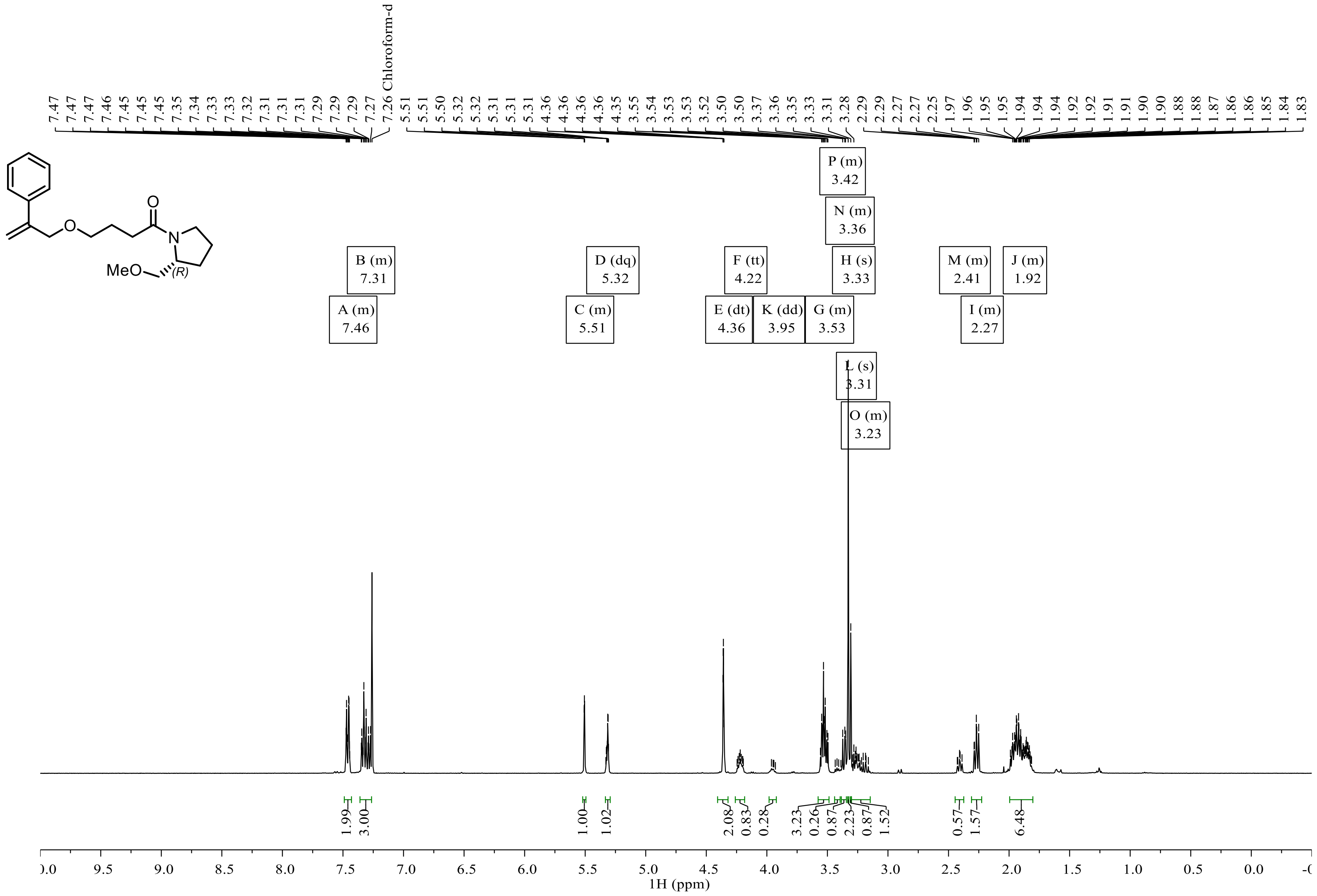

(^13^C NMR, $CDCl_3$, 101 MHz)

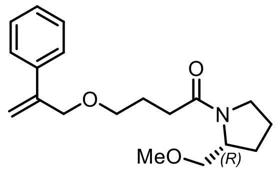

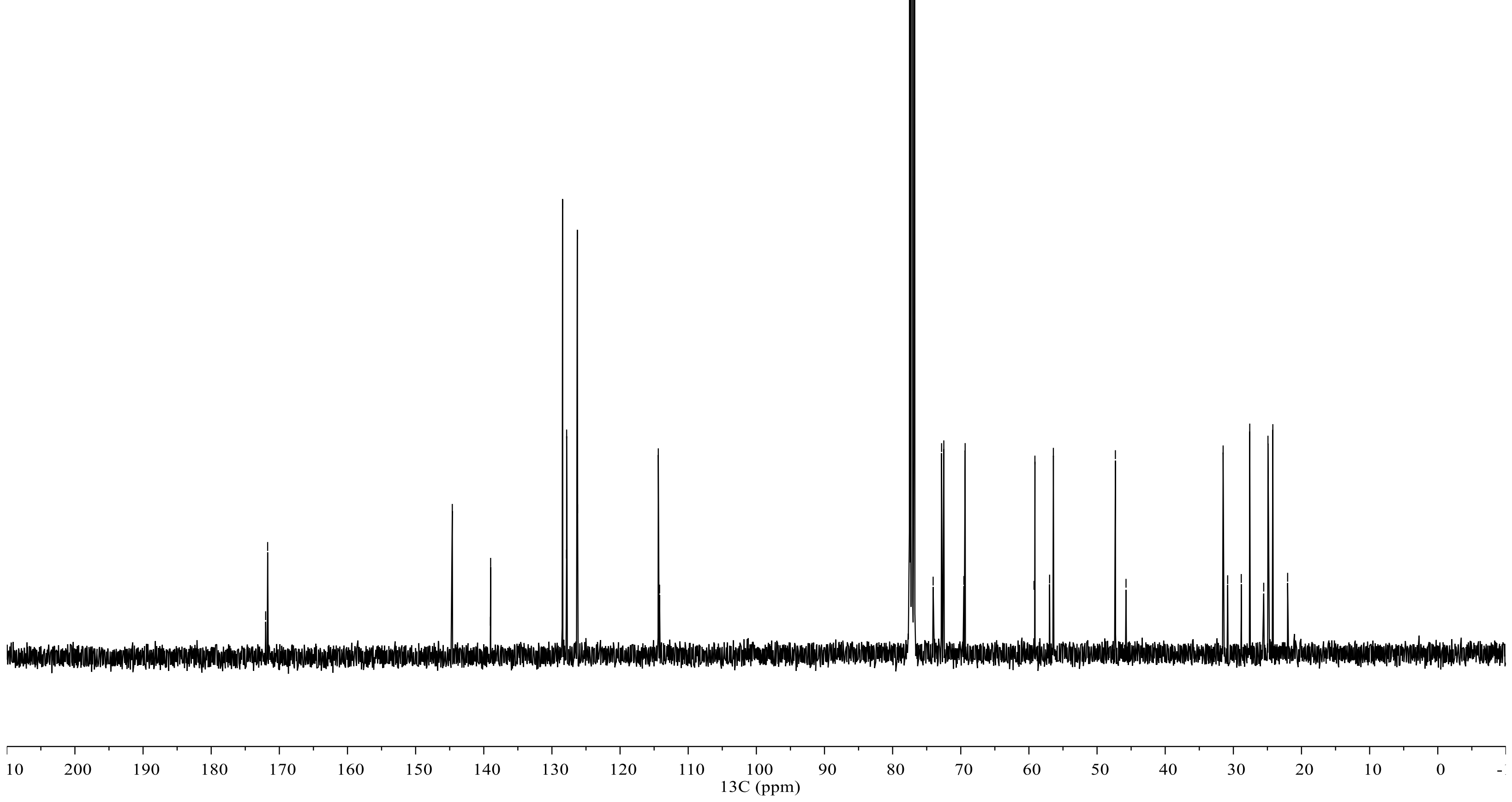

13PhCl: (^{1}H NMR, $CDCl_3$, 400 MHz)

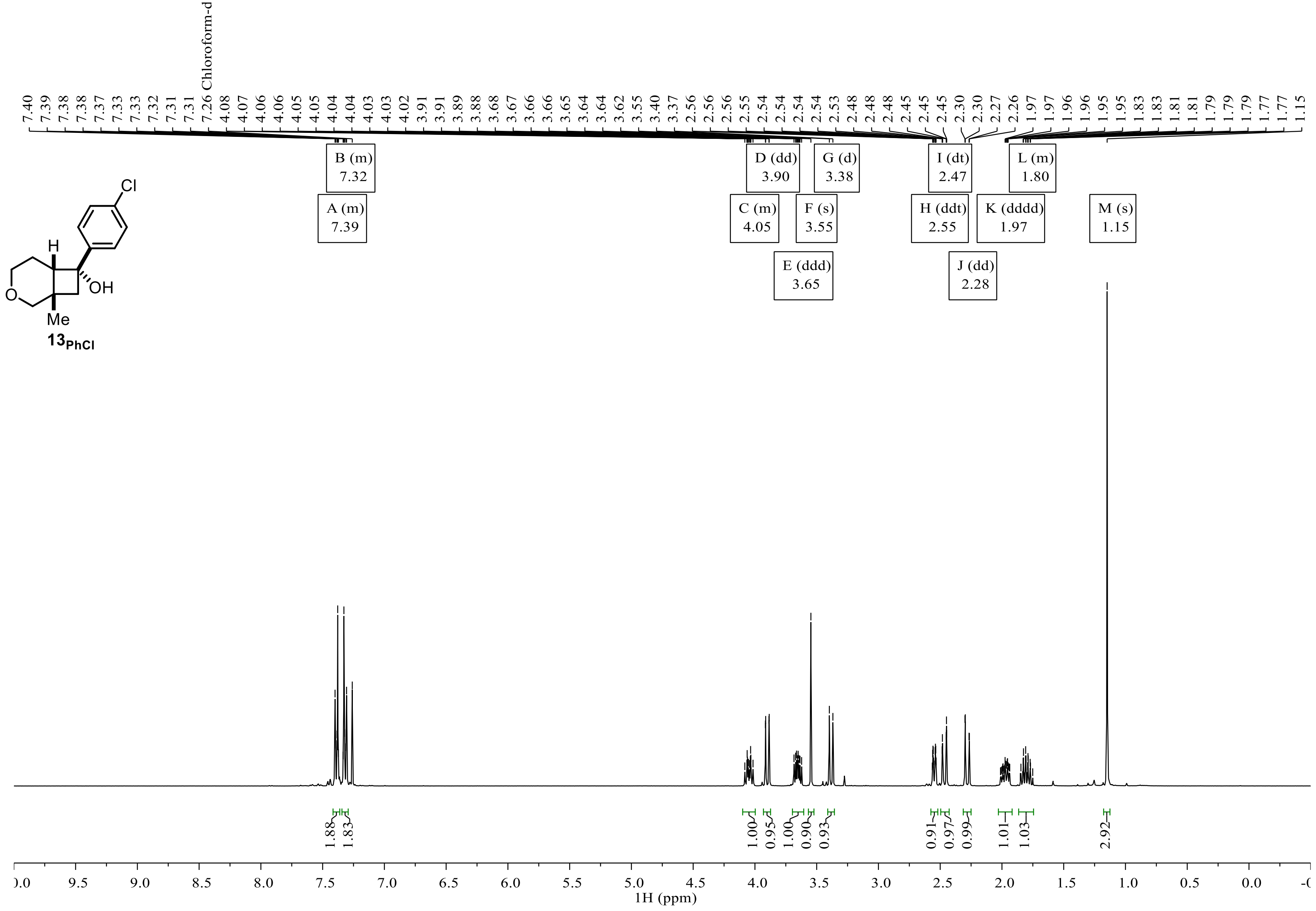

(^13C NMR, $CDCl_3$, 101 MHz)

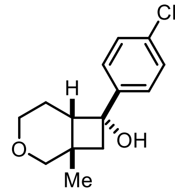

13_{PhCl}

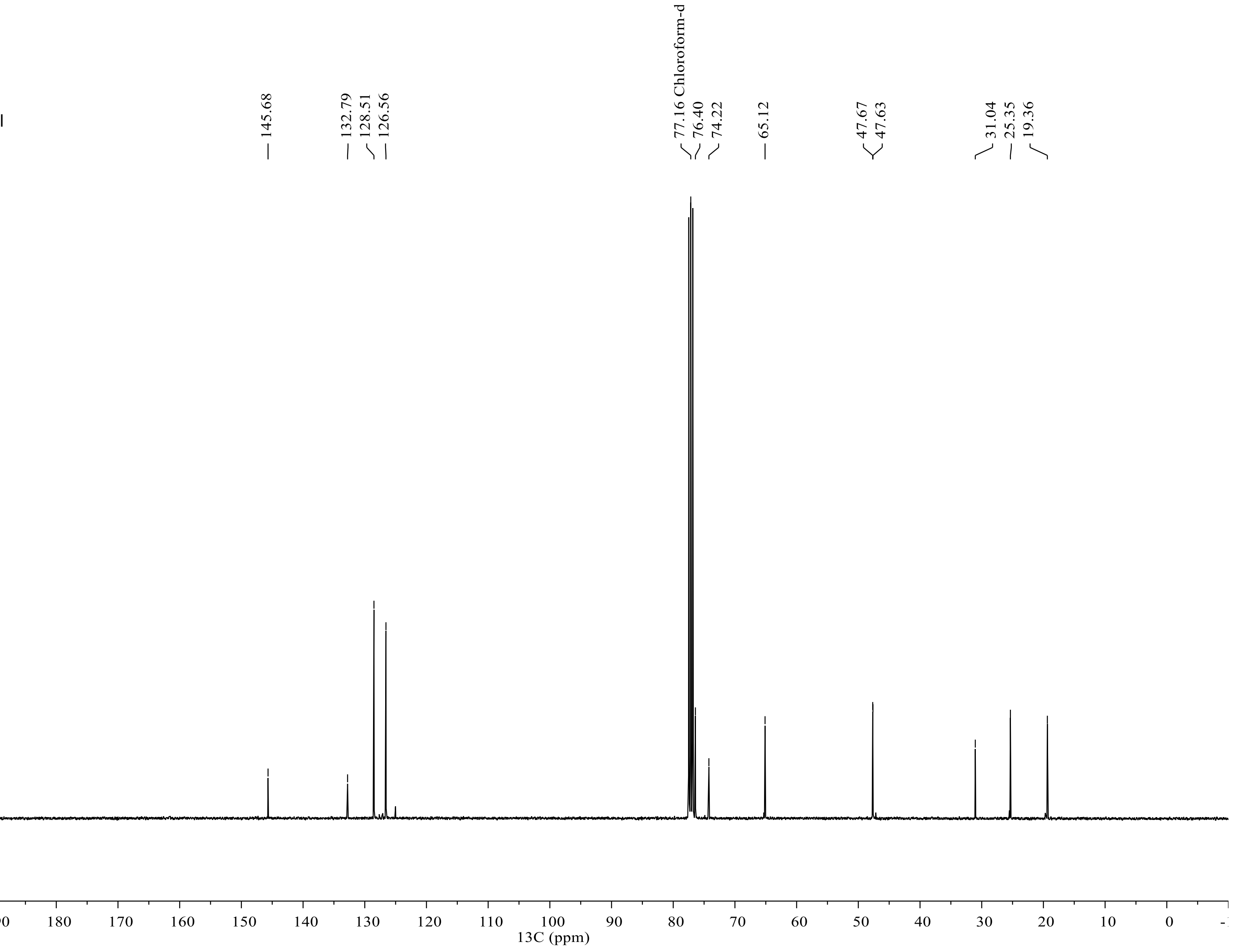

14PhCl: (^{1}H NMR, $CDCl_3$, 700 MHz)

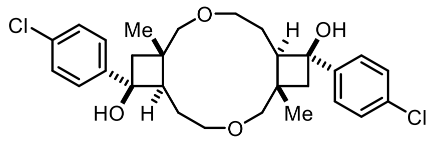

14PhCl

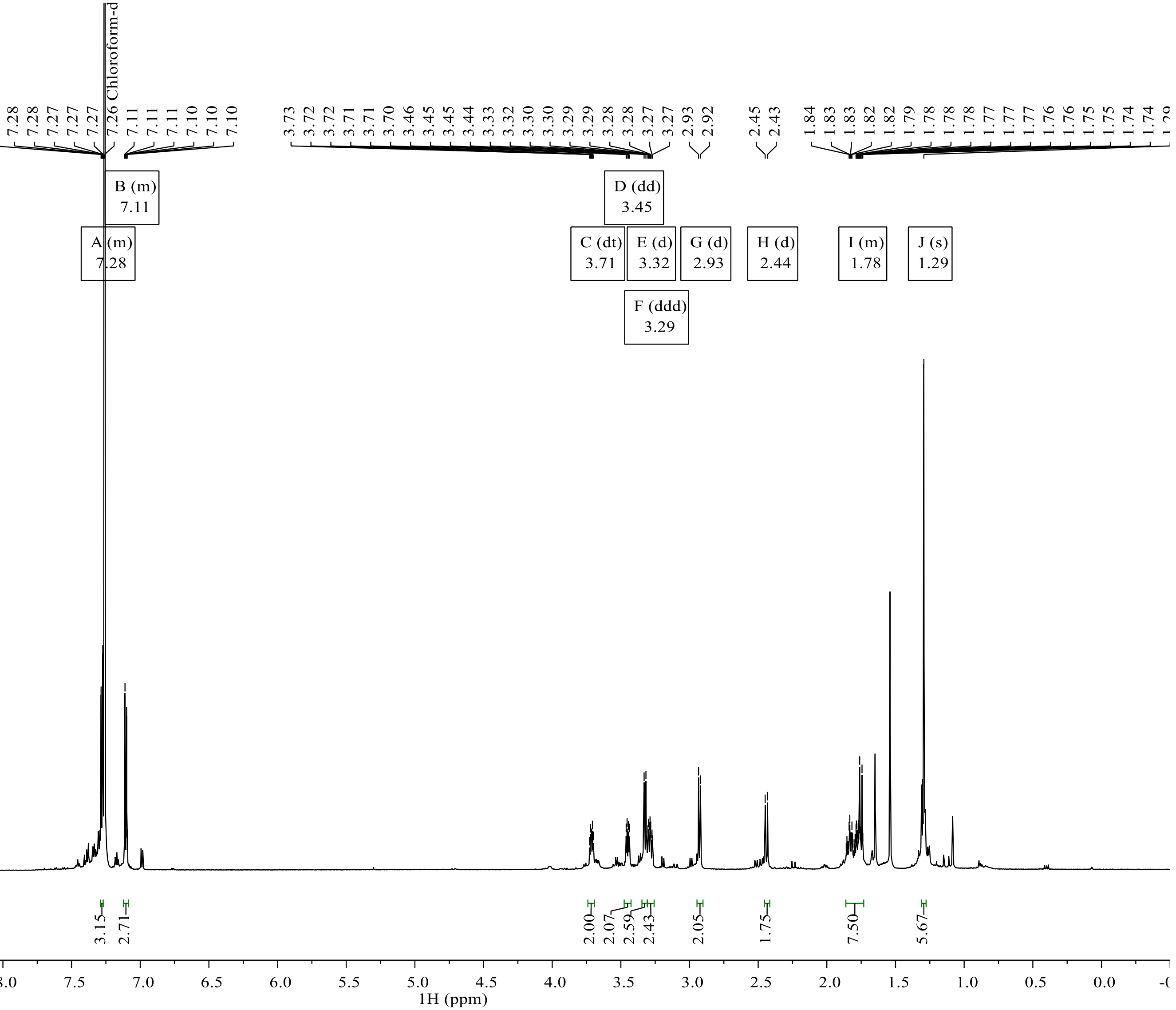

(^{13}C NMR, $CDCl_3$, 176 MHz)

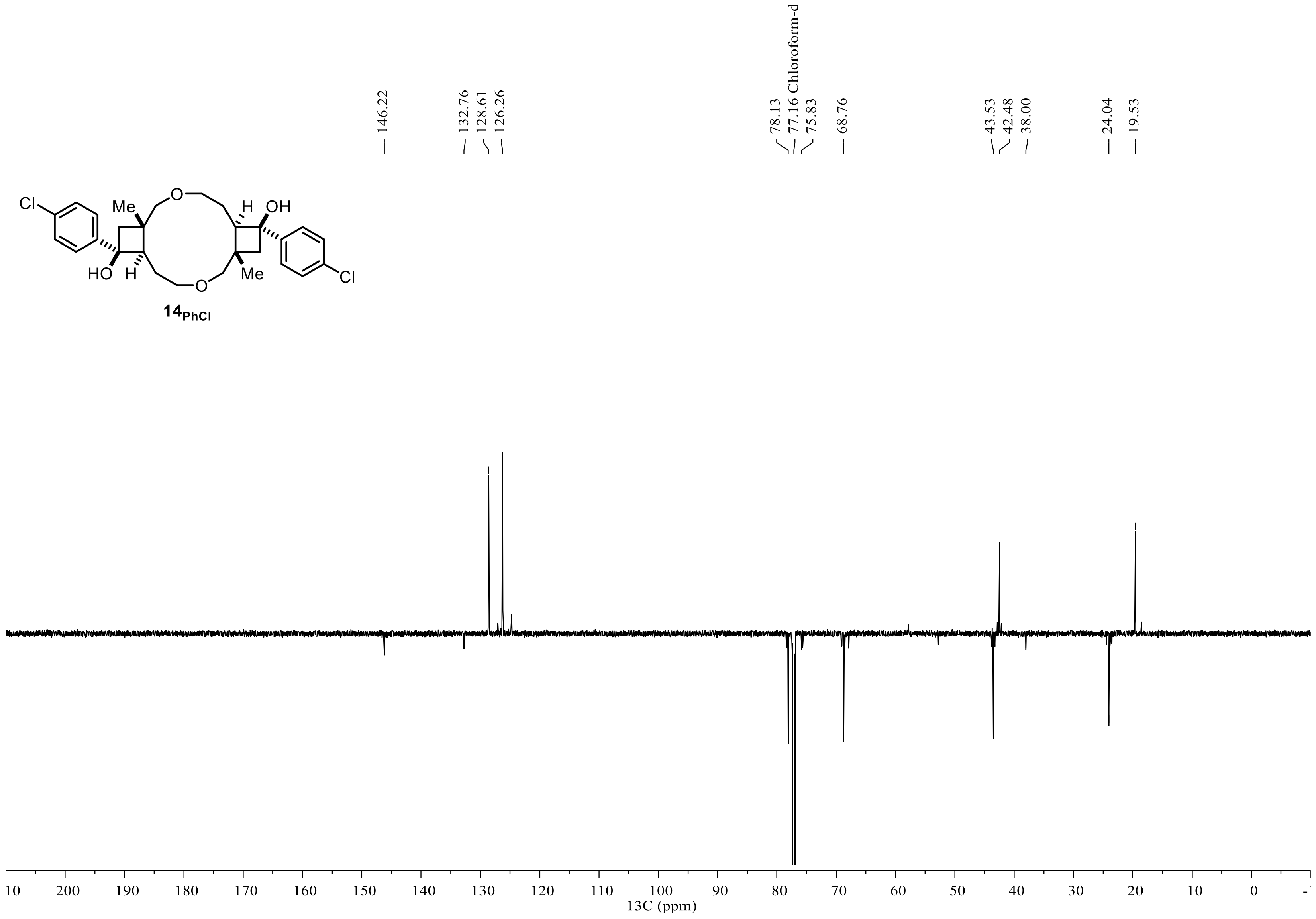

16: (^{1}H NMR, $CDCl_3$, 400 MHz)

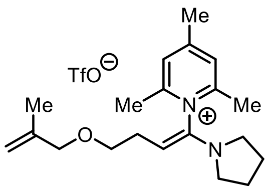

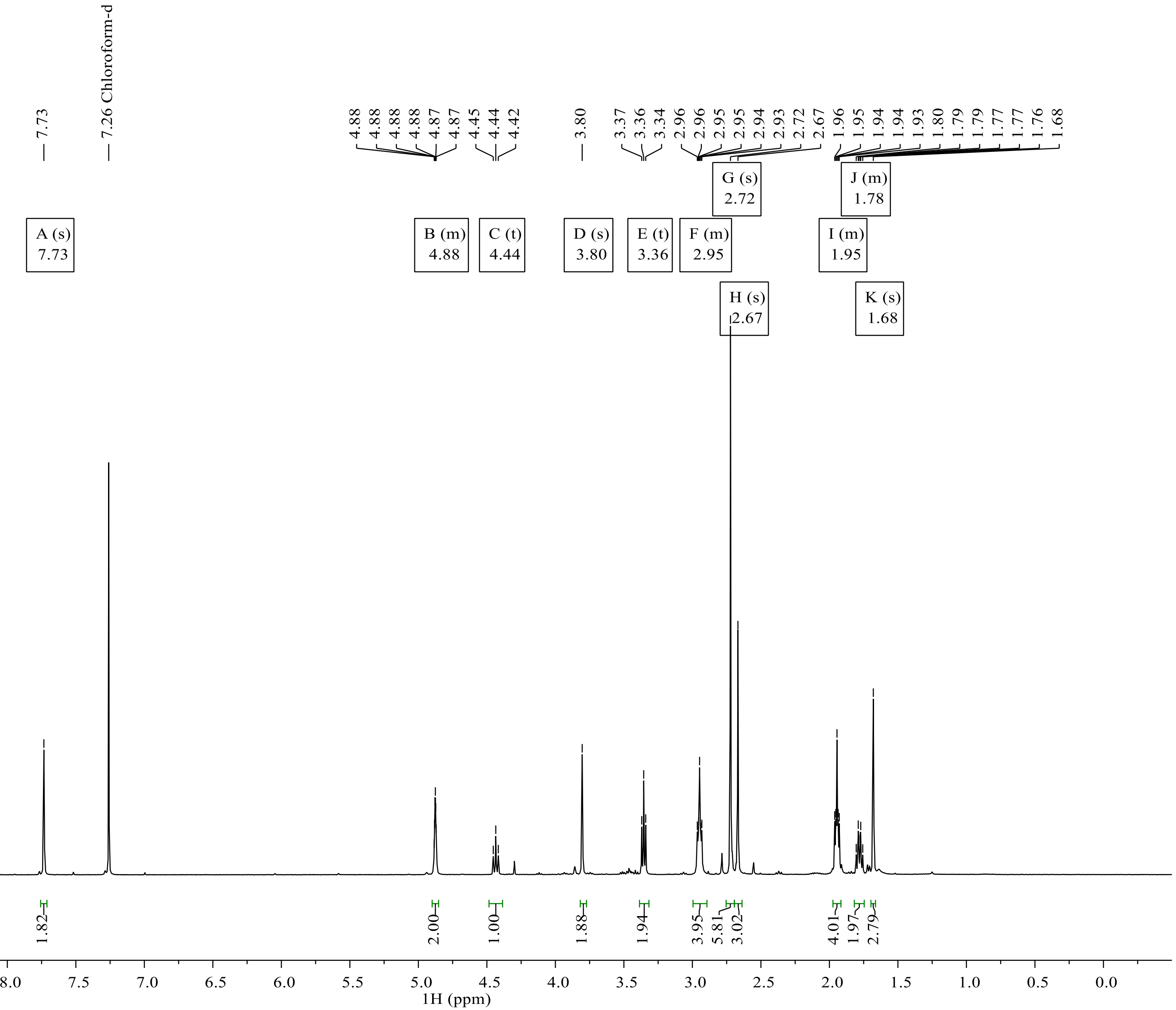

(^{13}C NMR, $CDCl_3$, 151 MHz)

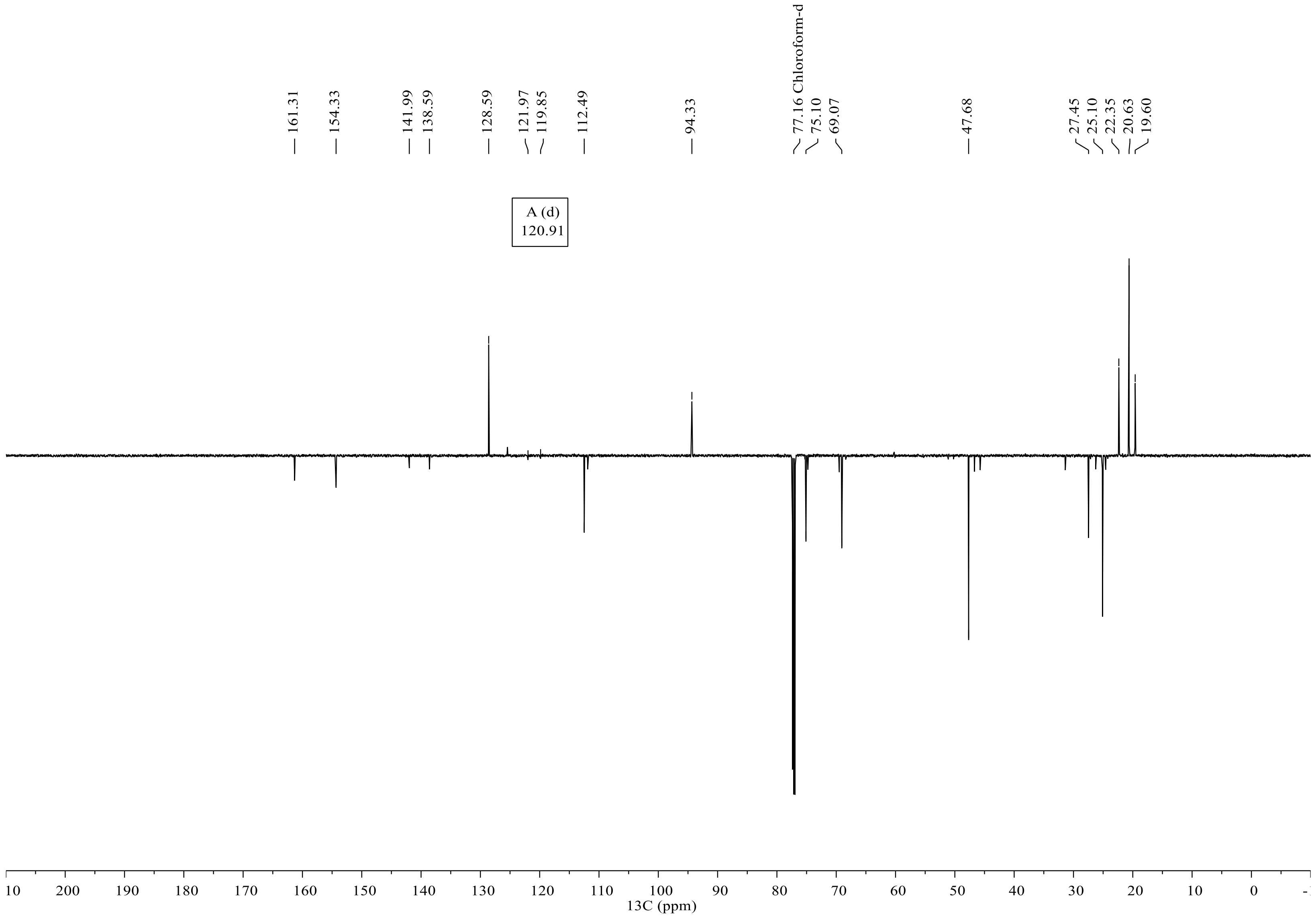

(^{19}F NMR, $CDCl_3$, 565 MHz)

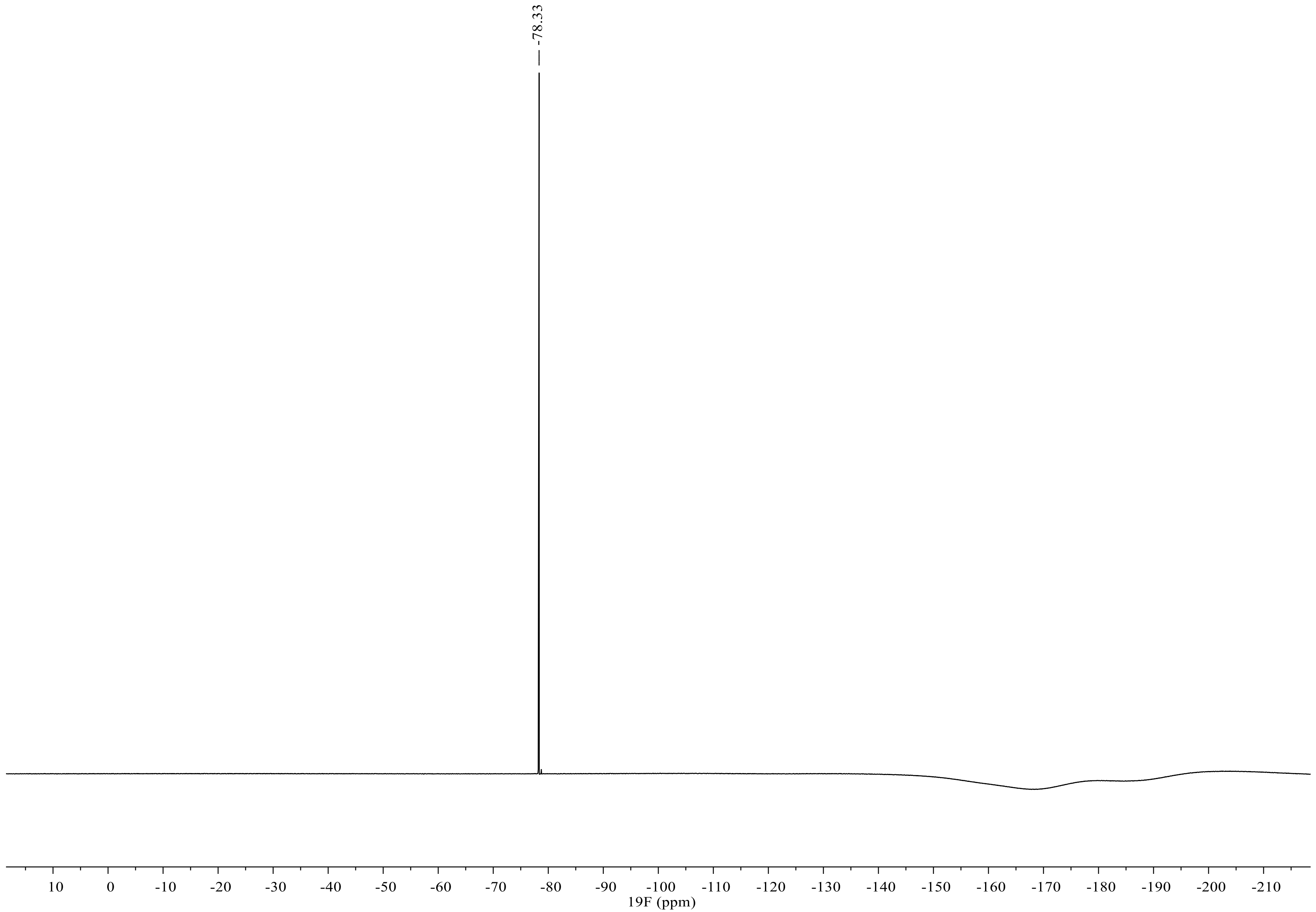

(^{1}H-^{1}H NOESY, $CDCl_3$, 600 MHz)

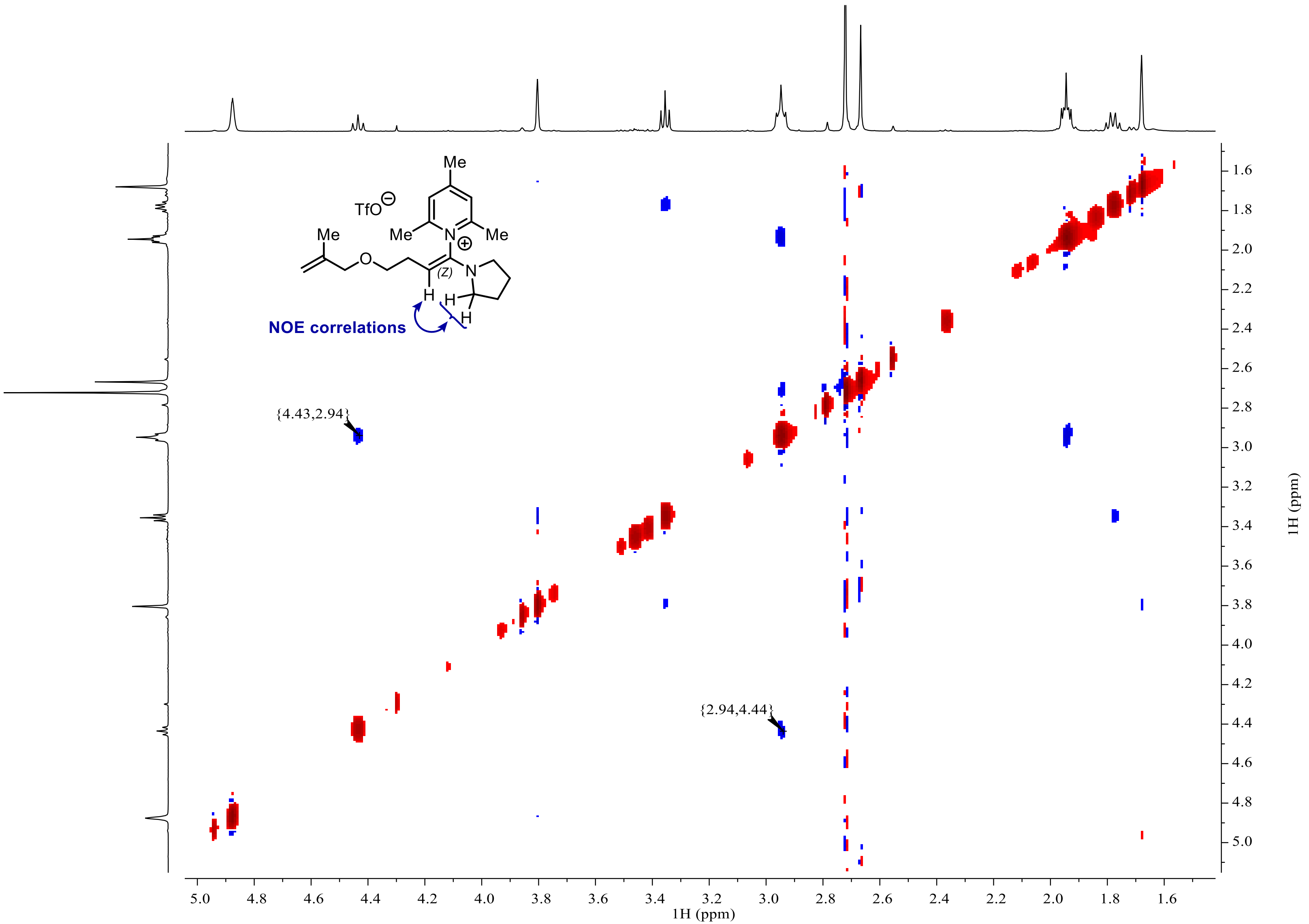

4. X-ray crystallography

13$_{PhCl}$:

CCDC number: 2174582

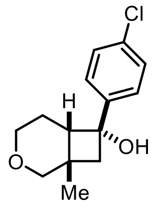

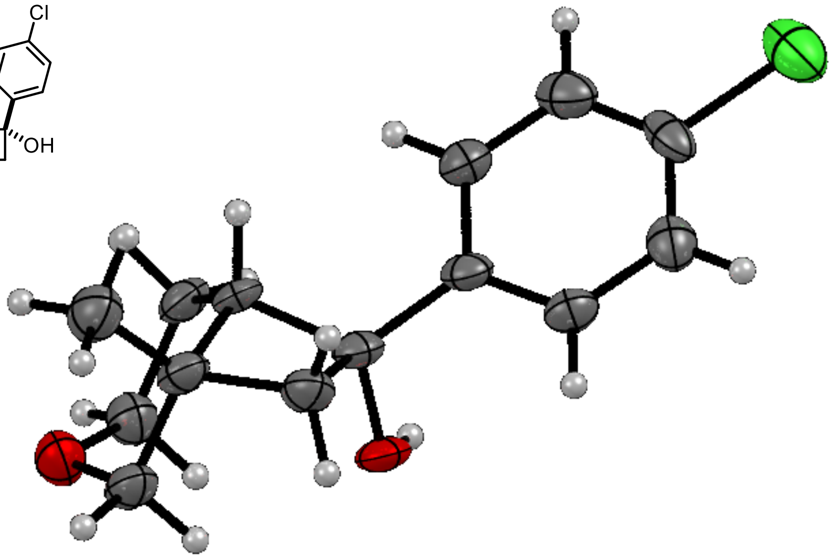

Identification code	ANFR_313_a	
Empirical formula	$C_{14}H_{17}ClO_2$	
Formula weight	252.72	
Temperature	100 K	
Wavelength	0.71073 Å	
Crystal system	Orthorhombic	
Space group	F d d 2	
Unit cell dimensions	a = 19.957(4) Å	a = 90°.
	b = 46.109(13) Å	b = 90°.
	c = 5.5709(12) Å	g = 90°.
Volume	5126(2) $Å^3$	
Z	16	
Density (calculated)	1.310 Mg/m^3	
Absorption coefficient	0.285 mm^{-1}	
F(000)	2144	
Crystal size	0.350 x 0.163 x 0.030 mm^3	
Theta range for data collection	1.767 to 27.146°.	
Index ranges	-24<=h<=24, -58<=k<=58, -7<=l<=7	
Reflections collected	24330	
Independent reflections	2719 [R(int) = 0.0811]	
Completeness to theta = 25.242°	99.3 %	
Absorption correction	Semi-empirical from equivalents	
Max. and min. transmission	0.9944 and 0.3932	
Refinement method	Full-matrix least-squares on F^2	
Data / restraints / parameters	2719 / 1 / 156	
Goodness-of-fit on F^2	0.828	
Final R indices [I>2sigma(I)]	R1 = 0.0483, wR2 = 0.1087	
R indices (all data)	R1 = 0.1041, wR2 = 0.1151	
Absolute structure parameter (Flack)	-0.04(11)	
Extinction coefficient	0.0013(3)	
Largest diff. peak and hole	0.506 and -0.223 e.$Å^{-3}$	

14PhCl:

CCDC number: 2173091

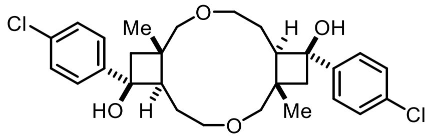

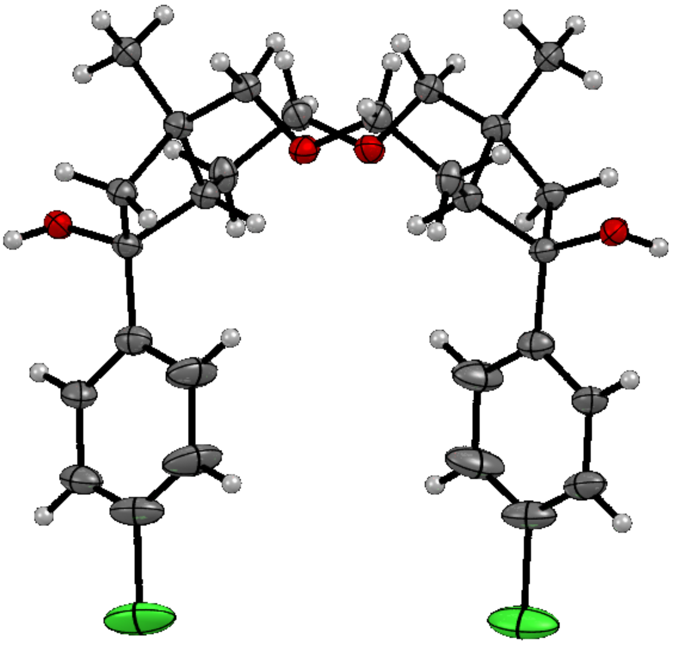

Identification code	ANFR_297_F1_a	
Empirical formula	$C_{14}H_{17}ClO_2$	
Formula weight	252.74	
Temperature	100 K	
Wavelength	0.71073 Å	
Crystal system	Tetragonal	
Space group	I -4	
Unit cell dimensions	a = 12.8356(9) Å	a = 90°.
	b = 12.8356(10) Å	b = 90°.
	c = 17.7160(13) Å	g = 90°.
Volume	2918.7(4) Å^3	
Z	8	
Density (calculated)	1.150 Mg/m^3	
Absorption coefficient	0.251 mm^{-1}	
F(000)	1072	
Crystal size	0.210 x 0.160 x 0.100 mm^3	
Theta range for data collection	2.299 to 29.172°.	
Index ranges	-17<=h<=17 -16<=k<=17, -24<=l<=23	
Reflections collected	33144	
Independent reflections	3580 [R(int) = 0.1757]	
Completeness to theta = 25.242°	98.0 %	
Absorption correction	Semi-empirical from equivalents	
Max. and min. transmission	0.9662 and 0.3679	
Refinement method	Full-matrix least-squares on F^2	
Data / restraints / parameters	3580 / 0 / 156	
Goodness-of-fit on F^2	0.992	
Final R indices [I>2sigma(I)]	R1 = 0.0777, wR2 = 0.1991	
R indices (all data)	R1 = 0.1056, wR2 = 0.2114	
Absolute structure parameter (Flack)	0.12(9)	
Extinction coefficient	n/a	
Largest diff. peak and hole	0.841 and -0.634 e.Å^{-3}	

31f:

CCDC number: 2145132

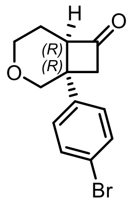

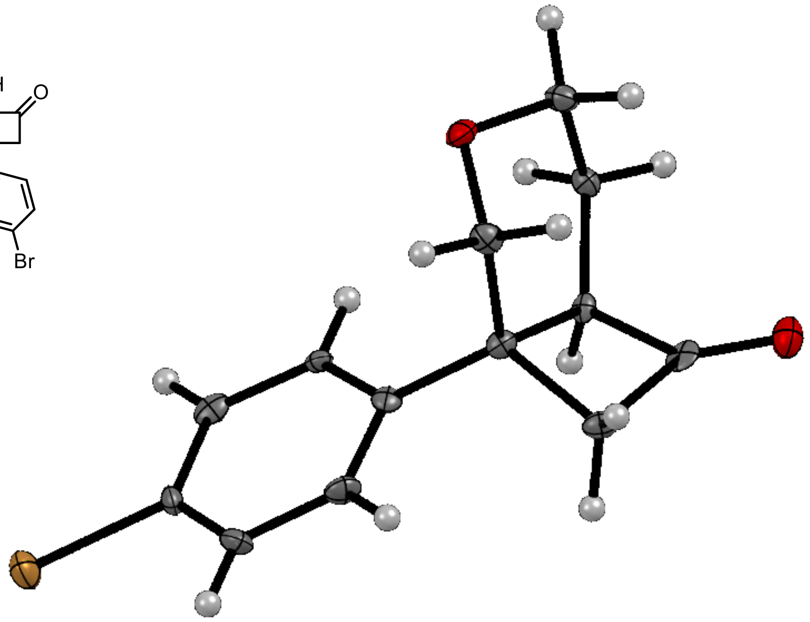

Identification code	ANFR_218_F2_a	
Empirical formula	$C_{13}H_{13}BrO_2$	
Formula weight	281.1485	
Temperature	100 K	
Wavelength	0.71073 Å	
Crystal system	Orthorhombic	
Space group	$P2_12_12_1$	
Unit cell dimensions	a = 8.1198(13) Å	a = 90°.
	b = 9.0016(14) Å	b = 90°.
	c = 15.531(2) Å	g = 90°.
Volume	1135.2(3) $Å^3$	
Z	4	
Density (calculated)	1.645 Mg/m^3	
Absorption coefficient	3.602 mm^{-1}	
F(000)	568	
Crystal size	0.200 x 0.093 x 0.030 mm^3	
Theta range for data collection	2.26 to 30.532°.	
Index ranges	-11<=h<=9, -12<=k<=12, -22<=l<=20	
Reflections collected	12478	
Independent reflections	3299 [R(int) = 0.0658]	
Completeness to theta = 25.242°	99.8 %	
Absorption correction	Semi-empirical from equivalents	
Max. and min. transmission	0.9967 and 0.7055	
Refinement method	Full-matrix least-squares on F^2	
Data / restraints / parameters	3299 / 0 / 145	
Goodness-of-fit on F^2	0.910	
Final R indices [I>2sigma(I)]	R1 = 0.0390, wR2 = 0.0540	
R indices (all data)	R1 = 0.0858, wR2 = 0.0592	
Absolute structure parameter (Flack)	0.005(12)	
Extinction coefficient	n/a	
Largest diff. peak and hole	0.615 and -0.577 e.$Å^{-3}$	

33d:

CCDC number: 2149707

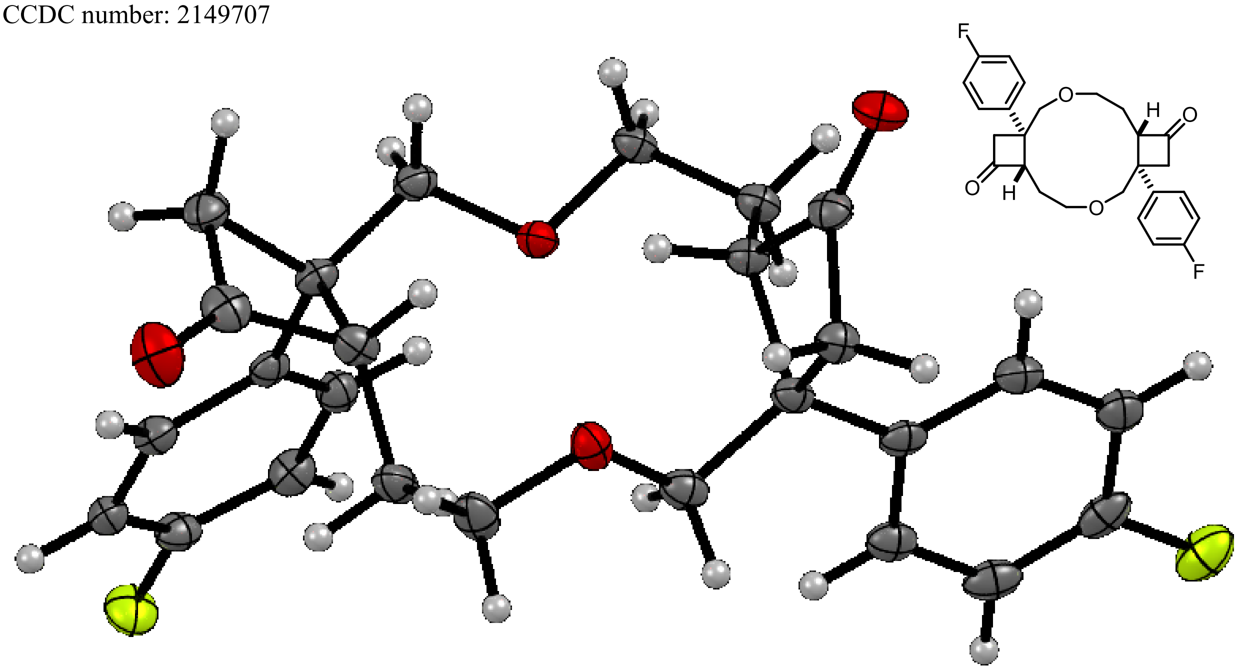

Identification code	ANFR_234_F3_a	
Empirical formula	$C_{26}H_{26}F_2O_4$	
Formula weight	440.47	
Temperature	100 K	
Wavelength	1.54178 Å	
Crystal system	Triclinic	
Space group	P -1	
Unit cell dimensions	a = 9.0782(7) Å	a = 81.134(6)°.
	b = 13.1363(10) Å	b = 84.589(6)°.
	c = 18.3651(15) Å	g = 79.086(6)°.
Volume	2120.0(3) $Å^3$	
Z	4	
Density (calculated)	1.380 Mg/m^3	
Absorption coefficient	0.862 mm^{-1}	
F(000)	928	
Crystal size	0.250 x 0.200 x 0.150 mm^3	
Theta range for data collection	2.44 to 71.33°.	
Index ranges	-11<=h<=10, -16<=k<=12, -14<=l<=22	
Reflections collected	34471	
Independent reflections	7569 [R(int) = 0.0254]	
Completeness to theta = 67.679°	94.3 %	
Absorption correction	Semi-empirical from equivalents	
Max. and min. transmission	0.6978 and 0.6419	
Refinement method	Full-matrix least-squares on F^2	
Data / restraints / parameters	7569 / 0 / 577	
Goodness-of-fit on F^2	1.035	
Final R indices [I>2sigma(I)]	R1 = 0.0351, wR2 = 0.0820	
R indices (all data)	R1 = 0.0526, wR2 = 0.0877	
Extinction coefficient	n/a	
Largest diff. peak and hole	0.239 and -0.220 e.$Å^{-3}$	

33d':

CCDC number: 2154716

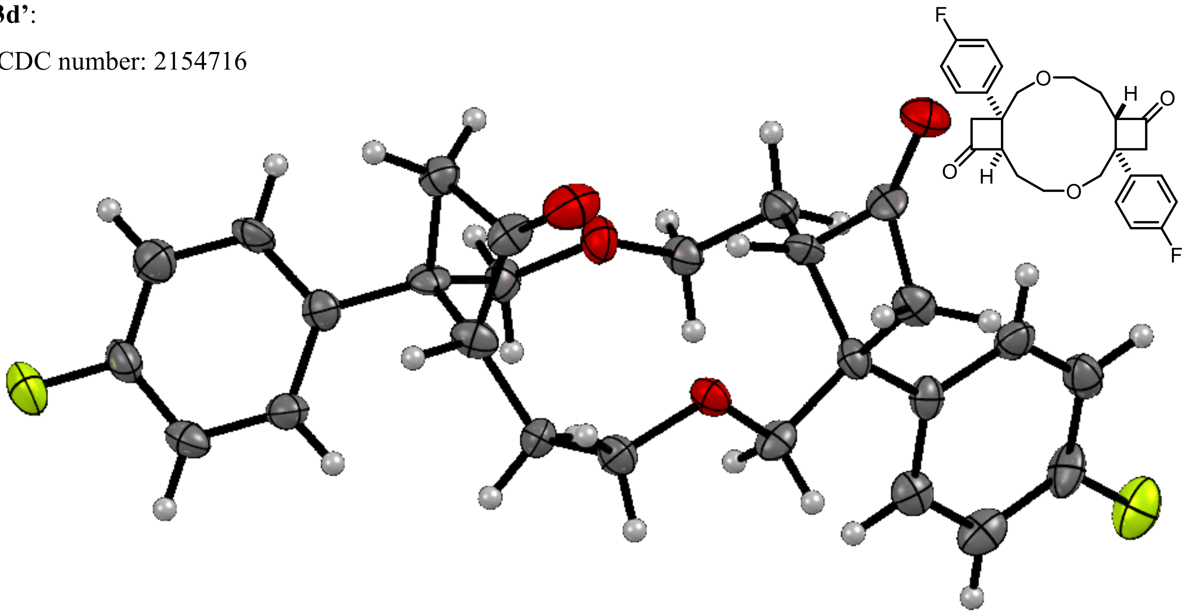

Identification code	ANFR_234_F3_cis-trans	
Empirical formula	$C_{26}H_{26}F_2O_4$	
Formula weight	440.47	
Temperature	100 K	
Wavelength	1.54178 Å	
Crystal system	Monoclinic	
Space group	P 2_1/c	
Unit cell dimensions	a = 16.490(2) Å	a = 90°.
	b = 13.7980(15) Å	b = 90.082(10)°.
	c = 19.195(3) Å	g = 90°.
Volume	4367.3(9) $Å^3$	
Z	8	
Density (calculated)	1.340 Mg/m^3	
Absorption coefficient	0.836 mm^{-1}	
F(000)	1856	
Crystal size	0.120 x 0.057 x 0.020 mm^3	
Theta range for data collection	2.680 to 50.433°.	
Index ranges	-16<=h<=16, -12<=k<=13, -19<=l<=14	
Reflections collected	18096	
Independent reflections	4546 [R(int) = 0.1094]	
Completeness to theta = 67.679°	99.3 %	
Absorption correction	Semi-empirical from equivalents	
Max. and min. transmission	0.9988 and 0.7638	
Refinement method	Full-matrix least-squares on F^2	
Data / restraints / parameters	4546 / 0 / 577	
Goodness-of-fit on F^2	1.016	
Final R indices [I>2sigma(I)]	R1 = 0.0581, wR2 = 0.0903	
R indices (all data)	R1 = 0.1494, wR2 = 0.1159	
Extinction coefficient	n/a	
Largest diff. peak and hole	0.237 and -0.277 e.$Å^{-3}$	

5. Computational part

5.1. General Computational Procedure

Quantum mechanical investigations of the intramolecular reactions of keteniminium ion **2** and congeners were conducted with density functional theory (DFT) calculations using Gaussian 16 (full citation below).

Geometries were prepared and conformationally sampled with Grimme's xTB[13] (ver. 6.3.2 and 6.4.1) /CREST[14] (ver. 2.10.2, 2.11) and Zimmerman's GSM[15] codes. xTB optimization was conducted at the GFN-XTB-2 level of theory with dichloromethane implicit solvation (6.3.2: GBSA; 6.4.1: ALPB). Conformational sampling with CREST was conducted at the GFN-XTB-2 level of theory with dichloromethane ALPB implicit solvation, an RMSD threshold of 0.5 Å, and for ion pairs, confinement by ellipsoidal potential (non-covalent interaction mode [-nci]). For ion pairs, multiple low-energy non-degenerate structures were selected for DFT optimization.

Initial DFT geometry optimizations were completed at the ωB97X-D/def2-SVP level with the solvation model based on density (SMD) for dichloromethane.[16-18] Single-point corrections to energy were made at the ωB97X-D/def2-TZVPP level with the SMD for dichloromethane.

Quasiharmonic corrections to enthalpy (Head-Gordon) and entropy (Grimme) were made using Paton's GoodVibes software[19-21] (ver. 3.0.2) and incorporated single-point corrections to energy. Temperature corrections were applied to 393.15 K (120 °C) unless otherwise specified. Free energies were balanced where appropriate with equivalents of water, pyridine base, and pyridinium triflate. Visualizations were prepared with Legault's CYLview20[22] and Gilbert's IQmol.

5.2. Complete Authorship of Gaussian 16

Gaussian 16, Revision A.03, M. J. Frisch, G. W. Trucks, H. B. Schlegel, G. E. Scuseria, M. A. Robb, J. R. Cheeseman, G. Scalmani, V. Barone, G. A. Petersson, H. Nakatsuji, X. Li, M. Caricato, A. V. Marenich, J. Bloino, B. G. Janesko, R. Gomperts, B. Mennucci, H. P. Hratchian, J. V. Ortiz, A. F. Izmaylov, J. L. Sonnenberg, D. Williams-Young, F. Ding, F. Lipparini, F. Egidi, J. Goings, B. Peng, A. Petrone, T. Henderson, D. Ranasinghe, V. G. Zakrzewski, J. Gao, N. Rega, G. Zheng, W. Liang, M. Hada, M. Ehara, K. Toyota, R. Fukuda, J. Hasegawa, M. Ishida, T. Nakajima, Y. Honda, O. Kitao, H. Nakai, T. Vreven, K. Throssell, J. A. Montgomery, Jr., J. E. Peralta, F. Ogliaro, M. J. Bearpark, J. J. Heyd, E. N. Brothers, K. N. Kudin, V. N. Staroverov, T. A. Keith, R. Kobayashi, J. Normand, K. Raghavachari, A. P. Rendell, J. C. Burant, S. S. Iyengar, J. Tomasi, M. Cossi, J. M. Millam, M. Klene, C. Adamo, R. Cammi, J. W. Ochterski, R. L. Martin, K. Morokuma, O. Farkas, J. B. Foresman, and D. J. Fox, Gaussian, Inc., Wallingford CT, 2016.

5.3. Reversible Stabilization of Keteniminium Ion 2 with Collidine

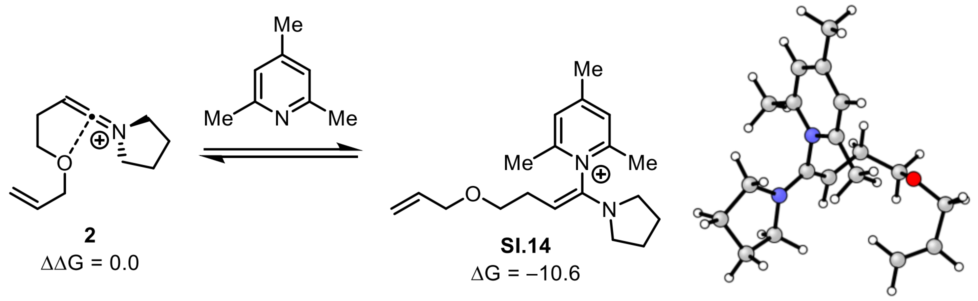

Computed without triflate counterion; energies vs infinitely-separated **2** and collidine. Additional base-stabilized structures computed for methyl olefin congener; see below.

5.4. Ynamide Enamine Triflate Model Comparison

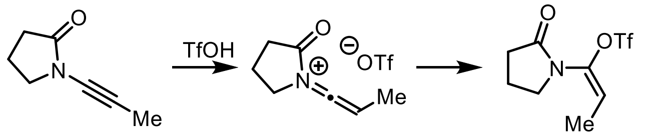

ynamide model — **SI.15** 2-amide-keteniminium — **SI.16** 6-amide-enamine

	**SI.15** **2-amide** model (ΔΔG)	**SI.16** **6-amide-enamine** model (ΔΔG)
ωB97X-D	8.7	0.0
MP2//ωB97X-D	10.8	0.0

SI.15

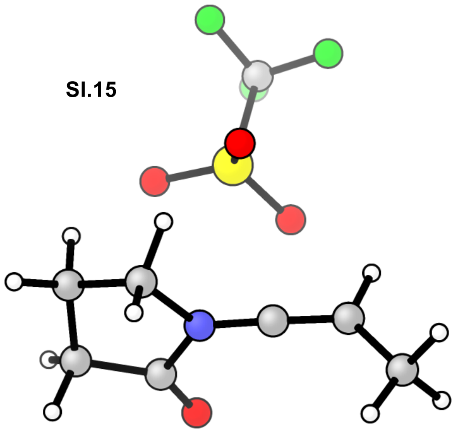

SI.16

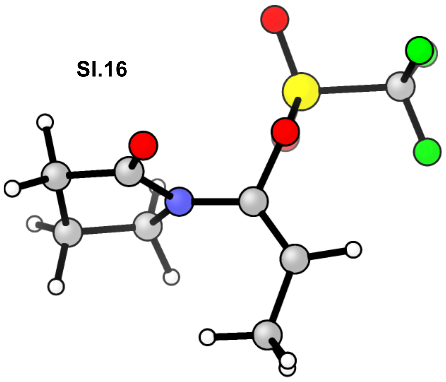

Above: Ketenimium ions produced through protonation of ynamides are higher in energy than the corresponding amide enamine triflate. **Below:** In contrast, keteniminium ions produced through ionization of enamine triflates are *lower* in energy than the corresponding enamine triflate. Coordinates provided below for ωB97X-D optimizations.

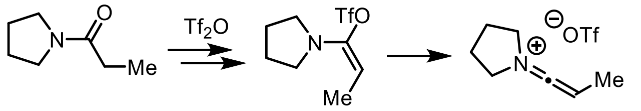

1-model amide — **SI.17** 6-*E*-*O*-enamine model — **SI.18** 2-model

	**SI.17** **6-*E*-*O*-enamine** model (ΔΔG)	**SI.18** **2** model (ΔΔG)
ωB97X-D	0.0	−10.0
M06-2X-D3	0.0	−10.5
B3LYP-D3	0.0	−13.5
MP2//ωB97X-D	0.0	−7.5

SI.17

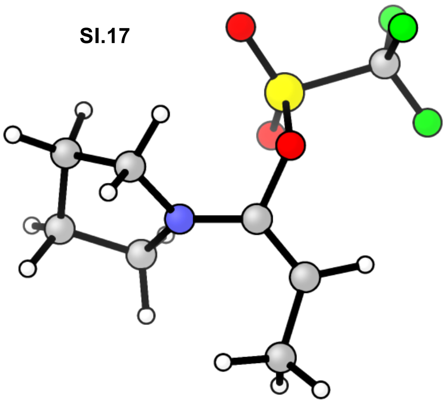

SI.18

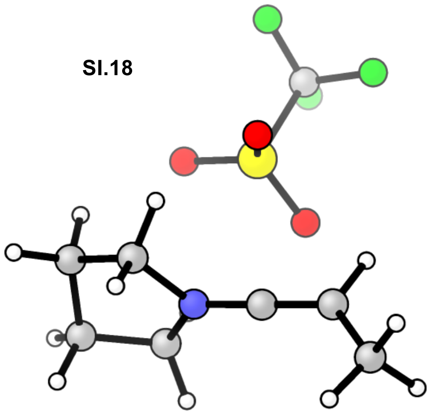

Anion positions located through metadynamics using xTB/CREST. Geometry optimizations utilizing *functional* indicated in the above tables as *functional*/def2-TZVPP/SMD(CH2Cl2)//*functional*/def2-SVP/SMD(CH2Cl2), QH corrections at 393.15 K, energies in kcal/mol.

5.5. Potential Energy Surface: Generation of Allyloxonium Ion 9 from Amide 1

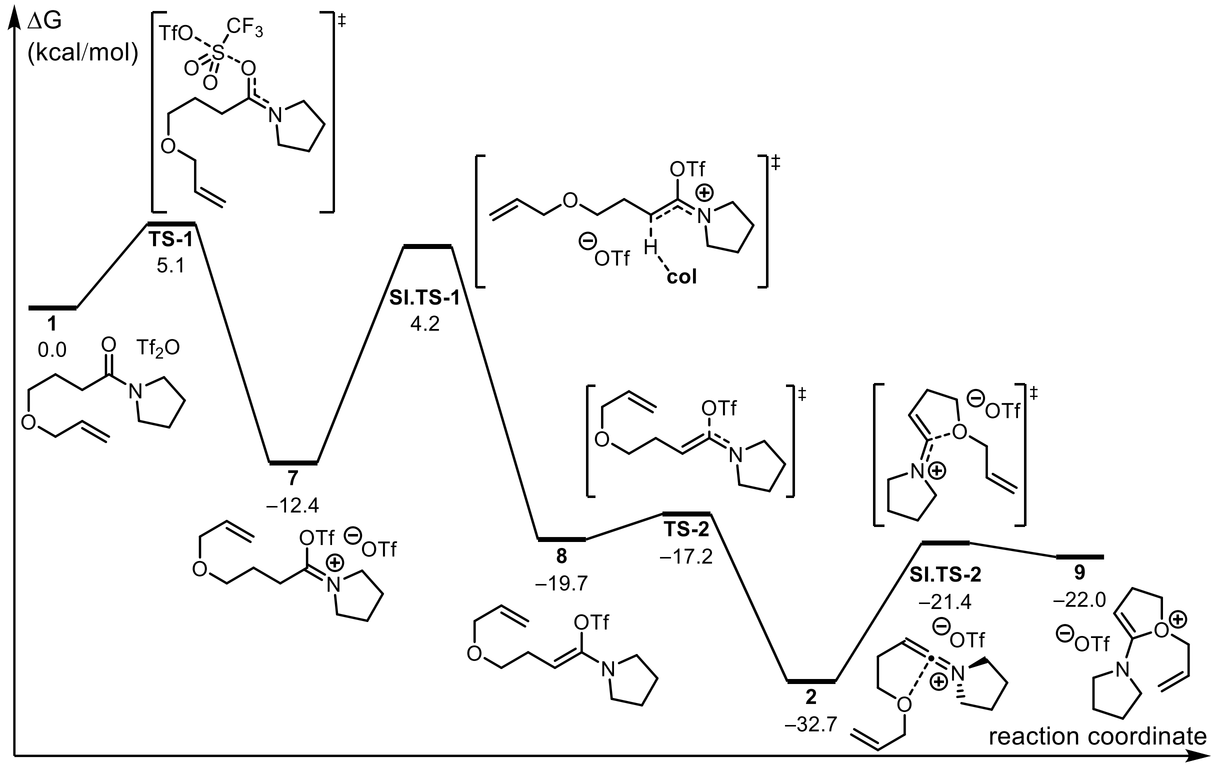

Temperature corrected to 373.15 K (120 °C) in accordance with experiment; **col** = 2,4,6-trimethylpyridine (collidine).

5.6. Potential Energy Surface: Methyl Olefin Congener (10)

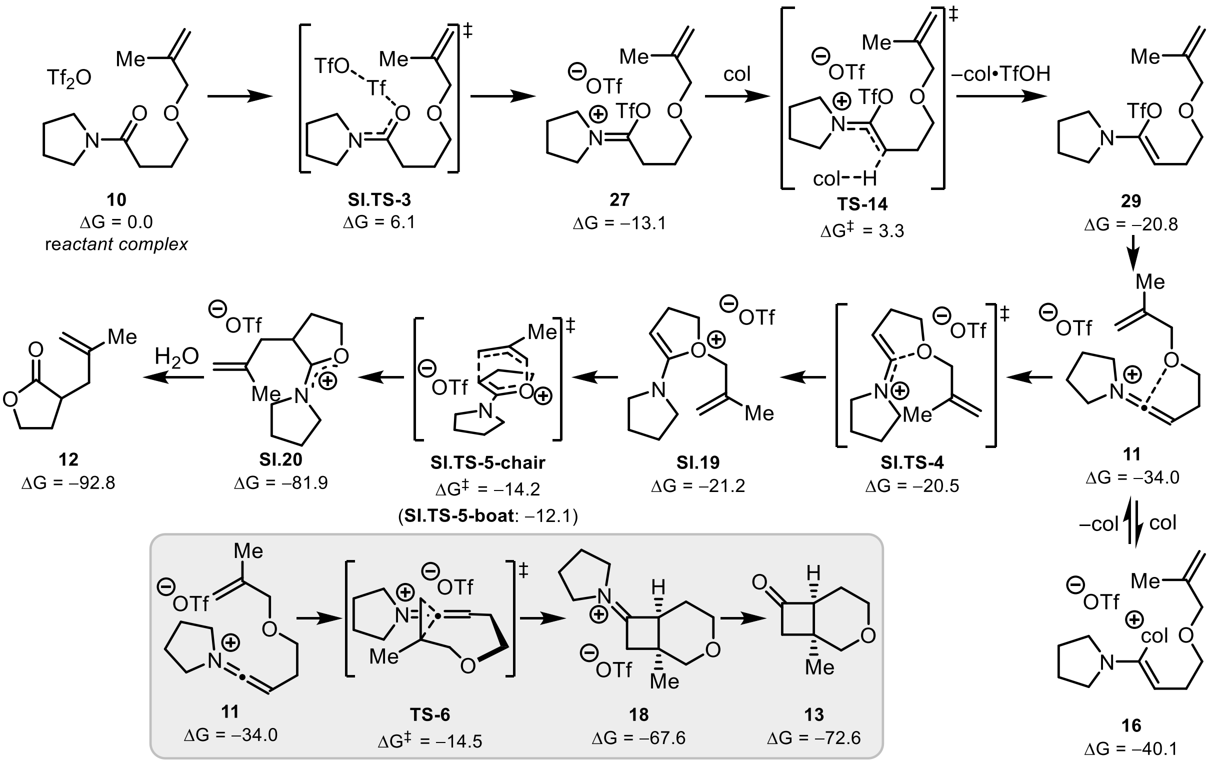

Temperature corrected to 373.15 K (120 °C) for comparison to allyl substrate **1**.

5.7. Forward Intrinsic Reaction Coordinate for TS-6

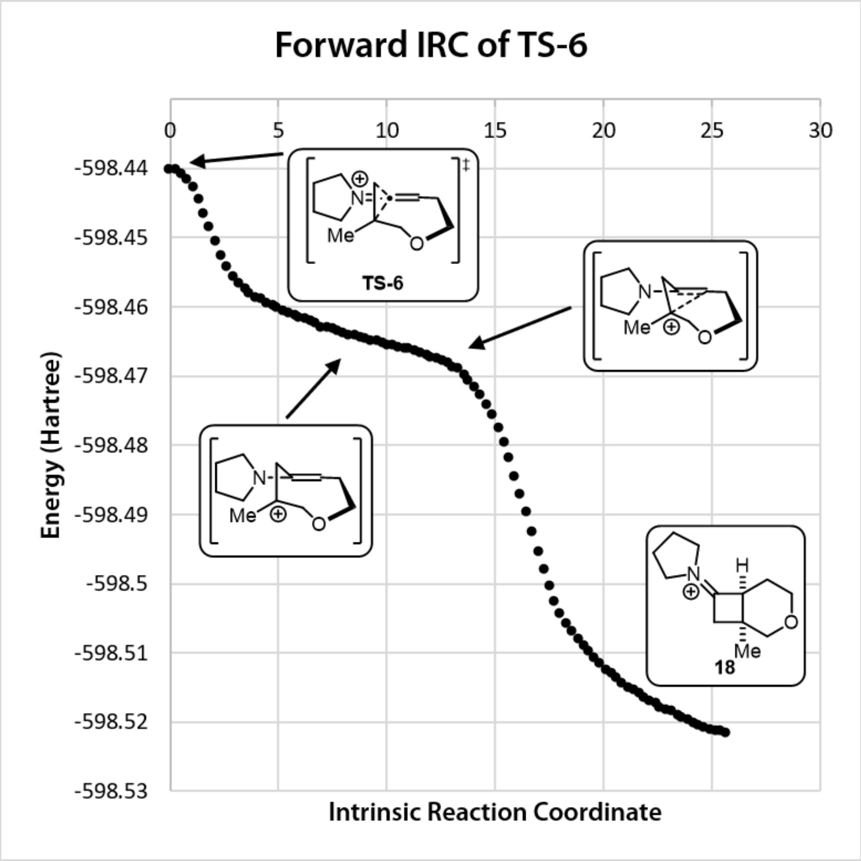

IRC of **TS-6** from **11** to **18**, exhibiting cationic entropic intermediate as shoulder along reaction coordinate.

5.8. Aryl Olefin Product Distribution Calculations

4-Methoxyphenyl substrate (30a)

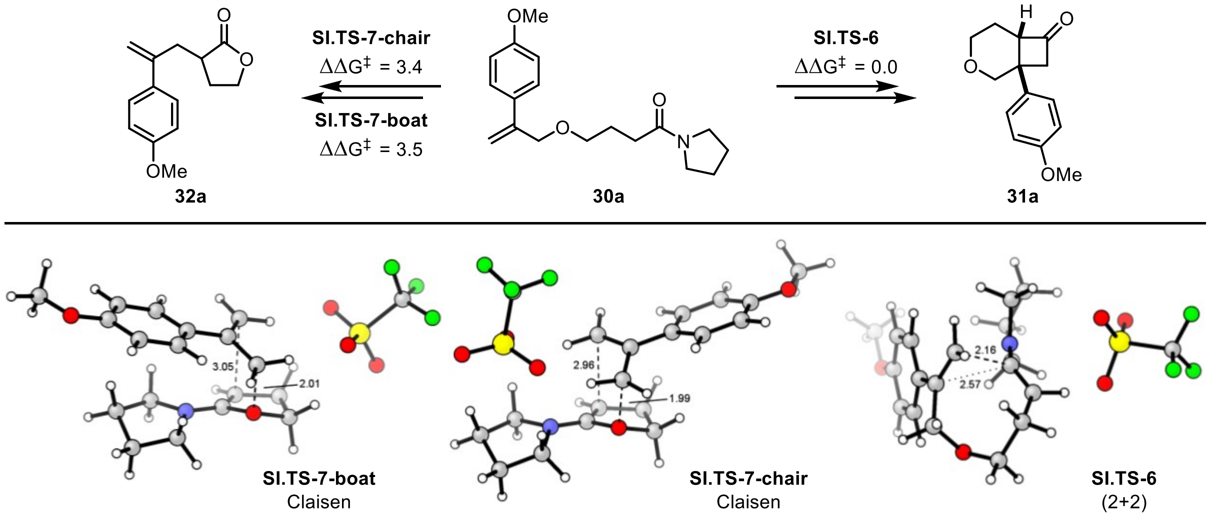

Energies in kcal/mol referenced against (2+2) transition structure **SI.TS-6** at 0 °C (273.15 K) in accordance with reaction conditions. Given the energy difference between (2+2) and Claisen TSs (≥ 3.4 kcal/mol), products derived from the (2+2) TS (e.g., **31a**) would be expected to yield a Boltzmann population of >99%. This is consistent with experiment wherein the lactone product **32a** was not observed.

Phenyl substrate (30c)

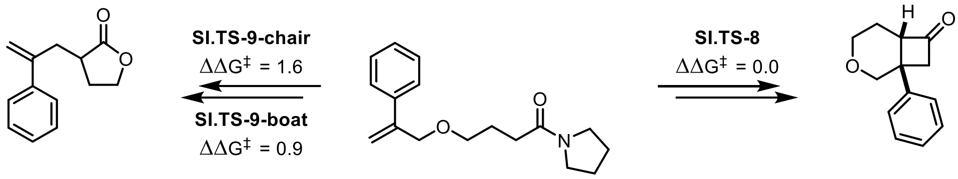

32c **30c** **31c**

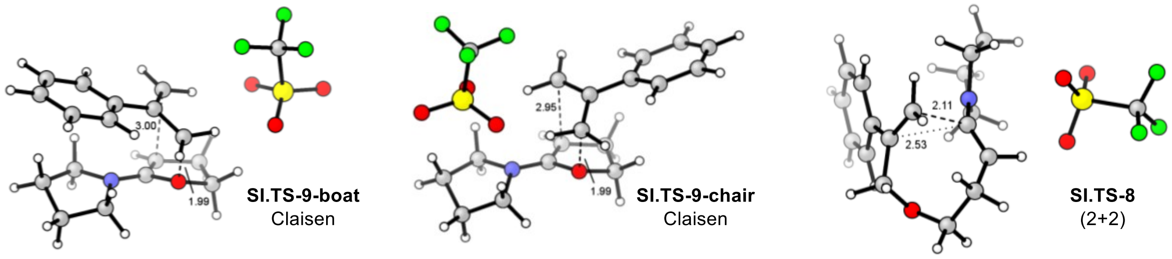

Energies in kcal/mol referenced against (2+2) transition structure **SI-TS-8** at 0 °C (273.15 K) in accordance with reaction conditions. The energy difference between Claisen and (2+2) TSs are considerably smaller than in the 4-methoxyphenyl case above. Boltzmann distributions computed at 0 °C for these barriers predicts a yield of lactone product **32c** of ~21%, in agreement with the observed NMR yield of 20%. The remainder of the mass balance (79%) is predicted to be derived from (2+2) products (i.e., monomer **31c** and the corresponding dimer); NMR yield for monomer **31c** is 58%.

5.9. Stabilized Cationic Intermediates of Aryl Olefins 30a, 30c (2+2) Reactions

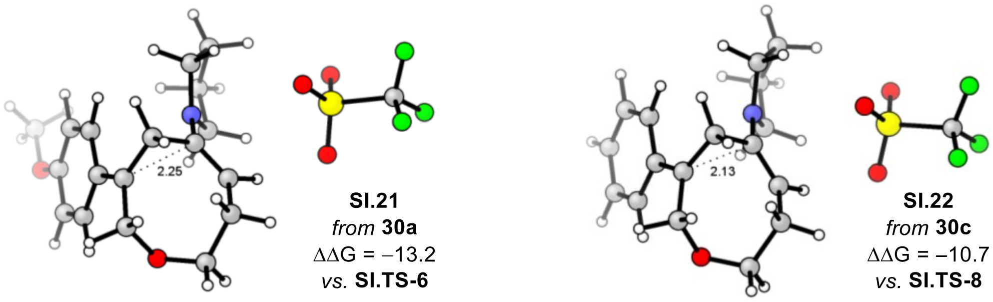

Energies in kcal/mol referenced against (2+2) transition structures **SI.TS-6** (for **SI.21**) and **SI.TS-8** (for **SI.22**) at 0 °C (273.15 K) in accordance with reaction conditions.

5.10. Diastereomeric Transition Structures of Methoxymethylpyrrolidine 34

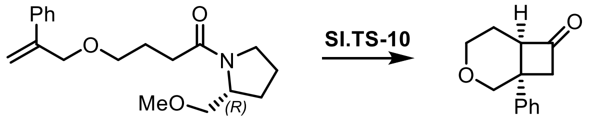

34 **31c**

e.r. 58:42

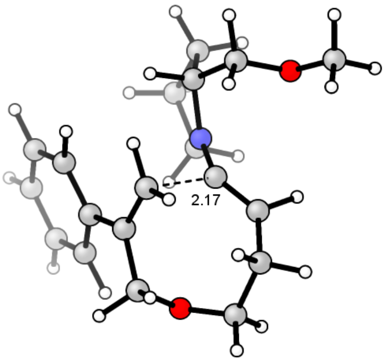

SI.TS-10-diastereomer1

minor product

$\Delta\Delta G^{\ddagger}$ = 0.5 kcal/mol

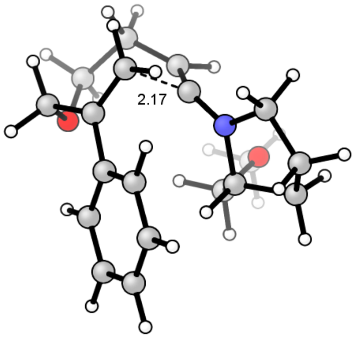

SI.TS-10-diastereomer2

major product

$\Delta\Delta G^{\ddagger}$ = 0.0 kcal/mol

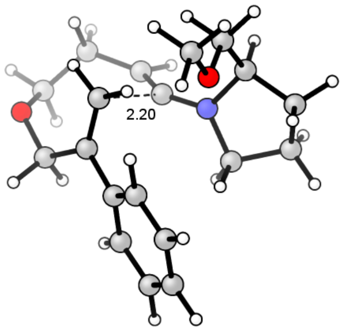

SI.TS-10-diastereomer3

major product

$\Delta\Delta G^{\ddagger}$ = 1.8 kcal/mol

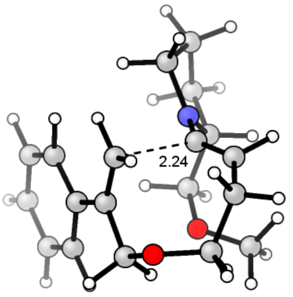

SI.TS-10-diastereomer4

minor product

$\Delta\Delta G^{\ddagger}$ = 2.8 kcal/mol

Predicted Boltzmann population at 0 °C: 71:29 e.r.

Energies in kcal/mol referenced against lowest-energy diastereomeric (2+2) transition structure **SI.TS-10-diastereomer2** at 0 °C (273.15 K) in accordance with reaction conditions. Structures optimized without triflate counterion. Diastereomeric TSs 2 and 3 produce the same product, expected to be major. Diastereomeric TSs 1 and 4 produce the same product, expected to be minor. Experimental e.r. suggests net free energy difference of ~0.2 kcal/mol at 0 °C between major and minor TSs, indicating a discrepancy of ~0.3 kcal/mol with the computed predictions.

5.11. Representative Diastereomeric TS of Imidazolidinone 35

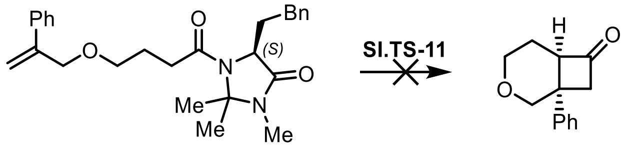

35 31c

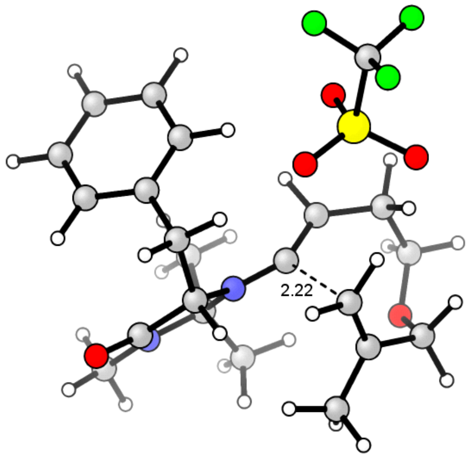

SI.TS-11-diastereomer1 (Me olefin congener)
Lowest energy diastereomeric TS

Representative MacMillan auxiliary (2+2) transition structure at 0 °C (273.15 K). Computationally-predicted stereoselectivity for methyl olefin congener considering all four diastereomeric TSs at 0 °C would be ~99:1 e.r. Neither (2+2) product nor allyl lactone product were observed experimentally, refinements to DFT model were not pursued.

5.12. Computed Structures and Energies

Structures below were supplied to the publisher as xyz files. Energies below are reported directly from the output file, without quasiharmonic and temperature corrections, at the optimization level of theory, ωB97X-D/def2-SVP/SMD(CH2Cl2). Individual energies represent energies of the indicated structure or complex without balancing molecules (e.g., collidine/collidinium triflate, water).

1

ZPE	0.349959
DE	0.380695
DH	0.381640
DG	0.283539
E	-2481.398588
H	-2481.016948
Gibbs free energy	-2481.115049

Cartesian coordinates

C	-3.064746	1.405737	-1.773461
N	-3.046081	1.567547	-0.318816
C	-2.177637	0.875546	0.436908
O	-1.347388	0.123671	-0.086937
S	1.229271	-0.153870	0.498689
O	0.780714	-0.438316	1.829292
O	1.227626	1.125276	-0.147870
C	0.724317	-1.564354	-0.693416
F	0.316720	-1.045271	-1.817869
F	1.779700	-2.324605	-0.919182

F	-0.200954	-2.285370	-0.118611
C	-2.195773	1.048678	1.942518
H	-2.925905	1.801097	2.267673
H	-1.195334	1.428280	2.202471
C	-2.430581	-0.272712	2.677087
C	-3.876830	-0.747977	2.601661
O	-4.373393	-0.850155	1.288715
C	-3.907603	-1.963976	0.563608
C	-4.570101	-1.978850	-0.778170
C	-3.922504	-2.147133	-1.931089
H	-4.454716	-2.181427	-2.886092
H	-2.833730	-2.262199	-1.961190
H	-5.660305	-1.858027	-0.768791
H	-4.161296	-2.894955	1.113006
H	-2.810779	-1.938517	0.439988
H	-3.982089	-1.716477	3.128669
H	-4.529679	-0.022464	3.112955
H	-1.741807	-1.031747	2.276352
H	-2.172561	-0.149289	3.740634
C	-4.209805	2.315137	0.166875
C	-4.852223	2.847902	-1.113904
H	-5.934272	3.000234	-0.999663
C	-4.489644	1.790213	-2.156388
H	-4.565435	2.152332	-3.190902
H	-5.148261	0.912833	-2.051420
H	-4.401782	3.815797	-1.386671
S	4.042810	-0.347947	-0.466304
O	3.453971	0.004001	-1.732656
O	4.995111	-1.410866	-0.309020
C	4.798905	1.185023	0.279765
F	5.293951	0.890072	1.461093
F	5.759913	1.582096	-0.527721
F	3.887226	2.123739	0.392680
O	2.879266	-0.607272	0.638071
H	-2.320863	2.075944	-2.238452
H	-2.799256	0.374651	-2.039624
H	-3.916021	3.124111	0.850752
H	-4.887534	1.630016	0.701646

TS-1

ZPE	0.349903
DE	0.379942
DH	0.380886
DG	0.283993
E	-2481.392478
H	-2481.011592
Gibbs free energy	-2481.108485
Imaginary frequency	-99.956

Cartesian coordinates

C	-3.564737	-1.737263	-0.018799
N	-3.488980	-0.527278	-0.852170
C	-2.348663	0.093606	-1.055071
O	-1.317518	-0.448974	-0.530927
S	0.569530	-0.001265	-0.850596
O	0.484533	1.254175	-0.142413
O	0.517504	-0.247118	-2.267791
C	0.722699	-1.619250	0.204836
F	0.070982	-2.554865	-0.442776
F	1.958751	-2.000409	0.363793
F	0.179677	-1.374159	1.371252
C	-2.278726	1.343248	-1.896271
H	-2.790331	1.133347	-2.848190
H	-1.228779	1.528930	-2.143643
C	-2.880682	2.603340	-1.253605
C	-2.399893	2.844382	0.163105
O	-2.888992	1.794953	0.963536
C	-2.206267	1.604354	2.179129
C	-2.975195	0.624177	3.008710
C	-2.449669	-0.453360	3.591123
H	-3.057177	-1.118060	4.212131
H	-1.390417	-0.705488	3.478727
H	-4.040617	0.853206	3.134528
H	-2.133501	2.566746	2.725698
H	-1.175650	1.247565	1.996145
H	-1.296255	2.871905	0.192686
H	-2.770406	3.819503	0.533281
H	-2.602208	3.462126	-1.881843

H	-3.979818	2.557853	-1.254357
C	-4.816971	-0.070188	-1.273835
C	-5.681668	-1.308855	-1.070418
H	-6.742568	-1.052956	-0.950109
C	-5.064516	-1.949525	0.173274
H	-5.318190	-3.011524	0.287941
H	-5.402926	-1.419310	1.077276
H	-5.586371	-1.981288	-1.937647
S	3.402259	0.749746	0.159611
O	2.916907	0.475945	1.501410
O	3.765306	2.102883	-0.208229
C	4.957934	-0.242608	-0.038533
F	5.429171	-0.122355	-1.268500
F	5.860507	0.217065	0.816003
F	4.737351	-1.520915	0.217775
O	2.550200	0.052562	-0.921313
H	-3.084974	-2.571450	-0.554107
H	-3.018996	-1.576348	0.920904
H	-4.799595	0.294244	-2.309317
H	-5.140327	0.749244	-0.613120

2

ZPE	0.306029
DE	0.328863
DH	0.329807
DG	0.248526
E	-1520.057943
H	-1519.728136
Gibbs free energy	-1519.809417

Cartesian coordinates

C	0.196936	2.352711	1.208124
N	0.664552	1.272920	0.297245
C	0.985173	0.121223	0.666713
C	1.245926	-1.094838	1.036530
C	2.598750	-1.647322	1.375084
H	2.836591	-2.420923	0.625908
H	2.534378	-2.173164	2.340008
C	3.689342	-0.585280	1.449333

O	3.685865	0.298530	0.353202
C	4.014882	-0.267945	-0.901048
C	5.390960	-0.868320	-0.936381
C	5.657073	-2.107360	-1.349076
H	6.680752	-2.490906	-1.388723
H	4.858824	-2.783339	-1.676123
H	6.204921	-0.209677	-0.608335
H	3.263036	-1.015295	-1.217793
H	3.961125	0.568829	-1.615194
H	4.668039	-1.083665	1.563320
H	3.531135	0.046488	2.336267
H	0.356157	-1.736739	1.070169
C	0.677770	1.723305	-1.121570
C	-0.050752	3.060182	-1.067119
H	-1.131579	2.883525	-1.160693
C	0.272938	3.594070	0.330528
H	1.289107	4.017439	0.359707
H	-0.431172	4.365224	0.668957
H	0.276484	3.730255	-1.872512
H	1.732250	1.825961	-1.416455
H	0.183514	0.956569	-1.730646
H	-0.836771	2.099562	1.483510
H	0.828670	2.373865	2.104038
C	-3.866272	-1.115609	-0.208971
F	-4.401915	-0.936140	-1.414122
F	-4.754463	-0.721424	0.700446
F	-3.654991	-2.419493	-0.041307
S	-2.285710	-0.165409	-0.041957
O	-2.702302	1.227165	-0.252684
O	-1.440702	-0.737180	-1.101165
O	-1.850361	-0.477678	1.329041

3

ZPE	0.310496
DE	0.331914
DH	0.332858
DG	0.257148
E	-1520.154402
H	-1519.821544
Gibbs free energy	-1519.897254

Cartesian coordinates

C	1.165912	2.007302	-1.161414
N	1.437660	1.422841	0.162519
C	1.558607	0.156897	0.408176
O	1.756713	-0.225085	1.632780
C	1.767632	-1.673611	1.726893
H	1.095698	-1.940393	2.550680
C	1.301388	-2.167309	0.360738
H	1.856021	-3.059665	0.042039
H	0.229757	-2.397574	0.388300
C	1.537595	-0.972119	-0.577510
H	0.679339	-0.823126	-1.247094
C	2.831963	-1.039856	-1.416565
C	4.085098	-1.163733	-0.599224
C	4.864952	-2.244889	-0.560620
H	5.764411	-2.277688	0.060956
H	4.636720	-3.134742	-1.157688
H	4.355209	-0.293027	0.013221
H	2.733050	-1.899967	-2.095551
H	2.887861	-0.137561	-2.047315
H	2.795228	-1.969777	1.977390
O	-1.073402	-0.129861	0.979022
S	-1.964201	0.254228	-0.129497
O	-1.394474	0.059806	-1.474074
O	-2.697077	1.507408	0.064235
C	-3.282084	-1.044528	-0.054415
F	-3.919537	-1.008490	1.112757
F	-4.178065	-0.869573	-1.021867
F	-2.754124	-2.260661	-0.197329
C	1.308703	2.418031	1.243529
C	1.064397	3.727129	0.496559
H	0.442057	4.415147	1.083332
C	0.401992	3.277244	-0.806365
H	0.455509	4.026767	-1.606506
H	-0.655474	3.023344	-0.632307
H	2.021669	4.228707	0.285460
H	2.209410	2.420367	1.871658
H	0.446397	2.121672	1.860301

H	0.581871	1.307786	-1.771042
H	2.123388	2.229222	-1.657964

4

ZPE	0.161335
DE	0.169955
DH	0.170899
DG	0.127004
E	-422.778286
H	-422.607387
Gibbs free energy	-422.651282

Cartesian coordinates

C	-1.215016	0.844715	-0.071277
C	-0.002358	-0.054908	-0.253019
C	-0.542277	-1.414739	0.187154
H	-0.062063	-2.266729	-0.310789
H	-0.418525	-1.533268	1.275792
C	-2.020039	-1.295741	-0.168620
H	-2.220025	-1.590729	-1.211022
H	-2.688479	-1.858963	0.494561
O	-2.327799	0.098659	-0.026255
C	1.238058	0.475684	0.464388
H	1.429273	1.506088	0.127725
H	1.019774	0.522892	1.546010
C	2.450205	-0.380220	0.237614
H	2.388660	-1.412905	0.604631
C	3.565406	0.022724	-0.372719
H	3.670566	1.044655	-0.754175
H	4.416465	-0.650769	-0.510542
H	0.190772	-0.069931	-1.342013
O	-1.243489	2.041912	0.018593

5

ZPE	0.312397
DE	0.332550
DH	0.333494
DG	0.261166
E	-1520.139250

H -1519.805756

Gibbs free energy -1519.878084

Cartesian coordinates

C	0.491815	2.701802	1.281231
N	1.007327	1.883125	0.162969
C	1.561487	0.742554	0.319375
C	1.820160	-0.089689	1.525224
H	1.092254	-0.014237	2.341896
H	2.843598	0.078297	1.899361
C	1.750167	-1.305134	0.557617
C	2.753137	-2.434738	0.688500
O	4.061037	-2.037752	0.358500
C	4.146463	-1.546207	-0.958409
C	3.436548	-0.206516	-1.085332
H	3.489309	0.163246	-2.119197
H	3.972706	0.520936	-0.455176
C	1.960047	-0.324242	-0.647120
H	1.274392	-0.451804	-1.494136
H	5.214344	-1.433988	-1.194180
H	3.718818	-2.281200	-1.669498
H	2.776784	-2.807938	1.723319
H	2.437049	-3.272100	0.035220
H	0.723014	-1.689693	0.539092
O	-1.224002	-0.074276	1.195163
S	-1.959187	-0.003410	-0.079084
O	-1.125408	-0.140708	-1.283634
O	-2.989013	1.037332	-0.144637
C	-2.926926	-1.582380	-0.072424
F	-2.111000	-2.634507	0.001530
F	-3.753971	-1.630292	0.968717
F	-3.651092	-1.701916	-1.182186
C	0.673637	2.480114	-1.145838
C	-0.409238	3.491279	-0.790693
H	-0.478447	4.296581	-1.533172
C	0.008574	3.977019	0.598775
H	-0.811981	4.445919	1.156907
H	0.831117	4.705122	0.520925
H	-1.381180	2.976563	-0.737159

H	0.337906	1.689218	-1.826890
H	1.579273	2.963004	-1.543458
H	1.281331	2.852090	2.028648
H	-0.336167	2.133177	1.730940

6

ZPE	0.162878
DE	0.170280
DH	0.171224
DG	0.130877
E	-422.754559
H	-422.583335
Gibbs free energy	-422.623682

Cartesian coordinates

C	-1.537051	-0.352259	-0.115344
O	-2.060842	-1.141480	-0.852964
C	-1.631855	1.158756	0.062717
H	-2.375272	1.409549	0.835256
H	-1.820441	1.762483	-0.837604
C	-0.190265	1.084721	0.634228
C	0.877980	1.332219	-0.434305
O	2.014007	0.523583	-0.259412
C	1.717934	-0.840761	-0.451694
C	0.803944	-1.374352	0.646506
H	1.380143	-1.456169	1.581403
H	0.450826	-2.385536	0.388999
C	-0.378128	-0.444678	0.871344
H	-0.827989	-0.604927	1.865931
H	1.247601	-0.984755	-1.445926
H	2.675053	-1.381679	-0.455237
H	0.449445	1.147969	-1.442106
H	1.214270	2.379007	-0.411090
H	0.005691	1.675356	1.538670

7

ZPE	0.351338
DE	0.381913
DH	0.382857

DG	0.284748
E	-2481.404145
H	-2481.021288
Gibbs free energy	-2481.119397

Cartesian coordinates

C	-0.052671	-1.314529	1.070347
N	1.413450	-1.441325	1.234457
C	2.268286	-0.579184	0.811695
O	1.718651	0.466950	0.165249
S	2.250300	2.035629	0.098525
O	2.586318	2.311211	-1.269768
O	3.133201	2.299142	1.202836
O	-3.004315	-1.584228	-0.857737
S	-3.138214	-0.437681	0.052261
O	-3.213455	-0.780339	1.479558
O	-2.291483	0.719750	-0.266705
C	-4.844138	0.187135	-0.315099
F	-5.760107	-0.750055	-0.073632
F	-4.956050	0.548420	-1.592881
F	-5.137045	1.247995	0.436705
C	0.574644	2.798058	0.467732
F	0.039784	2.186344	1.494390
F	0.806860	4.059642	0.757403
F	-0.176445	2.700443	-0.593776
C	3.745255	-0.668787	0.983774
H	3.986525	-1.615343	1.479906
H	4.029340	0.138689	1.677487
C	4.533349	-0.546743	-0.326068
C	4.281096	-1.741507	-1.237629
O	2.904741	-1.965629	-1.430931
C	2.315267	-1.251109	-2.503007
C	0.851158	-1.558667	-2.519312
C	-0.109002	-0.638532	-2.432721
H	-1.167965	-0.908148	-2.405428
H	0.133729	0.425373	-2.345884
H	0.591986	-2.622338	-2.586231
H	2.789213	-1.565955	-3.453235
H	2.476063	-0.162260	-2.398103

H	4.800997	-1.604014	-2.201918
H	4.684449	-2.652252	-0.767458
H	4.304025	0.395721	-0.846817
H	5.602520	-0.499381	-0.074816
C	1.776288	-2.762635	1.799460
C	0.427724	-3.394990	2.131471
H	0.474784	-4.488958	2.057920
C	-0.527196	-2.756459	1.126126
H	-1.583315	-2.802568	1.413810
H	-0.418564	-3.219789	0.133441
H	0.131795	-3.133052	3.158650
H	2.431714	-2.634949	2.669652
H	2.310684	-3.316385	1.013363
H	-0.287454	-0.794209	0.136678
H	-0.440647	-0.720321	1.910712

8

ZPE	0.307406
DE	0.329295
DH	0.330239
DG	0.252815
E	-1520.062171
H	-1519.731931
Gibbs free energy	-1519.809356

Cartesian coordinates

C	-3.058550	-0.413686	-0.153452
N	-1.945074	-1.342594	0.042561
C	-0.849393	-1.088401	0.806084
C	-0.045577	-1.967455	1.431241
C	1.299823	-1.620184	1.993421
H	1.328174	-0.561883	2.295242
H	1.512168	-2.221100	2.891234
C	2.416302	-1.862394	0.980462
O	2.068537	-1.208932	-0.211574
C	3.072939	-1.149851	-1.189907
C	4.188985	-0.209729	-0.826302
C	5.485618	-0.511963	-0.886964
H	6.257979	0.222860	-0.640877

H	5.826002	-1.507296	-1.195219
H	3.863283	0.787047	-0.505436
H	3.474578	-2.159082	-1.409444
H	2.573159	-0.784669	-2.101623
H	2.546042	-2.948151	0.793613
H	3.374519	-1.482201	1.382401
H	-0.331968	-3.020510	1.389194
O	-0.596396	0.291589	0.965441
S	0.237573	1.187595	-0.081237
O	0.092817	0.707512	-1.433524
O	1.505811	1.567652	0.490268
C	-0.811102	2.715939	0.098801
F	-2.016368	2.531184	-0.401936
F	-0.204949	3.681315	-0.568470
F	-0.906770	3.049733	1.369902
C	-2.278151	-2.701922	-0.349603
C	-3.522370	-2.515950	-1.212652
H	-4.143288	-3.421258	-1.254217
C	-4.218853	-1.331465	-0.540872
H	-4.949047	-0.827397	-1.188857
H	-4.746501	-1.671790	0.364386
H	-3.229924	-2.253610	-2.241938
H	-1.434242	-3.168810	-0.882962
H	-2.502427	-3.330625	0.534912
H	-3.267182	0.170530	0.755873
H	-2.828367	0.294288	-0.966330

TS-2

ZPE	0.306268
DE	0.327838
DH	0.328782
DG	0.253502
E	-1520.050023
H	-1519.721241
Gibbs free energy	-1519.796521
Imaginary frequency	-270.433

Cartesian coordinates

C	-2.981966	-1.548529	0.596309

N	-1.638544	-1.918711	0.109228
C	-0.571330	-1.756956	0.811930
C	0.437996	-2.325280	1.431120
C	1.692554	-1.695981	1.948656
H	1.475599	-0.678567	2.296433
H	2.083744	-2.284594	2.790233
C	2.761298	-1.625469	0.861257
O	2.211701	-0.968560	-0.245530
C	3.120950	-0.594992	-1.249432
C	4.026116	0.527072	-0.823870
C	5.352910	0.520079	-0.950822
H	5.961554	1.379396	-0.653541
H	5.882898	-0.344146	-1.367946
H	3.502554	1.390554	-0.396623
H	3.714087	-1.465564	-1.593444
H	2.496401	-0.256702	-2.091240
H	3.103210	-2.641583	0.577333
H	3.638885	-1.084653	1.261333
H	0.335680	-3.416563	1.476613
O	-0.379972	0.126018	1.125089
S	0.028730	1.133115	0.077086
O	-0.101746	0.661544	-1.297486
O	1.211708	1.899416	0.441173
C	-1.353043	2.356461	0.257791
F	-2.524951	1.791535	-0.028856
F	-1.167667	3.372779	-0.575253
F	-1.409666	2.828854	1.495696
C	-1.618045	-2.093728	-1.360544
C	-2.891635	-1.384547	-1.799295
H	-3.255488	-1.743971	-2.771103
C	-3.860301	-1.665764	-0.647932
H	-4.706407	-0.966554	-0.613857
H	-4.265722	-2.686156	-0.733049
H	-2.691536	-0.305276	-1.875976
H	-0.700997	-1.654519	-1.768854
H	-1.648875	-3.171801	-1.586386
H	-3.282134	-2.212297	1.418946
H	-2.939331	-0.515925	0.971874

9

ZPE	0.308461
DE	0.330209
DH	0.331153
DG	0.255051
E	-1520.051630
H	-1519.720477
Gibbs free energy	-1519.796579

Cartesian coordinates

C	3.714059	-1.656084	0.094198
N	2.407842	-1.004738	-0.017611
C	2.170315	0.105801	0.704841
C	2.724487	0.732464	1.744574
C	2.077254	2.066112	1.995517
H	2.692687	2.906688	1.634837
C	0.771648	1.935550	1.229773
H	-0.043213	1.494266	1.812992
H	0.427920	2.826599	0.697435
O	1.060860	0.902467	0.195881
C	1.109778	1.329906	-1.232669
H	1.205635	0.387904	-1.782272
H	0.114107	1.754787	-1.400320
C	2.235402	2.272058	-1.476128
C	2.039670	3.543842	-1.825260
H	2.879869	4.212882	-2.030167
H	1.031022	3.955686	-1.935923
H	3.248806	1.867523	-1.377435
H	1.870721	2.231981	3.061983
H	3.535689	0.309055	2.333683
C	1.332506	-1.982851	-0.300386
H	0.515682	-1.885870	0.428439
H	0.896554	-1.792316	-1.293200
C	2.055046	-3.328802	-0.249005
H	2.029989	-3.731709	0.775892
C	3.494040	-2.977658	-0.631917
H	4.224941	-3.741695	-0.334256
H	3.576987	-2.828916	-1.720505
H	1.594471	-4.071025	-0.915152
H	4.499092	-1.025578	-0.350207

H	3.976673	-1.838109	1.152991
C	-3.744180	-0.396404	0.033512
S	-1.939149	0.015026	-0.047342
O	-1.493282	-0.679183	-1.260997
O	-1.424880	-0.523764	1.221649
O	-1.940309	1.484428	-0.140175
F	-4.380267	0.031948	-1.055380
F	-3.926739	-1.712547	0.124995
F	-4.315816	0.172310	1.093582

TS-3-chair

ZPE	0.306726
DE	0.327895
DH	0.328839
DG	0.252957
E	-1520.038061
H	-1519.709222
Gibbs free energy	-1519.785104
Imaginary frequency	-366.719

Cartesian coordinates

C	1.480991	2.086060	-1.178640
N	1.624690	1.223453	-0.005052
C	1.592597	-0.109045	-0.174828
C	1.557320	-0.900652	-1.279023
C	1.520960	-2.342979	-0.859977
H	2.414599	-2.912100	-1.162204
C	1.380558	-2.217705	0.654986
H	0.333629	-2.264394	0.977044
H	2.006014	-2.890381	1.252494
O	1.803932	-0.846549	0.965425
C	3.664177	-0.736326	1.398486
H	3.622887	0.347317	1.534276
H	3.607188	-1.328343	2.314556
C	4.297975	-1.262492	0.246398
C	4.314445	-0.529018	-0.899770
H	4.673660	-0.952568	-1.841083
H	4.073308	0.537101	-0.889181
H	4.574158	-2.320034	0.231196

H	0.631891	-2.840432	-1.273045
H	1.328043	-0.538295	-2.278719
C	1.114059	1.866593	1.218282
H	0.434376	1.184410	1.744572
H	1.950656	2.134428	1.886420
C	0.402361	3.108405	0.685567
H	-0.624783	2.831334	0.404716
C	1.200354	3.457803	-0.571656
H	0.654712	4.110317	-1.266904
H	2.144328	3.959745	-0.303065
H	0.365851	3.921501	1.423784
H	2.390171	2.053576	-1.800119
H	0.623375	1.745086	-1.782473
C	-3.618673	-0.250464	0.114159
S	-1.792600	-0.379846	-0.174895
O	-1.251169	-0.372650	1.194472
O	-1.509049	0.822055	-0.970033
O	-1.659288	-1.657582	-0.884805
F	-4.062019	-1.274849	0.841922
F	-3.920258	0.873695	0.763426
F	-4.285883	-0.248643	-1.039009

TS-3-boat

ZPE	0.306217
DE	0.327613
DH	0.328557
DG	0.252950
E	-1520.037255
H	-1519.708698
Gibbs free energy	-1519.784305
Imaginary frequency	-259.153

Cartesian coordinates

C	-4.470434	-0.055468	1.180861
N	-3.620197	0.143117	0.016564
C	-2.451345	0.794521	0.120200
C	-1.826711	1.405619	1.156762
C	-0.486378	1.912264	0.697649
H	0.363029	1.373263	1.144403

C	-0.580098	1.696948	-0.811752
H	-0.915741	2.597048	-1.344371
H	0.324342	1.299777	-1.284173
O	-1.646134	0.705606	-0.991166
C	-0.936392	-1.174636	-1.080381
H	-1.408264	-1.378565	-2.043792
H	0.106434	-0.833455	-1.104514
C	-1.430682	-1.786820	0.095391
C	-0.846583	-1.490615	1.286765
H	-1.254806	-1.872934	2.226534
H	0.094744	-0.929427	1.329539
H	-0.343243	2.979937	0.920736
H	-2.292535	1.623201	2.115735
C	-4.299728	-0.151269	-1.247301
H	-4.157590	0.662419	-1.973352
H	-3.904943	-1.079198	-1.696278
C	-5.757560	-0.322663	-0.818409
H	-6.265498	0.654711	-0.822715
C	-5.647530	-0.847082	0.615180
H	-6.565520	-0.705894	1.201222
H	-5.410030	-1.922923	0.610486
H	-6.311373	-0.995350	-1.487228
H	-3.919061	-0.589863	1.972155
H	-4.797743	0.915298	1.597135
C	4.443900	-0.430986	-0.037065
S	2.724552	0.255717	0.017153
O	2.106963	-0.321606	-1.192315
O	2.924617	1.705345	-0.024628
O	2.197287	-0.273865	1.285563
F	4.425158	-1.761366	-0.009046
F	5.069417	-0.049596	-1.147816
F	5.153966	-0.005330	1.004600
H	-2.355138	-2.367403	0.051417

TS-4

ZPE	0.307270
DE	0.328436
DH	0.329380
DG	0.254350
E	-1520.032901

H -1519.703520

Gibbs free energy -1519.778551

Imaginary frequency -397.046

Cartesian coordinates

C	-1.069517	1.775906	-1.257310
N	-1.158463	1.319725	0.142735
C	-1.656786	0.165262	0.471248
C	-3.395032	0.450605	1.550216
H	-3.099315	1.437041	1.913575
H	-3.450354	-0.343207	2.301442
C	-3.981143	0.301860	0.330618
C	-4.673363	-0.938219	-0.168207
O	-3.899103	-1.584609	-1.151460
C	-3.108493	-2.649792	-0.689720
C	-2.234543	-2.322363	0.531909
H	-1.598249	-3.197155	0.723138
H	-2.873380	-2.198816	1.418212
C	-1.373985	-1.124040	0.338401
H	-0.329703	-1.257885	0.017459
H	-2.474396	-2.941573	-1.538493
H	-3.742894	-3.516851	-0.425102
H	-4.927495	-1.612614	0.667344
H	-5.617438	-0.627678	-0.643040
H	-3.960802	1.144077	-0.370712
C	-0.677257	2.332331	1.090835
C	0.058115	3.317644	0.187865
H	0.102534	4.324348	0.624353
C	-0.733058	3.257263	-1.121399
H	-0.164083	3.627192	-1.984784
H	-1.657329	3.851608	-1.040618
H	1.084767	2.957802	0.022217
H	-0.030361	1.854346	1.836974
H	-1.535276	2.809049	1.597385
H	-2.017287	1.573963	-1.778081
H	-0.263787	1.206854	-1.747088
C	3.651317	-1.044326	0.066599
F	3.240909	-2.249792	0.459842
F	4.532968	-0.599759	0.960065

F	4.281138	-1.185289	-1.097896
S	2.218145	0.119477	-0.087440
O	1.666299	0.137392	1.273535
O	1.371246	-0.552139	-1.091183
O	2.841695	1.366926	-0.540732

10

ZPE	0.378243
DE	0.410233
DH	0.411177
DG	0.311440
E	-2520.681664
H	-2520.270487
Gibbs free energy	-2520.370224

Cartesian coordinates

C	-2.834708	1.514630	-1.756132
N	-2.812699	1.701411	-0.304843
C	-1.953164	1.012469	0.464296
O	-1.132780	0.239557	-0.043698
S	1.440695	-0.091903	0.531292
O	1.003898	-0.266017	1.884706
O	1.471562	1.137481	-0.204398
C	0.867151	-1.563276	-0.551254
F	0.459648	-1.108051	-1.702977
F	1.890731	-2.375746	-0.737920
F	-0.073679	-2.208827	0.084945
C	-1.969325	1.215223	1.966331
H	-2.688432	1.984362	2.276801
H	-0.963413	1.585192	2.218509
C	-2.221907	-0.088732	2.726005
C	-3.678777	-0.534097	2.675449
O	-4.196287	-0.630692	1.370512
C	-3.781008	-1.763631	0.645648
C	-4.449529	-1.765109	-0.703886
C	-3.739427	-1.984991	-1.815557
H	-2.655642	-2.129908	-1.778444
H	-4.073283	-2.681635	1.199083
H	-2.685286	-1.782366	0.520304

H	-3.797441	-1.497679	3.208148
H	-4.308123	0.207889	3.192569
H	-1.553897	-0.868193	2.329482
H	-1.948601	0.046205	3.784278
C	-3.966933	2.471577	0.168448
C	-4.631147	2.954936	-1.121812
H	-5.713841	3.096016	-0.999205
C	-4.266548	1.872205	-2.137823
H	-4.354878	2.205480	-3.181004
H	-4.913304	0.990058	-2.004190
H	-4.198415	3.920262	-1.429748
S	4.224199	-0.464005	-0.464175
O	3.617706	-0.197985	-1.743183
O	5.143825	-1.543007	-0.237736
C	5.051121	1.099547	0.129726
F	5.594482	0.879888	1.306157
F	5.982668	1.408048	-0.748397
F	4.169766	2.069912	0.211863
O	3.077588	-0.590604	0.680078
H	-2.103737	2.188876	-2.235870
H	-2.555386	0.483387	-2.005314
H	-3.660008	3.307350	0.813650
H	-4.635898	1.811344	0.743409
H	-4.218055	-2.032222	-2.798745
C	-5.934048	-1.539078	-0.705403
H	-6.352320	-1.590718	-1.720718
H	-6.442509	-2.290326	-0.077593
H	-6.174092	-0.555657	-0.272867

11

ZPE	0.333928
DE	0.358271
DH	0.359215
DG	0.274011
E	-1559.340590
H	-1558.981375
Gibbs free energy	-1559.066579

Cartesian coordinates

C	-0.093739	2.331926	1.243058
N	0.374835	1.251294	0.333738
C	0.679438	0.095029	0.702389
C	0.927321	-1.124144	1.070847
C	2.273033	-1.685970	1.422560
H	2.513133	-2.461002	0.675848
H	2.195459	-2.211608	2.386628
C	3.370432	-0.631760	1.508754
O	3.380663	0.255529	0.415437
C	3.728669	-0.311098	-0.833916
C	5.126392	-0.885099	-0.859806
C	5.341259	-2.145651	-1.250074
H	6.351518	-2.562979	-1.308142
H	4.512789	-2.800273	-1.539677
H	2.994068	-1.074876	-1.147474
H	3.663116	0.521219	-1.553026
H	4.344567	-1.138387	1.627667
H	3.210525	-0.001964	2.396779
H	0.033579	-1.761011	1.091561
C	0.413910	1.708299	-1.082441
C	-0.301510	3.052336	-1.032277
H	-1.382653	2.887284	-1.142154
C	0.007765	3.576366	0.372374
H	1.027818	3.988813	0.418001
H	-0.692915	4.353418	0.704388
H	0.044293	3.722677	-1.829656
H	1.473803	1.801478	-1.360657
H	-0.078460	0.949648	-1.702913
H	-1.133799	2.088850	1.503001
H	0.525804	2.342384	2.147612
C	-4.171871	-1.085776	-0.250101
F	-4.692502	-0.887334	-1.458871
F	-5.064509	-0.689578	0.654062
F	-3.978779	-2.393834	-0.093617
S	-2.581483	-0.157066	-0.057583
O	-2.978991	1.242540	-0.258993
O	-1.732759	-0.729023	-1.113585
O	-2.164215	-0.487503	1.314830
C	6.224265	0.050606	-0.440216
H	6.064588	0.404051	0.591054

H	7.212081	-0.427978	-0.495476
H	6.232902	0.948243	-1.080783

TS-5-chair

ZPE	0.334539
DE	0.357275
DH	0.358220
DG	0.279539
E	-1559.319722
H	-1558.961502
Gibbs free energy	-1559.040182
Imaginary frequency	-266.635

Cartesian coordinates

C	1.069677	2.272730	1.243161
N	1.484300	1.506918	0.072199
C	2.455019	0.587179	0.184602
C	3.178704	0.130883	1.240129
C	4.159542	-0.907184	0.761657
H	3.926854	-1.925172	1.112583
C	4.024138	-0.764467	-0.753167
H	4.772370	-0.077973	-1.172454
H	4.022876	-1.697730	-1.326522
O	2.727588	-0.124710	-0.964120
C	1.270439	-1.470706	-1.209843
H	0.495330	-0.697599	-1.251596
H	1.666204	-1.825166	-2.165464
C	1.347131	-2.290767	-0.051938
C	0.845660	-1.742124	1.091580
H	0.953440	-2.254148	2.052955
H	0.246810	-0.825805	1.070937
H	5.190096	-0.682043	1.074241
H	3.158219	0.567299	2.236326
C	1.126227	2.169206	-1.189539
H	1.975321	2.168006	-1.888016
H	0.278503	1.647045	-1.659373
C	0.720552	3.572253	-0.739337
H	1.608248	4.222919	-0.682090
C	0.145365	3.337648	0.658444

H	0.116195	4.245884	1.275774
H	-0.876564	2.937778	0.585536
H	0.002354	4.035335	-1.430014
H	0.560513	1.621533	1.968613
H	1.952475	2.727006	1.732203
C	-3.373340	-1.001008	0.073889
S	-2.227713	0.444503	-0.093985
O	-1.496193	0.415629	1.182373
O	-3.120146	1.589837	-0.277042
O	-1.434729	0.073539	-1.279762
F	-2.680963	-2.125907	0.256288
F	-4.187999	-0.846765	1.114895
F	-4.121289	-1.151356	-1.016688
C	2.041518	-3.628605	-0.091889
H	3.019573	-3.574878	-0.591723
H	2.189764	-4.027429	0.920850
H	1.431039	-4.353667	-0.652941

TS-5-boat

ZPE	0.334998
DE	0.357414
DH	0.358358
DG	0.281566
E	-1559.317568
H	-1558.959209
Gibbs free energy	-1559.036002
Imaginary frequency	-252.311

Cartesian coordinates

C	4.391030	-0.122236	1.152272
N	3.518360	-0.346474	0.010497
C	2.321952	-0.944054	0.163159
C	1.698232	-1.519825	1.216617
C	0.329798	-1.979900	0.791235
H	-0.492028	-1.396862	1.235155
C	0.411782	-1.804348	-0.724024
H	0.721633	-2.726135	-1.235248
H	-0.488020	-1.399886	-1.198722
O	1.497884	-0.844378	-0.936852

C	0.819950	1.051908	-1.055907
H	1.280514	1.219984	-2.032571
H	-0.222151	0.711344	-1.058581
C	1.325749	1.724638	0.091767
C	0.718361	1.424787	1.273375
H	1.095416	1.826759	2.218913
H	-0.205698	0.838242	1.304364
H	0.146975	-3.034808	1.043274
H	2.172069	-1.734434	2.172401
C	4.231166	-0.297198	-1.270331
H	4.039006	-1.202170	-1.865686
H	3.905673	0.571527	-1.867495
C	5.696647	-0.167621	-0.848236
H	6.142833	-1.168439	-0.737066
C	5.620436	0.520400	0.516386
H	6.525256	0.382037	1.123378
H	5.456819	1.602513	0.392015
H	6.293890	0.388456	-1.583550
H	3.890204	0.519195	1.896155
H	4.648670	-1.076476	1.649553
C	-4.583386	0.396694	-0.081931
S	-2.872626	-0.303529	0.034219
O	-2.230826	0.210627	-1.191563
O	-3.087217	-1.751568	0.056715
O	-2.358863	0.279829	1.283831
F	-4.551096	1.726707	-0.116721
F	-5.193920	-0.030876	-1.184281
F	-5.315899	0.028241	0.966025
C	2.512063	2.647793	-0.002964
H	3.178051	2.370238	-0.830568
H	2.170782	3.678850	-0.188387
H	3.091337	2.652899	0.930915

TS-6

ZPE	0.335494
DE	0.357900
DH	0.358844
DG	0.281416
E	-1559.320247
H	-1558.961402

Gibbs free energy -1559.038831

Imaginary frequency -344.456

Cartesian coordinates

C	-0.883663	1.623475	-1.264855
N	-1.027648	1.152770	0.132054
C	-1.422930	-0.042491	0.431248
C	-3.111772	0.071901	1.705136
H	-2.806644	1.015976	2.163459
H	-3.005294	-0.821795	2.324599
C	-3.913986	0.067660	0.602270
C	-4.538299	-1.200021	0.061002
O	-3.812938	-1.654049	-1.060322
C	-2.949577	-2.737673	-0.832861
C	-1.947819	-2.540073	0.314960
H	-1.288622	-3.418711	0.328272
H	-2.486548	-2.541218	1.274265
C	-1.120326	-1.308443	0.190406
H	-0.078626	-1.388369	-0.160199
H	-2.410248	-2.891263	-1.778063
H	-3.528393	-3.656460	-0.620381
H	-4.612618	-1.971381	0.845206
H	-5.561887	-0.968894	-0.275342
C	-0.607494	2.175857	1.102095
C	0.090607	3.213512	0.228690
H	0.061722	4.215281	0.677432
C	-0.659636	3.120634	-1.102546
H	-0.093676	3.540662	-1.944719
H	-1.625446	3.647711	-1.041167
H	1.140890	2.918890	0.084766
H	0.048711	1.718497	1.852829
H	-1.500408	2.597270	1.595222
H	-1.772736	1.353310	-1.848369
H	-0.003096	1.120396	-1.693993
C	3.956354	-0.890270	0.095676
F	3.647829	-2.117666	0.512240
F	4.780252	-0.348103	0.990212
F	4.614293	-1.001037	-1.056407
S	2.430422	0.140259	-0.107220

O	1.850751	0.137216	1.241760
O	1.664079	-0.618621	-1.114107
O	2.951866	1.428694	-0.575448
C	-4.202363	1.310806	-0.169947
H	-3.540990	2.142679	0.107182
H	-4.133291	1.120125	-1.251375
H	-5.242022	1.619839	0.034389

12

ZPE	0.189594
DE	0.199493
DH	0.200437
DG	0.153593
E	-462.058551
H	-461.858114
Gibbs free energy	-461.904957

Cartesian coordinates

C	1.625865	-0.768399	0.082017
C	0.302171	-0.111654	-0.282443
C	0.618707	1.369284	-0.080695
H	0.036255	2.040566	-0.724664
H	0.442631	1.652125	0.969592
C	2.109137	1.415883	-0.397086
H	2.300381	1.545384	-1.474333
H	2.661950	2.184709	0.157079
O	2.614164	0.133562	0.001892
C	-0.879665	-0.717970	0.474704
H	-0.837912	-1.808104	0.332032
H	-0.746029	-0.528001	1.553053
C	-2.224402	-0.190108	0.020349
C	-2.913846	-0.805281	-0.947452
H	-2.538318	-1.717881	-1.422168
H	-3.876746	-0.420555	-1.298115
H	0.168693	-0.312052	-1.362147
O	1.826662	-1.911033	0.392159
C	-2.732052	1.050510	0.702873
H	-2.860245	0.868283	1.783020
H	-2.015884	1.882286	0.609658

H	-3.696886	1.379422	0.290977

13

ZPE	0.190502
DE	0.199328
DH	0.200273
DG	0.157137
E	-462.035178
H	-461.834905
Gibbs free energy	-461.878041

Cartesian coordinates

C	-1.595249	-0.515140	-0.039847
O	-2.204257	-1.495756	-0.372452
C	-1.585046	0.934894	-0.504859
H	-2.268006	1.541443	0.111690
H	-1.781087	1.129732	-1.569965
C	-0.122198	0.998002	0.023214
C	0.869245	0.627995	-1.092219
O	1.973105	-0.095126	-0.610625
C	1.597006	-1.379005	-0.166389
C	0.703429	-1.303945	1.066817
H	1.315625	-1.012876	1.934856
H	0.275534	-2.295084	1.286460
C	-0.404777	-0.280054	0.879275
H	-0.822828	0.032866	1.852341
H	1.077315	-1.918216	-0.984438
H	2.522957	-1.927448	0.058306
H	0.356699	0.034027	-1.876966
H	1.257416	1.538321	-1.574113
C	0.310324	2.238880	0.782681
H	0.322822	3.121499	0.123247
H	-0.369336	2.448410	1.623353
H	1.325701	2.104626	1.187807

16

ZPE	0.512477
DE	0.545607
DH	0.546551

DG	0.446271
E	-1925.279245
H	-1924.732694
Gibbs free energy	-1924.832974

Cartesian coordinates

C	-4.688401	1.160815	-1.146154
N	-3.798365	0.188086	-0.540213
C	-2.438851	0.282897	-0.674766
C	-1.750297	1.137660	-1.453310
C	-0.259918	1.295893	-1.492383
C	0.204483	2.711855	-1.146863
O	-0.103463	3.110510	0.170089
C	0.793362	2.640724	1.157101
C	2.163873	3.276402	1.070721
C	3.264197	2.519807	1.142665
H	4.265753	2.962252	1.130297
H	3.186795	1.431910	1.220597
H	0.888804	1.541775	1.127014
H	0.333987	2.918166	2.120888
H	1.292614	2.783798	-1.328535
H	-0.288818	3.440481	-1.810638
H	0.251584	0.572229	-0.841412
H	0.105955	1.085025	-2.512899
C	-4.508829	-0.921158	0.091954
C	-5.954547	-0.707371	-0.358051
H	-6.123559	-1.218456	-1.319160
C	-6.048702	0.806882	-0.549598
H	-6.170523	1.306837	0.424665
H	-6.883771	1.112974	-1.194242
H	-6.678740	-1.103103	0.367011
H	-4.420395	-0.873877	1.193950
H	-4.120042	-1.899876	-0.229575
H	-4.691343	1.069723	-2.249998
H	-4.363406	2.186738	-0.903429
C	-1.545425	-0.260470	1.507529
C	-1.286681	-1.823656	-0.286254
C	-0.857841	-1.114188	2.349624
C	-0.614397	-2.683668	0.561269

C	-0.355309	-2.333349	1.887901
H	-0.692299	-0.803006	3.381559
H	-0.243437	-3.625113	0.156280
N	-1.725550	-0.629674	0.205034
C	-2.099465	1.048190	1.965031
H	-1.756011	1.863145	1.310938
H	-3.199430	1.034059	1.928369
H	-1.782409	1.244834	2.995585
C	-1.513543	-2.146259	-1.724980
H	-1.304180	-3.208216	-1.898599
H	-2.541939	-1.923128	-2.040534
H	-0.818935	-1.556551	-2.340066
C	0.495666	-3.209203	2.745525
H	0.460578	-2.914811	3.802215
H	0.206584	-4.265429	2.646483
H	1.531896	-3.116538	2.378474
H	-2.334900	1.794582	-2.102996
C	2.189266	4.771958	0.931637
H	1.627893	5.250009	1.752101
H	3.216288	5.164453	0.935035
H	1.700189	5.085418	-0.004622
C	3.897418	-0.635549	-0.964731
F	3.581601	0.501696	-1.584293
F	4.741281	-0.343503	0.024967
F	4.541289	-1.410865	-1.834811
O	1.604328	-1.707599	-1.552571
O	2.901209	-2.689465	0.322635
O	1.809197	-0.484946	0.598973
S	2.376786	-1.482025	-0.324844

TS-7

ZPE	0.334742
DE	0.357052
DH	0.357996
DG	0.281296
E	-1559.288917
H	-1558.930921
Gibbs free energy	-1559.007621
Imaginary frequency	-438.023

Cartesian coordinates

C	1.721303	2.236016	-1.479123
N	1.942264	1.267351	-0.378500
C	2.450068	0.091187	-0.630060
C	2.407795	-0.785536	-1.634202
C	1.064574	-1.262952	-2.117398
H	1.050804	-1.479015	-3.198170
H	0.281513	-0.526046	-1.886562
C	0.757505	-2.562365	-1.353026
O	0.866810	-2.421111	0.052861
C	2.134637	-2.616104	0.640868
C	2.539922	-1.350191	1.370130
C	3.535226	-0.539442	0.840665
H	3.878187	0.323253	1.418720
H	4.286034	-0.991212	0.184390
H	2.074124	-3.432372	1.383651
H	2.893838	-2.905510	-0.099251
H	1.421642	-3.374261	-1.702489
H	-0.279078	-2.873617	-1.550130
H	3.254544	-1.460679	-1.777914
C	1.529031	1.828111	0.912944
C	1.373418	3.318369	0.629565
H	2.342393	3.830452	0.741954
C	0.912669	3.350387	-0.825975
H	-0.155574	3.098683	-0.883121
H	1.080353	4.318522	-1.316331
H	0.657913	3.785889	1.318908
H	0.580573	1.365810	1.223488
H	2.289287	1.616895	1.674173
H	2.703870	2.579636	-1.841441
H	1.188534	1.742989	-2.300860
C	1.670193	-0.961510	2.498743
H	1.495422	-1.828501	3.154675
H	2.063454	-0.120527	3.082228
H	0.675499	-0.688225	2.087749
C	-3.123704	-0.695634	-0.064147
F	-3.989794	-0.905338	0.925414
F	-3.818229	-0.482903	-1.180704
F	-2.415143	-1.812105	-0.230176

S	-2.023548	0.747407	0.313058
O	-1.354472	0.328587	1.557097
O	-1.155953	0.816099	-0.873562
O	-2.960103	1.862545	0.458877

17

ZPE	0.337138
DE	0.359882
DH	0.360826
DG	0.281434
E	-1559.335319
H	-1558.974493
Gibbs free energy	-1559.053885

Cartesian coordinates

C	-1.505593	1.943692	-0.880045
N	-2.543151	1.191369	-0.174314
C	-2.278078	0.025449	0.511647
C	-3.503285	-0.885518	0.736447
H	-4.430119	-0.336931	0.547474
H	-3.501680	-1.417519	1.692782
C	-2.950214	-1.615828	-0.391880
C	-2.278255	-2.963513	-0.218443
O	-0.927368	-2.934507	-0.579421
C	-0.032275	-2.652058	0.469701
C	-0.574378	-1.638900	1.497099
C	-1.014804	-0.378043	0.856749
H	-0.202246	0.326840	0.651596
H	0.869202	-2.242266	-0.001062
H	0.226799	-3.580156	1.011148
H	-2.440346	-3.364844	0.793439
H	-2.794349	-3.638551	-0.921104
C	-3.417266	2.198266	0.431347
C	-2.445686	3.274380	0.943469
H	-2.913265	4.269049	0.962129
C	-1.235001	3.190977	-0.013448
H	-0.291463	3.066632	0.535932
H	-1.137797	4.088557	-0.640477
H	-2.139776	3.033848	1.972931

H	-4.044441	1.766659	1.222109
H	-4.083209	2.611126	-0.345025
H	-1.912389	2.236269	-1.861869
H	-0.608359	1.339579	-1.059583
C	3.564333	-0.321304	0.250328
F	2.994893	-1.231216	1.046494
F	4.432112	0.371420	0.983677
F	4.244878	-0.973067	-0.689354
S	2.291110	0.797911	-0.501684
O	1.644443	1.385130	0.686194
O	1.439068	-0.134713	-1.258937
O	3.086650	1.723472	-1.305442
C	-3.224005	-1.209225	-1.788373
H	-3.409153	-0.130883	-1.876256
H	-2.413596	-1.528935	-2.458066
H	-4.147579	-1.735910	-2.090547
H	0.262007	-1.401837	2.170286
H	-1.366195	-2.096427	2.107199

18

ZPE	0.339739
DE	0.361452
DH	0.362397
DG	0.286780
E	-1559.419765
H	-1559.057369
Gibbs free energy	-1559.132986

Cartesian coordinates

C	0.393608	2.526379	1.364514
N	0.907315	1.745980	0.220401
C	1.483586	0.611836	0.328781
C	1.843999	-0.232376	1.497341
H	1.054169	-0.372897	2.248457
H	2.759744	0.148075	1.980786
C	2.115135	-1.392377	0.485887
C	3.461007	-2.099465	0.615891
O	4.553079	-1.297764	0.250201
C	4.463731	-0.892720	-1.094613

C	3.370052	0.148541	-1.271617
H	3.248350	0.400262	-2.334593
H	3.695428	1.068239	-0.760115
C	2.021690	-0.340428	-0.688215
H	1.295081	-0.596157	-1.472530
H	5.438423	-0.469122	-1.375965
H	4.276827	-1.770608	-1.744532
H	3.616465	-2.411442	1.660445
H	3.446038	-3.015157	-0.008862
O	-1.477166	-0.137835	1.250836
S	-2.105937	0.051762	-0.067624
O	-1.224610	-0.210423	-1.216781
O	-2.953356	1.242611	-0.184717
C	-3.315362	-1.349069	-0.151094
F	-2.691248	-2.522222	-0.053457
F	-4.200887	-1.270846	0.838799
F	-3.979908	-1.331780	-1.303718
C	0.607704	2.403890	-1.067793
C	-0.395380	3.484887	-0.681111
H	-0.377175	4.328773	-1.382629
C	0.024475	3.867103	0.740330
H	-0.772829	4.367919	1.304457
H	0.901700	4.532838	0.719966
H	-1.406783	3.051538	-0.671846
H	0.212386	1.654715	-1.764973
H	1.545576	2.824712	-1.461263
H	1.157723	2.579050	2.150891
H	-0.486487	1.987443	1.746673
C	0.979946	-2.404495	0.457451
H	1.070120	-3.064327	-0.419455
H	1.003015	-3.030533	1.364341
H	0.005674	-1.902686	0.414474

TS-8

ZPE	0.420105
DE	0.448360
DH	0.449304
DG	0.358928
E	-1906.522086
H	-1906.072781

Gibbs free energy -1906.163158

Imaginary frequency -73.454

Cartesian coordinates

C	0.448312	1.408867	1.480663
N	1.339216	2.086752	0.534397
C	2.125090	1.324118	-0.344166
C	3.510813	1.949508	-0.723579
H	3.545460	2.997933	-0.417976
H	3.825787	1.737821	-1.748740
C	3.756072	0.942077	0.264995
C	4.388375	-0.379207	-0.108463
O	4.476215	-0.644591	-1.472319
C	3.363710	-1.293258	-2.032829
C	2.172699	-0.374348	-2.295177
C	1.563975	0.348768	-1.147222
H	0.479317	0.217902	-1.022345
H	3.039876	-2.129399	-1.392190
H	3.693303	-1.702214	-2.997584
H	5.419974	-0.313465	0.278682
H	3.890095	-1.198103	0.439662
C	0.699708	3.296714	-0.010064
C	-0.734013	3.229102	0.513251
H	-1.198503	4.219877	0.618983
C	-0.587117	2.471058	1.832542
H	-1.527513	2.013931	2.167808
H	-0.211507	3.137346	2.626888
H	-1.344671	2.623563	-0.171266
H	0.738528	3.306595	-1.111082
H	1.215770	4.206831	0.347378
H	1.008455	1.047788	2.358525
H	-0.062961	0.546093	1.024536
C	-4.061934	0.721472	-0.555891
F	-3.815390	1.950381	-0.098488
F	-5.158536	0.274966	0.053866
F	-4.324652	0.816513	-1.857967
S	-2.631326	-0.418108	-0.245209
O	-1.547963	0.205804	-1.029861
O	-3.114559	-1.695238	-0.775179

O	-2.464314	-0.351959	1.211060
C	3.877143	1.289937	1.716393
H	3.178469	2.089334	1.991223
H	3.721171	0.411732	2.358450
H	4.903102	1.657538	1.881726
H	1.391804	-0.972178	-2.781168
H	2.485872	0.407065	-3.013038
C	1.332001	-1.855782	1.713166
C	0.475780	-2.493162	2.599587
C	-0.597445	-3.219445	2.075503
C	-0.767752	-3.287588	0.700136
C	0.164831	-2.608246	-0.077026
H	0.639546	-2.416996	3.675145
H	-1.598400	-3.811280	0.229696
N	1.174829	-1.914329	0.385681
H	2.178050	-1.263785	2.074298
H	-1.303837	-3.723577	2.738156
F	0.056265	-2.657774	-1.403352

19

ZPE	0.425623
DE	0.452811
DH	0.453755
DG	0.365943
E	-1906.570991
H	-1906.117236
Gibbs free energy	-1906.205048

Cartesian coordinates

C	3.712524	-1.320172	0.393165
N	2.950279	-0.223297	-0.179684
C	2.114796	0.609178	0.607206
C	0.611656	0.438098	0.342353
C	0.104639	1.155726	-0.908491
H	0.821214	1.079194	-1.736103
H	-0.842412	0.699941	-1.227852
C	-0.175466	2.626615	-0.609915
O	0.945072	3.391782	-0.233172
C	1.470930	3.101473	1.040199

C	2.541942	2.018250	1.007095
C	2.503693	0.918275	2.033588
H	3.462362	0.539300	2.395504
H	1.708562	0.931146	2.784480
H	0.658182	2.837722	1.741250
H	1.924509	4.031928	1.418028
H	-0.575525	3.098742	-1.518792
H	-0.958811	2.688955	0.170408
H	0.029075	0.837964	1.177992
C	3.410680	0.130825	-1.518456
C	4.707445	-0.663064	-1.671813
H	4.943246	-0.897447	-2.719550
C	4.444493	-1.902209	-0.813467
H	5.357959	-2.445450	-0.532967
H	3.785666	-2.601332	-1.354535
H	5.552565	-0.091844	-1.253292
H	3.560207	1.215630	-1.625666
H	3.059346	-2.061153	0.888247
H	4.442933	-0.975835	1.153729
C	-4.141054	0.785341	-0.597248
F	-4.882959	1.527970	0.221321
F	-3.345728	1.607842	-1.285629
F	-4.955384	0.187507	-1.463804
S	-3.151707	-0.462486	0.353523
O	-4.171683	-1.267291	1.024567
O	-2.399230	-1.154940	-0.706051
O	-2.335384	0.384476	1.240703
C	3.901452	2.526856	0.576859
H	4.301672	3.208931	1.344128
H	3.831301	3.088930	-0.366768
H	4.623865	1.709379	0.444445
C	-0.057114	-1.519177	1.621033
C	-0.287355	-2.861134	1.809338
C	-0.144704	-3.731858	0.725745
C	0.210651	-3.222438	-0.511112
C	0.412929	-1.857807	-0.626103
H	-0.579827	-3.217949	2.796100
H	0.317866	-3.840376	-1.402167
N	0.304054	-1.020536	0.413228
H	-0.150357	-0.790640	2.424013

H	-0.321934	-4.801988	0.845502
F	0.700417	-1.344911	-1.788554
H	2.685192	-0.171707	-2.295568

TS-9

ZPE	0.420805
DE	0.449085
DH	0.450029
DG	0.357447
E	-1906.515027
H	-1906.064998
Gibbs free energy	-1906.157580
Imaginary frequency	-306.874

Cartesian coordinates

C	3.616737	-0.854306	-1.513971
N	2.736577	-1.150970	-0.385006
C	1.369802	-1.038793	-0.496033
C	0.493634	-0.507268	0.486135
C	0.397375	-0.833785	1.925216
H	1.287493	-1.332138	2.325299
H	0.191680	0.080120	2.500837
C	-0.867649	-1.716865	2.086589
O	-0.880361	-2.876172	1.300003
C	-0.965259	-2.658667	-0.093201
C	0.413759	-2.437987	-0.705679
C	0.676608	-1.422405	-1.770708
H	1.320681	-1.726606	-2.599480
H	-0.174256	-0.794403	-2.052167
H	-1.657904	-1.839407	-0.338942
H	-1.373154	-3.585622	-0.526956
H	-0.922398	-2.047251	3.132938
H	-1.747398	-1.088543	1.863888
H	-0.448940	-0.138632	0.070344
C	3.453529	-1.235569	0.877713
C	4.919080	-1.268721	0.446710
H	5.592609	-0.880196	1.222912
C	4.918357	-0.434824	-0.834394
H	5.799435	-0.605643	-1.468281

H	4.879899	0.638184	-0.584834
H	5.221289	-2.304425	0.222718
H	3.165276	-2.135329	1.446475
H	3.252174	-0.356776	1.518689
H	3.195422	-0.057770	-2.145973
H	3.773879	-1.742656	-2.155487
C	1.283501	-3.664879	-0.615112
H	1.283913	-4.059245	0.410964
H	2.317160	-3.472043	-0.927914
H	0.857978	-4.439969	-1.274135
C	2.012297	2.095443	1.650826
C	2.857460	3.195268	1.609001
C	3.062894	3.828066	0.379917
C	2.418367	3.345589	-0.752184
C	1.593792	2.238436	-0.580358
H	3.345154	3.547712	2.518361
H	2.535207	3.796220	-1.738163
N	1.390582	1.627836	0.563175
H	1.826142	1.561703	2.587504
H	3.722489	4.694964	0.304781
F	0.961898	1.735106	-1.632739
C	-4.462395	0.093898	-0.025893
F	-5.159560	-0.079577	-1.146201
F	-5.240201	0.722202	0.852500
F	-4.179264	-1.113677	0.470078
S	-2.911838	1.060069	-0.347754
O	-2.195089	0.201272	-1.310623
O	-2.287539	1.134345	0.981713
O	-3.404726	2.325095	-0.888178

20

ZPE	0.339788
DE	0.361396
DH	0.362340
DG	0.286638
E	-1559.399240
H	-1559.036900
Gibbs free energy	-1559.112602

Cartesian coordinates

C	0.517914	3.079778	-1.044403
N	0.908135	2.054113	-0.052893
C	1.334626	0.896109	-0.378410
C	1.646422	-0.362621	0.341106
C	2.095459	-0.833322	1.698602
H	3.027531	-0.345009	2.020171
H	1.333067	-0.688564	2.477054
C	2.297896	-2.347650	1.469429
O	3.108249	-2.677115	0.354291
C	2.686768	-2.193140	-0.911938
C	2.634358	-0.675128	-0.807149
C	1.800371	0.307362	-1.686561
H	2.335912	0.973540	-2.376749
H	0.942124	-0.158750	-2.192180
H	1.689655	-2.594368	-1.180155
H	3.422808	-2.560413	-1.642218
H	2.783713	-2.811418	2.339056
H	1.297656	-2.810649	1.351735
H	0.719866	-0.920302	0.099455
C	0.530971	2.451657	1.317578
C	-0.535674	3.509221	1.069230
H	-1.499581	3.012854	0.874111
C	-0.031294	4.219226	-0.189473
H	0.773031	4.925435	0.068231
H	-0.817183	4.775037	-0.716807
H	-0.658724	4.180595	1.928708
H	1.420872	2.871902	1.810763
H	0.168709	1.571427	1.860003
H	-0.258372	2.627024	-1.678347
H	1.382520	3.352450	-1.663689
C	-2.754947	-1.648216	-0.199051
F	-3.503161	-2.020305	0.835739
F	-3.495181	-1.740838	-1.300355
F	-1.744925	-2.514458	-0.308944
S	-2.108908	0.072612	0.029597
O	-1.316150	-0.053346	1.263925
O	-1.317119	0.274887	-1.196406
O	-3.311266	0.899670	0.138543
C	4.030931	-0.097139	-0.580283

H	4.601368	-0.126228	-1.521921
H	4.583857	-0.676709	0.171002
H	3.993613	0.952378	-0.244380

21

ZPE	0.673780
DE	0.722049
DH	0.722993
DG	0.584336
E	-3118.708774
H	-3117.985781
Gibbs free energy	-3118.124438

Cartesian coordinates

C	-1.406591	3.742827	3.028338
N	-1.374074	2.953396	1.796356
C	-0.508574	1.918643	1.578976
C	-0.039420	1.540239	0.341830
C	-0.220176	2.314446	-0.910891
H	0.017473	3.371141	-0.691529
H	-1.278648	2.304583	-1.220772
C	0.612646	1.766473	-2.067223
O	1.931738	1.454046	-1.700858
C	2.789158	2.541008	-1.501341
C	3.615441	2.905949	-2.712656
C	3.594041	2.171902	-3.828690
H	4.207405	2.436254	-4.695496
H	2.964112	1.281155	-3.897785
H	2.237216	3.440603	-1.165624
H	3.461087	2.276763	-0.668079
H	0.582955	2.478880	-2.912332
H	0.159239	0.824652	-2.407677
H	0.630384	0.678812	0.294087
C	-2.529056	3.232005	0.935141
C	-3.491442	3.956071	1.870117
H	-4.071568	3.226677	2.458658
C	-2.541352	4.736007	2.779926
H	-2.158942	5.623287	2.250738
H	-3.006707	5.074596	3.715675

H	-4.200368	4.596092	1.327332
H	-2.256859	3.886341	0.089953
H	-2.930663	2.299229	0.512648
H	-1.636727	3.113403	3.907837
H	-0.440347	4.236235	3.215228
C	4.794192	-0.581398	0.051449
F	5.762096	0.162756	0.582808
F	4.519413	-0.102046	-1.156077
F	5.257851	-1.821485	-0.094869
S	3.297045	-0.582638	1.144037
O	2.997069	0.842925	1.341130
O	3.762904	-1.281713	2.348295
O	2.309275	-1.328459	0.344678
C	4.472916	4.127055	-2.537595
H	3.853097	5.018712	-2.343897
H	5.140395	4.011704	-1.666963
H	5.092727	4.322476	-3.424061
C	0.298804	-2.852534	-1.568131
N	-0.244876	-3.663093	-0.440627
C	0.334368	-3.853413	0.652528
C	0.998494	-4.066230	1.746684
C	0.965082	-3.181308	2.963957
H	1.690656	-2.370722	2.787206
H	1.341991	-3.745890	3.827629
C	-0.428868	-2.648385	3.297557
O	-1.160999	-2.225231	2.170746
C	-0.693161	-1.080301	1.490018
C	-1.029456	0.177432	2.268498
C	-0.017647	1.075128	2.782881
H	-0.236740	1.565589	3.735519
H	1.026293	0.769047	2.668864
H	0.385582	-1.144615	1.281196
H	-1.236567	-1.055315	0.536506
H	-0.333560	-1.842495	4.047570
H	-1.033739	-3.448842	3.746802
H	1.667109	-4.936026	1.726529
C	-1.593546	-4.204994	-0.773731
C	-1.856084	-3.687627	-2.186881
H	-2.430069	-2.754590	-2.151083
C	-0.464860	-3.404490	-2.760383

H	0.010968	-4.327946	-3.126040
H	-0.493646	-2.678331	-3.583060
H	-2.422238	-4.417999	-2.779164
H	-1.533156	-5.300333	-0.718549
H	-2.304288	-3.840460	-0.020910
H	1.388729	-2.950686	-1.595637
H	0.026743	-1.806165	-1.369944
C	-4.475596	-0.435832	-1.033591
F	-4.540244	0.275954	0.094892
F	-4.372984	-1.720918	-0.692629
F	-5.621439	-0.270189	-1.684156
S	-3.036139	0.093720	-2.081952
O	-3.240650	-0.649194	-3.326224
O	-1.875850	-0.347302	-1.284444
O	-3.205523	1.548846	-2.168556
C	-2.435691	0.275711	2.742568
H	-3.140443	-0.144920	2.015529
H	-2.508849	-0.318802	3.669644
H	-2.719550	1.304205	2.996028

TS-10

ZPE	0.512585
DE	0.544339
DH	0.545283
DG	0.447970
E	-1925.220429
H	-1924.675145
Gibbs free energy	-1924.772459
Imaginary frequency	-181.762

Cartesian coordinates

C	4.178823	0.820782	-1.548128
N	3.413624	0.309407	-0.415659
C	2.245478	-0.409511	-0.600601
C	1.125675	-0.389031	0.262899
C	1.107470	-0.441026	1.731121
H	2.040049	-0.113930	2.200540
H	0.259737	0.149113	2.106108
C	0.767002	-1.919609	2.098984

O	1.541150	-2.898082	1.465165
C	1.353507	-3.027606	0.066986
C	2.333011	-2.123000	-0.670453
C	2.009819	-1.253595	-1.831536
H	2.762201	-1.189301	-2.621296
H	0.978698	-1.275910	-2.193765
H	0.304942	-2.857220	-0.224497
H	1.606385	-4.069046	-0.186242
H	0.939580	-2.035411	3.177503
H	-0.304401	-2.090176	1.903296
H	0.197276	-0.757348	-0.181936
C	4.185374	0.330876	0.827912
C	5.592165	0.713134	0.368970
H	6.158820	1.240406	1.148779
C	5.327933	1.563663	-0.874102
H	6.203337	1.663238	-1.530520
H	5.003330	2.574963	-0.580643
H	6.157950	-0.190948	0.091738
H	4.154669	-0.645628	1.335846
H	3.794317	1.081340	1.536962
H	3.559771	1.472681	-2.183913
H	4.574780	0.008027	-2.188600
C	-4.136690	-1.038971	-0.415963
F	-4.968459	-0.575148	0.512832
F	-3.947048	-0.075011	-1.316600
F	-4.726829	-2.061941	-1.028366
S	-2.531107	-1.561614	0.346743
O	-2.916196	-2.631454	1.268831
O	-1.745431	-2.007427	-0.822539
O	-2.041968	-0.327765	0.976465
C	3.754819	-2.597223	-0.508073
H	3.852735	-3.530767	-1.087485
H	3.975289	-2.830711	0.542054
H	4.495723	-1.885722	-0.890207
C	-0.899906	1.882044	-1.054226
C	-1.964932	2.769422	-0.903501
C	-1.972410	3.684228	0.152695
C	-0.859590	3.692465	0.993823
C	0.180535	2.782965	0.784547
H	-2.793486	2.741561	-1.614765

H	-0.787972	4.412982	1.812373
N	0.136343	1.879472	-0.201471
C	-0.870266	0.904010	-2.190904
H	0.130988	0.875994	-2.644144
H	-1.120238	-0.107040	-1.833444
H	-1.600162	1.175977	-2.965049
C	1.403803	2.810344	1.655702
H	2.232989	2.310052	1.141246
H	1.695722	3.844946	1.886462
H	1.225405	2.298476	2.614189
C	-3.133540	4.610997	0.369997
H	-3.948641	4.076691	0.884794
H	-2.853476	5.473324	0.990966
H	-3.536264	4.977636	-0.585375

27

ZPE	0.379568
DE	0.411447
DH	0.412391
DG	0.311339
E	-2520.688344
H	-2520.275953
Gibbs free energy	-2520.377005

Cartesian coordinates

C	0.028129	-0.756838	-1.446652
N	-1.380831	-1.220545	-1.479188
C	-2.328436	-0.667833	-0.809560
O	-1.926929	0.382750	-0.066110
S	-2.719795	1.800741	0.234822
O	-2.778086	1.947579	1.660786
O	-3.864667	1.924027	-0.626533
O	3.113057	-1.418359	0.291558
S	3.429638	-0.183290	-0.439779
O	4.023060	-0.380434	-1.767427
O	2.414348	0.876480	-0.359496
C	4.825149	0.523364	0.554091
F	5.861885	-0.312644	0.590443
F	4.443066	0.754272	1.811340

F	5.248130	1.677167	0.038899
C	-1.351466	2.890712	-0.429979
F	-1.207103	2.633706	-1.710133
F	-1.734776	4.133682	-0.251676
F	-0.244505	2.648731	0.219915
C	-3.755962	-1.091572	-0.799494
H	-3.847893	-2.033907	-1.351621
H	-4.317451	-0.328665	-1.362801
C	-4.338289	-1.246854	0.610252
C	-3.665239	-2.386318	1.364365
O	-2.264513	-2.250100	1.350516
C	-1.705095	-1.523997	2.430204
C	-0.221655	-1.400134	2.204359
C	0.380459	-0.206540	2.213198
H	1.452540	-0.110197	2.024295
H	-0.189079	0.713905	2.372953
H	-1.903518	-2.070691	3.373811
H	-2.166690	-0.524428	2.519975
H	-4.050117	-2.444899	2.397231
H	-3.895493	-3.342110	0.867227
H	-4.262186	-0.307098	1.179588
H	-5.413491	-1.454232	0.513325
C	-1.528034	-2.446103	-2.296550
C	-0.181567	-2.561411	-3.003826
H	0.048977	-3.605218	-3.251940
C	0.797945	-1.941434	-2.011194
H	1.745594	-1.618171	-2.458678
H	1.037992	-2.645584	-1.201479
H	-0.199735	-1.981932	-3.939534
H	-2.378013	-2.343580	-2.981574
H	-1.710679	-3.286178	-1.609385
H	0.314432	-0.480320	-0.426378
H	0.103165	0.134927	-2.086125
C	0.515866	-2.685469	1.957668
H	1.561545	-2.492485	1.683957
H	0.481005	-3.332094	2.851415
H	0.036161	-3.249668	1.142658

TS-11

ZPE	0.380164
DE	0.410740
DH	0.411684
DG	0.314919
E	-2520.666181
H	-2520.254497
Gibbs free energy	-2520.351262
Imaginary frequency	-264.112

Cartesian coordinates

C	0.341529	-2.703304	0.487337
N	-0.107681	-1.421575	-0.108806
C	-0.578863	-0.470425	0.676989
C	-0.321727	-0.487866	2.153911
H	-0.880192	-1.297310	2.637354
C	-0.705721	0.910014	2.613656
C	-0.207712	1.785115	1.492584
H	-0.784320	2.697586	1.320425
H	0.871079	1.967088	1.524857
O	-0.426501	0.919588	0.277944
C	0.207768	1.316540	-1.047343
C	0.345932	2.801339	-1.115755
C	1.567815	3.329939	-0.986356
H	1.732899	4.407631	-1.075992
H	2.435243	2.695057	-0.788015
H	1.159734	0.777183	-1.084527
H	-0.514981	0.952182	-1.782634
H	-0.212585	1.183536	3.554088
H	-1.792143	1.007028	2.733676
H	0.758193	-0.650228	2.282680
C	-0.415361	-1.588854	-1.544767
C	-0.435545	-3.101590	-1.729252
C	0.617988	-3.570933	-0.730806
H	0.555734	-4.640459	-0.491015
H	1.628410	-3.352914	-1.108731
H	-0.216625	-3.384676	-2.767234
H	-1.425645	-3.502826	-1.460895
H	-1.371806	-1.107091	-1.785695

H	0.389174	-1.139627	-2.142560
H	1.235145	-2.543355	1.102869
H	-0.465885	-3.125668	1.107350
O	-3.409325	1.684595	0.795677
S	-3.225866	0.509945	-0.039353
O	-2.306418	-0.546541	0.612136
O	-2.936738	0.700757	-1.452967
C	-4.803806	-0.462551	0.040044
F	-5.111160	-0.729067	1.295457
F	-4.668452	-1.591789	-0.630876
F	-5.767362	0.258919	-0.504710
C	4.936375	0.222919	0.036475
F	4.872621	1.413303	-0.559740
F	5.467530	0.394857	1.243298
F	5.753713	-0.548979	-0.672902
S	3.254489	-0.543414	0.159644
O	3.508832	-1.820128	0.831207
O	2.505981	0.443475	0.962198
O	2.826337	-0.634799	-1.244958
C	-0.901753	3.591101	-1.395285
H	-1.718434	3.316065	-0.709345
H	-0.719635	4.670906	-1.310413
H	-1.268429	3.376988	-2.412468

28

ZPE	0.379963
DE	0.411751
DH	0.412696
DG	0.313701
E	-2520.683163
H	-2520.270468
Gibbs free energy	-2520.369462

Cartesian coordinates

C	0.513428	-2.880953	0.543677
N	0.118720	-1.553129	-0.005024
C	0.000639	-0.555212	0.784182
C	0.114022	-0.579592	2.263037
H	-0.351675	-1.481926	2.675660

C	-0.566947	0.717513	2.687239
C	-0.273131	1.660399	1.553422
H	-1.060300	2.385370	1.336556
H	0.730561	2.095638	1.573864
O	-0.261559	0.717206	0.357868
C	0.156442	1.280916	-1.074557
C	0.168404	2.763879	-1.046651
C	1.350584	3.378314	-0.905542
H	1.427826	4.469151	-0.932377
H	2.268757	2.804057	-0.755749
H	1.136834	0.819801	-1.228207
H	-0.646813	0.877805	-1.695074
H	-0.162411	1.112486	3.626147
H	-1.649386	0.566607	2.780052
H	1.190444	-0.586901	2.499894
C	-0.218002	-1.658251	-1.447894
C	-0.316203	-3.161929	-1.669045
C	0.722734	-3.722341	-0.704106
H	0.595152	-4.791036	-0.490838
H	1.739158	-3.558851	-1.092610
H	-0.123432	-3.417388	-2.718615
H	-1.323589	-3.516715	-1.402858
H	-1.156634	-1.121661	-1.631748
H	0.611812	-1.226948	-2.021544
H	1.414645	-2.761113	1.157540
H	-0.322218	-3.250714	1.157284
O	-3.304545	1.524467	0.782395
S	-3.278921	0.250440	0.051269
O	-2.688309	-0.877069	0.794694
O	-2.845699	0.347816	-1.353316
C	-5.063068	-0.219139	-0.084541
F	-5.603113	-0.357482	1.121831
F	-5.193064	-1.370561	-0.735690
F	-5.742590	0.713269	-0.743023
C	4.990272	0.362891	-0.096184
F	4.914604	1.497922	-0.786934
F	5.541355	0.628054	1.082762
F	5.784982	-0.473132	-0.753777
S	3.308454	-0.376735	0.122233
O	3.549655	-1.590773	0.904898

O	2.569763	0.682433	0.840433
O	2.844587	-0.590650	-1.258468
C	-1.142972	3.465299	-1.266566
H	-1.479276	3.318579	-2.306103
H	-1.937000	3.051565	-0.625738
H	-1.053944	4.543986	-1.079740

TS-12

ZPE	0.549317
DE	0.591366
DH	0.592310
DG	0.468196
E	-2886.543159
H	-2885.950849
Gibbs free energy	-2886.074963
Imaginary frequency	-1505.728

Cartesian coordinates

C	1.142011	1.107069	1.950622
N	1.046121	-0.111897	1.122808
C	0.803225	-0.007003	-0.148779
C	0.400030	1.095757	-0.926710
H	-0.859528	1.584929	-0.531330
C	0.468922	0.635042	-2.378879
C	0.294682	-0.863134	-2.283396
H	-0.745027	-1.204016	-2.228586
H	0.872231	-1.464909	-2.991525
O	0.863786	-1.178708	-0.923337
C	2.026535	-2.103566	-0.864350
C	1.591292	-3.532987	-0.799394
C	0.315435	-3.917744	-0.725116
H	0.068738	-4.982413	-0.688472
H	-0.509693	-3.203933	-0.687555
H	2.622433	-1.875647	-1.756525
H	2.620355	-1.790216	-0.000098
H	1.439984	0.889789	-2.824708
H	-0.334740	1.045140	-3.001461
H	0.958051	2.006406	-0.672456
C	1.017494	-1.338130	1.942420

C	0.575476	-0.809557	3.301849
C	1.241176	0.566105	3.375578
H	0.760130	1.238863	4.097545
H	2.298108	0.461001	3.662470
H	0.882816	-1.482331	4.113047
H	-0.520438	-0.717512	3.327590
H	0.326153	-2.062805	1.494863
H	2.025942	-1.774224	2.003151
H	2.011787	1.690912	1.622255
H	0.238905	1.711570	1.789118
O	-3.901390	-0.445006	-0.462004
S	-2.980229	-1.571620	-0.607923
O	-1.689563	-1.431185	0.098313
O	-2.855142	-2.127595	-1.961154
C	-3.796618	-2.927690	0.353430
F	-3.961655	-2.564749	1.624405
F	-3.058665	-4.036248	0.335194
F	-4.991297	-3.217418	-0.153127
C	4.994450	0.536792	0.659979
F	5.776325	-0.435761	0.198682
F	5.740470	1.364736	1.383022
F	4.096278	-0.024260	1.480647
S	4.154850	1.428985	-0.734529
O	3.334719	2.434252	-0.042068
O	5.268212	1.955153	-1.519758
O	3.391013	0.345277	-1.378839
C	-2.255005	3.313383	-1.124122
C	-3.343433	4.141347	-0.889127
C	-4.145551	3.956254	0.243731
C	-3.791478	2.928160	1.111644
C	-2.684553	2.120097	0.845214
N	-1.950027	2.319336	-0.261258
H	-3.567960	4.939122	-1.600072
H	-4.375169	2.737765	2.014605
C	-1.405822	3.493617	-2.346656
H	-1.612562	2.695335	-3.075393
H	-1.632124	4.452818	-2.828670
H	-0.334377	3.469920	-2.106027
C	-5.333761	4.835546	0.494249
H	-5.040794	5.896265	0.479578

H	-6.083365	4.695289	-0.300189
H	-5.806975	4.613298	1.459892
C	-2.289483	1.056338	1.820356
H	-1.801396	1.516508	2.694396
H	-3.184158	0.528302	2.175877
H	-1.629747	0.312632	1.365522
C	2.761911	-4.476010	-0.834931
H	3.337745	-4.355717	-1.767091
H	3.454576	-4.275425	-0.000976
H	2.425704	-5.518939	-0.764694

TS-13

ZPE	0.378618
DE	0.410088
DH	0.411032
DG	0.313421
E	-2520.673470
H	-2520.262438
Gibbs free energy	-2520.360049
Imaginary frequency	-195.124

Cartesian coordinates

C	0.823514	-2.665087	0.788677
N	0.290782	-1.408776	0.209358
C	0.131776	-0.362176	0.936789
C	0.367377	-0.269382	2.399421
H	0.045975	-1.184939	2.910166
C	-0.432500	0.974888	2.775275
C	-0.375686	1.810084	1.517306
H	-1.276207	2.396006	1.320171
H	0.544979	2.399626	1.435598
O	-0.307013	0.794582	0.437134
C	0.172389	1.501306	-1.287307
C	-0.151586	2.897129	-1.237035
C	0.888809	3.733965	-1.016574
H	0.760444	4.820063	-1.044446
H	1.887896	3.338637	-0.809109
H	1.216252	1.194691	-1.185252
H	-0.511925	0.845966	-1.825169

H	-0.002629	1.513876	3.627303
H	-1.472100	0.703967	2.997674
H	1.453733	-0.154321	2.544465
C	-0.203339	-1.614523	-1.165001
C	-0.327265	-3.130365	-1.255269
C	0.835062	-3.623370	-0.395769
H	0.722835	-4.664914	-0.069115
H	1.783604	-3.534647	-0.945857
H	-0.281361	-3.476557	-2.295628
H	-1.289805	-3.445339	-0.824427
H	-1.158430	-1.091926	-1.280634
H	0.536559	-1.229556	-1.879672
H	1.810488	-2.463407	1.224323
H	0.130158	-3.001562	1.574364
O	-4.847176	-0.472043	1.570774
S	-3.638321	-0.087445	0.846481
O	-2.484021	-0.984954	1.036432
O	-3.313414	1.347100	0.868477
C	-4.100394	-0.375426	-0.928974
F	-4.283676	-1.670006	-1.173796
F	-3.138891	0.059689	-1.756177
F	-5.218176	0.270760	-1.240378
C	4.029125	-0.355565	-0.959267
F	4.193764	0.601343	-1.869139
F	5.107989	-1.126729	-0.960101
F	2.987243	-1.102620	-1.342359
S	3.729424	0.376400	0.718039
O	3.483813	-0.803191	1.559652
O	4.951967	1.121997	0.993220
O	2.526580	1.207230	0.488065
C	-1.572049	3.319116	-1.484114
H	-1.873841	3.069716	-2.513478
H	-2.254079	2.782082	-0.805511
H	-1.697207	4.399456	-1.335003

TS-14

ZPE	0.547714
DE	0.590233
DH	0.591177
DG	0.465234

E -2886.565921

H -2885.974744

Gibbs free energy -2886.100687

Imaginary frequency -1290.512

Cartesian coordinates

C	-0.910773	-1.853222	2.100123
N	-0.968462	-0.530287	1.455896
C	-1.061409	-0.325251	0.170999
O	-1.269857	-1.472665	-0.578688
S	-2.740584	-2.065069	-0.931511
O	-3.081196	-1.735658	-2.290671
O	-3.641768	-1.821819	0.167414
O	3.956267	-0.017183	0.117888
S	2.872683	0.012636	1.100456
O	2.334237	1.346071	1.415815
O	1.857984	-1.045679	0.957420
C	3.729354	-0.447064	2.680124
F	4.744410	0.378222	2.923670
F	4.209630	-1.687088	2.622257
F	2.894131	-0.380541	3.720470
C	-2.210604	-3.854803	-0.936933
F	-1.925169	-4.244967	0.284124
F	-3.223764	-4.549131	-1.407503
F	-1.162905	-3.993282	-1.716727
C	-0.836982	0.880052	-0.526375
H	0.403940	0.799193	-1.042269
H	-0.748897	1.731960	0.153591
C	-1.649632	1.193238	-1.771802
C	-3.074474	1.623986	-1.463429
O	-3.032770	2.715453	-0.586549
C	-4.279840	3.121465	-0.113143
C	-4.156483	4.197224	0.932486
C	-2.971613	4.669870	1.330708
H	-2.904947	5.450718	2.094290
H	-2.040142	4.293642	0.901584
H	-4.830322	2.262009	0.325659
H	-4.917014	3.495760	-0.942670
H	-3.606056	1.887851	-2.399370

H	-3.638774	0.787787	-1.001339
H	-1.143299	2.005799	-2.316553
H	-1.677218	0.334541	-2.459335
C	-0.724036	0.558847	2.413787
C	-0.838443	-0.132164	3.767937
H	-0.247664	0.384975	4.535312
C	-0.338842	-1.547286	3.478777
H	-0.659284	-2.285298	4.225760
H	0.757676	-1.554121	3.422441
H	-1.888653	-0.153212	4.099219
H	-1.457575	1.362778	2.257742
H	0.289660	0.950935	2.236666
H	-0.277958	-2.526530	1.510460
H	-1.929728	-2.264896	2.162119
C	2.031618	-0.080071	-2.483186
C	3.164101	0.082985	-3.274623
C	3.793028	1.328041	-3.352986
C	3.234495	2.384066	-2.627256
C	2.097527	2.177004	-1.852316
N	1.531144	0.961369	-1.802307
H	3.560563	-0.773620	-3.823597
H	3.685745	3.377873	-2.658917
C	1.341674	-1.400284	-2.331823
H	0.277988	-1.324515	-2.598820
H	1.811442	-2.163752	-2.964597
H	1.405616	-1.714193	-1.278594
C	5.044035	1.515742	-4.158918
H	5.922718	1.286652	-3.533942
H	5.069557	0.840049	-5.025342
H	5.146709	2.552332	-4.508888
C	1.469438	3.270549	-1.043517
H	1.516279	2.995026	0.020419
H	2.000827	4.218987	-1.191969
H	0.413999	3.413602	-1.320290
C	-5.468301	4.683334	1.482074
H	-6.036528	3.854348	1.936080
H	-5.323472	5.460907	2.244970
H	-6.100773	5.099142	0.680039

29

ZPE	0.335518
DE	0.358749
DH	0.359693
DG	0.279344
E	-1559.345181
H	-1558.985488
Gibbs free energy	-1559.065837

Cartesian coordinates

C	-3.188037	-0.724406	-0.240958
N	-1.984487	-1.508653	0.038385
C	-0.968857	-1.105150	0.847288
C	-0.100310	-1.865870	1.537645
C	1.166990	-1.355326	2.154155
H	1.075011	-0.287190	2.404277
H	1.386517	-1.891311	3.090627
C	2.355294	-1.533958	1.212962
O	2.043927	-0.903213	0.000106
C	2.957478	-1.112460	-1.043610
C	4.287600	-0.426347	-0.820161
C	5.437181	-1.105231	-0.892086
H	6.404727	-0.609844	-0.761205
H	5.450845	-2.181365	-1.094172
H	3.111198	-2.195435	-1.216438
H	2.476901	-0.691547	-1.941963
H	2.550841	-2.612241	1.041313
H	3.269252	-1.105027	1.667796
H	-0.258121	-2.946312	1.511698
O	-0.894926	0.297776	0.978325
S	-0.099285	1.263348	-0.036068
O	-0.077414	0.733045	-1.376929
O	1.061681	1.810327	0.619960
C	-1.337466	2.653834	0.007815
F	-2.459933	2.314989	-0.594756
F	-0.793155	3.675762	-0.627804
F	-1.590445	2.993142	1.255593
C	-2.137414	-2.909188	-0.318725
C	-3.352285	-2.898841	-1.241479

H	-3.860605	-3.872071	-1.276993
C	-4.213580	-1.784484	-0.644807
H	-4.965656	-1.390839	-1.342344
H	-4.740539	-2.156260	0.248395
H	-3.043796	-2.636234	-2.266011
H	-1.220436	-3.289401	-0.797657
H	-2.328154	-3.532302	0.577594
H	-3.506151	-0.139998	0.635912
H	-3.002766	-0.020600	-1.068713
C	4.209965	1.043909	-0.526379
H	3.728015	1.578408	-1.362309
H	3.575675	1.227246	0.354326
H	5.203517	1.481915	-0.354711

SI.14

ZPE	0.454470
DE	0.477421
DH	0.478365
DG	0.402333
E	-925.114790
H	-924.636425
Gibbs free energy	-924.712457

Cartesian coordinates

C	0.834425	2.984443	0.241693
N	-0.211526	1.984685	0.113896
C	-0.064571	0.731430	0.638644
C	0.926449	0.289871	1.435145
C	1.126013	-1.118875	1.910411
C	2.455193	-1.714839	1.471088
O	2.436669	-1.915421	0.076423
C	3.683689	-2.193919	-0.499276
C	4.475108	-0.970834	-0.872655
C	4.036328	0.284451	-0.781567
H	4.663463	1.127799	-1.083683
H	3.035136	0.507263	-0.398798
H	5.480435	-1.174991	-1.260748
H	3.497475	-2.788223	-1.410795
H	4.291110	-2.838924	0.165363

H	3.283745	-1.040772	1.759128
H	2.620947	-2.680375	1.986184
H	1.106946	-1.142359	3.013636
H	0.315742	-1.781959	1.567912
C	-1.421261	2.500545	-0.524347
C	-1.160283	4.005805	-0.590858
H	-1.682400	4.480928	-1.432325
C	0.362981	4.100478	-0.687282
H	0.756969	5.083642	-0.396766
H	0.692766	3.892759	-1.717771
H	-1.508267	4.484772	0.338011
H	-2.327096	2.264775	0.055882
H	-1.549268	2.071258	-1.535848
H	1.810094	2.560667	-0.049639
H	0.926300	3.335279	1.287856
C	-2.169641	-0.429593	1.015837
C	-0.974393	-0.769333	-1.031232
C	-3.153351	-1.300548	0.585537
C	-1.963301	-1.638708	-1.462510
C	-3.071241	-1.927817	-0.663053
H	-4.004516	-1.487002	1.241571
H	-1.855106	-2.097646	-2.445997
N	-1.101941	-0.190436	0.198278
C	-2.238029	0.252772	2.343016
H	-2.061792	1.333508	2.246976
H	-3.226231	0.090378	2.789348
H	-1.473609	-0.150334	3.023618
C	0.237348	-0.471271	-1.845970
H	1.126969	-0.844938	-1.313255
H	0.160022	-0.967312	-2.819959
H	0.361326	0.610043	-1.998874
C	-4.125656	-2.890010	-1.106828
H	-5.126253	-2.537052	-0.820190
H	-4.092744	-3.055956	-2.191299
H	-3.965253	-3.859687	-0.607726
H	1.669151	1.023785	1.756823

SI.15

ZPE	0.190503
DE	0.208331
DH	0.209275
DG	0.142722
E	-1362.999994
H	-1362.790719
Gibbs free energy	-1362.857272

Cartesian coordinates

C	-2.011905	-1.297058	0.829931
N	-1.874669	-0.462869	-0.400778
C	-2.141434	0.766443	-0.404427
C	-2.505091	2.007771	-0.343784
C	-3.884929	2.524717	-0.612084
H	-3.843792	3.247307	-1.440131
H	-4.243177	3.058170	0.280428
H	-1.690990	2.696448	-0.082629
C	-1.294646	-1.239029	-1.516314
C	-0.735047	-2.495475	-0.840096
H	0.319480	-2.334506	-0.591155
C	-1.542456	-2.664709	0.444657
H	-2.455034	-3.265949	0.287946
H	-0.987740	-3.110813	1.279284
H	-0.816154	-3.364335	-1.503737
H	-2.102773	-1.462011	-2.226811
H	-0.528370	-0.625805	-2.003567
C	2.633322	0.059594	-0.373992
F	2.209934	-0.733518	-1.362183
F	3.469112	-0.644434	0.383686
F	3.312304	1.063771	-0.920517
S	1.207732	0.694844	0.627379
O	0.577988	-0.538369	1.128411
O	0.407222	1.415219	-0.382310
O	1.845950	1.534062	1.636456
H	-4.588281	1.721241	-0.862135
O	-2.492815	-0.858796	1.819652

SI.16

ZPE	0.192845
DE	0.209672
DH	0.210617
DG	0.145706
E	-1363.032443
H	-1362.821826
Gibbs free energy	-1362.886737

Cartesian coordinates

C	2.306036	-0.143930	-1.383896
N	1.839566	0.100149	-0.015422
C	0.701255	0.838876	0.301721
C	0.571981	2.162621	0.247985
C	1.669594	3.104083	-0.111581
H	1.749514	3.897776	0.647049
H	2.639980	2.595617	-0.192798
H	-0.411921	2.578751	0.478925
O	-0.383312	0.066643	0.777503
S	-1.292411	-0.776752	-0.246516
O	-1.441619	-2.109902	0.274007
O	-0.907358	-0.510403	-1.613015
C	-2.902292	0.103806	0.053813
F	-2.782649	1.371462	-0.290966
F	-3.231686	0.015496	1.324637
F	-3.826653	-0.472483	-0.689304
C	2.570730	-0.579292	0.941347
C	3.645413	-1.356516	0.207794
H	3.297961	-2.401187	0.135066
C	3.722133	-0.684089	-1.162255
H	4.436520	0.152624	-1.128247
H	4.032440	-1.363158	-1.966545
H	4.582557	-1.354916	0.779152
H	1.653731	-0.879981	-1.880888
H	2.283240	0.785025	-1.970371
H	1.450129	3.600224	-1.070896
O	2.350696	-0.544766	2.130615

SI.17

ZPE	0.211103
DE	0.227460
DH	0.228404
DG	0.164288
E	-1289.069377
H	-1288.840973
Gibbs free energy	-1288.905089

Cartesian coordinates

C	-2.698369	0.025430	0.949331
N	-1.954395	0.162228	-0.308135
C	-0.897251	1.016266	-0.430185
C	-0.848855	2.336536	-0.223470
C	-2.056997	3.189690	-0.011097
H	-1.940254	4.156763	-0.523226
H	-2.965455	2.700877	-0.392895
H	0.139856	2.800807	-0.194351
O	0.331503	0.392169	-0.828529
S	1.041514	-0.634470	0.178857
O	1.064252	-1.952836	-0.407585
O	0.612090	-0.410870	1.540239
C	2.756324	0.063055	0.017537
F	2.759516	1.323414	0.404324
F	3.160854	-0.021915	-1.232769
F	3.556840	-0.641678	0.794259
C	-2.204963	-0.950328	-1.225037
C	-2.804959	-2.017356	-0.314688
H	-1.992745	-2.563745	0.191800
C	-3.595256	-1.188858	0.699596
H	-4.551971	-0.867824	0.257250
H	-3.819651	-1.733040	1.627349
H	-3.420914	-2.745390	-0.860358
H	-2.915366	-0.652901	-2.017905
H	-1.278346	-1.279667	-1.716399
H	-1.996043	-0.151472	1.782385
H	-3.282421	0.928422	1.185833
H	-2.215835	3.410385	1.058496

SI.18

ZPE	0.209955
DE	0.227263
DH	0.228207
DG	0.161535
E	-1289.065075
H	-1288.836868
Gibbs free energy	-1288.903540

Cartesian coordinates

C	2.391597	-0.931583	-1.214012
N	2.255610	-0.089658	0.009137
C	2.021408	1.139494	-0.004553
C	1.765287	2.410454	-0.012997
C	2.768036	3.520288	0.050075
H	2.560144	4.145362	0.931004
H	2.661552	4.156548	-0.840767
H	0.692156	2.634115	-0.080258
C	2.312621	-0.920051	1.247845
C	2.215706	-2.342855	0.712418
H	1.155690	-2.611131	0.598468
C	2.883659	-2.261616	-0.662054
H	3.980385	-2.251181	-0.563583
H	2.605760	-3.092865	-1.322893
H	2.699514	-3.060336	1.387546
H	3.275443	-0.715017	1.737242
H	1.481704	-0.619933	1.897608
H	1.384502	-1.005721	-1.648488
H	3.067344	-0.438886	-1.922966
C	-2.906513	0.067913	-0.009999
F	-3.464835	-0.624044	0.980155
F	-3.437850	-0.349281	-1.156564
F	-3.226096	1.350259	0.150777
S	-1.068620	-0.157101	-0.016551
O	-0.902484	-1.603393	-0.208106
O	-0.664203	0.354704	1.301811
O	-0.633972	0.669812	-1.154797
H	3.797949	3.146615	0.107019

SI.TS-1

ZPE	0.519957
DE	0.560940
DH	0.561884
DG	0.439192
E	-2847.284366
H	-2846.722482
Gibbs free energy	-2846.845174
Imaginary frequency	-1311.685

Cartesian coordinates

C	1.290578	-2.204356	-1.475646
N	1.097892	-0.748016	-1.371559
C	1.203998	-0.054526	-0.271756
O	1.691048	-0.769186	0.812216
S	3.271502	-0.904827	1.163062
O	3.606485	-0.015044	2.243786
O	4.039342	-0.954975	-0.056253
O	-3.756772	-0.647411	0.277895
S	-2.748052	-0.802492	-0.770454
O	-2.523389	0.379491	-1.618963
O	-1.524062	-1.518976	-0.372336
C	-3.551967	-1.985176	-1.951872
F	-4.734826	-1.523611	-2.349747
F	-3.741603	-3.174784	-1.385634
F	-2.800232	-2.166395	-3.041143
C	3.137047	-2.611735	1.904821
F	2.842752	-3.488815	0.972873
F	4.309383	-2.889549	2.432249
F	2.209357	-2.604906	2.834772
C	0.759541	1.263741	-0.039198
H	-0.408606	1.164227	0.623819
H	0.455351	1.758854	-0.965440
C	1.548486	2.171541	0.890037
C	2.833894	2.693115	0.268035
O	2.521178	3.341740	-0.933631
C	3.631611	3.740300	-1.678057
C	3.217223	4.332739	-2.988011
C	1.959008	4.480519	-3.401750

H	1.733922	4.923014	-4.376119
H	1.116324	4.167285	-2.779154
H	4.049050	4.656905	-3.624240
H	4.306991	2.879408	-1.869149
H	4.241346	4.482021	-1.119709
H	3.345331	3.383247	0.968190
H	3.536913	1.856596	0.075402
H	0.909668	3.028622	1.155828
H	1.790578	1.664477	1.836167
C	0.586206	-0.170431	-2.623862
C	0.789579	-1.292282	-3.635879
H	0.073028	-1.222160	-4.464709
C	0.611280	-2.554965	-2.793641
H	1.048763	-3.451724	-3.251665
H	-0.456128	-2.736364	-2.611027
H	1.806724	-1.248316	-4.056221
H	1.136682	0.751621	-2.859946
H	-0.479676	0.067562	-2.482527
H	0.835222	-2.701542	-0.611450
H	2.369386	-2.421635	-1.500582
C	-1.732121	0.626481	2.482045
C	-2.826678	0.877932	3.304128
C	-3.701651	1.925266	3.007600
C	-3.424954	2.702256	1.878599
C	-2.314962	2.419995	1.089117
N	-1.504274	1.399199	1.410124
H	-2.999035	0.241570	4.174459
H	-4.076731	3.534813	1.605827
C	-0.786271	-0.507397	2.730350
H	0.248233	-0.147012	2.822795
H	-1.054612	-1.049218	3.645973
H	-0.830174	-1.198753	1.874771
C	-4.916029	2.187147	3.848107
H	-5.785067	1.667390	3.412556
H	-4.785124	1.815518	4.873781
H	-5.156717	3.259098	3.882502
C	-1.981637	3.213353	-0.137275
H	-2.023579	2.547081	-1.011557
H	-2.696848	4.033728	-0.276550
H	-0.968947	3.638879	-0.071102

SI.TS-2

ZPE	0.306662
DE	0.328336
DH	0.329280
DG	0.252703
E	-1520.048622
H	-1519.719342
Gibbs free energy	-1519.795920
Imaginary frequency	-164.177

Cartesian coordinates

C	-3.225906	1.816257	-0.480650
N	-2.254532	1.368089	0.539905
C	-2.254493	0.192056	1.073966
C	-2.705088	-0.643006	1.985806
C	-2.263038	-2.071120	1.937085
H	-3.066102	-2.691533	1.505263
C	-1.003373	-2.077172	1.073457
H	-0.097137	-1.911838	1.669383
H	-0.890573	-2.991998	0.478962
O	-1.105605	-0.943176	0.206203
C	-1.309320	-1.151017	-1.200316
H	-1.293726	-0.138328	-1.627831
H	-0.423023	-1.683441	-1.572899
C	-2.587599	-1.868027	-1.499314
C	-2.641949	-3.041235	-2.129637
H	-3.596250	-3.527353	-2.351464
H	-1.730924	-3.552604	-2.458957
H	-3.512247	-1.371705	-1.178856
H	-2.056303	-2.457567	2.944535
H	-3.390122	-0.260637	2.746012
C	-1.120551	2.311786	0.676521
H	-0.955602	2.543219	1.737861
H	-0.215963	1.837739	0.267845
C	-1.567651	3.510883	-0.155081
H	-2.159252	4.207255	0.460062
C	-2.450169	2.886872	-1.238963
H	-3.116294	3.610150	-1.728058
H	-1.823133	2.417851	-2.013676

H	-0.710122	4.061617	-0.563921
H	-3.546794	0.969628	-1.101577
H	-4.109161	2.237156	0.026043
C	3.663700	0.162075	-0.079479
S	1.852156	-0.225313	-0.000311
O	1.286948	0.705145	-0.991124
O	1.522099	0.066015	1.401327
O	1.807224	-1.644378	-0.380402
F	4.145422	-0.041535	-1.305303
F	3.894255	1.434548	0.243764
F	4.354463	-0.605033	0.763595

SI.TS-3

ZPE	0.378606
DE	0.409556
DH	0.410500
DG	0.313757
E	-2520.676009
H	-2520.265509
Gibbs free energy	-2520.362252
Imaginary frequency	-97.187

Cartesian coordinates

C	-3.485807	-1.444854	1.049922
N	-3.461841	-0.962505	-0.338851
C	-2.320119	-0.699040	-0.945377
O	-1.267464	-0.955761	-0.282436
S	0.652059	-0.605927	-0.782162
O	0.441914	0.821153	-0.756209
O	0.644300	-1.500774	-1.909103
C	0.819106	-1.551537	0.901838
F	0.226711	-2.709596	0.739912
F	2.067562	-1.759037	1.222852
F	0.236011	-0.836838	1.828089
C	-2.292687	-0.217502	-2.375869
H	-2.825968	-0.972614	-2.975940
H	-1.252195	-0.233884	-2.716882
C	-2.900374	1.165089	-2.644873
C	-2.278102	2.266508	-1.811374

O	-2.502130	1.956557	-0.462154
C	-1.820921	2.772069	0.458382
C	-2.085385	2.254517	1.847137
C	-1.076858	1.948459	2.669138
H	-1.259751	1.591627	3.687765
H	-0.034623	2.042224	2.351204
H	-2.186130	3.817592	0.374148
H	-0.737973	2.776241	0.245987
H	-1.192927	2.337561	-2.014181
H	-2.730074	3.243856	-2.071193
H	-2.748755	1.394275	-3.710060
H	-3.987256	1.157430	-2.478839
C	-4.811219	-0.826163	-0.894367
C	-5.650443	-1.691914	0.039438
H	-6.709155	-1.400939	0.029565
C	-4.973015	-1.482938	1.394306
H	-5.205898	-2.270243	2.123281
H	-5.283398	-0.518723	1.826104
H	-5.581548	-2.748167	-0.265611
S	3.341829	0.731740	-0.185631
O	2.818734	1.050351	1.130942
O	3.614964	1.792545	-1.130967
C	4.968173	-0.119266	0.092019
F	5.470525	-0.537177	-1.056598
F	5.802749	0.750968	0.639491
F	4.824714	-1.149734	0.906147
O	2.586240	-0.456634	-0.837268
H	-3.019547	-2.441855	1.093379
H	-2.897156	-0.772190	1.689692
H	-4.841515	-1.152818	-1.942296
H	-5.127787	0.227858	-0.844679
C	-3.533224	2.112677	2.220648
H	-3.659132	1.695418	3.229977
H	-4.043462	3.089937	2.178793
H	-4.046753	1.458545	1.499135

SI.TS-4

ZPE	0.335181
DE	0.357968
DH	0.358912

DG	0.280981
E	-1559.330697
H	-1558.971785
Gibbs free energy	-1559.049716
Imaginary frequency	-138.336

Cartesian coordinates

C	-2.018105	-1.171749	-1.433581
N	-2.500617	0.112143	-0.894052
C	-1.738712	1.147588	-0.692765
C	-1.180221	2.203939	-1.246131
C	-0.214280	2.990114	-0.425784
H	0.810856	2.745633	-0.748309
C	-0.466239	2.559354	1.011927
H	-1.215909	3.202133	1.501780
H	0.452756	2.500148	1.606388
O	-1.006554	1.235340	0.902134
C	-1.672025	0.716500	2.064221
H	-2.684918	1.145693	2.125344
H	-1.084292	1.073317	2.925097
C	-1.681136	-0.786717	2.061385
C	-2.819266	-1.443957	2.307144
H	-2.837251	-2.535039	2.383915
H	-3.766255	-0.915849	2.454388
H	-0.350700	4.073058	-0.548699
H	-1.400027	2.431025	-2.290698
C	-3.954567	0.098123	-0.641768
H	-4.439976	0.833499	-1.303046
H	-4.174878	0.367128	0.400968
C	-4.358156	-1.337035	-0.981821
H	-5.381463	-1.392005	-1.376723
C	-3.295262	-1.791845	-1.985508
H	-3.511055	-1.383956	-2.985872
H	-3.222370	-2.884514	-2.069361
H	-4.307892	-1.962303	-0.077128
H	-1.595458	-1.783597	-0.621146
H	-1.229874	-0.992769	-2.174980
C	-0.355434	-1.455269	1.849342
H	0.370408	-1.125480	2.610073

H	0.077120	-1.169174	0.878778
H	-0.449649	-2.549184	1.899424
C	3.464867	-0.998793	-0.164648
F	3.757334	-1.495538	-1.365455
F	4.602887	-0.605820	0.404951
F	2.958454	-1.987666	0.573976
S	2.261758	0.404136	-0.295502
O	2.967621	1.388550	-1.124711
O	1.103903	-0.230612	-0.943611
O	2.075050	0.793066	1.109047

SI.19

ZPE	0.336951
DE	0.359904
DH	0.360849
DG	0.282095
E	-1559.332833
H	-1558.971984
Gibbs free energy	-1559.050738

Cartesian coordinates

C	-3.434199	1.982180	0.277109
N	-2.191287	1.231968	0.096132
C	-1.989384	0.131390	0.845114
C	-2.547830	-0.425024	1.923261
C	-1.951336	-1.775485	2.211468
H	-2.602923	-2.603576	1.886306
C	-0.653298	-1.711452	1.427980
H	0.166495	-1.236354	1.977426
H	-0.305314	-2.630174	0.949776
O	-0.947731	-0.740416	0.335611
C	-1.069287	-1.227447	-1.079232
H	-1.042427	-0.305582	-1.666230
H	-0.129249	-1.772305	-1.218301
C	-2.311370	-2.037963	-1.308429
C	-3.424950	-1.412112	-1.707734
H	-4.351330	-1.963348	-1.894349
H	-3.440384	-0.329913	-1.866594
H	-1.735747	-1.915234	3.279774

H	-3.318037	0.061689	2.518180
C	-1.072905	2.114661	-0.303288
H	-0.209146	1.978297	0.362213
H	-0.735683	1.864340	-1.321266
C	-1.687786	3.512874	-0.245639
H	-1.555347	3.941976	0.760176
C	-3.173498	3.261096	-0.509440
H	-3.821590	4.087145	-0.186710
H	-3.349307	3.086538	-1.583156
H	-1.225248	4.198175	-0.968951
H	-4.293463	1.397176	-0.085617
H	-3.603536	2.214020	1.345465
C	3.927094	0.226550	-0.073783
S	2.096677	-0.061906	-0.066693
O	1.649037	0.601918	-1.296405
O	1.671369	0.573452	1.190849
O	1.995683	-1.530676	-0.082677
F	4.487942	-0.299304	-1.161214
F	4.201655	1.529671	-0.058821
F	4.501104	-0.326714	0.993064
C	-2.230270	-3.525560	-1.103768
H	-1.418164	-3.954309	-1.712682
H	-2.014084	-3.784792	-0.055327
H	-3.172389	-4.016396	-1.383975

SI.TS-5-chair

ZPE	0.334539
DE	0.357275
DH	0.358220
DG	0.279539
E	-1559.319722
H	-1558.961502
Gibbs free energy	-1559.040182
Imaginary frequency	-266.635

Cartesian coordinates

C	1.069677	2.272730	1.243161
N	1.484300	1.506918	0.072199
C	2.455019	0.587179	0.184602

C	3.178704	0.130883	1.240129
C	4.159542	-0.907184	0.761657
H	3.926854	-1.925172	1.112583
C	4.024138	-0.764467	-0.753167
H	4.772370	-0.077973	-1.172454
H	4.022876	-1.697730	-1.326522
O	2.727588	-0.124710	-0.964120
C	1.270439	-1.470706	-1.209843
H	0.495330	-0.697599	-1.251596
H	1.666204	-1.825166	-2.165464
C	1.347131	-2.290767	-0.051938
C	0.845660	-1.742124	1.091580
H	0.953440	-2.254148	2.052955
H	0.246810	-0.825805	1.070937
H	5.190096	-0.682043	1.074241
H	3.158219	0.567299	2.236326
C	1.126227	2.169206	-1.189539
H	1.975321	2.168006	-1.888016
H	0.278503	1.647045	-1.659373
C	0.720552	3.572253	-0.739337
H	1.608248	4.222919	-0.682090
C	0.145365	3.337648	0.658444
H	0.116195	4.245884	1.275774
H	-0.876564	2.937778	0.585536
H	0.002354	4.035335	-1.430014
H	0.560513	1.621533	1.968613
H	1.952475	2.727006	1.732203
C	-3.373340	-1.001008	0.073889
S	-2.227713	0.444503	-0.093985
O	-1.496193	0.415629	1.182373
O	-3.120146	1.589837	-0.277042
O	-1.434729	0.073539	-1.279762
F	-2.680963	-2.125907	0.256288
F	-4.187999	-0.846765	1.114895
F	-4.121289	-1.151356	-1.016688
C	2.041518	-3.628605	-0.091889
H	3.019573	-3.574878	-0.591723
H	2.189764	-4.027429	0.920850
H	1.431039	-4.353667	-0.652941

SI.TS-5-boat

ZPE	0.334998
DE	0.357414
DH	0.358358
DG	0.281566
E	-1559.317568
H	-1558.959209
Gibbs free energy	-1559.036002
Imaginary frequency	-252.311

Cartesian coordinates

C	4.391030	-0.122236	1.152272
N	3.518360	-0.346474	0.010497
C	2.321952	-0.944054	0.163159
C	1.698232	-1.519825	1.216617
C	0.329798	-1.979900	0.791235
H	-0.492028	-1.396862	1.235155
C	0.411782	-1.804348	-0.724024
H	0.721633	-2.726135	-1.235248
H	-0.488020	-1.399886	-1.198722
O	1.497884	-0.844378	-0.936852
C	0.819950	1.051908	-1.055907
H	1.280514	1.219984	-2.032571
H	-0.222151	0.711344	-1.058581
C	1.325749	1.724638	0.091767
C	0.718361	1.424787	1.273375
H	1.095416	1.826759	2.218913
H	-0.205698	0.838242	1.304364
H	0.146975	-3.034808	1.043274
H	2.172069	-1.734434	2.172401
C	4.231166	-0.297198	-1.270331
H	4.039006	-1.202170	-1.865686
H	3.905673	0.571527	-1.867495
C	5.696647	-0.167621	-0.848236
H	6.142833	-1.168439	-0.737066
C	5.620436	0.520400	0.516386
H	6.525256	0.382037	1.123378
H	5.456819	1.602513	0.392015
H	6.293890	0.388456	-1.583550

H	3.890204	0.519195	1.896155
H	4.648670	-1.076476	1.649553
C	-4.583386	0.396694	-0.081931
S	-2.872626	-0.303529	0.034219
O	-2.230826	0.210627	-1.191563
O	-3.087217	-1.751568	0.056715
O	-2.358863	0.279829	1.283831
F	-4.551096	1.726707	-0.116721
F	-5.193920	-0.030876	-1.184281
F	-5.315899	0.028241	0.966025
C	2.512063	2.647793	-0.002964
H	3.178051	2.370238	-0.830568
H	2.170782	3.678850	-0.188387
H	3.091337	2.652899	0.930915

SI.20

ZPE	0.338576
DE	0.361348
DH	0.362292
DG	0.283325
E	-1559.432961
H	-1559.070669
Gibbs free energy	-1559.149636

Cartesian coordinates

C	1.284552	1.845671	-1.211449
N	1.223138	1.781423	0.258594
C	1.344784	0.699637	0.959626
O	1.245335	0.770347	2.252423
C	1.350728	-0.553185	2.838855
H	0.562652	-0.630517	3.596326
C	1.187608	-1.513800	1.667306
H	1.786011	-2.422237	1.802286
H	0.132220	-1.788631	1.550082
C	1.629641	-0.679930	0.452159
H	0.973009	-0.862943	-0.410398
C	3.113704	-0.823541	0.032590
C	3.321920	-1.885145	-1.022225
C	3.842472	-1.569523	-2.213473

H	4.002399	-2.329691	-2.984400
H	4.127823	-0.541620	-2.458797
H	3.488142	0.135301	-0.354686
H	3.713650	-1.059444	0.928801
H	2.334410	-0.611971	3.325951
O	-1.347650	0.300044	0.993270
S	-1.933751	0.165506	-0.351530
O	-1.028839	-0.403470	-1.364852
O	-2.736627	1.305810	-0.798922
C	-3.178046	-1.185032	-0.112570
F	-4.097740	-0.831899	0.781643
F	-3.798082	-1.469968	-1.254304
F	-2.586564	-2.297944	0.323516
C	0.774517	3.054945	0.850982
C	0.689205	3.995292	-0.349057
H	-0.089383	4.756463	-0.209834
C	0.409373	3.053915	-1.521554
H	0.644058	3.491770	-2.500356
H	-0.646913	2.741964	-1.519738
H	1.650197	4.512651	-0.495228
H	1.474765	3.383809	1.630300
H	-0.209364	2.873762	1.310448
H	0.908825	0.913060	-1.648896
H	2.330238	2.005674	-1.518000
C	2.910880	-3.288867	-0.674050
H	3.397954	-3.623165	0.257038
H	1.822772	-3.353616	-0.507074
H	3.170284	-3.994867	-1.475388

SI.TS-6

ZPE	0.422933
DE	0.450809
DH	0.451753
DG	0.361101
E	-1865.275121
H	-1864.823368
Gibbs free energy	-1864.914019
Imaginary frequency	-352.993

Cartesian coordinates

C	0.470110	-1.024078	-0.628957
N	0.186503	-0.272005	0.617217
C	-0.014007	1.002808	0.646968
C	1.448552	1.872081	1.979600
H	1.471997	0.981866	2.611165
H	0.924012	2.732670	2.400573
C	2.315214	2.044524	0.934367
C	2.360271	3.373112	0.201592
O	1.630853	3.304050	-1.004287
C	0.365513	3.912150	-1.005204
C	-0.588246	3.432705	0.097765
H	-1.559296	3.914281	-0.082129
H	-0.237173	3.797587	1.073923
C	-0.779594	1.955273	0.144731
H	-1.692589	1.514497	-0.286567
H	-0.065271	3.694208	-1.992559
H	0.462799	5.010554	-0.914003
H	1.987498	4.177317	0.856219
H	3.404266	3.615857	-0.049110
C	0.008040	-1.167953	1.772659
C	-0.144455	-2.539573	1.124202
H	0.158744	-3.349678	1.800816
C	0.724902	-2.441931	-0.131890
H	0.461895	-3.188809	-0.892878
H	1.788199	-2.568462	0.122108
H	-1.196370	-2.693568	0.841071
H	-0.874812	-0.852110	2.341505
H	0.903969	-1.115264	2.412807
H	1.317987	-0.574306	-1.159941
H	-0.432814	-0.963589	-1.256259
C	-5.129348	-0.405141	-0.333252
F	-5.285107	0.912320	-0.207262
F	-5.877266	-0.997621	0.595392
F	-5.596657	-0.761533	-1.527697
S	-3.347727	-0.875931	-0.147054
O	-3.023675	-0.399425	1.203678
O	-2.705287	-0.121747	-1.240525
O	-3.357648	-2.331253	-0.324520
C	3.150788	0.950157	0.422132

C	3.448582	-0.175755	1.204084
C	3.644906	0.974719	-0.900322
C	4.184014	-1.245651	0.704097
H	3.119278	-0.222942	2.243630
C	4.361535	-0.088314	-1.416843
H	3.419306	1.820869	-1.550937
C	4.635579	-1.216624	-0.622263
H	4.396999	-2.094692	1.353303
H	4.721541	-0.075540	-2.447294
O	5.329986	-2.200854	-1.205597
C	5.620149	-3.371986	-0.472882
H	6.175441	-4.032125	-1.150097
H	6.245909	-3.153536	0.408165
H	4.699016	-3.883950	-0.148548

SI.TS-7-chair

ZPE	0.421767
DE	0.449869
DH	0.450813
DG	0.359427
E	-1865.268886
H	-1864.818073
Gibbs free energy	-1864.909458
Imaginary frequency	-275.813

Cartesian coordinates

C	2.620463	-2.447561	1.217254
N	1.751175	-2.312133	0.052478
C	0.431577	-2.518718	0.174230
C	-0.364204	-2.798830	1.240878
C	-1.783424	-2.980238	0.773283
H	-2.473855	-2.212827	1.153139
C	-1.616984	-2.914310	-0.744804
H	-1.569884	-3.910257	-1.205352
H	-2.354249	-2.299345	-1.273335
O	-0.305144	-2.307495	-0.968352
C	-0.455388	-0.338116	-1.232793
H	0.629850	-0.192030	-1.243958
H	-0.940465	-0.450055	-2.204701

C	-1.174724	0.104937	-0.089316
C	-0.438300	0.152712	1.062966
H	-0.892422	0.395058	2.027318
H	0.651410	0.059560	1.038340
H	-2.191285	-3.958834	1.068331
H	0.005547	-3.045882	2.233810
C	2.485337	-2.425889	-1.215320
H	1.965377	-3.100663	-1.910353
H	2.579405	-1.434095	-1.683575
C	3.851476	-2.951604	-0.775851
H	3.833783	-4.052108	-0.720124
C	4.022828	-2.354084	0.621766
H	4.768502	-2.881128	1.232477
H	4.322928	-1.298397	0.549695
H	4.649336	-2.658355	-1.472002
H	2.415612	-1.649045	1.945055
H	2.451662	-3.426325	1.705073
C	2.640103	3.065949	0.126948
S	3.155111	1.294122	-0.031856
O	2.678870	0.713754	1.233894
O	4.609256	1.354941	-0.182921
O	2.418353	0.864420	-1.233834
F	1.318305	3.155860	0.276606
F	3.213116	3.636322	1.184006
F	2.979815	3.764311	-0.953831
C	-2.645394	0.350346	-0.139333
C	-3.272800	0.726996	-1.339256
C	-3.449409	0.242424	1.000777
C	-4.637256	0.969485	-1.397308
H	-2.685216	0.854996	-2.251174
C	-4.822023	0.483257	0.961060
H	-3.009144	-0.048936	1.957164
C	-5.430279	0.847645	-0.246552
H	-5.113651	1.267847	-2.333348
H	-5.404916	0.379431	1.876459
O	-6.743042	1.097943	-0.392461
C	-7.587561	1.012084	0.731991
H	-8.596859	1.265416	0.384573
H	-7.601600	-0.006913	1.154463
H	-7.291576	1.726000	1.519025

SI.TS-7-boat

ZPE	0.421678
DE	0.449857
DH	0.450801
DG	0.359648
E	-1865.268770
H	-1864.817969
Gibbs free energy	-1864.909123
Imaginary frequency	-264.695

Cartesian coordinates

C	2.906163	2.095057	-1.139235
N	1.972895	2.013429	-0.027034
C	0.650593	2.143019	-0.220700
C	-0.110831	2.389945	-1.314841
C	-1.561095	2.426988	-0.912427
H	-2.161167	1.601656	-1.324667
C	-1.456319	2.368856	0.611564
H	-1.490817	3.365326	1.072319
H	-2.174913	1.706999	1.105254
O	-0.112454	1.850616	0.885249
C	-0.101623	-0.142155	1.156466
H	0.383873	-0.058462	2.129773
H	-1.196776	-0.181593	1.165588
C	0.578757	-0.687583	0.028876
C	-0.146932	-0.655877	-1.128631
H	0.267387	-0.964218	-2.091050
H	-1.210631	-0.402186	-1.115683
H	-2.051185	3.362383	-1.221160
H	0.283733	2.686868	-2.284500
C	2.620030	2.187036	1.276573
H	2.107172	2.955632	1.873151
H	2.599841	1.243379	1.846092
C	4.050960	2.580083	0.905578
H	4.126834	3.675168	0.814422
C	4.262222	1.923917	-0.460144
H	5.079120	2.376171	-1.038465
H	4.481292	0.852276	-0.336179
H	4.778354	2.251334	1.660279

H	2.686794	1.310730	-1.881764
H	2.826323	3.074662	-1.646975
C	-5.466838	-1.329705	0.072725
S	-4.059783	-0.134396	-0.073218
O	-3.246703	-0.441057	1.119570
O	-4.709930	1.177451	-0.054290
O	-3.434825	-0.513418	-1.350990
F	-5.025303	-2.584919	0.071504
F	-6.141847	-1.129550	1.201834
F	-6.311736	-1.187071	-0.945259
C	2.002962	-1.127231	0.096525
C	2.642380	-1.353672	1.327422
C	2.750147	-1.363210	-1.063244
C	3.965427	-1.765965	1.394517
H	2.101569	-1.222566	2.266720
C	4.077754	-1.784724	-1.015341
H	2.306064	-1.209655	-2.048213
C	4.703064	-1.981549	0.222038
H	4.449134	-1.936761	2.358271
H	4.614624	-1.947315	-1.950064
O	5.980432	-2.372227	0.376836
C	6.772433	-2.592117	-0.767209
H	7.764756	-2.891521	-0.407687
H	6.363177	-3.399556	-1.397637
H	6.873985	-1.676124	-1.373868

SI.TS-8

ZPE	0.390511
DE	0.415742
DH	0.416686
DG	0.332518
E	-1750.866587
H	-1750.449901
Gibbs free energy	-1750.534069
Imaginary frequency	-367.682

Cartesian coordinates

C	0.566878	-1.196787	-0.987964
N	0.497185	-0.594002	0.363455

C	0.551672	0.681331	0.567175
C	2.106750	1.094730	1.931372
H	1.964952	0.159260	2.476434
H	1.748634	1.991864	2.440168
C	3.003447	1.198701	0.902824
C	3.317754	2.547916	0.283734
O	2.583610	2.733950	-0.907393
C	1.469010	3.584379	-0.829281
C	0.447148	3.216635	0.255894
H	-0.409009	3.895164	0.140390
H	0.875711	3.425919	1.247449
C	-0.032618	1.807956	0.189918
H	-1.031724	1.594290	-0.223497
H	0.995331	3.540295	-1.819869
H	1.789510	4.628370	-0.653019
H	3.123153	3.352432	1.011114
H	4.387633	2.588494	0.028325
C	0.244723	-1.604878	1.404062
C	-0.111836	-2.850039	0.597447
H	0.123348	-3.773576	1.143000
C	0.692892	-2.685046	-0.694502
H	0.306701	-3.296189	-1.521211
H	1.749178	-2.950073	-0.531874
H	-1.188348	-2.837655	0.369775
H	-0.567007	-1.258473	2.055601
H	1.163135	-1.752010	1.994863
H	1.407337	-0.769741	-1.548084
H	-0.376792	-0.956834	-1.503005
C	-4.818430	0.192901	-0.117294
F	-4.808953	1.491398	0.181294
F	-5.565208	-0.431952	0.790835
F	-5.405838	0.048458	-1.303178
S	-3.096290	-0.489129	-0.140563
O	-2.624201	-0.232167	1.225904
O	-2.445250	0.325387	-1.184677
O	-3.293465	-1.898444	-0.494240
C	3.631696	0.003418	0.304792
C	3.831444	-1.165159	1.058424
C	4.032924	0.009547	-1.043740
C	4.401881	-2.296664	0.481006

H	3.567063	-1.185516	2.117435
C	4.586577	-1.127513	-1.623136
H	3.869613	0.900789	-1.651989
C	4.772964	-2.284029	-0.863235
H	4.558352	-3.192659	1.085528
H	4.874707	-1.111798	-2.676441
H	5.214046	-3.173628	-1.318553

SI.TS-9-chair

ZPE	0.389109
DE	0.414544
DH	0.415488
DG	0.330841
E	-1750.862920
H	-1750.447432
Gibbs free energy	-1750.532079
Imaginary frequency	-272.467

Cartesian coordinates

C	-1.697273	2.685479	1.156060
N	-0.835129	2.393975	0.014541
C	0.497026	2.393910	0.165641
C	1.302813	2.565482	1.248352
C	2.742561	2.529721	0.811370
H	3.306755	1.678877	1.220213
C	2.601585	2.457670	-0.709070
H	2.717342	3.438653	-1.189192
H	3.248707	1.726785	-1.207060
O	1.217885	2.052377	-0.955025
C	1.065952	0.079950	-1.186454
H	-0.027973	0.107784	-1.227585
H	1.591147	0.091559	-2.143757
C	1.676540	-0.447234	-0.016593
C	0.922676	-0.357069	1.121715
H	1.317842	-0.652682	2.097288
H	-0.139995	-0.099941	1.073401
H	3.281382	3.444842	1.100005
H	0.953095	2.884901	2.227830
C	-1.515369	2.597642	-1.272079

H	-0.880255	3.168317	-1.964719
H	-1.754886	1.624315	-1.727223
C	-2.789897	3.340830	-0.873149
H	-2.598683	4.425415	-0.832851
C	-3.083683	2.803193	0.528306
H	-3.749773	3.451911	1.113293
H	-3.544599	1.806652	0.463681
H	-3.608910	3.165171	-1.584175
H	-1.636582	1.877935	1.899968
H	-1.386222	3.634000	1.633487
C	-2.568131	-2.771949	0.142549
S	-2.776851	-0.943776	-0.067496
O	-2.241663	-0.418885	1.199029
O	-4.216505	-0.765620	-0.258829
O	-1.948387	-0.672997	-1.256082
F	-1.283504	-3.075595	0.330618
F	-3.252895	-3.213353	1.194722
F	-2.993067	-3.430681	-0.932844
C	3.089201	-0.932006	-0.027190
C	3.639644	-1.484686	-1.192799
C	3.889672	-0.876006	1.123676
C	4.952730	-1.953046	-1.211809
H	3.032605	-1.572278	-2.096954
C	5.201205	-1.344932	1.105833
H	3.490832	-0.449300	2.046871
C	5.739980	-1.882693	-0.063423
H	5.358506	-2.383511	-2.130186
H	5.807509	-1.285348	2.012587
H	6.768848	-2.249332	-0.077603

SI.TS-9-boat

ZPE	0.389061
DE	0.414663
DH	0.415608
DG	0.329914
E	-1750.863806
H	-1750.448199
Gibbs free energy	-1750.533892
Imaginary frequency	-263.363

Cartesian coordinates

C	3.603286	-1.350578	1.085774
N	2.657642	-1.446302	-0.015724
C	1.430250	-1.956955	0.171729
C	0.791537	-2.470413	1.252309
C	-0.593878	-2.903601	0.851737
H	-1.402776	-2.318736	1.316478
C	-0.555533	-2.705296	-0.663240
H	-0.332997	-3.632401	-1.208355
H	-1.443261	-2.219185	-1.083829
O	0.590630	-1.819643	-0.908033
C	0.054633	0.095216	-1.044988
H	0.528119	0.226147	-2.019122
H	-1.007823	-0.169970	-1.066542
C	0.571846	0.728161	0.123159
C	-0.082349	0.401371	1.277188
H	0.254232	0.742802	2.258437
H	-1.032329	-0.140825	1.243107
H	-0.773528	-3.962800	1.090749
H	1.277156	-2.703517	2.197951
C	3.300446	-1.341930	-1.329589
H	2.996791	-2.171125	-1.984920
H	3.019622	-0.396143	-1.821951
C	4.791510	-1.366542	-0.990214
H	5.155508	-2.406089	-0.977175
C	4.848519	-0.773363	0.418755
H	5.768641	-1.027958	0.961690
H	4.769526	0.323780	0.372533
H	5.390919	-0.806353	-1.720699
H	3.197513	-0.708639	1.884260
H	3.799882	-2.348818	1.519794
C	-4.023028	1.371355	-0.020076
S	-3.837911	-0.472663	-0.062395
O	-3.037393	-0.679645	-1.286028
O	-5.211432	-0.963072	-0.127230
O	-3.120325	-0.752964	1.192823
F	-2.829049	1.964734	0.027414
F	-4.656761	1.811572	-1.103606
F	-4.714979	1.755003	1.049133

C	1.809590	1.564612	0.091607
C	2.315992	2.063651	-1.117490
C	2.481638	1.906988	1.275556
C	3.464186	2.854738	-1.145869
H	1.807270	1.852503	-2.059908
C	3.624253	2.702075	1.249462
H	2.121314	1.544709	2.239678
C	4.126223	3.176562	0.037095
H	3.835690	3.228400	-2.102757
H	4.130003	2.947311	2.186180
H	5.024143	3.798148	0.016646

SI.21

ZPE	0.425824
DE	0.453562
DH	0.454506
DG	0.364523
E	-1865.301619
H	-1864.847113
Gibbs free energy	-1864.937095

Cartesian coordinates

C	0.251506	-1.144683	-0.542370
N	0.366659	-0.436426	0.730271
C	0.279597	0.926178	0.821102
C	1.175999	1.563125	1.911974
H	1.402087	0.825167	2.689197
H	0.715773	2.445359	2.369086
C	2.294102	1.899810	1.014273
C	2.409681	3.317632	0.481717
O	1.944234	3.405119	-0.843234
C	0.619894	3.846447	-1.022004
C	-0.412526	3.193107	-0.088574
H	-1.401812	3.530183	-0.432400
H	-0.281054	3.601281	0.925505
C	-0.388243	1.703293	-0.068122
H	-1.030631	1.186565	-0.788402
H	0.375700	3.621840	-2.069748
H	0.561645	4.942473	-0.883692

H	1.886854	4.016403	1.152898
H	3.471663	3.605161	0.483760
C	0.013328	-1.299039	1.864806
C	-0.372714	-2.626506	1.207350
H	-0.124951	-3.493312	1.835876
C	0.373037	-2.605226	-0.128158
H	-0.061846	-3.285939	-0.872991
H	1.432859	-2.875156	0.008930
H	-1.454719	-2.631262	1.011065
H	-0.826050	-0.869141	2.429721
H	0.868784	-1.422400	2.554520
H	1.042813	-0.808990	-1.231786
H	-0.723979	-0.963895	-1.024109
C	-5.213314	-0.249270	-0.274915
F	-5.379556	1.065327	-0.128888
F	-5.831464	-0.853572	0.739258
F	-5.822417	-0.615499	-1.401845
S	-3.413270	-0.690534	-0.312037
O	-2.934931	-0.214880	0.991628
O	-2.924957	0.066273	-1.477696
O	-3.430225	-2.148177	-0.485640
C	3.180401	0.905844	0.531589
C	3.214682	-0.394240	1.101225
C	3.993608	1.138836	-0.621565
C	3.974063	-1.412858	0.553431
H	2.687404	-0.595751	2.031127
C	4.733454	0.131331	-1.180763
H	3.991661	2.116777	-1.103276
C	4.723027	-1.166622	-0.609572
H	3.985530	-2.390697	1.033216
H	5.333829	0.298483	-2.076405
O	5.458914	-2.075727	-1.227635
C	5.492125	-3.410235	-0.747563
H	6.139516	-3.965662	-1.435498
H	5.915474	-3.455946	0.267857
H	4.485575	-3.856737	-0.753028

SI.22

ZPE	0.393058
DE	0.418332
DH	0.419276
DG	0.333986
E	-1750.888555
H	-1750.469278
Gibbs free energy	-1750.554568

Cartesian coordinates

C	0.437795	-1.447569	-0.845718
N	0.665569	-0.924567	0.500860
C	0.792281	0.399604	0.768716
C	1.737325	0.764330	1.930737
H	1.914619	-0.101248	2.576113
H	1.394551	1.620080	2.520071
C	2.796858	1.096625	0.969147
C	3.136487	2.552117	0.691806
O	2.731154	2.976141	-0.585473
C	1.452492	3.554452	-0.669914
C	0.381282	2.850200	0.179104
H	-0.585273	3.297895	-0.096194
H	0.548005	3.083967	1.240974
C	0.286427	1.383672	-0.035734
H	-0.362975	1.057213	-0.853958
H	1.175514	3.519165	-1.732616
H	1.491338	4.614337	-0.357772
H	2.729199	3.192890	1.489088
H	4.232534	2.643250	0.733403
C	0.259877	-1.882902	1.537291
C	-0.340070	-3.040019	0.736354
H	-0.210516	-4.007799	1.240183
C	0.370752	-2.951648	-0.615158
H	-0.168135	-3.470683	-1.419575
H	1.387624	-3.372145	-0.552290
H	-1.414659	-2.857233	0.587544
H	-0.478045	-1.427442	2.212406
H	1.126796	-2.212544	2.137885
H	1.255036	-1.133480	-1.513843

H	-0.514339	-1.081890	-1.264877
C	-4.756552	0.346692	-0.123500
F	-4.727439	1.651795	0.146508
F	-5.416880	-0.259635	0.862003
F	-5.450951	0.176893	-1.247438
S	-3.040539	-0.333934	-0.289329
O	-2.449632	-0.055347	1.025664
O	-2.492352	0.455911	-1.405499
O	-3.271741	-1.751672	-0.591614
C	3.557759	0.068392	0.293574
C	3.543117	-1.268827	0.743187
C	4.292649	0.376364	-0.878662
C	4.219173	-2.262986	0.041046
H	3.045803	-1.533120	1.674588
C	4.952796	-0.619346	-1.577814
H	4.301528	1.397292	-1.261274
C	4.912575	-1.943892	-1.123289
H	4.204518	-3.290556	0.409337
H	5.500546	-0.370538	-2.488869
H	5.435831	-2.725821	-1.678403

SI.TS-10-diastereomer1

ZPE	0.422097
DE	0.442411
DH	0.443355
DG	0.372690
E	-943.666046
H	-943.222690
Gibbs free energy	-943.293355
Imaginary frequency	-334.264

Cartesian coordinates

C	-0.340414	-1.252085	-1.513941
N	-0.909462	-0.568196	-0.330237
C	-0.686612	3.252660	-0.325734
C	0.192547	1.006004	1.768680
H	-1.954690	1.754558	-1.389816
H	-0.276090	1.913805	2.153826
C	0.677789	3.593980	-0.943101

C	1.956154	2.420916	0.703933
O	1.702389	2.678404	-0.659995
C	1.384768	1.073600	1.104982
C	-0.822327	0.702419	-0.130670
H	-1.411659	3.979509	-0.716083
H	-0.649859	3.408277	0.762635
C	-1.168726	1.874692	-0.631258
H	-0.187100	0.070169	2.181431
H	0.597821	3.617183	-2.038856
H	0.955595	4.609197	-0.603442
H	1.549199	3.213352	1.351618
H	3.046776	2.411637	0.850178
C	-1.730370	-1.481781	0.502588
C	-1.911872	-2.690157	-0.416326
H	-2.083716	-3.608347	0.160478
C	-0.625129	-2.723280	-1.238574
H	-0.723187	-3.297059	-2.169256
H	0.195456	-3.160353	-0.651183
H	-2.782663	-2.518895	-1.066725
H	-1.141630	-1.763072	1.392259
H	0.724528	-1.012096	-1.616443
H	-0.879144	-0.888468	-2.403626
C	2.113031	-0.138606	0.680582
C	3.055295	-0.073690	-0.363049
C	1.859838	-1.386189	1.275684
C	3.697702	-1.222956	-0.810927
H	3.255943	0.876573	-0.859620
C	2.515236	-2.532827	0.835501
H	1.159154	-1.466948	2.108346
C	3.429146	-2.456733	-0.214861
H	4.413668	-1.156162	-1.632913
H	2.310887	-3.491450	1.317228
H	3.939386	-3.357081	-0.564429
C	-3.007639	-0.806956	0.961055
H	-2.765677	0.044384	1.628682
H	-3.589719	-1.533021	1.561968
O	-3.718967	-0.372449	-0.158909
C	-4.864642	0.368066	0.157298
H	-5.357394	0.645705	-0.784493
H	-5.576959	-0.217031	0.768893

H	-4.612725	1.292903	0.711064

SI.TS-10-diastereomer2

ZPE	0.422256
DE	0.442414
DH	0.443358
DG	0.373550
E	-943.668497
H	-943.225140
Gibbs free energy	-943.294947
Imaginary frequency	-330.344

Cartesian coordinates

C	-0.830731	1.691171	-2.133972
N	-0.111033	1.089960	-0.994231
C	3.141361	-0.920499	-0.882747
C	0.246885	-1.557880	-2.015407
H	2.617701	1.246135	-0.740038
H	1.111471	-1.888301	-2.594373
C	3.100436	-1.689924	0.445934
C	1.091136	-2.883246	-0.065546
O	1.819315	-2.094839	0.852252
C	0.026934	-2.054301	-0.761180
C	0.905564	0.304297	-1.127100
H	2.933706	-1.607968	-1.714408
H	4.170566	-0.563423	-1.023180
C	2.210928	0.242573	-0.925276
H	-0.542332	-1.065728	-2.585956
H	3.774229	-2.561848	0.353263
H	3.481201	-1.056577	1.259354
H	0.606310	-3.694934	0.497515
H	1.752224	-3.350822	-0.812541
C	-0.622540	1.609966	0.311574
C	-1.313581	2.900154	-0.119201
H	-2.062873	3.214271	0.619483
C	-1.916077	2.537118	-1.474401
H	-2.173043	3.412626	-2.084374
H	-2.828731	1.938190	-1.333761
H	-0.565097	3.700127	-0.223311

H	-1.374441	0.905361	0.694925
H	-1.225251	0.906300	-2.794769
H	-0.116827	2.301200	-2.708197
C	-1.197574	-1.727651	-0.007176
C	-1.195652	-1.768938	1.399352
C	-2.366515	-1.307590	-0.665782
C	-2.312448	-1.359500	2.120756
H	-0.297063	-2.078523	1.934767
C	-3.485929	-0.907833	0.058272
H	-2.413496	-1.305310	-1.756218
C	-3.457663	-0.920324	1.453497
H	-2.287824	-1.377494	3.212404
H	-4.386799	-0.587738	-0.469583
H	-4.333683	-0.599778	2.021817
C	0.461313	1.740706	1.355150
H	0.909582	0.747773	1.560496
H	-0.017458	2.076916	2.295595
O	1.432581	2.649104	0.922810
C	2.471444	2.831331	1.845586
H	2.097334	3.226480	2.808486
H	3.010094	1.885894	2.048418
H	3.178838	3.555552	1.419314

SI.TS-10-diastereomer3

ZPE	0.421914
DE	0.442279
DH	0.443223
DG	0.372966
E	-943.663940
H	-943.220716
Gibbs free energy	-943.290973
Imaginary frequency	-286.288

Cartesian coordinates

C	1.577662	2.130432	-0.645937
N	0.552228	1.093954	-0.970325
C	-3.142215	2.030187	-0.347452
C	-1.043814	0.358548	1.342347
H	-1.643405	2.393056	-1.944340

H	-1.824108	1.025700	1.709383
C	-4.058229	0.806617	-0.254570
C	-2.829692	-1.124458	0.425954
O	-3.722974	-0.110042	0.757385
C	-1.390708	-0.825401	0.762139
C	-0.692188	1.229897	-0.651398
H	-3.016809	2.453333	0.661552
H	-3.684880	2.785672	-0.932302
C	-1.799733	1.858424	-0.996095
H	-0.018844	0.535002	1.670498
H	-5.065524	1.173460	-0.009391
H	-4.119266	0.297890	-1.233549
H	-2.913984	-1.385697	-0.644543
H	-3.114340	-2.030467	0.989504
C	1.110206	0.036462	-1.837350
C	2.302444	0.734003	-2.476874
H	3.039191	0.013591	-2.855600
C	2.835419	1.593621	-1.332703
H	3.484434	2.413527	-1.667106
H	3.397902	0.971963	-0.620270
H	1.970556	1.364908	-3.316064
H	1.443667	-0.810014	-1.221547
H	1.241696	3.068080	-1.118357
C	-0.384932	-1.860938	0.447431
C	-0.573009	-2.755129	-0.620574
C	0.806416	-1.940533	1.189363
C	0.421363	-3.666227	-0.964755
H	-1.483300	-2.721378	-1.220974
C	1.796408	-2.854913	0.846623
H	0.960336	-1.281647	2.044387
C	1.612235	-3.712212	-0.239452
H	0.267912	-4.340900	-1.809675
H	2.717699	-2.899589	1.431365
H	2.393251	-4.425143	-0.513220
H	0.340279	-0.309174	-2.539319
C	1.720883	2.391117	0.840501
H	2.477554	3.189089	0.969786
H	0.768370	2.780163	1.254110
O	2.107299	1.216270	1.491477
C	2.397772	1.393851	2.852805

H	1.517006	1.755708	3.416013
H	2.703194	0.420952	3.261089
H	3.223478	2.114329	2.999918

SI.TS-10-diastereomer4

ZPE	0.422796
DE	0.442745
DH	0.443689
DG	0.375625
E	-943.665307
H	-943.221618
Gibbs free energy	-943.289682
Imaginary frequency	-305.763

Cartesian coordinates

C	0.672718	2.005221	0.899986
N	0.035316	1.765696	-0.424224
C	-3.614882	0.494688	-0.482027
C	-0.928969	-0.542099	-1.949126
H	-2.595994	2.405320	-0.013612
H	-1.969809	-0.541093	-2.271097
C	-3.595338	-0.893734	0.157622
C	-1.594021	-2.161863	-0.194302
O	-2.903035	-1.874356	-0.575581
C	-0.542384	-1.407753	-0.972338
C	-1.121070	1.219599	-0.575981
H	-4.457138	1.040760	-0.034335
H	-3.862820	0.392514	-1.552687
C	-2.407710	1.372041	-0.341434
H	-0.208541	-0.061212	-2.612784
H	-3.208536	-0.832535	1.189937
H	-4.640273	-1.234000	0.217317
H	-1.428063	-3.241907	-0.350225
H	-1.441650	-1.958584	0.879925
C	0.759779	2.445523	-1.517234
C	1.624949	3.456297	-0.775353
H	2.514419	3.736110	-1.354369
C	1.961472	2.739075	0.531136
H	2.278275	3.421524	1.330256

H	2.768581	2.006083	0.368295
H	1.045680	4.369733	-0.571903
H	1.369578	1.709208	-2.060968
H	-0.009001	2.676910	1.448466
C	0.871272	-1.649844	-0.618503
C	1.212257	-2.458107	0.481958
C	1.917754	-1.070929	-1.357883
C	2.542435	-2.656710	0.839520
H	0.439116	-2.931873	1.086213
C	3.247629	-1.262125	-0.995749
H	1.699494	-0.471197	-2.241487
C	3.565498	-2.052014	0.109331
H	2.780974	-3.284165	1.700929
H	4.040682	-0.797582	-1.585735
H	4.608818	-2.203797	0.395005
H	0.038219	2.889941	-2.214679
C	0.915802	0.739171	1.710480
H	1.628385	0.099550	1.172646
H	1.390267	1.048139	2.661917
O	-0.225077	-0.033218	1.944577
C	-1.128581	0.487064	2.882847
H	-0.618373	0.772265	3.821147
H	-1.674888	1.369218	2.501321
H	-1.861386	-0.299625	3.110402

SI.TS-11-diastereomer1

ZPE	0.500337
DE	0.533157
DH	0.534102
DG	0.434884
E	-2037.236266
H	-2036.702164
Gibbs free energy	-2036.801382
Imaginary frequency	-311.916

Cartesian coordinates

C	-3.005923	0.428729	0.493039
N	-1.691906	0.390277	-0.242277
C	-0.626575	0.950642	0.226031

C	0.352986	2.168085	-1.344504
H	0.067312	1.513861	-2.171826
H	1.381406	2.065256	-0.991257
C	-0.410069	3.249543	-1.035608
C	0.021954	4.263458	-0.000641
O	-0.586801	3.960702	1.240540
C	0.259492	3.411388	2.218391
C	1.005459	2.141065	1.794877
H	1.575389	1.778021	2.659534
H	1.758542	2.368596	1.025477
C	0.118974	1.046448	1.312261
H	0.044920	0.134596	1.918209
H	-0.386977	3.198404	3.081805
H	1.014700	4.153446	2.537729
H	1.119814	4.297129	0.084242
H	-0.321703	5.264049	-0.307905
C	-1.762186	-0.381893	-1.484886
C	-3.228995	-0.764710	-1.553616
C	4.758809	-0.928578	-0.432108
F	4.605481	-2.249502	-0.372263
F	5.645997	-0.573687	0.494937
F	5.265175	-0.625578	-1.625348
S	3.140964	-0.068157	-0.162105
O	2.748461	-0.521580	1.180184
O	2.312621	-0.573010	-1.265680
O	3.500379	1.355607	-0.259618
C	-0.810055	-1.582918	-1.538333
H	0.222291	-1.206345	-1.586207
H	-1.026254	-2.100555	-2.485531
C	-3.483351	1.861210	0.697767
H	-2.679286	2.471680	1.134784
H	-3.792119	2.303915	-0.257273
H	-4.338733	1.875734	1.387352
C	-2.886590	-0.312275	1.821430
H	-2.261756	0.256395	2.523537
H	-3.882883	-0.408129	2.275107
H	-2.462037	-1.315225	1.678513
H	-1.554938	0.281289	-2.339771
C	-0.959343	-2.519385	-0.362835
C	-0.013676	-2.505015	0.669873

C	-2.061962	-3.378693	-0.253605
C	-0.183111	-3.307463	1.799276
H	0.865671	-1.860096	0.591849
C	-2.231300	-4.180739	0.874319
H	-2.796731	-3.418433	-1.061779
C	-1.295678	-4.141533	1.909653
H	0.564825	-3.280258	2.595819
H	-3.097873	-4.843007	0.943379
H	-1.428692	-4.768541	2.794671
O	-3.735091	-1.396307	-2.460778
C	-5.256521	-0.511099	-0.202192
H	-5.412038	-1.166840	0.667880
H	-5.779316	0.441692	-0.033917
H	-5.678804	-0.997394	-1.089880
N	-3.850706	-0.294830	-0.449914
C	-1.728391	3.500973	-1.687546
H	-2.152810	2.593733	-2.140891
H	-2.448401	3.938544	-0.982170
H	-1.580781	4.243023	-2.491850

SI.TS-11-diastereomer2

ZPE	0.499778
DE	0.532818
DH	0.533762
DG	0.433304
E	-2037.231151
H	-2036.697389
Gibbs free energy	-2036.797846
Imaginary frequency	-357.517

Cartesian coordinates

C	1.099941	-0.973315	1.731472
N	0.980763	-0.024192	0.576188
C	1.078284	1.262275	0.733758
C	-0.876184	2.768240	-0.282778
C	-4.532533	-0.080545	-0.119403
F	-5.123337	-0.318476	1.049616
C	-0.903464	2.021951	0.863960
F	-4.096658	1.181752	-0.106391

S	-3.132415	-1.262193	-0.410510
O	-2.612466	-0.836306	-1.718853
O	-3.777657	-2.571708	-0.376909
O	-2.236211	-0.972881	0.726323
C	1.584540	-0.472082	-1.825059
H	1.521092	0.578263	-2.146086
H	1.212928	-1.088136	-2.657911
C	0.129715	-0.612404	2.849983
H	0.254536	-1.314997	3.686035
H	0.350461	0.395941	3.229134
H	-0.904133	-0.660095	2.484915
C	2.546936	-0.993658	2.219374
H	3.232223	-1.223389	1.391056
H	2.816774	-0.022936	2.658532
H	2.665725	-1.755993	3.002454
H	-0.403023	-0.365266	-0.972043
C	3.011009	-0.825806	-1.480604
C	3.411222	-2.163166	-1.349971
C	3.952170	0.179099	-1.230217
C	4.708482	-2.482950	-0.951940
H	2.695147	-2.961264	-1.560528
C	5.251603	-0.137095	-0.831540
H	3.661011	1.226140	-1.350979
C	5.631559	-1.470822	-0.683506
H	5.001118	-3.531068	-0.853040
H	5.970853	0.662907	-0.639321
H	6.647584	-1.722168	-0.370067
O	0.157660	-3.050706	-0.979444
C	0.573883	-3.453516	1.816612
H	1.445501	-3.653245	2.456058
H	-0.334879	-3.425878	2.437896
H	0.473646	-4.267429	1.088055
N	0.742765	-2.225336	1.079221
C	0.604686	-0.687837	-0.668798
F	-5.451891	-0.184132	-1.075670
H	-1.380898	1.037596	0.843216
H	-0.800378	2.511490	1.837796
C	0.462138	-2.136266	-0.239396
C	-0.426896	4.216147	-0.293287
O	0.899412	4.295399	-0.765538

C	1.870878	4.574860	0.211367
C	1.854848	3.610707	1.400294
H	2.703386	3.872361	2.048867
H	0.945863	3.761480	1.997628
C	1.988923	2.180105	1.001904
H	3.004281	1.777099	0.895015
H	2.838758	4.524208	-0.308179
H	1.751338	5.603503	0.600378
H	-0.541085	4.675203	0.701583
H	-1.071720	4.774493	-0.990507
C	-1.220947	2.211734	-1.618795
H	-1.951720	2.875857	-2.108284
H	-0.321955	2.224762	-2.259001
H	-1.632178	1.195735	-1.575582

SI.TS-11-diastereomer3

ZPE	0.500795
DE	0.533477
DH	0.534421
DG	0.434867
E	-2037.220446
H	-2036.686025
Gibbs free energy	-2036.785579
Imaginary frequency	-275.815

Cartesian coordinates

C	0.463296	-0.212404	2.402878
N	0.735620	-0.239343	0.918447
C	0.622829	0.816301	0.170015
C	2.861118	2.163437	0.054053
C	-4.145537	0.529690	-1.318605
F	-4.620587	0.134416	-2.496934
C	2.486547	1.281121	-0.910021
F	-5.179950	0.817357	-0.532034
S	-3.073127	-0.779717	-0.558085
O	-3.987988	-1.903919	-0.384781
O	-2.623990	-0.129410	0.689907
O	-2.014562	-0.949805	-1.564850
C	1.717218	-2.513752	0.174917

H	1.281683	-3.505439	-0.029308
H	2.279573	-2.606782	1.115150
C	-0.250869	1.060764	2.824583
H	-0.399429	1.042462	3.913134
H	0.360229	1.941389	2.582249
H	-1.228026	1.141920	2.331480
C	1.743557	-0.416671	3.211929
H	1.476764	-0.642406	4.254490
H	2.335411	-1.253988	2.819135
H	2.354693	0.492649	3.211897
H	-0.081123	-1.497942	-0.544684
C	2.650152	-2.137944	-0.947454
C	2.171005	-1.953677	-2.251718
C	4.015867	-1.956816	-0.702799
C	3.033127	-1.572008	-3.277338
H	1.108569	-2.098211	-2.466064
C	4.882639	-1.576969	-1.729323
H	4.403676	-2.101981	0.309299
C	4.391747	-1.375944	-3.018426
H	2.641987	-1.425767	-4.287044
H	5.944830	-1.432542	-1.517635
H	5.065741	-1.072314	-3.822863
O	-0.987015	-3.258891	1.326656
C	-1.224520	-1.630817	3.675781
H	-2.070607	-0.928383	3.718740
H	-1.620845	-2.650365	3.591988
H	-0.639390	-1.552701	4.604089
N	-0.399753	-1.386545	2.518926
C	0.507016	-1.598206	0.379034
F	-3.457803	1.659027	-1.514570
H	2.844704	0.254253	-0.867481
H	2.062874	1.615746	-1.859081
C	-0.404986	-2.199333	1.441479
C	2.527720	3.640460	-0.009468
O	1.370283	3.916720	0.749185
C	0.198398	4.132333	0.005438
C	-0.163537	2.992337	-0.956123
H	-1.132455	3.235011	-1.411857
H	0.565246	2.947938	-1.777348
C	-0.270937	1.680472	-0.266258

H	-1.272185	1.302704	-0.003248
H	-0.603428	4.269451	0.744601
H	0.278200	5.064246	-0.584833
H	2.418370	3.972625	-1.054691
H	3.360526	4.208981	0.433759
C	3.645405	1.732233	1.244645
H	3.239795	2.177649	2.166184
H	3.696271	0.639941	1.338425
H	4.674995	2.116136	1.138863

SI.TS-11-diastereomer4

ZPE	0.499783
DE	0.532821
DH	0.533765
DG	0.433716
E	-2037.229596
H	-2036.695831
Gibbs free energy	-2036.795880
Imaginary frequency	-268.808

Cartesian coordinates

C	-0.079406	2.735112	0.847624
N	-0.485369	1.366152	0.381437
C	-0.624498	0.355835	1.172860
C	-3.236322	0.255207	1.645926
C	3.906766	-1.803562	0.407965
F	3.060502	-2.317197	1.305561
C	-2.384381	0.597996	2.641197
F	4.372315	-2.804404	-0.333317
S	3.058920	-0.533925	-0.645264
O	1.972422	-1.308639	-1.269367
O	4.112508	-0.069521	-1.541674
O	2.591271	0.447693	0.353277
C	-1.398743	1.080779	-2.010267
H	-0.970712	1.258024	-3.011618
H	-2.104025	1.906545	-1.830045
C	0.713607	2.644218	2.141335
H	0.088429	2.240534	2.949224
H	1.592685	2.001201	1.997814

H	1.039608	3.649076	2.443379
C	-1.294901	3.652836	0.966124
H	-0.958468	4.680066	1.165803
H	-1.872450	3.649146	0.031202
H	-1.946220	3.348989	1.794300
H	0.431369	0.298883	-1.165772
C	-2.125843	-0.246701	-2.034833
C	-3.504095	-0.274139	-2.283247
C	-1.449689	-1.466355	-1.893864
C	-4.193821	-1.481719	-2.387551
H	-4.045498	0.668587	-2.399752
C	-2.138315	-2.675447	-2.001254
H	-0.372994	-1.485570	-1.702660
C	-3.511637	-2.690004	-2.245606
H	-5.269375	-1.477249	-2.580343
H	-1.592144	-3.615520	-1.891880
H	-4.047244	-3.638762	-2.325844
O	1.192367	2.578226	-2.451433
C	1.601907	4.323474	-0.212468
H	1.967776	4.520716	-1.227573
H	1.042337	5.202425	0.139880
H	2.466777	4.155921	0.447219
N	0.756905	3.155058	-0.272555
C	-0.213559	1.182692	-1.061491
F	4.927719	-1.268183	1.071061
H	-2.204644	1.645520	2.886982
H	-2.035189	-0.148635	3.354499
C	0.667392	2.380618	-1.374279
C	-3.616166	-1.177354	1.394855
O	-3.010083	-2.100794	2.243739
C	-1.814993	-2.702089	1.808779
C	-0.531531	-2.007532	2.264928
H	0.280791	-2.742017	2.174589
H	-0.606017	-1.774547	3.341058
C	-0.081749	-0.786746	1.527912
H	0.969620	-0.750860	1.199545
H	-1.814085	-2.815163	0.711300
H	-1.806351	-3.712052	2.245208
H	-4.708965	-1.252560	1.540696
H	-3.433712	-1.413217	0.329223

C	-3.860395	1.271157	0.747678
H	-4.136289	2.180619	1.298220
H	-3.139847	1.565425	-0.032373
H	-4.749105	0.871486	0.239753

TS-6 – 2-fluoropyridine complex

ZPE	0.417870
DE	0.447393
DH	0.448337
DG	0.352803
E	-1906.502466
H	-1906.054129
Gibbs free energy	-1906.149663
Imaginary frequency	-349.620

Cartesian coordinates

C	-0.162759	-1.931594	1.633353
N	0.876988	-0.914284	1.351730
C	1.141921	-0.504564	0.153530
C	3.207322	-0.728940	-0.245141
H	3.517012	-0.550645	0.787769
H	3.398649	0.083021	-0.950787
C	2.958897	-1.996110	-0.684340
C	2.733321	-2.319629	-2.145319
O	1.353183	-2.466468	-2.399335
C	0.729968	-1.380784	-3.035792
C	0.931859	-0.023028	-2.347092
H	0.326263	0.715275	-2.890170
H	1.980426	0.295006	-2.451601
C	0.532550	-0.012916	-0.913912
H	-0.417326	0.471050	-0.649861
H	-0.340598	-1.625788	-3.063220
H	1.084787	-1.287693	-4.079652
H	3.192515	-1.557255	-2.795829
H	3.217846	-3.283407	-2.369860
C	1.521571	-0.439209	2.585927
C	0.622821	-0.997928	3.684291
H	1.166038	-1.134920	4.628625
C	0.104902	-2.307410	3.083778

H	-0.799343	-2.682721	3.581100
H	0.878685	-3.089594	3.143092
H	-0.216155	-0.308938	3.866502
H	1.599975	0.655711	2.568612
H	2.537269	-0.866605	2.646766
H	-0.083323	-2.759386	0.917587
H	-1.138893	-1.439196	1.517360
C	-3.479332	-0.826299	-0.400643
F	-4.432911	-0.828036	-1.328284
F	-3.930528	-1.501404	0.656164
F	-2.432942	-1.498150	-0.892050
S	-3.005351	0.908687	0.064637
O	-2.455874	1.428489	-1.197785
O	-2.005170	0.700313	1.126072
O	-4.269171	1.500566	0.499344
C	2.845432	-3.148498	0.256042
H	3.724436	-3.802146	0.122272
H	2.810638	-2.835354	1.307852
H	1.959557	-3.756069	0.016916
C	0.318789	2.974102	0.754527
C	0.129651	3.340829	-0.573047
C	1.243600	3.387467	-1.413085
C	2.495106	3.054088	-0.905302
C	2.541804	2.693580	0.437502
H	-0.875474	3.542090	-0.942585
H	3.401382	3.063783	-1.511838
N	1.519738	2.659647	1.253498
H	-0.532383	2.893916	1.435045
H	1.137443	3.669296	-2.462970
F	3.717584	2.331134	0.961955

TS-6 - collidine complex

ZPE	0.508364
DE	0.542137
DH	0.543081
DG	0.439532
E	-1925.211685
H	-1924.668604
Gibbs free energy	-1924.772153
Imaginary frequency	-279.392

Cartesian coordinates

C	4.353581	-1.805796	0.370689
N	3.329375	-0.865033	-0.144507
C	2.235390	-0.608298	0.484051
C	0.528573	-1.052222	-0.893101
H	1.113245	-0.795379	-1.780043
H	-0.236907	-0.337896	-0.586612
C	0.538790	-2.315692	-0.396798
C	-0.391205	-2.728288	0.721799
O	0.325056	-2.792366	1.941069
C	0.118052	-1.720292	2.821546
C	0.388544	-0.329354	2.229461
H	0.271030	0.402438	3.040655
H	-0.390192	-0.093262	1.489081
C	1.739702	-0.168534	1.624887
H	2.486797	0.424647	2.170390
H	0.785091	-1.905675	3.676299
H	-0.921697	-1.720116	3.199480
H	-1.250221	-2.041421	0.781965
H	-0.776398	-3.739146	0.510361
C	3.729352	-0.280743	-1.437894
C	5.219330	-0.588846	-1.504001
H	5.586635	-0.610480	-2.538242
C	5.323594	-1.942699	-0.796700
H	6.340326	-2.178624	-0.456163
H	4.993692	-2.749028	-1.470247
H	5.789384	0.176777	-0.954343
H	3.457971	0.783874	-1.464950
H	3.183248	-0.803131	-2.241705
H	3.881915	-2.744677	0.685120
H	4.830155	-1.338294	1.247164
C	-4.552635	-0.886036	-0.399033
F	-4.093721	-2.136060	-0.328214
F	-5.310751	-0.788437	-1.488401
F	-5.325419	-0.672190	0.663218
S	-3.150200	0.323704	-0.456706
O	-3.820014	1.627317	-0.497092
O	-2.435050	-0.060725	-1.680008

O	-2.436288	0.034570	0.802098
C	1.469662	-3.369590	-0.901201
H	1.925549	-3.917855	-0.062919
H	0.894128	-4.105314	-1.488422
H	2.256203	-2.958544	-1.549227
C	2.187790	2.864466	0.356945
C	1.188356	3.101341	1.303578
C	-0.155479	2.970598	0.941302
C	-0.427573	2.620955	-0.382206
C	0.623296	2.397773	-1.274537
H	1.461186	3.365607	2.328517
H	-1.459608	2.502190	-0.716836
N	1.903833	2.518596	-0.904158
C	0.335684	2.003119	-2.696120
H	0.135181	2.897245	-3.309629
H	1.197602	1.483576	-3.137286
H	-0.553530	1.357530	-2.745961
C	-1.276401	3.162736	1.921145
H	-1.978625	2.318358	1.852083
H	-0.907457	3.246328	2.953091
H	-1.843648	4.077362	1.685242
C	3.644003	2.911873	0.728454
H	4.046311	1.886672	0.797798
H	4.224389	3.436725	-0.044419
H	3.805940	3.406305	1.696124

2 – linear conformer

ZPE	0.305818
DE	0.328863
DH	0.329808
DG	0.249131
E	-1520.050031
H	-1519.720224
Gibbs free energy	-1519.800901

Cartesian coordinates

C	3.369817	1.898457	-0.967647
C	4.597678	1.254511	-0.329977
C	4.012827	0.341132	0.749375

C	2.889090	1.182561	1.332932
H	3.514790	2.932370	-1.303952
H	2.953352	1.287742	-1.778188
H	5.241399	2.022955	0.124738
H	5.189794	0.709856	-1.076532
H	3.587480	-0.565540	0.296664
H	4.742485	0.052833	1.516839
H	2.081786	0.620835	1.813361
H	3.264574	1.954512	2.021189
N	2.357931	1.879281	0.131256
C	1.171152	2.242600	-0.006908
C	-0.050135	2.650842	-0.160472
H	-0.240543	3.692904	0.131529
C	-1.184878	1.817450	-0.686690
H	-1.549345	2.291906	-1.611982
H	-0.845041	0.803824	-0.933280
C	-2.328911	1.755210	0.315702
H	-1.987038	1.264330	1.247754
H	-2.657469	2.779056	0.590688
O	-3.372809	1.036713	-0.270536
C	-4.442933	0.777354	0.601667
H	-4.070252	0.256761	1.509014
H	-4.918694	1.718917	0.941940
C	-5.439265	-0.096457	-0.094775
H	-5.021678	-0.995322	-0.564384
C	-6.749222	0.141369	-0.144064
H	-7.186964	1.035751	0.313243
H	-7.436428	-0.551782	-0.637818
C	0.406257	-1.775061	0.863581
S	0.700763	-1.706274	-0.969633
O	1.380496	-0.406999	-1.128166
O	1.543026	-2.877525	-1.212406
O	-0.655662	-1.755482	-1.521770
F	-0.338405	-2.825493	1.193822
F	-0.223571	-0.675950	1.294168
F	1.556509	-1.862995	1.536587

6. References

1. Stang, P. J.; Dueber., T. E., Preparation of vinyl trifluoromethanesulfonates: 3-methyl-2-buten-2-yl triflate. *Org. Synth.* **1974,** *54*, 79.

2. Bruker SAINT v8.38B Copyright © 2005-2019 Bruker AXS.
3. Sheldrick, G. M. (1996). *SADABS.* University of Göttingen, Germany.
4. Dolomanov, O. V.; Bourhis, L. J.; Gildea, R. J.; Howard, J. A. K.; Puschmann, H., OLEX2: a complete structure solution, refinement and analysis program. *J. Appl. Cryst.* **2009,** *42* (2), 339-341.
5. Hubschle, C. B.; Sheldrick, G. M.; Dittrich, B., ShelXle: a Qt graphical user interface for SHELXL. *J. Appl. Cryst.* **2011,** *44* (6), 1281-1284.
6. Sheldrick, G. M. (2015). *SHELXS v 2016/4* University of Göttingen, Germany.
7. Sheldrick, G. M. (2015). *SHELXL v 2016/4* University of Göttingen, Germany.
8. Spek, A. L., Structure validation in chemical crystallography. *Acta Cryst.* **2009,** *D65* (2), 148-155.
9. Wei, W.-X.; Li, Y.; Wen, Y.-T.; Li, M.; Li, X.-S.; Wang, C.-T.; Liu, H.-C.; Xia, Y.; Zhang, B.-S.; Jiao, R.-Q.; Liang, Y.-M., Experimental and Computational Studies of Palladium-Catalyzed Spirocyclization via a Narasaka–Heck/C(sp3 or sp2)–H Activation Cascade Reaction. *J. Am. Chem. Soc.* **2021,** *143* (20), 7868-7875.
10. Madelaine, C.; Valerio, V.; Maulide, N., Unexpected Electrophilic Rearrangements of Amides: A Stereoselective Entry to Challenging Substituted Lactones. *Angew. Chem. Int. Ed.* **2010,** *49* (9), 1583-1586.
11. Takagi, K.; Fukuda, H.; Shuto, S.; Otaka, A.; Arisawa, M., Safe Removal of the Allyl Protecting Groups of Allyl Esters using a Recyclable, Low-Leaching and Ligand-Free Palladium Nanoparticle Catalyst. *Adv. Synth. Catal.* **2015,** *357* (9), 2119-2124.
12. Boschi, F. New routes to enantioenriched substances through small organic molecules. Alma Mater Studiorum Università di Bologna, Bologna, 2009.
13. Bannwarth, C.; Ehlert, S.; Grimme, S., GFN2-xTB-An Accurate and Broadly Parametrized Self-Consistent Tight-Binding Quantum Chemical Method with Multipole Electrostatics and Density-Dependent Dispersion Contributions. *J. Chem. Theory. Comput.* **2019,** *15* (3), 1652-1671.
14. Pracht, P.; Bohle, F.; Grimme, S., Automated exploration of the low-energy chemical space with fast quantum chemical methods. *Phys. Chem. Chem. Phys.* **2020,** *22* (14), 7169-7192.
15. Zimmerman, P. M., Growing string method with interpolation and optimization in internal coordinates: method and examples. *J. Chem. Phys.* **2013,** *138* (18), 184102.
16. Chai, J. D.; Head-Gordon, M., Long-range corrected hybrid density functionals with damped atom-atom dispersion corrections. *Phys. Chem. Chem. Phys.* **2008,** *10* (44), 6615-6620.
17. Weigend, F.; Ahlrichs, R., Balanced basis sets of split valence, triple zeta valence and quadruple zeta valence quality for H to Rn: Design and assessment of accuracy. *Phys. Chem. Chem. Phys.* **2005,** *7* (18), 3297-3305.
18. Marenich, A. V.; Cramer, C. J.; Truhlar, D. G., Universal solvation model based on solute electron density and on a continuum model of the solvent defined by the bulk dielectric constant and atomic surface tensions. *J. Phys. Chem. B* **2009,** *113* (18), 6378-6396.
19. Luchini, G.; Alegre-Requena, J. V.; Funes-Ardoiz, I.; Paton, R. S., GoodVibes: automated thermochemistry for heterogeneous computational chemistry data. *F1000Research* **2020,** *9*.
20. Li, Y.-P.; Gomes, J.; Mallikarjun Sharada, S.; Bell, A. T.; Head-Gordon, M., Improved Force-Field Parameters for QM/MM Simulations of the Energies of Adsorption for Molecules in Zeolites and a Free Rotor Correction to the Rigid Rotor Harmonic Oscillator Model for Adsorption Enthalpies. *J. Phys. Chem. C.* **2015,** *119* (4), 1840-1850.
21. Grimme, S., Supramolecular binding thermodynamics by dispersion-corrected density functional theory. *Chem. Eur. J.* **2012,** *18* (32), 9955-9964.
22. CYLview20; Legault, C. Y., Université de Sherbrooke, 2020. http://www.cylview.org/ (accessed 2022-10-31).
